# Sphenodontian phylogeny and the impact of model choice in Bayesian morphological clock estimates of divergence times and evolutionary rates

**DOI:** 10.1186/s12915-020-00901-5

**Published:** 2020-12-07

**Authors:** Tiago R. Simões, Michael W. Caldwell, Stephanie E. Pierce

**Affiliations:** 1 Museum of Comparative Zoology & Department of Organismic and Evolutionary Biology, Harvard University, Cambridge, MA 02138 USA; 2 Department of Biological Sciences, University of Alberta, Edmonton, Alberta T6G 2E9 Canada; 3 Department of Earth and Atmospheric Sciences, University of Alberta, Edmonton, Alberta T6G 2E9 Canada

**Keywords:** Phylogenetics, Macroevolution, Morphological clocks, Bayesian inference, Prior models, Divergence times, Evolutionary rates, Sphenodontians, Sphenodon, “Living fossil”

## Abstract

**Background:**

The vast majority of all life that ever existed on earth is now extinct and several aspects of their evolutionary history can only be assessed by using morphological data from the fossil record. Sphenodontian reptiles are a classic example, having an evolutionary history of at least 230 million years, but currently represented by a single living species (*Sphenodon punctatus*). Hence, it is imperative to improve the development and implementation of probabilistic models to estimate evolutionary trees from morphological data (e.g., morphological clocks), which has direct benefits to understanding relationships and evolutionary patterns for both fossil and living species. However, the impact of model choice on morphology-only datasets has been poorly explored.

**Results:**

Here, we investigate the impact of a wide array of model choices on the inference of evolutionary trees and macroevolutionary parameters (divergence times and evolutionary rates) using a new data matrix on sphenodontian reptiles. Specifically, we tested different clock models, clock partitioning, taxon sampling strategies, sampling for ancestors, and variations on the fossilized birth-death (FBD) tree model parameters through time. We find a strong impact on divergence times and background evolutionary rates when applying widely utilized approaches, such as allowing for ancestors in the tree and the inappropriate assumption of diversification parameters being constant through time. We compare those results with previous studies on the impact of model choice to molecular data analysis and provide suggestions for improving the implementation of morphological clocks. Optimal model combinations find the radiation of most major lineages of sphenodontians to be in the Triassic and a gradual but continuous drop in morphological rates of evolution across distinct regions of the phenotype throughout the history of the group.

**Conclusions:**

We provide a new hypothesis of sphenodontian classification, along with detailed macroevolutionary patterns in the evolutionary history of the group. Importantly, we provide suggestions to avoid overestimated divergence times and biased parameter estimates using morphological clocks. Partitioning relaxed clocks offers methodological limitations, but those can be at least partially circumvented to reveal a detailed assessment of rates of evolution across the phenotype and tests of evolutionary mosaicism.

## Background

Morphology provides the only source of data to understand the relationships and broadscale evolutionary patterns across the vast majority of life that has ever existed on this planet [1]. Morphological characters and fossil data (both specimens and their ages) also contribute to improved divergence time estimates in total-evidence phylogenies of extant organisms [2, 3], providing a more holistic reconstruction of the tree of life. Additionally, it constitutes the sole source of data to investigate the phylogeny and macroevolution of most extinct lineages. Therefore, improvements on the phylogenetic analysis of morphological data and divergence time estimates for evolutionary trees are essential for the entire field of evolutionary and comparative biology.

Currently, one of the most widespread methods to time-calibrate trees and estimate macroevolutionary parameters is the utilization of relaxed molecular clocks in Bayesian phylogenetic inference. By using either a fixed tree topology, or by co-estimating tree topologies (among species relationships) with divergence times and rates of evolution, clock-based Bayesian inference has become the workhorse for time-calibrated trees in evolutionary biology [4, 5]. Importantly, advances in the last decade have also enabled the utilization of morphological data in relaxed clock Bayesian inference analysis, especially through the development of tree models (modeling diversification parameters) that take into account fossil sampling probabilities—the fossilized birth-death (FBD) tree model [6, 7]. Since then, there has been rapidly growing interest to implement relaxed clock Bayesian inference analyses with morphological data (morphological clocks). Morphological clocks have been used to estimate divergence times for entirely extinct lineages [e.g., [8–10]], or in combination with molecular data (“morpho-molecular clocks” in total-evidence dating) to estimate evolutionary trees and divergence times for clades with extant representatives [e.g., [11–15]].

Nearly all relaxed clock Bayesian inference models were initially developed for molecular sequence data to reconstruct epidemiological and/or phylogenetic trees [16, 17], being later co-opted for applications with morphological datasets. As a result, most studies assessing the impact of model choice on species relationships and macroevolutionary parameters (divergence times and evolutionary rates) have only been conducted on molecular datasets (either empirical or simulated) (e.g., [18–22]), with little to no assessment of available models on morphological data. Some exceptions include studies that have included morphological data, but combined with molecular data in total-evidence dating, which creates different phylogenetic inference problems given the interplay between morphological and molecular signals (e.g., [11, 12, 14, 15]). Importantly, several features of morphological datasets make them quite distinct from molecular datasets. Such features include the following: the greater influence of natural selection on the phenotype and its potential impact on developing realistic morphological models [23]; the lack of direct comparability of character states among different characters (as opposed to nucleotides in molecular sequences), thus hampering the development of more complex morphological substitution models [24]; and the much smaller size of the vast majority of morphological datasets compared to molecular sequences. Therefore, it is expected that the impact of model specification on tree topology, divergence times, and evolutionary rates on morphological datasets may vary substantially from molecular datasets.

Recently, some studies have started to direct their attention to the performance of morphology only datasets in a probabilistic framework, especially so as to compare the performance of software and optimality criteria under various conditions using non-clock Bayesian inference—e.g., [23, 25–28]. However, very few studies have directed their attention to the performance of model choice using relaxed Bayesian clocks with morphological data only. Some of this pioneer work assessed the impact of uncertainty in fossil ages when calibrating the morphological clock [29, 30] different software/packages on divergence time estimates (using standard parameters) [31], sampling of autapomorphies [32], and using mechanistic tree priors (e.g., fossilized birth-death tree model) instead of the uniform tree prior [33]. Yet, several parameters available for character evolution, tree models, and clock models have never been tested to assess either their individual or combined impact on tree topology, divergence times, and evolutionary rates.

Here, we follow the approach taken by previous molecular studies to assess the impact of model choice (e.g., [20–22]), but applied to a new empirical morphological data of sphenodontian reptiles. Sphenodontians are a unique lineage of diapsid reptiles, with an extremely long evolutionary history dating at least as far back as the Middle Triassic at ~ 230Mya [34], but currently represented by a single living species, *Sphenodon punctatus*, inhabiting small islands off the coast of New Zealand [35]. In contrast, their sister clade, squamates (lizards and snakes), is currently represented by ~ 10,650 extant species [36], indicating both lineages had very different evolutionary histories after their split from a common ancestor at about 260–270 Mya [13, 37]. Despite considerable efforts to understand broad scale phylogenetic relationships, divergence times and evolutionary patterns among the various families of squamates (e.g., [13, 38–41]), there has been comparatively less effort to understand the species level relationships and macroevolutionary patterns in sphenodontians. For instance, *Sphenodon punctatus* has long been characterized as a “living fossil” [42], implying a relatively conserved morphology and low rates of evolution of its lineage for several million years [43]. However, only recently have quantitative tools been used to assess such assumptions, although limited to studying skull or mandibular shape evolution, and finding contrasting results [42, 43]. Furthermore, there has been no attempt to provide a detailed assessment of divergence times for the main sphenodontian clades using relaxed clock models and the FBD model. Finally, there has never been an assessment of evolutionary rates within sphenodontians taking into account information from all body regions, nor contrasting rates of evolution among different subdivisions of their phenotype.

**Fig. 1:**
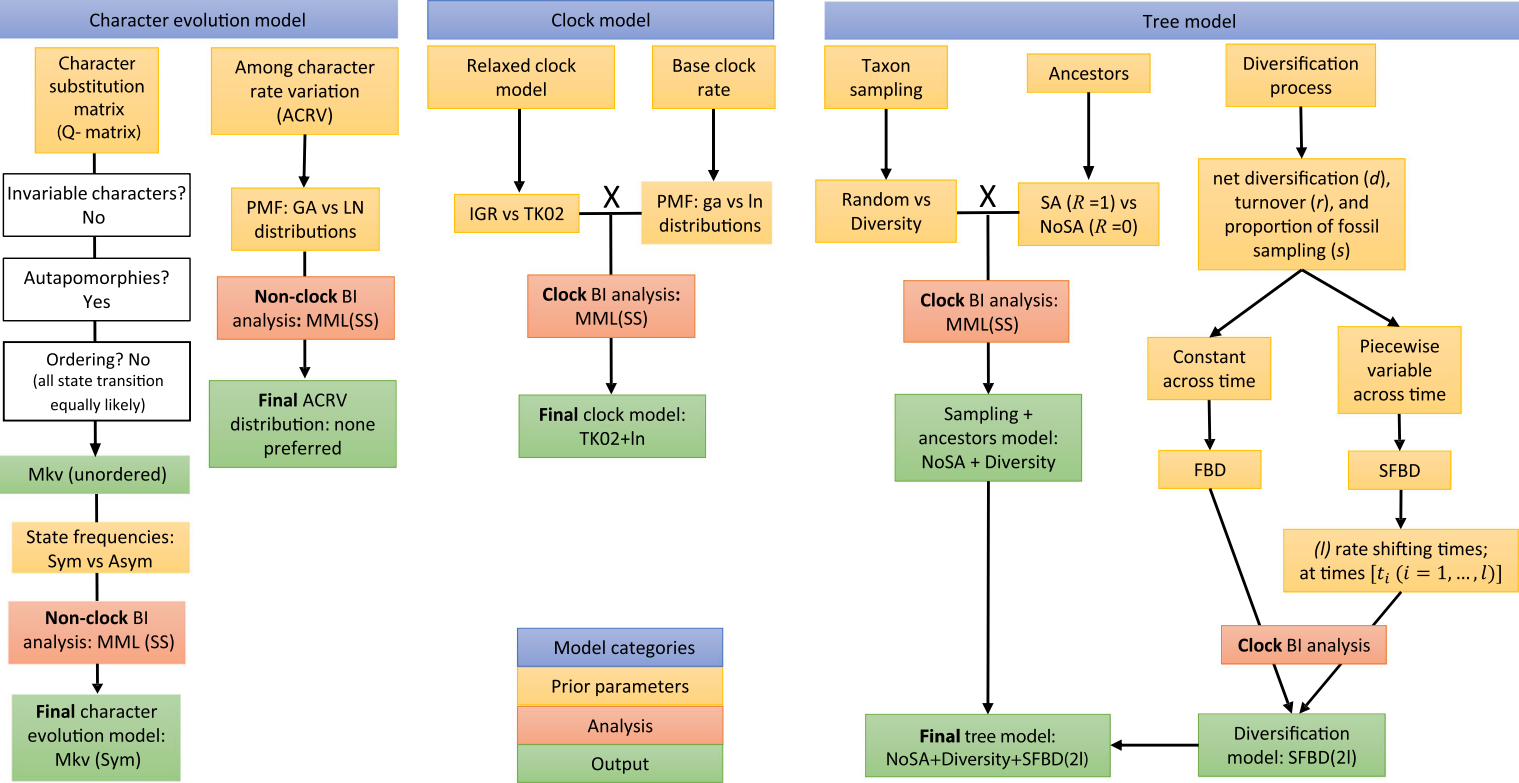
Summary of models and parameters available for morphological characters that were tested and implemented herein. ACRV, among character rate variation; Asym, asymmetric state frequencies; BI, Bayesian inference; FBD, fossilized birth-death model; GA *or* ga, Gamma distribution; IGR (independent gamma rates uncorrelated clock model); LN *or* ln, lognormal distribution MML (SS), marginal model likelihoods (using the stepping-stone procedure); NoSA, no sampling of ancestors; PMF, probability mass function; SA, sampling of ancestors; SFBD, skyline fossilized birth-death model; Sym, symmetric state frequencies; TK02 (Thorne and Kishino continuous autocorrelated clock model). See the “Methods” section for additional details and explanation for different models

Our study provides a new morphological phylogenetic data matrix of sphenodontian reptiles, constructed under strict criteria to avoid potential sources of biases in morphological datasets, such as logical or biological dependencies among characters, inherent to problematic character constructions [44–46]. Using this new empirical dataset (see Additional files 1, 2, 3, 4) and taking into account recent advances in the implementation of morphological clocks (e.g., the benefits of accounting for fossil age uncertainty, sampling autapomorphies, and using mechanistic tree models [29, 32, 33]), we investigated the impact of several additional available prior model choices (Fig. 1) on morphological data to answer the following questions: (1) What is the impact of distinct taxon sampling prior strategies for the FBD model with morphological data only? (2) Under the correct taxon sampling assumption, what is the impact of different clock models on divergence times and evolutionary rates? (3) What is the impact of allowing FBD model parameters to vary across time? (4) What is the impact of partitioned morphological clocks on divergence times and evolutionary rates? and (5) To what extent do the limitations of morphological data (i.e., low number of characters) prevent complex model implementations? We discuss important biases stemming from various model choices that may lead to unreliable divergence times and rates of evolution using morphological clocks. We also compare our results to previous assessments of the behavior of those same model parameters in molecular or combined evidence datasets. Finally, we provide potential sources of correction to alleviate some of the biases introduced by inappropriate modeling of morphological datasets, along with a new hypothesis of sphenodontian relationships and macroevolutionary patterns.

## Methods

For a workflow of the analytical procedures, see Fig. 1.

### Morphological dataset construction

One of the major goals of this study is to provide a comprehensive reevaluation of morphological characters used and analytical approaches taken towards understanding phylogenetic relationships in sphenodontians. Therefore, several modifications to characters previously utilized in sphenodontian phylogenies (e.g., [47–54]) were implemented, as well as including several additional characters from various regions of the skeleton that have never been considered before. The essential criteria for character construction and selection, including characters utilized in other lepidosaurian datasets, have been thoroughly described and illustrated by some of us before [44, 55]. Those conceptual approaches and guidelines have been subsequently used in the construction of new morphological datasets for diapsid and lepidosaur reptiles [13], early tetrapods [56], chelonioid turtles [57], and pterosaurs (Rodrigues, T., pers. com.). In brief, we follow fundamental principles of character construction, such as avoiding logical and biological dependencies among morphological characters, utilizing the criterion of similarity to avoid character constructions which are extremely likely to have originated from unrelated historical processes (non-homologous characters) [58–60], and maintaining a clear and standard protocol towards the formulation and description of characters statements [45]—see Simões et al. [44] for further details.

Concerning the character codification process—creating character states from a particular transformation series [44]—we follow a contingent coding approach: the absence/presence of a particular anatomical structure is defined as a separate character from the different variation of that anatomical structure, such as shape or size. As previously demonstrated, this is the least spurious option available concerning the treatment of inapplicable and missing data scorings in the dataset [46, 61]. All characters are treated as unordered to avoid any constraints on the direction of evolutionary transformations (i.e., a “flat prior” on the direction of evolution).

We have sampled for the present dataset not only potential synapomorphic characters (with all states present in two or more taxa) but also direct autapomorphies (characters in which one state is observed in only one taxon, in the case of binary characters). Although autapomorphies are of no consequence towards establishing sister group relationships using maximum parsimony (apart from artificially inflating consistency indices [62]), they directly impact branch length estimates and evolutionary rates [24, 32]. For this reason, all kinds of variable morphological features (representing candidate synapomorphic and autapomorphic characters) should be sampled when methods that consider branch lengths are utilized (i.e., likelihood/statistical based methods).

Besides sampling direct autapomorphies, it is also important to consider that many characters have character states that are eventually recovered in the analysis as independently acquired by distantly related terminal taxa: homoplastic autapomorphies. In fact, the latter is the sole responsible for all character changes contributing to branch lengths at the tips (i.e., terminal taxa) in datasets that do not specifically try to sample for autapomorphies (and which are quite frequent in many morphological datasets). Therefore, although autapomorphies should be sampled as frequently as synapomorphies (as the number of tips is roughly equal to the number of internal nodes), it is also important to take homoplastic autapomorphies into account to avoid oversampling of character changes at the tips relative to internal branches. In the present dataset, a considerable number of similar character states seem to occur at low frequency and/or terminal taxa that are highly unlikely to be recovered as sister taxa. Those characters are likely to represent homoplastic autapomorphies, which was subsequently confirmed in our preliminary analyses and then in the final results. Therefore, we limited the number of direct autapomorphies sampled to be ~ 20%. In the final version of the dataset, we had 22 direct autapomorphies out of 131 characters (17% of the entire dataset). Out of those 22 characters, six characters were multistate characters, and only one of the character states was autapomorphic, therefore contributing both to estimates of tree topology and branch lengths at the tips. Methods for a more objective quantification of the recommended number of direct autapomorphies to be sampled still have to be developed for morphological phylogenetics.

Finally, one rarely discussed aspect of character sampling that impacts not only tree topology but also macroevolutionary estimates is the proportion of characters sampled across different regions of the phenotype. Characters sampled mostly from one body region to the neglect of others will create a tendency for evolutionary rates to reflect changes associated to that region only and taxa that can be diagnosed by them. For example, many morphological datasets have historically focused on characters sampled from the skull, with a very small proportion of characters sampled from the postcranium (frequently less than 20% of the entire dataset—e.g., [47, 63]]. To minimize such biases in the present dataset, we specifically aimed towards sampling characters with equal effort among all body regions for all sampled taxa (irrespective of their presumed phylogenetic placement), which inherently leads to a more homogeneous sampling of characters throughout the phenotype—see Additional file 2 (character list).

### Taxon sampling

For outgroup comparison, we selected a collection of early evolving lepidosaurs and squamates (*n* = 8) and chose the archosauromorph *Prolacerta broomi* as the designated outgroup for the parsimony analyses with TNT and relaxed clock analyses. This relatively large sample of outgroup taxa avoids long unsampled branches that would not capture important character transformations leading up to sphenodontians.

For ingroup taxonomic selection, the following criteria were considered: (i) personal observation of specimens, (ii) completeness of specimens, and (iii) representation of as many morphotypes as possible. Although there are no previous attempts to revise all sphenodontian species and provide a total number of valid species, our own estimates based on the published literature indicate ca. 60 currently valid species, but the taxonomic status of some are dubious and in need of revision (e.g., various species assigned to the genus *Homeosaurus*). A total of 30 ingroup taxa were included in our dataset, thus representing half of all known sphenodontian diversity, and representing most of the phylogenetically informative taxa (as several taxa are represented by fragmentary jaws and dentitions only). Total taxon sampling included 38 terminal taxa.

Several described species of sphenodontians have not been included in the current version of this dataset, owing to extremely fragmentary and poorly informative specimens, and which were also not personally studied by any of us, such as *Fraserosphenodon*, *Rebbanasaurus*, *Godavarisaurus*, and *Sphenocondor*. Importantly, a few of those taxa consist of isolated jaw elements and/or postcranial material, some of which have been assigned to a single species despite being recovered in complete dissociation, as well as occurring in localities with other reptile remains (occasionally including other lepidosaurs) mixed in with those elements—e.g., *Fraserosphenodon*, *Sigmala*, and *Pelecymala* [64, 65]. In certain instances, the erection of new taxa was considered “not desirable” based on isolated elements collected in faunal assemblages, but was performed in spite of that in the hopes of future identification of more complete specimens [65], which, however, has not occurred thus far. Other taxa not currently sampled include *Polysphenodon* and *Brachyrhinodon* [49, 66]. Those taxa were personally observed (T.R.S.) and consist of matrix impressions only that are extremely faint as currently preserved. Latex molds were available for some of specimens, but even in those only a small number of characters could be identified—see Additional file 3 for list of sampled taxa, along with their occurrence data, stratigraphic interval, age, anatomical bibliography, and personally observed specimen numbers.

### Rogue taxa identification and data filtering

The accumulation of taxa represented only by fragmentary remains increases the overall amount of missing information in the dataset, which reduces the ability of statistical methods to infer accurate phylogenies for morphological data, even at high levels of taxonomic sampling [27]. We thus focused on sampling species with more completely preserved specimens; our goal was to construct a robust general framework of sphenodontian relationships (into which more incomplete taxa may be added in the future by other researchers to address individual taxonomic problems). Additionally, even taxa represented by relatively complete specimens may still behave as rogue taxa, making it important to identify those rogue taxa (using several available procedures) and remove them from the dataset for subsequent (taxon reduced) analyses. Following this premise, the analyses conducted here followed a two-step procedure. The first step was to run an initial equal weights maximum parsimony and a non-clock Bayesian inference analysis. The various optimal trees obtained—most parsimonious trees from maximum parsimony and posterior trees after discarding the burn-in from Bayesian inference—were then subjected to rogue taxon identification using TNT’s [67, 68] pruning trees algorithm, as well as RogueNaRok [69]. Three ingroup taxa were detected as rogue taxa (see the “Results” section). In step two, rogue taxa were deleted from the dataset and a second set of analyses were conducted with a total of 35 terminal taxa, as described below, utilizing maximum parsimony (equal weights and implied weighting), non-clock Bayesian inference, and relaxed clock Bayesian inference.

### Equal weights maximum parsimony

Analyses were conducted in TNT 1.1 [67] with all characters unordered. All heuristic searches were done under equal weights and consisted of 100 rounds of random addition sequence (RAS) of taxa followed by Tree Bisection Reconnection (TBR) branch swapping, holding 100 trees per replication and collapsing branches of zero length after tree search. The resulting trees were used as starting trees for a final round of TBR branch swapping.

### Implied weighting maximum parsimony

We followed the same procedures as above for equal weights maximum parsimony but choosing the implied weighting option with a concavity index (*K*) = 12. The higher the concavity index the lower the penalty for homoplastic characters, but a precise assessment of *K* values for different dataset sizes is lacking. Values above the default value of 3 are necessary for the present dataset as it would result in some characters having a fit = 0 (effectively being removed from the analysis). *K* = 12 reduces the penalty for homoplastic characters and we consider it a more conservative approach, as previously used in other studies [13, 70].

### Bayesian inference

#### Models of morphological character evolution

Both non-clock and relaxed morphological clock analysis were conducted using Mr. Bayes v. 3.2.7a [71] using the CIPRES Science Gateway v.3.3 [72]. The morphological partition was analyzed using the Mkv model [24] with all characters unordered. Since morphological data sets include variable characters only, the Mkv model introduces an ascertainment correction bias that corrects for the absence of invariable characters for the model of character evolution. Further, we choose the Mkv model instead of the Mk-parsinf [32] of character evolution (the latter modeling data sets with parsimony informative characters only) given our explicit sampling for autapomorphies in the present dataset. We tested for the best fitting probability mass function (gamma or lognormal) to model among character rate variation (ACRV) in our dataset, using the stepping-stone sampling strategy to assess the marginal model likelihoods [73] and calculating Bayes factors (BF) [74]—50 steps for 100 million generations. We found that both functions provided nearly equal marginal model likelihoods with Bayes factor = 2*log*_*e*_(*B*_10_) = 2.04 (in favor of lognormal). Since there is no substantial difference, we chose a gamma distribution as it is more widely implemented, making our results easier to compare to previous studies.

Another factor to consider in models of character evolution for morphological data with a potential impact on estimates of evolutionary rates and divergence times is accounting for uneven (or asymmetric) distribution of state frequencies among characters. Currently, nearly all empirical Bayesian phylogenetic analyses of morphological data assume state frequencies as equal (symmetric) for all characters. In this approach, different states are always expected to have a similar frequency among characters (e.g., states “0” and “1” will always have a frequency of 0.5 each for binary characters, a three state character will have each state with a frequency of 0.33, and so on). This simplifying assumption of the distribution of state frequencies is a result of the difficulty of utilizing a single Q-matrix (a matrix of character state substitution that governs different models of character evolution) to model all morphological characters as usually done for molecular data. This limitation is imposed by the lack of comparability (homology) between states of distinct morphological characters (e.g., “absence” of the humerus is not the same as the “absence” state for the femur), a problem not faced by comparing the same nucleotides or amino acids across molecular sites [24]. A potential solution to this problem is to model character state frequencies varying across characters according to a particular distribution, as similarly performed when accounting for rate variation among character. This has been implemented in Mr. Bayes v. 3.2., which uses a symmetric Dirichlet distribution to model variation in state frequencies across characters [74] and has been previously explored for a collection of morphological datasets by Wright et al. [75]. The latter study found that most of the analyzed morphological datasets do not have strong levels of state frequency heterogeneity, therefore assuming a symmetric state frequency provides an adequate substitution model. However, ongoing research suggests that in datasets where there is heterogenic state frequencies in the characters, this model violation may result in strongly biased estimates of divergence times [76].

Here, we tested the fit of our data to both symmetric and asymmetric character state frequencies through Bayes factors comparisons. We tested a wide range of alpha values that govern the shape of the symmetric Dirichlet distribution to account for variable levels of asymmetry in character state frequencies. This was done by sampling alpha values from a uniform distribution between 0.05 (high asymmetry) and 20 (low asymmetry). We used Bayes factors to also compare the fit of a substitution model accounting for asymmetric state frequencies against the more simplistic and widespread symmetric state frequencies model on the final best performing analyses. The results indicate significantly higher marginal model likelihoods for the symmetric model against the asymmetric model for the present dataset under both non-clock (− 754.57 vs − 976.33) and morphological clock (− 1193.14 vs − 1225.60) analyses using the chosen gamma distribution for ACRV.

Convergence of independent runs was assessed using: average standard deviation of split frequencies (ASDSF ~ 0.01), potential scale reduction factors [PSRF ≈ 1 for all parameters], and effective sample size (ESS) for each parameter greater than 200.

#### Clock rates and models

The base of the clock rate was given an informative prior as per previous non-clock analysis—the median value for tree height in substitutions from posterior trees divided by the age of the tree based on the median of the distribution for the root prior (5.3456/267.1 = 0.02). Additionally, we tested for different probability mass functions for the prior on the base of the clock rate—a lognormal and a gamma distribution. For rates sampled from a lognormal distribution, the mean of the lognormal distribution was given the value based on the non-clock tree estimate (0.02) in natural log scale = − 3.9120. We chose to use the exponent of the mean to provide a broad standard deviation (*e*^0.02^ =1.0202). For rates sampled from a gamma distribution, the mean of the gamma distribution was also taken from the previous non-clock estimate (mean = 0.02) and provided a broad standard deviation = 0.5 (0.02, 0.5).

We tested between two different clock models with distinct assumptions of the mode of character evolution: one uncorrelated clock model [independent gamma rate (IGR) [77]], in which rates are free to change more dramatically among neighboring branches, thus reflecting a more punctuated mode of evolution [17]; one autocorrelated clock model [continuous autocorrelated clock of Thorne and Kishino [78](TK02)] [11], which include an autocorrelation parameter that limits how much rates may depart in relation to the rates on the parent branch, thus representing a more gradualistic mode of evolution [17].

As for rate variation among characters, we tested the fit of the clock models to the data: the IGR and TK02 clock models under both a gamma and lognormal distributions for the base of the clock rate, generating four different clock model combinations: IGR+ga; IGR+ln; TK02+ga; TK02+ln. The resulting marginal model likelihoods are respectively − 1211.33, − 1208.93, − 1209.39, and − 1195.67. Therefore, the TK02+ln model has a higher marginal likelihood than all other models, with a BF = 26.52 relative to the second best-ranking model (IGR+ga). This is a significant difference between models and indicates a strong preference for the TK02+ln model [79].

#### Tree models: variations on the FBD process

##### Taxon sampling strategies in the FBD process model

All relaxed morphological clock analyses implemented the fossilized birth-death (FBD) process for the parameterization of our tree model. In the FBD process, there are different strategies to model how both fossil and extant taxa are sampled in the construction of the tree prior using the fossilized birth-death (FBD) process in Mr. Bayes: “fossiltip” (in which fossils and extant taxa are assumed to be sampled randomly and fossils can only be tips), “random” (in which fossils and extant taxa are assumed to be sampled randomly and fossils can be either tips or ancestors), and “diversity” (in which fossils are assumed to be sampled randomly whereas extant taxa are assumed to be sampled in a way to maximize diversity, and fossils can be tips or ancestors) [80]. Those variations on the FBD model have drastic effects on estimates of branch lengths and divergence time estimates [81] and the incorporation of diversity sampling has been shown to improve divergence time estimates in total-evidence datasets [11, 12, 15].

The present dataset, despite being primarily comprised of fossil taxa, includes the single living representative among all sphenodontians (*Sphenodon punctatus*) and thus, for modeling purposes, it should fall within the assumptions of the diversity sampling strategy of the FBD process. In diversity sampling, it is assumed that there is one representative extant species being sampled per clade surviving after the cutoff time *x*_*cut*_ (time after which no more fossils are sampled) [15]. In the context of our dataset, *x*_*cut*_ is approximately at the Cretaceous-Palaeogene boundary at 66 Mya, with *Sphenodon* representing the only lineage we currently know of that survived the K-Pg extinction. Therefore, we implemented the diversity sampling strategy, but also implemented all other sampling strategies to test for the impact of sampling model mismatch on tree topology, divergence times and evolutionary rates.

It is important to note that the three available strategies in Mr. Bayes allow different modeling strategies for how the extant taxa are sampled (randomly vs maximizing diversity) and the sampling of ancestors. A major software alternative BEAST2 [82], currently lacks these modeling parameters and necessarily assumes a random sampling strategy for extant taxa, and therefore, we did not utilize BEAST 2. Further, Mr. Bayes implements only three combinations out of four as explicitly named sampling strategies, since it does not directly allow the sampling of extant taxa to maximize diversity while also prohibiting fossils to be ancestors (fossils as tips only). Therefore, to test the impact of the latter we utilized the diversity sampling strategy but reduced the probability of fossils being ancestors to zero by reducing to zero the probability of branch moves that would place fossils as ancestors—changing tuning parameters for move proposals to "propset AddBranch$prob = 0; propset DelBranch$prob = 0;". This effectively implements in Mr. Bayes a diversity sampling strategy with fossils as tips only (not sampling for ancestors), and it is referred here as the “NoSA diversity” sampling strategy. We also note that a similar combination of model parameters can be performed in the sister program to Mr. Bayes, RevBayes [83], which has an overall similar performance for tree inference to Mr. Bayes using non-clock Bayesian inference for morphological datasets [27]. Our tests were performed in Mr. Bayes given its greater stability when analyzed in high-performance computer clusters (RevBayes runs were often terminated prematurely in some tests). However, the extremely similar behavior of both software to analyze datasets varying in taxon number, amounts of missing data, and levels of character homoplasy [27] suggest that our conclusions here may extend to analyses with RevBayes as well.

##### The skyline FBD (SFBD)

In the skyline variation of the FBD process (SFBD), net diversification, relative extinction (or turnover), and fossil sampling probability parameters are allowed to change in a piecewise constant manner along the tree, instead of assuming a constant rate for those parameters across the entire tree [16]. The SFBD provides a stronger parameterization of the fossilized birth-death model, thus permitting the modeling of the very distinct expectations on the probability of fossilization, speciation, or extinction across time. This can be achieved by indicating the number of rate shifting times (*l*) and the time (*t*_*i*_) of those respective rate shifts [*t*_*i*_ (*i* = 1, …, *l*)] [12, 15]. Despite its potential to provide a more accurate modeling of the diversification process across different geological timescales (a highly relevant aspect of higher-level phylogenies with taxonomic sampling extending into deep time), to our knowledge the SFBD has never been tested in morphology-only empirical datasets.

To test the impact of the SFBD model, we chose the best performing sampling strategy analyses in our results (the two in which ancestors were not sampled: fossiltip and NoSA diversity—see the “Results” section) and implemented variations of the SFBD as focal analyses. In analyses using the fossiltip sampling strategy, we implemented two rate shift times to separate the birth-death tree into three time slices (strategy SFBD3l): one at the Jurassic-Cretaceous boundary at 145 Mya (after which the availability of sphenodontian fossils decreases considerably [52, 84]), and another at the Cretaceous-Palaeogene (KPg) at 66 Mya, which marks the end Cretaceous mass extinction. After 66 Mya, there are no new genera of sphenodontians currently known and the fossil record is extremely scarce for the entire lineage during the Cenozoic. Only one genus that originated in the late Cretaceous (*Kawasphenodon*) survives the K-Pg boundary into the Paleocene in Argentina [85]. There are fragmentary sphenodontine remains from the Miocene of New Zealand [86] and subfossils of the modern *Sphenodon punctatus* from the Pleistocene-Holocene of New Zealand [85, 86]. Thus, the KPg also represents the time after which no more fossils are sampled for the current tree (*x*_*cut*_). This prior knowledge makes it reasonable to expect that fossil sampling rates should be estimated separately and have distinct values before and after the KPg, as well as before and after the Jurassic-Cretaceous border.

The diversity sampling strategy considers that the fossil sampling rate after cutoff time *x*_*cut*_ is zero, and so, it is argued that estimating a separate fossilization rate parameter after *x*_*cut*_ should provide “uncertain estimates” [15]. However, to our knowledge, this was never in fact tested empirically in order to learn what kind of bias may result from estimating separate FBD parameters after *x*_*cut*_. Therefore, for NoSA diversity sampling strategy, we implemented two different rate shift time strategies: SFBD3l (as described above), and SFBD2l, in which we fixed one rate shift time only at 145 Mya. As expected, strategy SFBD3l revealed unrealistically biased parameter estimates for NoSA diversity analyses (see the “Results” section), and so strategy SFBD2l was the preferred one for subsequent analyses and considerations.

When choosing the fossiltip sampling strategy, Mr. Bayes does not allow setting up the SFBD process with its standard coding (prset samplestrat = fossiltip), followed by the number of rate shift times, and the age of those rate shift times. We circumvented this by specifying the random sampling strategy (prset samplestrat = random) with two rate shifts (SFBD3l), but reducing the probability of fossils being ancestors to zero by reducing to zero the probability of branch moves that would place fossils as ancestors [with the command "propset AddBranch$prob = 0; propset DelBranch$prob = 0;"]. In practice, this has the desired effect of keeping fossils as tips only (fossiltip sampling strategy), while allowing for the SFBD tree model.

All of the steps above for both sampling strategies (and for SFBD2l and SFBD3l subdivisions) would result in a considerable increase in the number of parameters for the analysis if all three FBD free parameters—net diversification (*d*), turnover (*r*), and proportion of fossil sampling (*s*)—were to be estimated separately for each time slice. Specifically, the total number of free parameters would be 3 *l* (*l* = rate shift times). We further tested the impact of increasing the number of free parameters by applying the SFBD tree model with all three parameters free to change across time slices [SFBD (sdr) model] and a separate analysis in which only one of those parameters, the proportion of fossil sampling, was allowed to shift across time slices [SFBD(s) model]. We chose the proportion of fossil sampling for the latter as this parameter is the most straightforward of the FBD process to be modeled based on prior knowledge of the fossil record, as it is relatively simple to identify periods of geological time when we expect to find shifts in the likelihood of sampling fossils for particular clades. Such rate shift time periods include mass extinctions, the first or last occurrence of a clade in the fossil record, among other criteria. Estimates of shifting net diversification or relative extinction (turnover) in the fossil record may coincide with some of those rate shift times for the probability of sampling fossils (e.g., mass extinctions). However, identifying good candidate rate shift times specifically for diversification and extinction in lineages through time, represents a considerably more difficult challenge and for which various distinct methods with contrasting results are available [87–89]. Therefore, we consider the fossil sampling probability (*s*) the best candidate parameter in the SFBD model to be estimated independently across time slices, also providing the SFBD model with the least number of free parameters: only one per time slice, with a total of 1 *l* free SFBD parameters. Finally, recognizing the major changes in fossil sampling on the vertebrae fossil record may be relatively straightforward and able to be assessed qualitatively (as herein). However, for groups with much denser taxon sampling in the fossil record in which those patterns are not easily recognizable (e.g., hard-bodied invertebrates), we recommend a quantitative assessment of changes in the completeness of the fossil record.

#### Morphological data partitioning

Partitioning of datasets into subsets that share similar models of evolution and branch lengths is already widely implemented for molecular data sets and is known to improve overall performance on phylogenetic inference [19, 90, 91]. Regarding morphological datasets, previous studies have shown the potential of data partitioning in morphological data sets [12, 92], but they have rarely ever been applied to empirical data sets or used for macroevolutionary inferences—but see [14].

Character partitioning is automatically performed by Mr. Bayes (and it is also possible in BEAST2 and RevBayes) to subdivide characters according to the number of states and assigning them different character evolution Q-matrices. However, given the small number of available variations on the Mk model of morphological evolution (see Fig. 1), most of the benefits of character partitioning are likely to be obtained by assigning a separate morphological clock to each partition and inferring separate estimates of character evolution from each of them. Even small morphological data sets tend to comprise data from multiple anatomical regions that may be subject to different selection pressures or distinct evolutionary rates, thus being prone to constitute separate evolutionary modules. Treating potentially distinct morphological partitions as a single partition may thus have severe consequences to phylogenetic inference, as they represent an inappropriate modeling of morphological evolution. Further, model underparameterization (such as “lumping” partitions together) may lead to greater phylogenetic error than an equivalent degree of overparameterization (e.g., “splitting” partitions), as already detected for molecular datasets [91, 93, 94]. Underpartitioning also results in less precise divergence time estimates by highly increasing the range of the 95% highest posterior density interval for divergence times [19]. On the other hand, overpartitioning, or not having enough data per partition, may lead to convergency problems between MCMC runs, using multiple relaxed clocks [14]. The later may be an especially relevant, yet poorly understood, limitation of morphological data sets in phylogenetics, as those typically comprise relatively few numbers of characters that may prevent reasonable sample sizes for parameter estimation across all partitions.

To understand the potential impact of data partitioning to estimates of topology, divergence time, and evolutionary rates on a standard sized morphological dataset, we employed a series of additional analyses under a functional-based partitioning of morphological characters. In principle, we should aim for each partition to contain enough character state changes to match at least the total number of tree branches: each partition should contain at least 2n-1 characters (*n* = number of taxa in the dataset), assuming that each character has at least one state change. The latter assumption applies to nearly all morphological datasets, even when including autapomorphies, and highly homoplastic characters may bring down the minimal number of characters per partition even further. For the present dataset, 2n-1 = 69, which suggests a maximum of two or three morphological partitions. Therefore, we limited the number of partitions for the present dataset to only three morphological partitions (skull [47 characters], mandible+ dentition [44 characters], and postcranial [38 characters]).

#### Root and tips age calibration

The prior on the age of the root was assessed by taking available ages for fossils and previous divergence time estimates. The minimum age is based on the oldest known lepidosauromorphs: *Palaeagama vielhaueri* (Middle Lopingian, Late Permian-middle Olenekian, Early Triassic [95]) and *Sophineta cracoviensis* (Early late Olenekian, Early Triassic [96]) = minimum of 247.2 Mya. For the mean age, we used the previous median estimate for the Lepidosauromorpha node [13] = 287 Mya. Uncertainty on the age of the root may result in very unprecise divergence time estimates, leading many molecular clock studies to use an exact age or upper bound constraint on the root age. However, to account for uncertainty on the root age, we initially utilized a soft upper bound by applying an offset exponential distribution on the root age, which attributes a higher probability for the age of the root to be closer to the minimum age, but also providing a small (but nonzero) probability for the root age to be older than expected based on the input values. For a final set of analyses, we also utilized a truncated normal distribution for the root age ("NoR" tree prior), thus allowing variation on the age of the root, but with a hard upper bound. The latter prevents the age of the root to be older than the earliest Permian and potential overestimates on the root age.

All our calibrations (apart from the root node) were based on tip-dates, which account for the uncertainty in the placement of fossil taxa and avoids the issue of bound estimates for node-based age calibrations [11]. The fossil ages used for tip-dating correspond to a uniform prior distributions on the age range of the stratigraphic occurrence of the fossils (Supplementary File 2), which avoids biases in divergence time estimates introduced by single point age calibrations [29].

#### Result outputs

Our reported summary trees were calculated with standard output tree procedures available in Mr.Bayes. These include a 50% majority rule consensus tree (MRC) and the tree that is compatible with all input trees and that contains the largest possible number of taxa [97] termed a maximum compatibility tree (MCT) [97]. The mean posterior estimate for the base of the clock (evolutionary) rate value for all morphological characters (a generalized absolute background rate) was obtained from the log files and analyzed with Tracer v. 1.7.1 [98], representing numbers of substitutions per character per million years. A second parameter estimated in Bayesian relaxed clocks is the variance on the base of the clock rate, which represents the deviations from the clock rate parameter at every branch of the phylogeny, thus representing a relative clock (evolutionary) rate estimate. Therefore, relative rate values at each branch of the evolutionary tree were determined, with values > 1 representing accelerating rates, and values < 1 representing decelerating rate values [80].

All input and output files from the analysis performed, full tables with macroevolutionary parameters and R scripts for statistical analyses (Additional files 5, 6, 7) are available online [99].

## Results

### Sphenodontian phylogeny and systematics

#### Tree topologies

Initial analyses (first step procedures—see the “Methods” section) containing all 38 taxa (Additional file 4) indicated *Clevosaurus convallis* and *Ankylosphenodon pacchyostosus* act as rogue taxa, considerably reducing resolution and generating a large number of polytomies. Subsequent analyses following the removal of those two rogue taxa (with 36 taxa) contained a much better resolved and more robust phylogenetic hypothesis under both maximum parsimony and Bayesian inference, but still contained one taxon (*Opisthias*) creating an unresolved relationship between sphenodontines and eilenodontines. *Opisthias* is currently known from very limited jaw elements, some of which there is dubious association with the type material and were not included herein (see Suppl. Info.). The removal of *Opisthias* considerably improved the stability of the inferred relationships among later branching sphenodontians, especially on the dichotomy between sphenodontines and eilenodontines, while keeping all other major sister group relationships unchanged. Therefore, while we provide the results with *Opisthias* included and provide scorings for *C*. *convallis*, *A*. *pacchyostosus*, and *O*. *rarus* in our dataset for subsequent studies (Additional file 4), most of our reported results focused on the more stable phylogenetic framework obtained by analyzing 35 terminal taxa and excluding the three aforementioned species.

**Fig. 2:**
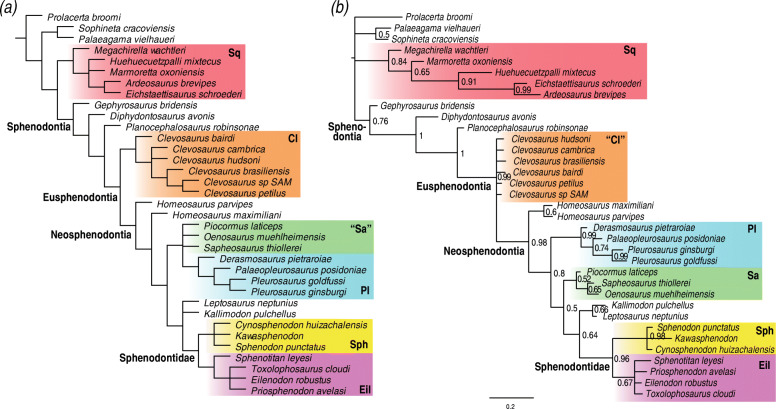
Maximum parsimony and non-clock Bayesian phylogenetic analyses. **a** Strict consensus of 12 trees (274 steps each) under equal weights maximum parsimony (same result as implied weighting maximum parsimony—see Suppl. Data). **b** MRC tree from non-clock Bayesian inference analysis. Node numbers indicate posterior probabilities. Cl, Clevosauridae; Eil, Eilenodontinae; Pl, Pleurosauridae; Sa, Saphaeosauridae; Sph, Sphenodontinae; Sq, Squamata

Final analyses subsequent to rogue taxon removal (second step procedures) under both equal weights and implied weighting maximum parsimony have well-resolved topological relationships and strong agreement between them concerning sphenodontian relationships (Fig. 2a). They all indicate *Gephyrosaurus* is the sister taxon to Sphenodontia, thus forming a monophyletic Rhynchocephalia, and with *Diphydontosaurus* and *Planocephalosaurus* as the earliest diverging sphenodontians. We recover a monophyletic Eusphenodontia, with an early dichotomy between Clevosauridae and Neosphenodontia. We have a partially resolved relationship within clevosaurids, with *Clevosaurus bairdi* as the earliest branching species of *Clevosaurus*, followed by *C*. *cambrica* + *C*. *hudsoni*, *C*. *brasiliensis*, and having *C*. *petilus* and *Clevosaurus* SAM as the latest diverging members of the clade. Neosphenodontia is characterized by an early divergence of a paraphyletic *Homeosaurus*, followed by a dichotomy between the clade comprising “saphaeosaurids” + pleurosaurids and the clade comprising *Kallimodon*, *Leptosaurus*, Sphenodontinae + Eilenodontinae (= Sphenodontidae, see the “Clade definitions” section). In the analysis in which *Opisthias* was included, it formed a clade with eilenodontines, thus forming a monophyletic Opisthodontia.

The non-clock Bayesian inference analysis has reduced resolution compared to the strict consensus tree obtained from both equal and implied weighting maximum parsimony, as is typical of morphological datasets (Fig. 2b). Nonetheless, the non-clock analysis recovers all major sphenodontian clades with well-resolved relationship, including between and within clades. The major exception is the poorly resolved relationship of the different species of *Clevosaurus*, creating a large polytomy between all *Clevosaurus* taxa and Neosphenodontia. Overall, the structure of the tree is very similar to the maximum parsimony analyses, including the placement of *Kallimodon* + *Leptosaurus* as the sister clade to Sphenodontidae. One notable difference is the placement of saphaeosaurids as the sister clade to of *Kallimodon* + *Leptosaurus* + Sphenodontidae, instead of being a sister clade to Pleurosauridae. Another difference is the monophyly of the genus *Homeosaurus*.

**Fig. 3:**
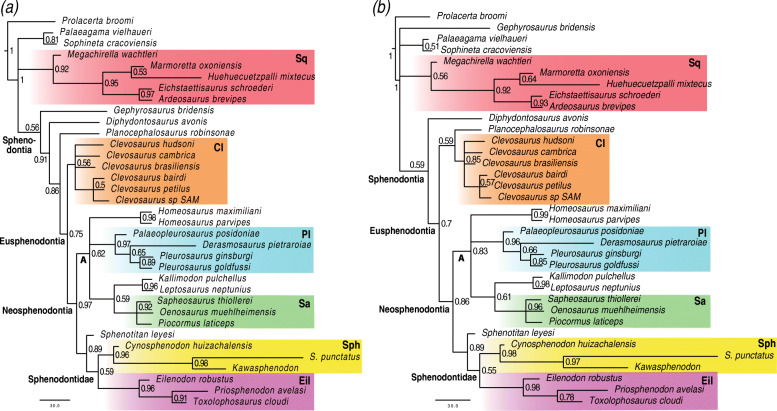
Relaxed morphological clock Bayesian inference with a single morphological clock partition. **a** MRC tree using the uncorrelated clock model. **b** MRC tree using the best fit continuous autocorrelated clock model. Node numbers indicate posterior probabilities. A, unnamed clade A; Cl, Clevosauridae; Eil, Eilenodontinae; Pl, Pleurosauridae; Sa, Saphaeosauridae; Sph, Sphenodontinae; Sq, Squamata

The relaxed morphological clock Bayesian inference analyses implementing the uncorrelated clock model recover tree topologies similar to that of maximum parsimony (Fig. 3a). In contrast, all analyses implementing the best fit clock model (autocorrelated) indicate an unstable phylogenetic position of *Gephyrosaurus*. *Gephyrosaurus* falls either in a basal polytomy between Sphenodontia, Squamata, *Palaeagama*, and *Sophineta*, or in a polytomy between Squamata, *Palaeagama*, and *Sophineta* only (Fig. 3b and Suppl. Figs. 3–8). In those same analyses, *Planocephalosaurus* falls as a sister taxon to Clevosauridae, instead of being the sister taxon to Eusphenodontia.

In contrast to the maximum parsimony and non-clock Bayesian inference analyses, in all clock trees (regardless of the clock model), there is a greater agreement in the relationships between later evolving lineages of sphenodontians. *Homeosaurus*, saphaeosaurids, pleurosaurids, and *Kallimodon* + *Leptosaurus* always form a large clade (unnamed clade A), although with poorly resolved internal relationships, and in which saphaeosaurids are almost always the sister clade to *Kallimodon* + *Leptosaurus*. Additionally, clade A is always the sister clade to Sphenodontidae, therefore refuting the hypothesis of the sister group relationship between *Kallimodon* + *Leptosaurus* and Sphenodontidae, as suggested by the maximum parsimony and non-clock Bayesian inference results. Finally, the internal relationships within sphenodontines and eilenodontines have stronger support and resolution using relaxed morphological clocks than all other analyses.

#### Clade definitions

Since the first phylogenetic analysis of sphenodontian relationships [49], new clades and clade names have been proposed but not always followed by a strict phylogenetic definition of those names. Below, we provide node or stem-based definition of clade names for all well-supported clades found in the analyses performed herein, along with revisions of previous apomorphy-based definitions, and definition for clades without prior phylogenetic-based definition.

Sphenodontia Williston, 1925. Definition: the most inclusive clade including *Sphenodon* and *Diphydontosaurus*, but not *Iguana iguana* and *Gekko gecko*.

Eusphenodontia Herrera-Flores et al., 2018. Definition: “the least inclusive clade containing *Polysphenodon muelleri* Jaekel, 1911, *Clevosaurus hudsoni*, and *Sphenodon punctatus*.” [100].

Clevosauridae Bonaparte and Sues, 2006. Definition: “all taxa more closely related to *Clevosaurus* than to *Sphenodon*.” [101].

Neosphenodontia Herrera-Flores et al., 2018. Definition: “the most inclusive clade containing *S*. *punctatus* but not *C*. *hudsoni*” [100].

Pleurosauridae Lydekker, 1880: Previously diagnosed based on the following apomorphy-based definition: “Sphenodontia showing a backward shift of both orbits and external nares, an elongation and triangularization of the skull, and an elongation of the pre- and postsacral skeleton by addition of vertebrae. Caudal autotomy is lost.” Redefined as: The least inclusive clade containing *Palaeopleurosaurus posidoniae*, *Pleurosaurus goldfussi*, and *Derasmosaurus pietraroiae*.

Saphaeosauridae Bau, 1825. Definition: The least inclusive clade containing *Sapheosaurus thiollerei*, *Piocormus laticeps*, and *Oenosaurus muehlheimensis*.

Eilenodontinae Rasmussen and Callison, 1981: The most inclusive clade containing *Eilenodon robustus*, but not *Sphenodon punctatus*.

Sphenodontinae Nopcsa, 1928: Previously diagnosed based on the following apomorphy-based definition: “caniniform tooth posterior to an edentulous margin” [47]. Redefined as: The most inclusive clade containing *Sphenodon punctatus*, but not *Eilenodon robustus*.

Sphenodontidae Cope, 1869. The clade Sphenodontidae was previously illustrated in a phylogeny by Reynoso [47] equivalent to the more recently defined Eusphenodontia [77]. We concur with the latter taxonomy as Eusphenodontia includes traditionally recognized families (e.g., Pleurosauridae, Saphaeosauridae), and so it would be unreasonable for this clade to have a family suffix following the International Code of Zoological Nomenclature (ICZN) rules. Sphenodontidae has subsequently been utilized to define other clades, including the clade formed by *Sphenodon* and *Cynosphenodon* (= Sphenodontinae as used herein and by Reynoso [48]) and Dupret [102]. A third application of the name Sphenodontidae was made by Herrera-Flores et al. [100] for a clade formed by Sphenodontinae plus some additional species, such as *Derasmosaurus*, *Oenosaurus*, *Zapatodon*, and *Ankylosphenodon*. These latter species were found to fall elsewhere (*Derasmosaurus*, *Oenosaurus*), were not included (*Zapatodon*), or were excluded as they behaved as rogue taxa (*Ankylosphenodon*). Besides the conflicts in previous usages of the term Sphenodontidae, to our knowledge, it was never formally defined. To solve these issues, here we provide a formal definition of Sphenodontidae, and in a manner that at least two traditionally recognized subfamilies are included within it (Eilenodontinae and Sphenodontinae). We choose this definition because, following the ICZN, Sphenodontinae must be necessarily included in Sphenodontidae (as it carries the type genus of both family and subfamily, *Sphenodon*), and also because of the stable sister group relationship between sphenodontines and eilenodontines in our analyses and most previous phylogenies. New definition: The most inclusive clade containing *Eilenodon robustus* and *Sphenodon punctatus*, but not *Kallimodon pulchellus* or *Saphaeosaurus thiollerei*.

### The impact of clock models, clock partitions, and changes to the FBD model on phylogeny and macroevolutionary parameters

#### The impact of node depth on node age uncertainty

**Fig. 4:**
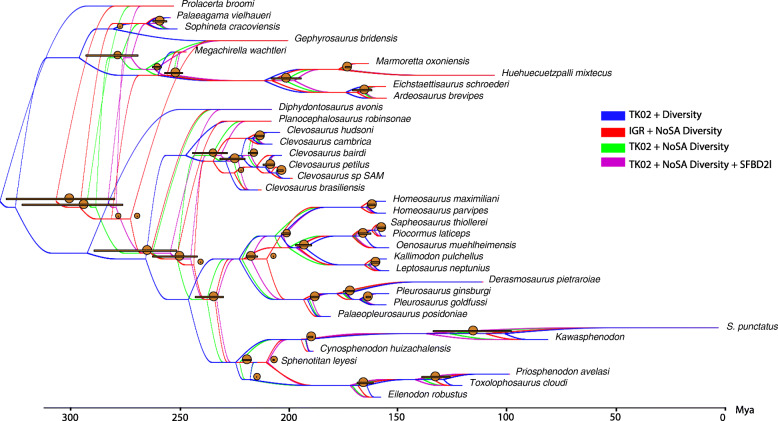
Density tree contrasting divergence times under distinct clock and tree models (single morphological clock). Each tree represents the maximum compatibility tree and median divergence times obtained from each model combination. The greatest disparity of divergence time variation given model choice is observed on nodes closer to the root. Sampling for ancestors (blue) yields much older age estimates for the root and most other nodes compared to not sampling for ancestors, regardless of the clock model. When not sampling for ancestors, the uncorrelated clock model (red) results in relatively older divergence times compared to the best fit autocorrelated clock model (green) in nodes closer to the root, but slightly younger on nodes closer to the tips. The SFBD tree model (purple) further reduces divergence times compared to the simpler FBD tree model when using the best fit autocorrelated clock model. Orange bars indicate range between maximum and minimum divergence times for each model combination and orange circles represent the midpoint between these respective maximum and minimum divergence times. Div, diversity sampling with fossils as tips or ancestors; FBD, fossilized birth-death tree model with constant perimeter rates across time; IGR, independent gamma rates uncorrelated clock model; NoSA diversity, diversity sampling with fossils as tips only; SFBD(s)2 l, skyline FBD tree model with one rate shift point for the relative fossilization parameter; TK02, continuous autocorrelated clock model

**Fig. 5:**
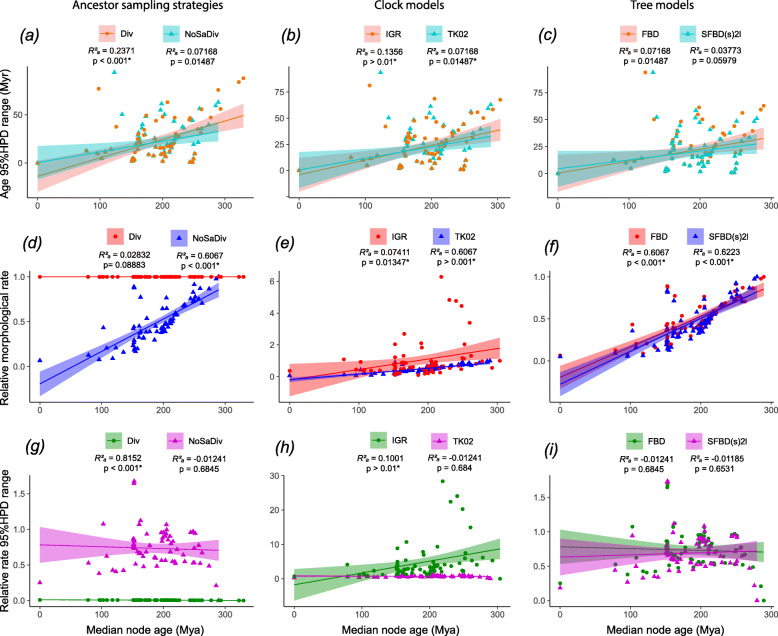
Linear regression between divergence time and evolutionary rate parameters. Data obtained from MCT trees under different tree model, clock model, and sampling strategies with a single morphological clock partition. **a**–**c** Precision around individual node divergence time estimates, based on 95% highest posterior density (HPD) ranges against median divergence times. **d**–**f** Median relative evolutionary rates against median divergence times. **g**–**i** Precision around individual node relative evolutionary rate estimates, based on 95% HPD ranges against median divergence times. Abbreviations: See Fig. 4 and “Methods” for model abbreviations

Observed variations in divergence times are highly correlated with node depth: nodes that are shallower on the tree (closest to the extant tips) have lower variation on estimated median divergence times, regardless of the strategy for taxon sampling, clock model, or partitioning (Fig. 4, Tables 1, 2, and 3). However, the median age estimates for deeper nodes (closest to the root) are highly variable across different sampling strategies and data partitioning schemes. Further, uncertainty on divergence times (measured by 95% HPD ranges) for each individual analysis has a general tendency to reduce on younger nodes (Fig. 5a–c).

**Table 1:** Median divergence time estimates obtained with various models and a single morphological clock

FBD model	Sampling strategy	Full model	Root		Sphenodontia		Sphenodontinae		Eilenodontinae	
			M	Rng HPD	M	Rng HPD	M	Rng HPD	M	Rng HPD
FBD	All sampling priors	FossilTip+IGR	307.3	71.1	**262.8**	**57.5**	191.3	19.8	165.5	36.5
		FossilTip+TK02	**293.6**	69.8	**262.7**	60.9	189.6	17.1	168.3	40.9
		Random+IGR	330.8	94.3	290.4	80.8	191.2	21.9	165.5	38.1
		Random+TK02	335.2	91.2	293.1	78.3	188.9	16.4	161.1	30.4
		Diversity+IGR	326.4	89.7	286.8	78.1	191	21.2	165.2	37.4
		Diversity+TK02	329.7	87.8	289.5	75.6	189	16.7	161.3	30.2
		NoSA_Diversity+IGR	303.8	**67.6**	**260.2**	**55**	191.1	18.9	165.1	35.5
		NoSA_Diversity+TK02	**289.4**	**62.1**	**259.5**	**56.3**	189.4	16.4	167.9	40
SFBD	NoSA diversity	SFBD(s)2l+IGR	**297.4**	60.8	**255.3**	50.0	190.8	18.0	165.0	34.4
		SFBD(s)3l+IGR	**298.7**	63.3	**256.3**	51.2	190.8	18.3	165.0	34.5
		SFBD (sdr)2l+IGR	305.3	77.9	**261.7**	63.2	191.5	20.6	167.4	40.6
		SFBD(s)2l+TK02*	**279.7**	**51.1**	**251.3**	48.6	189.0	15.1	168.7	39.4
		SFBD(s)3l+TK02	**281.5**	53.3	**252.8**	50.0	189.0	15.3	168.6	39.3
		SFBD (sdr)2l+TK02	**282.1**	60.9	**253.5**	56.3	189.2	16.4	170.6	41.9

Results obtained under the FBD (top) and the skyline FBD (SFBD) tree model (bottom) with distinct taxon sampling strategies and relaxed clock models. The SFBD analyses are focused on the optimal sampling strategy (NoSA diversity). Median values are reported given the very broad and skewed 95% HPD distributions. Values in bold highlight divergence times in stronger agreement with the fossil record (e.g., within the Permian for the root age), which also have narrower 95% HPD ranges. *Abbreviations*: *M* median value for node age, *Rng HPD* range between lower and upper bound values for the 95% HPD. *Model combination with the most precise (smallest 95% HPD range) and conservative (greatest agreement with the fossil record) root age estimate under a single morphological partition

**Table 2:** Median divergence time estimates obtained with various models and partitioned morphological clocks

Full model	Root		Sphenodontia		Sphenodontinae		Eilenodontinae	
	M	Rng HPD	M	Rng HPD	M	Rng HPD	M	Rng HPD
FBD+IGR	321.5	74.3	275.5	65.9	192.1	20.0	167.1	37.8
FBD+IGR+Start	321.2	74.3	275.5	66.4	192.1	20.0	167.1	37.6
SFBD(s)2l+IGR+Start	316.3	69.4	271.0	62.9	191.9	19.2	166.9	36.8
FBD+TK02	320.6	83.2	282.7	72.4	189.6	17.6	164.0	35.6
FBD+TK02+Start	320.9	86.7	282.3	75.1	189.5	17.4	164.2	35.5
SFBD(s)2l+TK02+Start	310.5	79.1	274.6	69.6	189.3	16.6	163.8	34.4

Results obtained under the FBD and SFBD tree models with no sampling of ancestors (NoSA_Diversity) taxon sampling strategy and distinct relaxed clock models. Median values are reported given the very broad and skewed 95% HPD distributions. *Abbreviations*: *M* median value for node age, *Rng HPD* range between lower and upper bound values for the 95% HPD, *S* sampling strategy

**Table 3:** Median divergence time estimates obtained with informative priors and partitioned morphological clocks

Constraints	Full model	Root		Sphenodontia		Sphenodontinae		Eilenodontinae	
No constraints		**M**	**Rng HPD**	**M**	**Rng HPD**	**M**	**Rng HPD**	**M**	**Rng HPD**
	FBD+Start+LExct	325.0	87.8	285.5	76.5	189.7	17.9	164.2	18.5
	FBD+Start+NoR	**290.4**	**18**	**262.4**	**37.9**	188.7	14.7	163.3	32.8
	SFBD(s)2l+Start+NoR*	**289.5**	**18.4**	**260.2**	**38.1**	188.6	14.2	163.7	33.1
	FBD+Start+LExct+NoR	**290.7**	**18.8**	**262.5**	**38.3**	188.7	14.8	163.2	32.8
	SFBD(s)2l+Start+LExct+NoR	**290.4**	**18.3**	**261.9**	**38.3**	188.7	14.6	163.7	33.4
Clock rate + topology	FBD+NoR	**287.8**	**17.4**	**261.8**	**32.9**	188.1	12.5	164.6	33.7
	FBD+LExct+NoR	**287.9**	**17.4**	**262.0**	**32.7**	188.2	12.6	164.7	34.1

Results obtained under the FBD and SFBD tree models with a NoSA diversity taxon sampling strategy and the (best fit) autocorrelated relaxed clock model. More informative priors are implemented to reduce deep root attraction. Median values are reported given the very broad and skewed 95% HPD distributions. Values in bold highlight divergence times in stronger agreement with the fossil record (e.g., within the Permian for the root age), which also have narrower 95% HPD ranges. *Abbreviations*: *M* median value for node age, *Rng HPD* range between lower and upper bound values for the 95% HPD, *S* sampling strategy. *Model combination with the most conservative root age estimate (greatest agreement with the fossil record) without topology or rate constraints

#### The impact of ancestors and taxon sampling strategies

Within each partitioning scheme, the biggest impact on divergence time estimates for the oldest nodes is whether or not ancestors are sampled (*R* = 0 vs *R* = 1, respectively), instead of the strategy upon which extant taxa are sampled: randomly (fossil tips/random) vs to maximize diversity (diversity/NoSA diversity) (Table 1, Figs. 4 and 5a). The only noticeable impact of the taxon sampling strategy is on the higher rates of relative fossilization under fossiltip compared to NoSA diversity sampling (for both single and multiple clock partitioning schemes—Tables 4, 5, and 6). Despite its impact on fossilization rates (higher fossilization rates would tend to reduce the length of ghost lineages), divergence times under a fossiltip sampling strategy were only slightly older than NoSA diversity sampling. We note that the reduced impact on the extant taxon sampling strategy in the present dataset when compared to previous assessments of this prior [12, 15] is quite likely to be a function of the extremely low diversity of extant sphenodontians. Therefore, our results and discussions focus on the observed impact of sampling for ancestors.

**Table 4:**
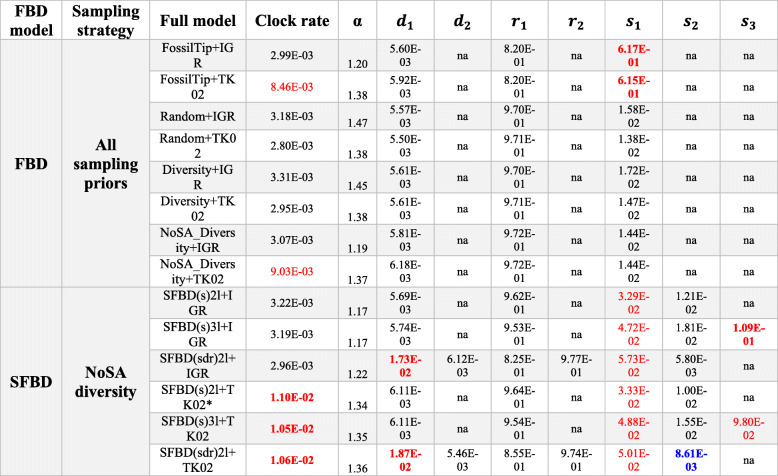
Mean posterior parameters obtained with various models and a single morphological clock

Results obtained under the FBD (top) and the skyline FBD (SFBD) tree model (bottom) with distinct taxon sampling strategies and relaxed clock models. The SFBD analyses are focused on the optimal sampling strategy (NoSA diversity). Mean values are reported given the very small variance for those parameter estimates (see full descriptive statistics in online Supplementary Information). Estimated means that are higher than modal mean values observed across all strategies with a single partition are highlighted in red, whereas means smaller than modal values are highlighted in blue. Mean values in bold indicate deviations that are an order of magnitude higher or lower than modal values. *Abbreviations*: *α* prior on the shape of the gamma parameter for rate variation among characters, *d* net diversification, *r*, turnover, *s*, proportion of fossil sampling, *S* sampling strategy. For full model explanation, see the “Methods” section. * Preferred model combination for single partition morphological data (see Table 1)

**Table 5:**
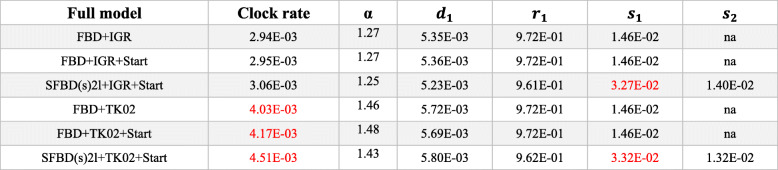
Mean posterior parameters obtained with various models and partitioned morphological clocks

Results obtained under the FBD and SFBD tree models with a NoSA diversity taxon sampling strategy and distinct relaxed clock models. Mean values are reported given the very small variance for those parameter estimates (see full descriptive statistics in online Supplementary Information). Color schemes are the same as in “Table 4.” *Abbreviations*: *α* prior on the shape of the gamma parameter for rate variation among characters, *d* net diversification, *r* turnover, *s* proportion of fossil sampling, *S* sampling strategy. For full model explanation, see the “Methods” section

**Table 6:**
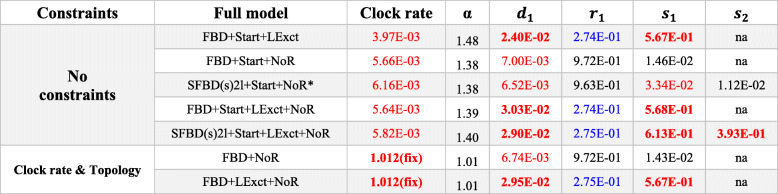
Median divergence time estimates obtained with informative priors and partitioned morphological clocks

Results obtained under the FBD and SFBD tree models with a NoSA diversity taxon sampling strategy and the (best fit) autocorrelated relaxed clock model. More informative priors are implemented to reduce deep root attraction. Mean values are reported given the very small variance for those parameter estimates (see full descriptive statistics in online Supplementary Information). Color schemes are the same as in “Table 4.” *Abbreviations*: *α* prior on the shape of the gamma parameter for rate variation among characters, *d* net diversification, *r* turnover, *s* proportion of fossil sampling, *S* sampling strategy. For full model explanation, see the “Methods” section. *Preferred model combination for multiple partitioned morphological data (see Table 3)

Not sampling for ancestors (*R* = 1) provided younger divergence times that are in much stronger agreement with the fossil record compared to sampling for ancestors in the current dataset, with differences for the age of the root ranging between ~ 23 Myr (between *R* = 0 vs *R* = 1 using the uncorrelated clock model) to ~ 40 Myr (between *R* = 0 vs *R* = 1 using the autocorrelated clock model). This difference is reduced to ~ 20Myr for both clock models for the age of the Sphenodontia clade and reduces progressively towards tip nodes (Table 1).

#### The impact of relaxed clock models

The relaxed clock model had an impact on divergence times, although not as substantial as sampling for ancestors (Table 1, Figs. 4 and 5b). When ancestors are excluded from the sampling strategy, the autocorrelated relaxed clock model (autocorrelated) provided younger divergence times compared to the uncorrelated relaxed clock model (uncorrelated). When ancestors are allowed, the uncorrelated clock model provided slightly younger divergence times. However, as noted above, this effect is concentrated on the deepest nodes and has little to no effect on nodes closer to the tips. In those node ages closer to the tips, the much smaller impact, when present, is the reverse to the one observed among the deepest nodes: the uncorrelated clock provides slightly younger ages when not sampling for ancestors, whereas the autocorrelated clock provides younger ages when sampling for ancestors.

#### The impact of the skyline FBD (SFBD) tree model

The analyses under the SFBD tree model provided the youngest root divergence time estimates under either single or multiple partitioned morphological clocks, especially under the best-fit autocorrelated clock model (Tables 1 and 2, Figs. 4 and 5c). This effect was achieved by an increase in the base of the clock rate for trees inferred under the SFBD process with both uncorrelated and autocorrelated clock models (Tables 4 and 5). However, while the increase in clock rate values under the uncorrelated clock model were very slight, the rate increase under the autocorrelated clock model was an order of magnitude higher (Tables 4 and 5), thus explaining the much greater reduction in the age of the root when the SFBD process is combined with the autocorrelated clock model.

Although all implementations of the SFBD model yielded consistent results (younger ages) and in greater agreement with the fossil record, the individual parameters for the FBD model and clock rate varied strongly among implementations. Regardless of the partitioning strategy and clock model, subdividing the FBD process into two time slices (SFBD2l—see the “Methods” section) increased relative fossilization rates for the first time slice (from the root until 145 Mya) and provided lower fossilization rates for the second time slice (from 145Mya to the present) (Tables 4, 5, and 6). Importantly, the lower fossilization rate for the second time slice provides a strong deviation from prior values (Additional file 5). The greatest deviation was provided by the SFBD (sdr) tree model, which found fossilization rate parameters an order of magnitude lower for the second time slice. Combined, these results indicate that the data provides significant information to update the prior values and match our expectations from the fossil record of a much lower rate of fossil preservation and sampling for sphenodontians after the end of the Jurassic.

As previously suspected [15], subdividing the FBD process into three time slices (SFBD3l) with the diversity sampling strategy (NoSA diversity) created highly biased relative fossilization values after *x*_*cut*_ at 66Mya (Table 1). Specifically, relative fossilization was found to be higher than for the first time slice (root to 145Mya), which falls in strong disagreement with our knowledge from the fossil record (Table 4).

For a single morphological partition, net diversification has higher values under the SFBD process when net diversification is allowed to change over time [SFBD (sdr)], irrespective of the clock model (Table 4). For multiple partitioned morphological clocks, net diversification always has similar values between analyses with the FBD or SFBD process (Table 5), unless a strong prior is placed constraining relative probabilities of extinction (LExct-Table 6). Variations on relative extinction were even milder than net speciation by implementing the SFBD process, with no substantial changes to that parameter, except under the LExct prior (Table 6).

#### The impact of multiple morphological clock partitions

**Fig. 6:**
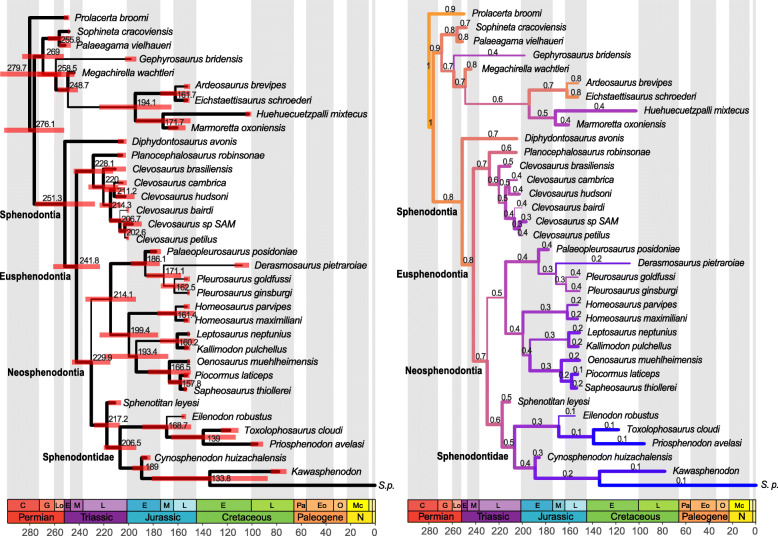
Tree with the best performing model combination with a single morphological clock partition. Model combination: autocorrelated clock + no sampling of ancestors + maximizing diversity + two-time-slices skyline FBD. **a** median ages and 95% highest posterior density (HPD) intervals (red bars) for divergence times. Estimated median ages for the tips are in Additional file 6 and will be omitted for simplicity. **b** Overall relative rates of morphological evolution. Branch colors and values indicate relative evolutionary rates. C, Cisuralian; E, Early; Eo, Eocene; G, Guadalupian; L, Late; Lo, Lopingian; M, Middle; Mc, Miocene; N, Neogene; O, Oligocene; Pa, Paleocene; S.p., *Sphenodon punctatus*

Partitioning morphological data and unlinking the clock rate variation parameters for each partition did not result in any considerable difference in tree topology to the equivalent analyses performed with a single morphological partition (Figs. 5 and 6; Suppl. Info. Fig. 8, Tables in Additional file 5). The only noticeable and consistent topological difference between single and multiple partitioned approaches is a slight decrease in tree resolution and support under the uncorrelated relaxed clock model with multiple partitions (Tables in Additional file 5). Therefore, despite the reduced amount of characters associated with each clock under a partitioned analysis, the available data was still informative enough to provide results with reasonable resolution.

On the other hand, increasing the number of morphological clock partitions does reduce the overall clock rate estimate for the trees analyzed under the autocorrelated clock model compared to equivalently modeled single partitioned analyses (Tables 4 and 5), bringing those values close to the prior clock rates values and clock rates obtained under the uncorrelated clock model. This reduction in the estimated base of the clock rate in autocorrelated trees thus resulted in longer branch durations and much older divergence times for the oldest nodes in the tree (Table 5).

Providing the non-clock tree as a starting tree for the multiple partitions analysis did not improve tree resolution nor result in any detectable difference to clock rate values or divergence times (Tables 2 and 5). However, we note that providing a non-clock tree to more complex phylogenetic problems (larger datasets that take exponentially longer time to converge) may result in substantial improvement in convergence diagnostics.

Combining the SFBD tree model with the autocorrelated clock model under multiple partitions did result in younger divergence time estimates for the oldest nodes in the tree, similarly to analyses under a single partition. However, the reduction in divergence times for the deepest nodes was not as strong as for the analyses under a single morphological clock. As a result, divergence times are still considerably overestimated when compared to the expectations of the sphenodontian fossil record and when compared to the less parameterized single partition analyses performed herein (Tables 2 and 3). It is likely that, despite containing enough data to provide reasonable resolution for tree topology, partitioning of the morphological data (each partition including about 1/3 of the data under a single partition), does not provide sufficiently informative data to estimate clock rates that can substantially deviate from prior values, thus impacting divergence times.

**Fig. 7:**
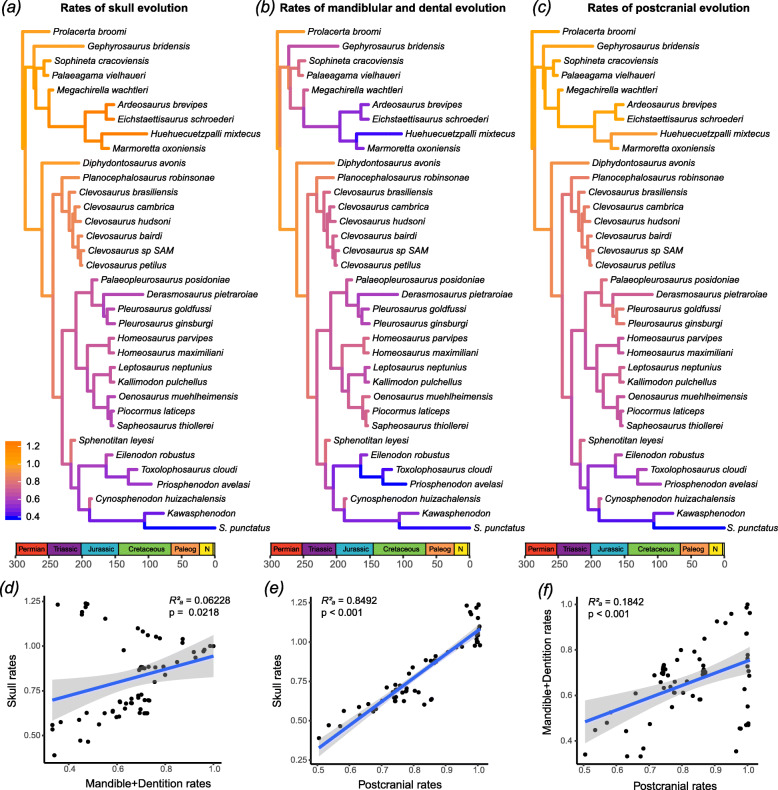
Relative rates of morphological evolution for each morphological clock partition. Rates extracted from the tree with the best performing model combination under multiple partitioned morphological clocks: autocorrelated clock + no sampling of ancestors + maximizing diversity + two-time-slices skyline FBD + truncated normal prior on the root age. Branch colors and values indicate relative evolutionary rates. **a** Rates of evolution for skull characters. **b** Rates of evolution for mandibular and dental characters. **c** Rates of evolution for postcranial characters. **d** Linear regression between skull and mandibles+dentition evolutionary rates. **e** Linear regression between skull and postcranial evolutionary rates. **f** Linear regression between mandible+dentition and postcranial evolutionary rates

Implementing strategies to reduce overestimated divergence times closer to the root (i.e., DRA correction [12]) such as attributing lower extinction probabilities (LExct), had a detectable impact on FBD model parameters, especially net diversification and relative extinction, but not on background clock rates or divergence times (Tables 3 and 6). On the other hand, placing a more informative root age prior (NoR) resulted in considerably younger divergence times, approximating the values observed under a single clock partition (Table 3). Such improvement in divergence times comes at the cost of reduced tree resolution for the MRC tree (Suppl. Data), although the topology of the MCT is very similar to the one obtained under the same models with a single morphological partition, with the exception of the placement of *Gephyrosaurus* (Figs. 3b and 7).

Importantly, clock partitioned analyses with constrained tree topologies and a fixed value for the base of the clock rate also yielded realistic divergence times comparable to the ones obtained under single clock partitioning scheme and to the partitioned clock analyses with the informative NoR root prior (Table 6). Finally, the evolutionary rate estimates for each morphological partition under the two different strategies to improve the quality of multiple partitioned morphological clocks also produced very similar results (Fig. 7 and Suppl. Fig. 8).

#### The impact of asymmetric state frequencies

Although our dataset has the better fit for a symmetric model of character state frequencies (see the “Methods” section), we assessed what would be the impact of modeling the least-fit asymmetric character state frequencies on inferences of divergence times and evolutionary rates under both a single clock and partitioned morphological clocks. The results (Suppl. Figs. 10 and 11) indicate almost no detectable impact of this assumption, as we observed the same overall pattern of rate change across lineages and across time to the results using symmetric state frequencies, as well as similar levels of correlation between rates from distinct morphological partitions.

### Divergence times and evolutionary rates

It is impossible to know the true age of divergence for each clade assessed here since the true timetree is not known (that would require simulated true timetrees). However, the assessment of empirical datasets provides a detailed understanding of how much variation and uncertainty in macroevolutionary estimates (e.g., divergence times and evolutionary rates) may result from different parameter modeling and assumptions in real case scenarios that may not be captured by simulated data. In the empirical case explored here, the available fossil data places the oldest known sphenodontians and squamates in the Middle Triassic (230–242 Mya) [13, 34]. Further, recent transcriptomic and total-evidence based studies estimate the sphenodontian-squamate split between the Middle and Late Permian (~ 270–260 Mya) [13, 37, 103] or perhaps closer to the Permian-Triassic boundary at 252Mya [104]. Therefore, given current knowledge, it would be expected for the oldest nodes in our tree (marking the divergence of early lepidosauromorph lineages) to be at least within the Permian (not older than 298.9 Mya). We thus interpret here the base of the Permian as a maximum root age threshold, and therefore, we use it to assess the direction of biases in estimated divergence times. Additionally, we utilized explicit model misspecifications, such as assuming a random sampling of taxa and implementing a least-fit clock model (uncorrelated), to also observe the most likely direction of age estimates under least-fit model assumptions. Both approaches indicate that, in the present dataset, divergence times closer to the root are biased towards unreasonably old divergence times (overestimated) and with exceptionally wide 95% HPD ranges under incorrect model selection or overparameterization (Tables 1, 2, and 3, Figs. 4 and 5a–c). On the other hand, divergence time estimates for nodes closer to the tips are considerably more robust to model misspecifications and overparameterization (Tables 1, 2, and 3, Figs. 4 and 5a–c).

By combining the most appropriate taxon sampling strategy (diversity without sampling for ancestors) and the best-fit clock model (autocorrelated) under a single clock partition, we obtain considerably younger and more conservative divergence times compared to other approaches, thus placing the age of the root of Lepidosauromorpha in the late Cisuralian at ~ 279 Mya (Table 1, Figs. 4 and 5). Given our MCT result, placing *Gephyrosaurus* and other putative early diverging Late Permian/Early Triassic lepidosauromorphs on the squamate stem, the age for the squamate-sphenodontian split is pushed back into the Early-Middle Permian boundary, implying a long stem ghost lineage for sphenodontians. Further, the Sphenodontia node suffers less from model misspecifications, but some divergence times are still up to 30 My older and have much wider age 95% HPD ranges than estimates with the preferred models, the latter placing that node at ~260Mya (Fig. 6). In sharp contrast, most node ages closer to the tips are considerably and consistently more robust to model misspecifications and partitioning strategy, with very similar divergence times and much narrower 95% HPD ranges, such as sphenodontines, eilenodontines, pleurosaurids, saphaeosaurids, among others (Tables 1, 2, 3, Fig. 4). One notable exception is the node on the split between *Sphenodon* and *Kawasphenodon*, which has much greater age uncertainty compared to other nodes closer to the tips. We attribute this much greater uncertainty to the extremely long ghost lineage leading to *Sphenodon*, owing to the almost entire lack of Cenozoic fossils for that lineage.

Evolutionary rates under a single clock partition indicate a clear pattern of higher relative rates among the oldest nodes and decreasing progressively towards the tips, especially under the best performing model combinations (Figs. 5d–f, 6). Notably, however, precision on individual relative evolutionary rate estimates are similar regardless of node age (Fig. 5g–i). This suggests that higher morphological rates at the early evolution of sphenodontians is a pattern induced by the data and not by potential modeling bias. Only in trees with highly overestimated divergence times do relative rates deviate from this pattern substantially, becoming homogeneous throughout the tree (Fig. 5d).

The decrease in relative evolutionary rates reaches its lowest values in Sphenodontidae, as both sphenodontines and eilenodontines depict relative rates which are only 10% of that observed at the time of the sphenodontian-squamate split. Relative rate increase within sphenodontians is rarely observed, with only a minor increase in relative rates (+ 0.1 relative to the parent branch) in only a few instances, such as in the branch leading to *C*. *peitulus* and branches within the aquatically adapted pleurosaurids.

When observing relative evolutionary rates across morphological partitions, we observe the same general pattern of a steady and continued decrease in rates as observed under a single clock partition (Fig. 7 and Suppl. Fig. 8). However, breaking down rate values across distinct regions of the phenotype allows a more detailed understanding of this relative rate decrease across time in sphenodontians. For instance, rates of mandibular and dental evolution drop in the early stages of sphenodontian and, most notably, squamate evolution, but increase again in certain lineages, such as Saphaeosauridae, and the branch leading to *Homeosaurus*, *Sphenotitan*, and *Cynosphenodon* (Fig. 7b and Suppl. Fig. 8b). Rates of both skull and postcranial evolution maintain high values in early lepidosaur evolution, including in early squamates, but drop considerably within Neosphenodontia (Fig. 7a, c and Suppl. Fig. 8a,c). However, rates of postcranial evolution increase again in the aquatically adapted Pleurosauridae. Finally, all morphological regions decrease in relative rates in Sphenodontidae, with the lowest rates observed in the lineage leading to the modern tuatara, *Sphenodon punctatus*. Those patterns are the same for both best performing partitioned clock MCT trees (with constrained and unconstrained tree topologies), with the only notable difference of observing a stronger drop in rates of evolution in eilenodontines under a constraint tree topology (Suppl. Fig. 8) compared to the unconstrained topology analysis (Fig. 7).

Linear regression models also indicate poor integration between relative rates of morphological evolution in the mandible+dentition against either the relative rates of skull or postcranial evolution (Fig. 7d, f). However, they demonstrate a strong predictability of the postcranial rates of evolution based on the skull rates of evolution (Fig. 7e). This result indicates a clear pattern of independent evolutionary trajectories of the mandible and dentition relative to the rest of the phenotype in sphenodontians—suggestive of each as independent evolutionary modules. At the same time, the highly similar evolutionary rates between skull and postcranium reject the hypothesis that those major functional subdivisions of the phenotype to be operating as independent evolutionary modules in sphenodontians and are instead evolutionarily integrated.

## Discussion

### Re-grafting sphenodontian systematics

Our non-clock Bayesian inference analysis provides a better resolved relationship among the major clades of sphenodontians than Bayesian inference analyses inferred with prior datasets [100, 105, 106]. This could be a result of the deletion of rogue taxa, character choice, or data scoring. In a recent study, the removal of rogue taxa from the previously available sphenodontian dataset [105] did not result in a detectable improvement in resolution for non-clock Bayesian analysis. Therefore, we attribute the improved resolution observed herein to be the result of (i) considerable corrections and revisions on the construction of the morphological dataset, avoiding issues related to logical and biological dependency, and (ii) that our new dataset includes the majority of available taxa and with most of the relevant specimens personally observed by us, improving the quality and amount of data scored.

An important systematic change to previous sphenodontian phylogenies includes the robust and consistent placement of *Derasmosaurus pietraroiae*, from the Early Cretaceous of Italy, as a member of the aquatically adapted Pleurosauridae. *Derasmosaurus* was recovered in previous phylogenetic analyses as more closely related to sphenodontines [100, 107] or in an unresolved relationship to other sphenodontians [106]. However, *Derasmosaurus* has a number of expected adaptations to aquatic environments, including an elongate body plan, reduced limbs, and late mesopodial ossification, which can be detected from illustrations in its descriptions [107, 108] and high-resolution pictures available to us (I. Paparella, pers. comm.). *Derasmosaurus *is also preserved in sediments typically associated with deposition in a marine environment [107, 108] (although we recognize that other sphenodontians are found in marine deposited sediments, but with the exception of pleurosaurids, do not possess obvious aquatic adaptations). We do not consider this phylogenetic placement to be the result of convergent evolution because (i) none of the aforementioned morphologies frequently regarded as functional changes to tetrapods secondarily adapted to aquatic environments were used as morphological characters for the present phylogeny and (ii) this result is found across all methods implemented herein, including specific ones that reduce the impact of homoplasy on final tree topology, namely implied weighting maximum parsimony and relaxed clock Bayesian inference. Our placement of *Derasmosaurus* within the aquatically adapted lineage of sphenodontians (pleurosaurids) is thus consistent with its morphology and the environment of preservation. The identification of *Derasmosaurus* as a pleurosaurid is relevant to sphenodontian phylogeny as *Derasmosaurus* represents the latest occurrence of that clade in the fossil record and is the only pleurosaurid from the Early Cretaceous. Another taxon previously associated with pleurosaurids, *Vadasaurus herzogi*, from the Late Jurassic of Germany [106], was not available for study.

Another key result is the robust placement of *Oenosaurus* within saphaeosaurids. This differs from previous analyses that found *Oenosaurus* forming a clade with *Derasmosaurus* and *Zapatodon*, and closely related to sphenodontines [100, 106, 109], opisthodontians [54], or in a poorly resolved relationship between sphenodontines and opisthodontians [43]. Here, we detected several morphological similarities in skull morphology between *Oenosaurus* and *Saphaeosaurus*, most notably the complete fusion of the marginal dentition into a single longitudinally elongate structure (Suppl. Fig. 2). This highly unusual dental morphology was considered a unique anatomical structure of *Oenosaurus* among all tetrapods [54]. However, this feature is also present in *Saphaeosaurus*, although largely overlooked owing to the absence of a detailed published description of *Saphaeosaurus*. We could not assess the presence of this feature in the closely related *Piocormus laticeps* (further preparation or CT scanning of the type material would be necessary), but it is possible that this feature is a synapomorphy of Saphaeosauridae.

Less stable results include the uncertain placement of *Gephyrosaurus* among early lepidosaurs in the relaxed clock Bayesian inference analysis with the best fit (autocorrelated) clock model. Interestingly, nearly all recent analyses using previous sphenodontian datasets (with the exception of [101, 109]) also found *Gephyrosaurus* in a poorly resolved relationship between sphenodontians and squamates [100, 105, 106, 110], or nesting within squamates [43, 100], thus not forming a monophyletic Rhynchocephalia. Other analyses, usually using older versions of a previous dataset, did not include squamates (apart from the single outgroup tip) to enable testing the placement of *Gephyrosaurus* among early lepidosaurs, or did not include *Gephyrosaurus* at all [47–54]. On the other hand, it has been suggested that incomplete phylogenies, not including a homogeneous representation of taxa throughout the entire chronological history of the group, may generate important biases to tree topology [111]. The partial sampling of early squamates (for outgroup comparison) in the present and all previous sphenodontian datasets thus may have some influence on the placement of *Gephyrosaurus*. The only analyses with a broad scale sampling across time scales of both sphenodontians and squamates ([13] and subsequent expansions [38, 112, 113]) finds *Gephyrosaurus* as the sister taxon to sphenodontians (following its traditional placement in a monophyletic Rhynchocephalia), although not as strongly supported as other sphenodontian clades. We currently consider the placement of *Gephyrosaurus* as ambiguous.

The concept of the name Rhynchocephalia was initially conceived to include *Sphenodon punctatus* upon the realization of its quite distinct systematic placement relative to squamates among reptiles by Günther [114]. However, the concept was later expanded and for decades it was used to include both sphenodontians and rhynchosaurs [115, 116], which was later refuted since the first computer-based phylogeny of reptiles placed rhynchosaurs as a lineage of archosauromorphs [117]. Therefore, for the past three decades, the concepts of Rhynchocephalia and Sphenodontia have become nearly synonymous with each other, the only difference between them being the placement of *Gephyrosaurus* as a non-sphenodontian rhynchocephalian [47]. Given the excessive redundancy the latter utilization creates, and the ambiguous placement of *Gephyrosaurus* (and therefore the instability of the modern usage of the term Rhynchocephalian), we suggest here the sole utilization of the term Sphenodontia and abandoning the term Rhynchocephalia, and the use of a phylogenetic-based definition of the term Sphenodontia (see the “Clade definitions” section in the “Results” section).

### Biases in ancient divergence time estimates

Similar to patterns observed for total-evidence and molecular datasets [12, 14], our recovered median age estimate among the oldest nodes on the tree were highly variable and dependent on sampling strategy and model implementation. Additionally, the precision of individual age estimates (95%HPD ranges) always decreases towards older nodes (Fig. 5a–c). These results indicate a systematic bias on divergence time estimates towards older and less precise ages for nodes closer to the root regardless of data type and that caution should be taken when interpreting divergence times for the earliest branching clades in relaxed clock Bayesian inference. To limit the bias, we recommend extensive outgroup sampling where possible, especially for datasets aimed towards divergence time estimates, and note that the most reliable estimates will be obtained closer to the crown. Importantly, certain sampling strategies and appropriate models highly decrease the bias towards overestimated divergence times among the oldest nodes (see the “Results” section and further discussion below).

To our knowledge, this is the first time that the considerable negative impact of sampling for ancestors in morphological datasets such as the one utilized here (where distantly related species are used to assess higher-level phylogenies) has been detected. Previously, one study [31] indicated that sampling for ancestors had an impact on divergence time estimates using the BEAST2 evolutionary package [82]. However, the latter did not assess the most likely direction of bias on such estimates and the potential explanation for such differences. We suggest that this bias is caused by the extremely low probability that the 38 sampled species over the course of 240 million years from various parts of the planet actually represent direct ancestor-descendants, thus unnecessarily increasing the number of parameters and move proposals for the analysis. Additionally, ancestors may lead to an increase in branch durations, as fossils placed as intermediate stages between successive speciation events (within a lineage instead of branching from it and becoming an extinct tip) will increase the number of character transformations associated to that lineage. This is expected to be especially relevant in the case of datasets that sample for autapomorphies, such as herein. Those autapomorphies will increase the total number of character substitutions (i.e., increase in branch length) between speciation events in order to account for the necessary additional transformations leading to ancestors, and from ancestors to the subsequent cladogenic event, instead of accumulating on the side branch represented by an extinct tip. Unless evolutionary rates are considered to increase during those branches with ancestors (mitigating the impact of a higher number of character changes on increasing branch duration), the increase in branch lengths will result in longer branch durations.

We suspect that the negative impact of trying to sample for ancestors in higher-level phylogenies (i.e., sampling taxa across various orders/families, or across vast period of geological time) is not exclusive to the present dataset as its inferred causes are inherent aspects of many broad scale phylogenetic problems. For instance, there still are several logistical challenges for constructing densely sampled morphological datasets (e.g., specimen accessibility, number of high-quality CT scanned specimens in online repositories) and the vertebrate fossil record is frequently incomplete and sometimes with few phylogenetically informative specimens. Similar examples of higher-level phylogenies with chronologically sparse taxon sampling—low taxon number:age of the tree ratio ratios (< 1, and frequently < 0.5)—include recently published datasets on pterosaurs [118], dinosaurs [119, 120], early tetrapods [56], gnathostomes [121], mammals [122], diapsid, and squamate reptiles [13, 37], among many others. Notably, many of those datasets comprehend the largest taxonomic sampling for their respective taxonomic groups using morphological data, and increasing taxonomic sampling to considerably higher levels would be simply impossible on the short- to mid-term as some of them already include most or all of the informative fossil taxa available (such as herein). Therefore, we consider that future applications of the FBD tree model in broad scale phylogenies using morphological data (for extinct lineages or in total-evidence dating) should test the impact of sampling for ancestors.

Nevertheless, it is not straightforward to test whether sampling for ancestors should be accounted for in the tree model and whether it will negatively impact resulting evidence times. The simplest solution would be assessing model fit using Bayes factors, which can be extremely useful for model comparison of both hierarchically nested and non-nested models [74], and formed the basis of our model for comparison among distinct probability mass functions governing rate variation among characters and different clock models (see above and the “Methods” section). However, as previously suggested [11, 15], when models differ by a large number of parameters or have different dimensionalities, such as different tree models (e.g., variations on the FBD model and sampling strategies) or calibration approaches (e.g., node vs tip-dating), they represent the simultaneous change of several parameters that make model performance comparison extremely more complex in a manner that may not be fully captured by comparing marginal model likelihoods only. Despite this theoretical issue, we still attempted to assess marginal model likelihoods for the four main combinations of sampling strategies (fossiltip, random, NoSA diversity, diversity) using the best-fit clock model (autocorrelated), with and without clock partitioning. However, we struggled to obtain convergence among independent runs with data partitioning for all categories except diversity, which we attribute to the excessive number of free parameters given dataset size. The marginal likelihoods were higher for strategies that do not include sampled ancestors for the single partition analyses that reached convergency between runs: fossiltip (− 1190.21), random (− 1195.67), NoSA diversity (− 1192.46), and diversity (− 1198.24), which are also the models that are in much greater agreement with the fossil record (see the “Results” section). Given the theoretical and practical issues above, we suggest caution on the interpretation of complex model comparisons on the marginal likelihoods only, especially given the relatively small difference in log likelihoods among those strategies. Instead (or in addition to assessment of marginal likelihoods), we suggest directly observing the results from both approaches and their respective fit to ad hoc expectations from the fossil record and the evolutionary process, as performed herein—when divergence time estimates seem to be overestimated for the oldest nodes on the tree compared to the fossil record or other external sources of data. This would be mostly expected in sparsely sampled datasets, as argued above, and also in phylogenetic trees where the rate of extinction is higher than the rate of origination [123], and when most or all taxa are sampled from widely disparate geographic regions (e.g., across various continents for global analyses of clade relationships). The influence of high extinction rates may be especially relevant in datasets focusing on periods of mass extinctions or lineages that have drastically reduced their diversity through time, as in the case of sphenodontians. On the other hand, datasets focusing on periods of taxonomic radiations or including taxonomic groups with evidence for a linear or exponential increase on speciation rates across time will be, in fact, more likely to include direct ancestors and descendents [123].

Our results may also help explain overestimated divergence times provided in the recent literature related to the sampling of fossils. Analyses using the FBD model with sampled ancestors suggest that focusing taxonomic sampling only on the oldest known fossils may generate overestimated divergence times because the FBD process assumes a random sampling of fossil taxa [124]. This may be corrected by an extensive sampling of fossil species, although unpractical under many different circumstances [124]. On the other hand, ongoing research using the uniform tree prior (which does not include sampled ancestors) indicates that it is actually advantageous to focus fossil taxon sampling on the oldest known taxa [76]. The discrepancy between these results suggest the tree model, and not fossil sampling strategy, may be the cause of overestimated divergence times, an explanation that is supported by our findings herein. We implemented a more homogeneous sampling of fossil taxa through time (only limited by the fossil record of the group), thus not focusing on the oldest known fossils only. Yet, we still observed important overestimates of divergence times when using the FBD model with inappropriate prior parameters and when sampling for ancestors, similarly to the results of the first study [124]. We thus suspect that the overestimated ages observed in [124] may be a consequence of implementing the FBD model while always accounting for ancestors and with sparse sampling of taxa (~ 1% of total taxonomic diversity), which matches the conditions under which we would expect to observe biases from accounting for sampled ancestors.

### Clock models have a major impact on background and relative evolutionary rates

Divergence time estimates that are in stronger agreement with the fossil record are those obtained when ancestors are not being sampled and when the preferred clock model (autocorrelated) is utilized (Table 1). As seen from Table 4, the autocorrelated clock choice results in estimates for the base of the clock rate that are always two to three times higher than prior rates (Tables in Additional file 5) and also higher than the uncorrelated clock rates in single partition analyses. Under multiple clock partitioning, the base of the clock rate was still higher using the autocorrelated clock model, although only slightly (Table 5). Absolute branch lengths (i.e., accumulated number of substitutions) and clock rate values are first estimated by Mr. Bayes and subsequently used to estimate divergence times and create summary clock trees. Therefore, we suggest that it is the higher background morphological rate of evolution under the autocorrelated model that increases the likelihood of faster character substitutions along the entire tree, and as a consequence, the likelihood of shorter branch durations (i.e., chronological time) in our tree modeled with the autocorrelated clock compared to a tree inferred with the uncorrelated clock model.

It has been previously argued that autocorrelated clocks will increase divergence times owing to the “smoothing effect” of the autocorrelation parameter *p*(*v*) on evolutionary rates (avoiding drastic rate shifts across neighboring branches), which would tend to increase overall branch duration [11]. This has led to previous suggestions that autocorrelated models lack reliability [15]. However, as shown above, despite observing the smoothing effect in our results, it did not result in overestimated divergence times compared to the uncorrelated clock model. Recent studies using molecular data also found the uncorrelated clock model to yield considerably older divergence times closer to the root compared to the autocorrelated clock model [20, 22].

It is not straightforward why the relaxed clock model has such an influence on the estimated values for the base of the clock rate, since the clock model parameterizes the pace of rate change among lineages, whereas the inferred values for the base of the clock rate are sampled from a separate prior—the lognormal prior distribution for the clock rate parameter when using the autocorrelated clock. It is possible that, if the data implies a large number of substitutions in some of the early branches of the tree, the uncorrelated clock will accommodate some of those by expanding relative clock rates on only a few branches, thus not requiring the base of the clock rate to deviate considerably from prior values. On the other hand, the autocorrelated clock does not accommodate sudden changes and, in order to accommodate for large number of substitutions on early branches, there are two viable options: (i) to increase branch duration, in order to accommodate a larger number of substitutions, therefore overestimating divergence time estimates [11], or (ii) background rate values will deviate more strongly from prior parameters to much higher values, therefore accommodating those large number of substitutions without impacting branch duration as strongly. Here, we find the latter option to be operating, as we observed a much higher base for the clock rate than prior and uncorrelated clock estimates (Tables 4 and 5), while not deviating strongly from those background rates across neighboring branches (Fig. 5), resulting in a relatively short branch duration and overall younger age for the oldest tree nodes (Figs. 4 and 5). This effect may also explain the contrast of results in previous studies using molecular data concerning the performance of different clock models [11, 15, 20, 22]. We therefore suggest that future studies estimating divergence times and evolutionary rates should always perform model marginal likelihood comparisons between clock models and assess the potential bias that may be introduced by model choice, as the behavior of the clock model seems to be dataset dependent.

### The skyline FBD (SFBD) provides reliable macroevolutionary parameter estimates in fossil-rich phylogenies

Recent simulation-based studies on the FBD tree model have confirmed that it is capable of obtaining reliable macroevolutionary parameter estimates under intensive fossil sampling strategies [2]. However, there have been comparatively limited attempts to understand the impact of the skyline extension of the FBD tree model. Previous studies implementing the SFBD tree model in combined-evidence datasets (thus inclusive of morphological data) detected an opposite pattern to the one observed here: a considerable increase in divergence time estimates instead of a reduction of the same [12, 15]. In their strategy of implementation of the SFBD model, previous studies placed the first rate shift time very close to the root. This choice seems to have forced the tree prior to expand branch durations between the time of origin of the FBD process and the first rate shift time in order to provide meaningful estimates of the FBD parameters. The practical consequence is a deep root attraction (DRA) problem [12], with the age of the root being overestimated by dozens of millions of years when compared to the other analyses conducted herein and the fossil record [12, 15]. When the first-rate shift time is located at a much younger time than the priors for the age of the root, as implemented here, there is enough time and data between the root and the first rate shifting point for the net speciation, turnover, and relative fossilization parameters to be meaningfully estimated without artificially pushing root ages back in time. Therefore, strategies similar to the one implemented here for indicating rate shift times for the SFBD tree model seem to be able to provide improved estimates for the parameters on the FBD model even in morphological datasets (much smaller in size than molecular datasets) and reduce the impact of DRA by reducing the age of the root.

As shown in our results, the relative fossilization parameter (*s*) is the most flexible one in the SFBD process for the kind of dataset analyzed here. Parameter values change in a consistent and predictable manner across different strategies of data partitioning and clock model, always yielding results that are consistent with the expectations from the fossil record. The only instance of bias is when we model a shift in parameter values after *x*_*cut*_ at 66 Mya for a diversity sampling strategy, as previously predicted [15], specifically obtaining overestimated relative fossilization rates. Although we did not observe a negative impact of allowing all three FBD parameters (net speciation, turnover, and relative fossilization) to vary across time slices, net speciation and turnover did not change as strongly as relative fossilization across time bins. The situation may change for larger datasets with denser taxon sampling, and in which more data is provided to estimate those parameters. However, for many morphological datasets (small-medium sized taxon numbers), allowing only the relative fossilization to shift across times provides a balance between reducing the number of free parameters (reducing chances of overparameterization) and still accounting for important parameter rate shifts and more realistic divergence times and evolutionary rates.

### Morphological clock partitioning: escaping from stuck clocks towards reliable divergence times and evolutionary rates

One of the potential risks of increasing the number of data partitions is to increase the number of free parameters and thus overparameterizing phylogenetic inference using probabilistic methods [14]. This is especially true in the case of morphological data, as the much smaller size of morphological datasets compared to molecular datasets (especially in the genomic era) will tend to create morphological partitions that do not have enough data to modify prior information, or to reach the stationarity phase/convergence between runs. Although we did not detect a considerable decrease in resolution in tree topology in our dataset by increasing the number of partitions, we did detect an impact of partitioning on clock rate values and, subsequently, divergence time estimates. This result may also explain the observed increase in divergence time estimates for previous studies inclusive of morphological data in which separate morphological clocks were also attributed to each partition [10, 14]. Those studies promptly recognize that the observed increase in divergence times was likely a consequence of overparameterization given the nature of morphological datasets. Here, we further suggest that overestimated divergence times in partitioned morphological clocks are, more specifically, a result of the reduced amount of data in morphological partitions being unable to modify the clock rate prior distributions, which in turn, may get stuck at relatively low levels (closer to prior values), yielding excessively long branch durations (and divergence times).

On the other hand, simply ignoring the potential for different regions of the phenotype to evolve at very distinct rates provides an oversimplification of the evolutionary process, and such “underparameterization” (such as “lumping” partitions together) may lead to greater phylogenetic error than an equivalent degree of overparameterization (e.g., “splitting” partitions), as previously detected for molecular datasets [91, 93, 94]. Therefore, studies using relaxed morphological clocks should find a balance between ignoring the potential for rate variation among morphological evolutionary modules (no clock partitioning) and overparameterization (excessive clock partitioning and uninformative priors).

One immediate solution to this problem is constraining the prior values for the base of the clock rate to values from the single morphological clock analysis and constraining the tree topology. As expected, the implementation of this approach brought divergence time estimates among the oldest nodes in the tree to values very similar to the ones with the best performing single clock analysis (Suppl. Fig. 7 and Table 3). Additionally, we also detected very similar divergence times and resulting tree topologies to the best performing single clock analysis by simply placing a hard maximum bound on the age of the root (Table 3), despite lower clade support values compared to single clock trees (Suppl. Fig. 6 and Table 3). Finally, the estimated rates of evolution for each morphological partition along the tree presented very similar values between both approaches (Fig. 7 and Suppl. Fig. 8).

In summary, morphological clock partitioning in a standard sized morphological dataset may suffer from overparameterization when trying to estimate macroevolutionary parameters. However, minor constraints on the age of the root, or fixing clock base rate and tree topology (similarly to phylogenetic comparative method approaches), does yield reliable divergence times and very similar results concerning relative evolutionary rates for each morphological partition (Tables 3 and 6, Figs. 6 and 7, Suppl. Fig. 8). Finally, despite the potentially negative impact of assuming symmetric state frequencies for phylogenetic inference when there is heterogeneity of state frequencies among characters [76], we find that the opposite model mismatch—allowing for asymmetric state frequencies when a symmetric model has a better fit to the data using relaxed clocks—did not result on any detectable impact to the overall patterns under both single and partitioned clock analyses (Suppl. Figs. 9–10). This result might reflect the fact that this symmetric model is essentially a more restrictive version of the asymmetric model (equivalent to an asymmetric model with high alpha values). Therefore, if the alpha value is sampled from a wide uniform distribution (not fixed), it is possible for the asymmetric model to recover very high alpha values, and relatively symmetric state frequencies. Indeed, our mean alpha was 2.7 (single clock) and 4.4 (partitioned clocks), thus treating state frequencies with a low level of asymmetry. However, we note that runs under the asymmetric state frequencies model take much longer to converge (3–7 times longer) and are more prone to overparameterization. This may make them impractical for datasets with much higher taxon sampling as those usually will already take considerable time to converge, especially when combined with molecular data.

### Morphological clocks as a tool to test evolutionary integration and modularity

Estimated rates of morphological evolution across separate functional subdivisions of the phenotype as performed here (skull, mandible+dentition, postcranium) revealed that whereas some of those regions evolve at distinct evolutionary rates across lineages (mandible+dentition relative to the skull or postcranium), other morphological regions evolve at a very similar pace across lineages (skull and postcranium). The latter leads to the rejection of our initial hypothesis that all of those separate functional subdivisions follow independent evolutionary trajectories and that phenotypic subdivisions adapted to very distinct functions [e.g., feeding (skull) vs. locomotion (postcranium)] may still be evolving in conjunction—i.e., are evolutionary integrated.

While testing integration across distinct morphological components of the sphenodontian phenotype is not our primary goal, our results suggest that the application of partitioned morphological clocks can be utilized as a phylogenetic-based approach to detect evolutionary integration and modularity across major anatomical regions of the vertebrae body. Functional subdivisions of the phenotype or another criterion (e.g., correlation analysis of the character data matrix) can be used as a candidate morphological model to be tested by morphological clocks, which will in turn enable the assumption and direct measures of evolutionary mosaicism when inferring phylogenetic trees.

### Sphenodontian macroevolution: the return of a “living fossil” and the importance of accounting for mosaic evolution

It has been previously argued that the New Zealand tuatara, *Sphenodon punctatus*, cannot be characterized as a “living fossil” because it is not a product of evolutionary stasis based on a large variety of skull shapes among fossil sphenodontians [42], and extremely fast rates of molecular change in the modern *Sphenodon* compared to other vertebrates [125]. However, as previously acknowledged by others [43], large ancient morphological disparity in a clade does not necessarily mean that such disparity was achieved at fast rates of evolution [126] or that those rates were sustained across time. Additionally, comparing rates of molecular evolution in a species separated by at least 240 million years from its closest living relative ignores all the variation that could have existed during the evolution of the entire clade. The high rates of molecular evolution detected for *Sphenodon* were measured from mitochondrial sites only [125], which are highly variable (i.e., fast evolving), when compared to nuclear sites in broad scale taxonomic datasets among many vertebrates, including lepidosaurs [13, 127, 128]. Finally, subsequent studies found much slower molecular rates on the branch leading to *Sphenodon* based on a larger sample of loci (including nuclear loci) [37] and based on full genomic data [129].

A recent study [43] utilized geometric morphometric data from the mandible of *Sphenodon* and several species of fossil sphenodontians to estimate morphological disparity and evolutionary rates in the group. That study found that the modern *Sphenodon* falls close to the centroid of the morphospace of Triassic sphenodontians (indicating morphological conservation of its jaw morphology). Additionally, despite detecting heterogenic rates of evolution in sphenodontians, the *Sphenodon* lineage had significantly low rates of jaw evolution. Based on these results, the authors suggest that the modern tuatara does conform to what would be expected for a “living fossil” [43].

Our results also find extremely low rates of evolution on the tuatara lineage [43], reinforcing the idea of *Sphenodon* as a “living fossil.” This is supported both by the total relative rate of morphological evolution and by the clock partitioned analyses investigating evolutionary rates across major individual morphological subdivisions. However, we note that the low rates of evolution in sphenodontines, including *Sphenodon*, are one of the major patterns in common across all estimates of evolutionary rates (Figs. 6 and 7, Suppl. Fig. 8). Most other clades display distinct evolutionary rates depending on the morphological subdivision being assessed, with some clades demonstrating a clear increase in relative rates of evolution that cannot be captured by analyzing total rates of morphological evolution or only one morphological partition. Therefore, we suggest that studies focusing on morphological rates of evolution should take into consideration the variation on rates among morphological subdivisions whenever possible, such as by the implementation of partitioned morphological clocks.

## Conclusions

Despite the oversimplistic substitution model utilized to handle morphological data in probabilistic approaches to phylogenetics (the Mk model [24]), a wave of recent studies have demonstrated that even in such circumstances, probabilistic methods still reach higher accuracy levels than previous criteria (maximum parsimony), with Bayesian inference performing the best regardless of missing data, characters and taxon number (e.g., [23, 26, 27]). Additionally, the several advances in modeling relaxed clocks in the last decade have made it possible to incorporate a variety of macroevolutionary parameters for the analysis of morphological data in Bayesian phylogenetics. Such parameters include introducing rate variation among lineages, providing stronger tree calibration and accounting for fossil placement uncertainty with the inclusion of fossils as tips, accounting for fossil age uncertainty, the availability of tree models accounting for relative fossilization rates, and shifts in those rates across time. Altogether, morphological clock Bayesian inference provides a powerful parametric approach to infer evolutionary patterns and relationships among living and extinct lineages. Yet, the behavior of morphological data across various available models remains poorly explored and understood.

Our findings indicate considerable impact on divergence times and evolutionary rates given taxon sampling strategy choice, with a notable poor performance when allowing for the inclusion of sampled ancestors, which is expected to impact similar higher-level or deep-time phylogenies. We also find a substantial impact of clock model choice and on variations of the FBD tree model on macroevolutionary parameters. Contrary to previous findings, our results indicate that stronger agreement between estimated divergence times and the fossil record can be achieved by accounting for variation of relative fossilization across time using the skyline FBD tree model, as well as parameters on net diversification and turnover rates. Further, we find that morphological clock partitioning is impacted by overparameterization as recently suggested, generating overestimated divergence times for deeper nodes on the tree, contrary to what has been observed on molecular clock partitioning. However, placing informative priors on the root age or applying constraints results in divergence times similar to single clock analyses and provides converging results of evolutionary rate variation patterns across lineages. Therefore, we strongly suggest thorough testing of different clock models, partitions, sampling strategies, and variations of the FBD model in future applications of morphological clocks.

Our thorough investigation of evolutionary relationships in a new dataset of sphenodontian reptiles reveals new results with localized but important revisions of previous hypotheses of sphenodontian relationships. We find that most sphenodontian clades originated in the Triassic, with some important lineage diversification in the Jurassic, but a sharp decrease from the Cretaceous onwards. Additionally, total relative rates of morphological evolution decline continuously throughout the entire sphenodontian tree. However, when inferring relative rates of evolution across morphological subdivisions, we find localized rate increases, such as among the aquatically adapted pleurosaurids. We also find a strong correlation of skull and postcranial rates, but a decoupling of these modules and mandibular+ dental rates of evolution in sphenodontians. This suggests that inferred morphological disparity and evolutionary rates estimated only from mandibular characters should be taken with caution. We recommend evolutionary rate studies to take into account both the general or total rate of morphological evolution as well as localized rates of evolution across the phenotype in order to reveal potentially hidden patterns generated by evolutionary mosaicism.

## Supplementary Information

**Additional file 1.** Text document containing additional methodological information and supplementary figures.

**Additional file 2.** Text document containing list of sampled taxa, along with their occurrence data, stratigraphic interval, age, anatomical bibliography, and personally observed specimen numbers.

**Additional file 3.** Text document including phylogenetic morphological characters list with individual character descriptions.

**Additional file 4.** Phylogenetic data matrices. Data matrix containing all scored taxa (38 taxa) and data matrix with all taxa used for final analysis (35 taxa).

**Additional file 5.** Excel spreadsheet containing tables with summary statistics for all posterior parameter estimates, as well as divergence times and prior parameter values (effective priors) for important focal clades.

**Additional file 6.** Input files including the dataset and all necessary coding (see Mr. Bayes blocks) to reproduce the analyses.

**Additional file 7.** R scripts used for the construction of the main text figures and statistical analyses.

## Data Availability

Supplementary Data freely available at Harvard’s Dataverse online repository [99].
