# Supplementary material for: Sphenodontian phylogeny and the impact of model choice in Bayesian morphological clock estimates of divergence times and evolutionary rates
Source: BMC Biol. 2020 Dec 7;18:191. doi: 10.1186/s12915-020-00901-5 (PMC7720557; doi:10.1186/s12915-020-00901-5)
Supplement: Supplementary file 1 — Additional file 1. Text document containing additional methodological information and supplementary figures. [file 12915_2020_901_MOESM1_ESM.pdf]

Supplementary Information

Sphenodontian phylogeny and the Impact of Model Choice and Clock Partitioning in Bayesian Morphological Clock Estimates of Divergence Times and Evolutionary Rates

Tiago R. Simões, Michael W. Caldwell and Stephanie E. Pierce

Supplementary Figures

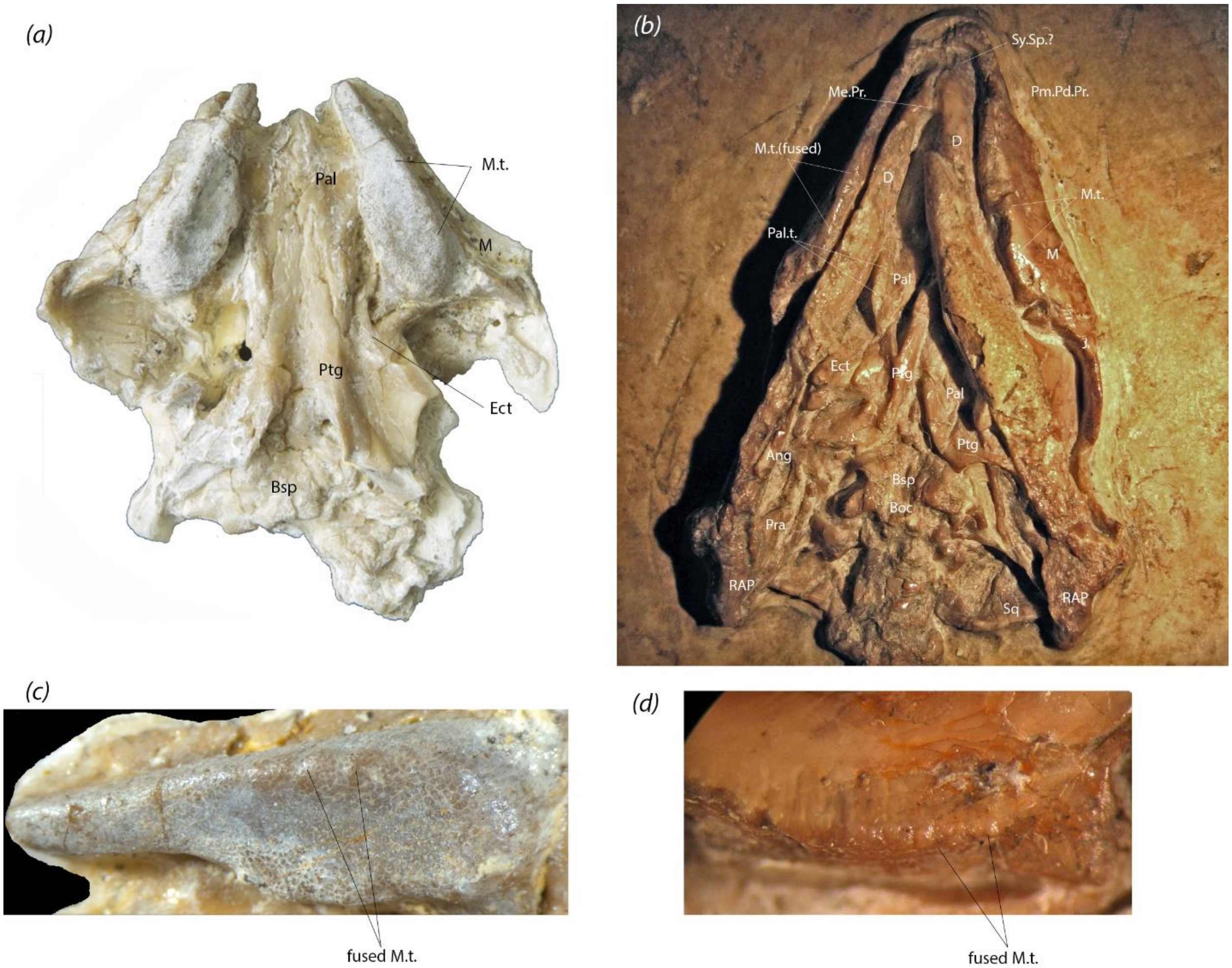

Supplementary Figure 1. Skull of *Oenosaurus* and *Saphaeosaurus* with details of their dentition. (a) Skull of *Oenosaurus* in ventral view. (b) Skull of *Saphaeosaurus* in ventral view. (c) Detailed view of the maxillary toothplate of *Oenosaurus* in occlusal view, depicting individual maxillary teeth fused into a single dental ridge. (d) Detailed view of the maxillary toothplate of *Saphaeosaurus* in occlusal view, depicting individual maxillary teeth fused into a single dental ridge.

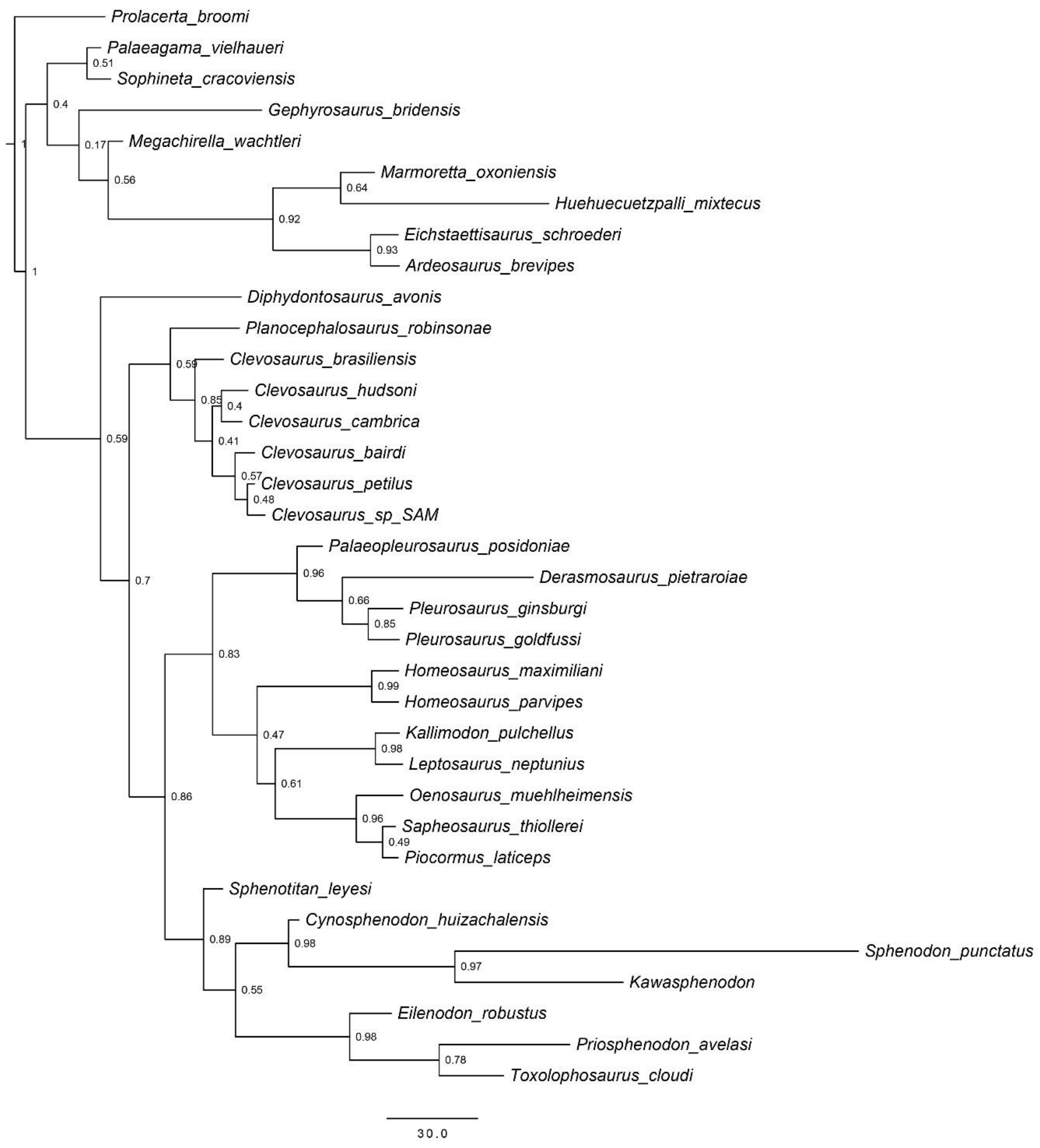

Supplementary Figure 3. Maximum compatibility tree (MCT) from single morphological relaxed clock Bayesian inference analysis implementing the best performing model combination (TK02+DvNoSA+SFBD(s)2l). Node values indicate posterior probabilities.

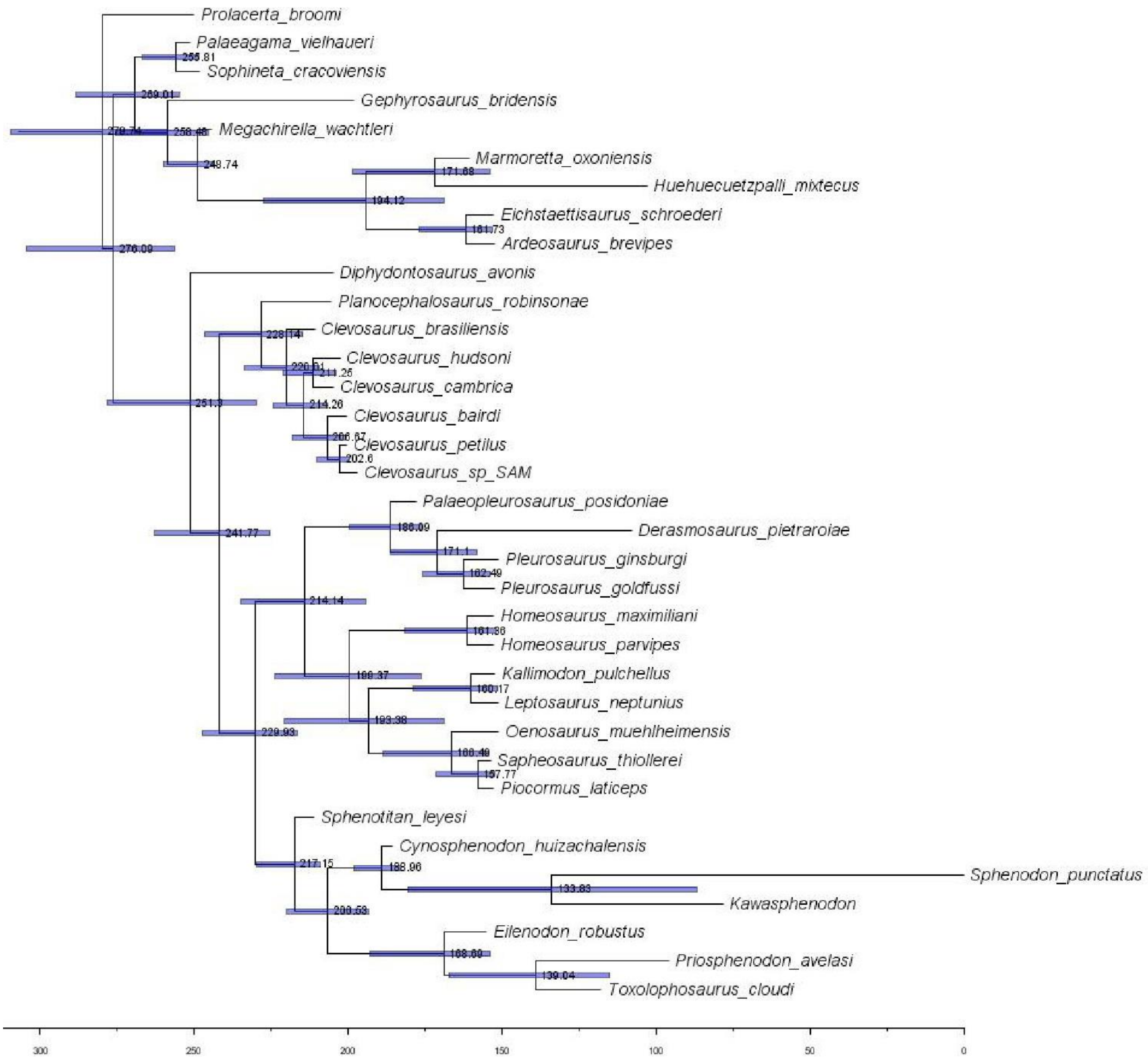

Supplementary Figure 4. Maximum compatibility tree (MCT) from single morphological relaxed clock Bayesian inference analysis implementing the best performing model combination (TK02+DvNoSA+SFBD(s)2l). Node values indicate median estimates for divergence times and node bars represent 95% HPD ranges.

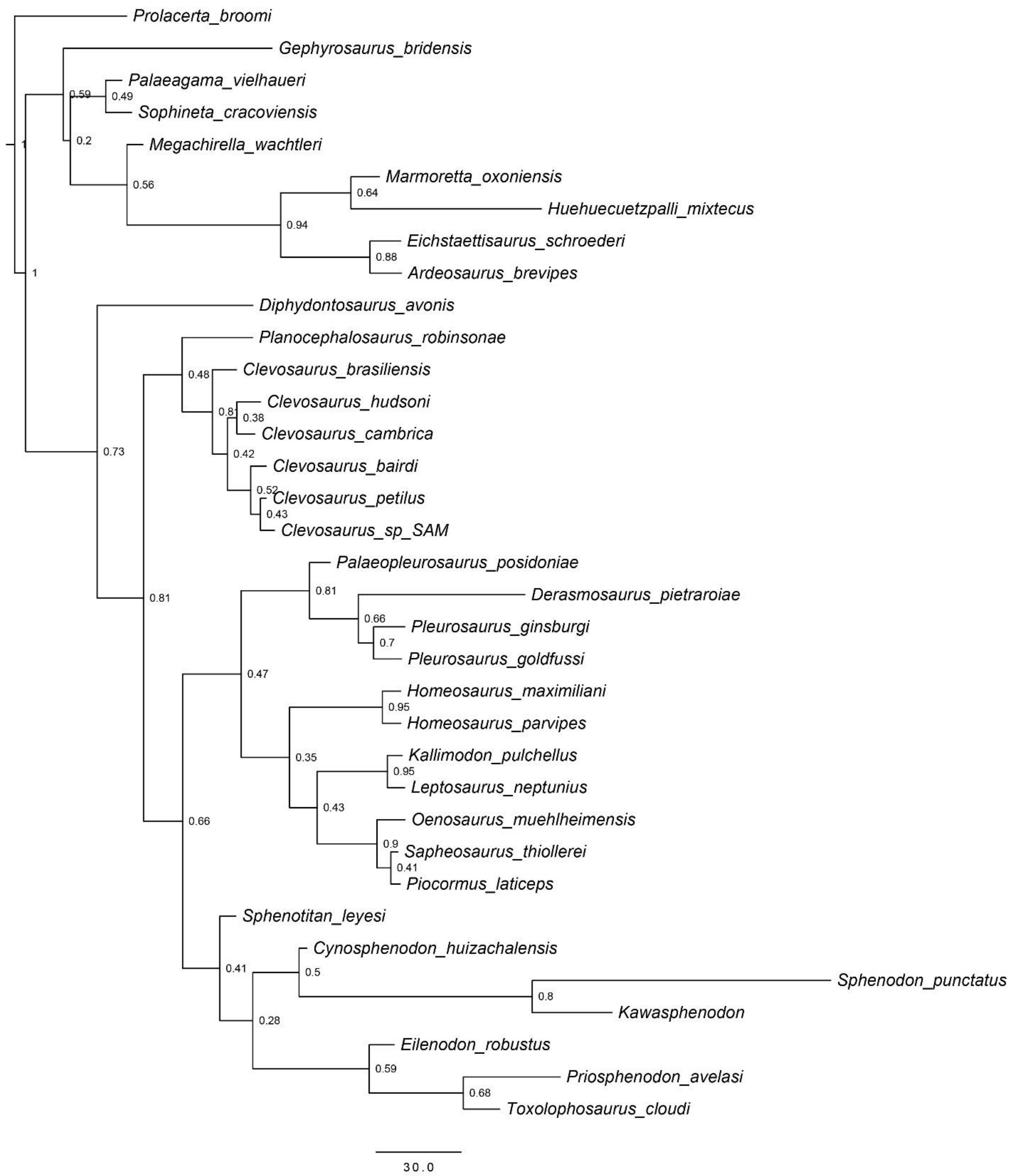

Supplementary Figure 5. Maximum compatibility tree (MCT) from the partitioned morphological relaxed clock Bayesian inference analysis implementing the best performing model combination (TK02+DvNoSA+SFBD(s)2l+NoR). Node values indicate posterior probabilities.

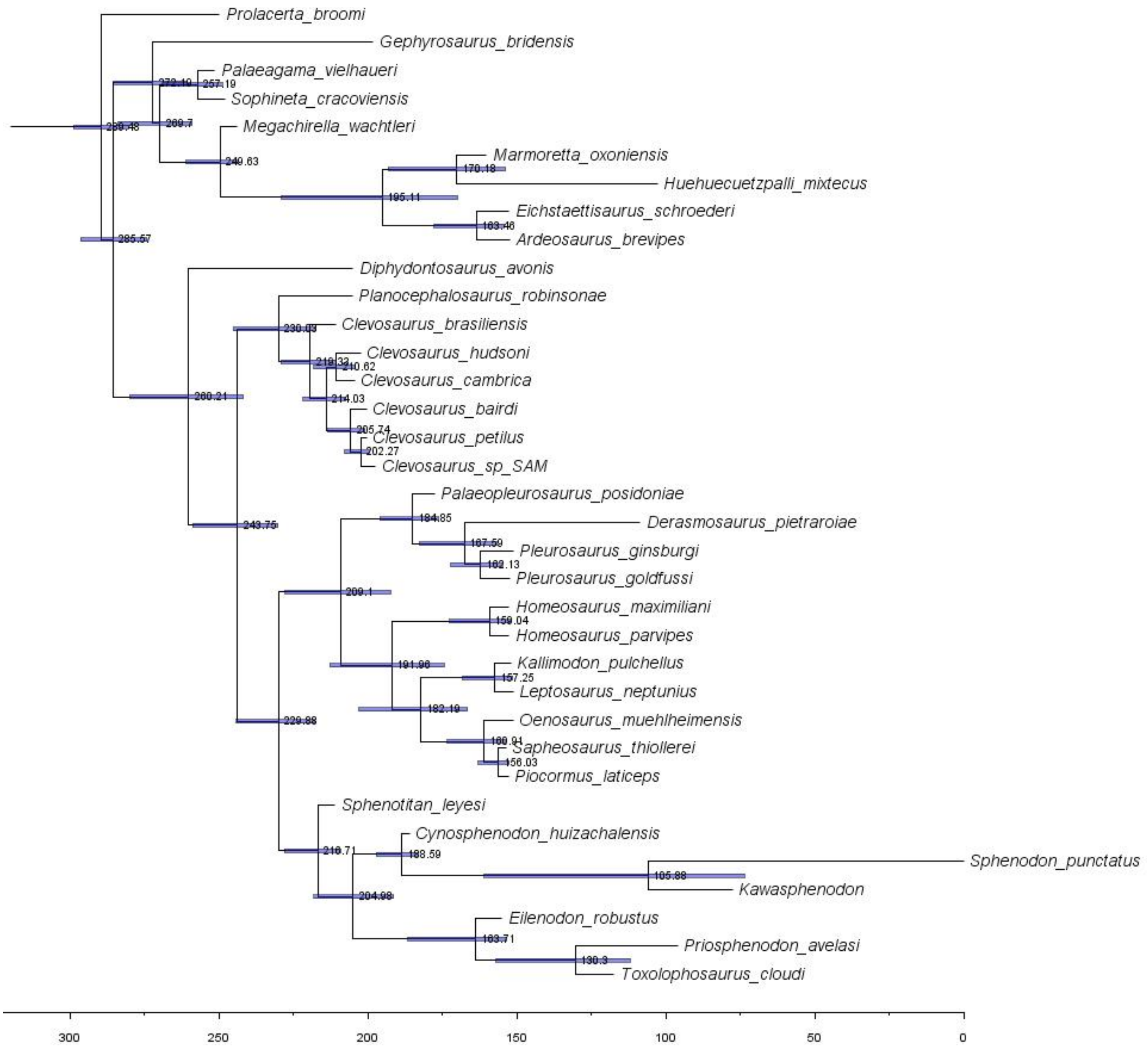

Supplementary Figure 6. Maximum compatibility tree (MCT) from partitioned morphological relaxed clock Bayesian inference analysis implementing the best performing model combination (TK02+DvNoSA+SFBD(s)2l+NoR). Node values indicate median estimates for divergence times and node bars represent 95% HPD ranges.

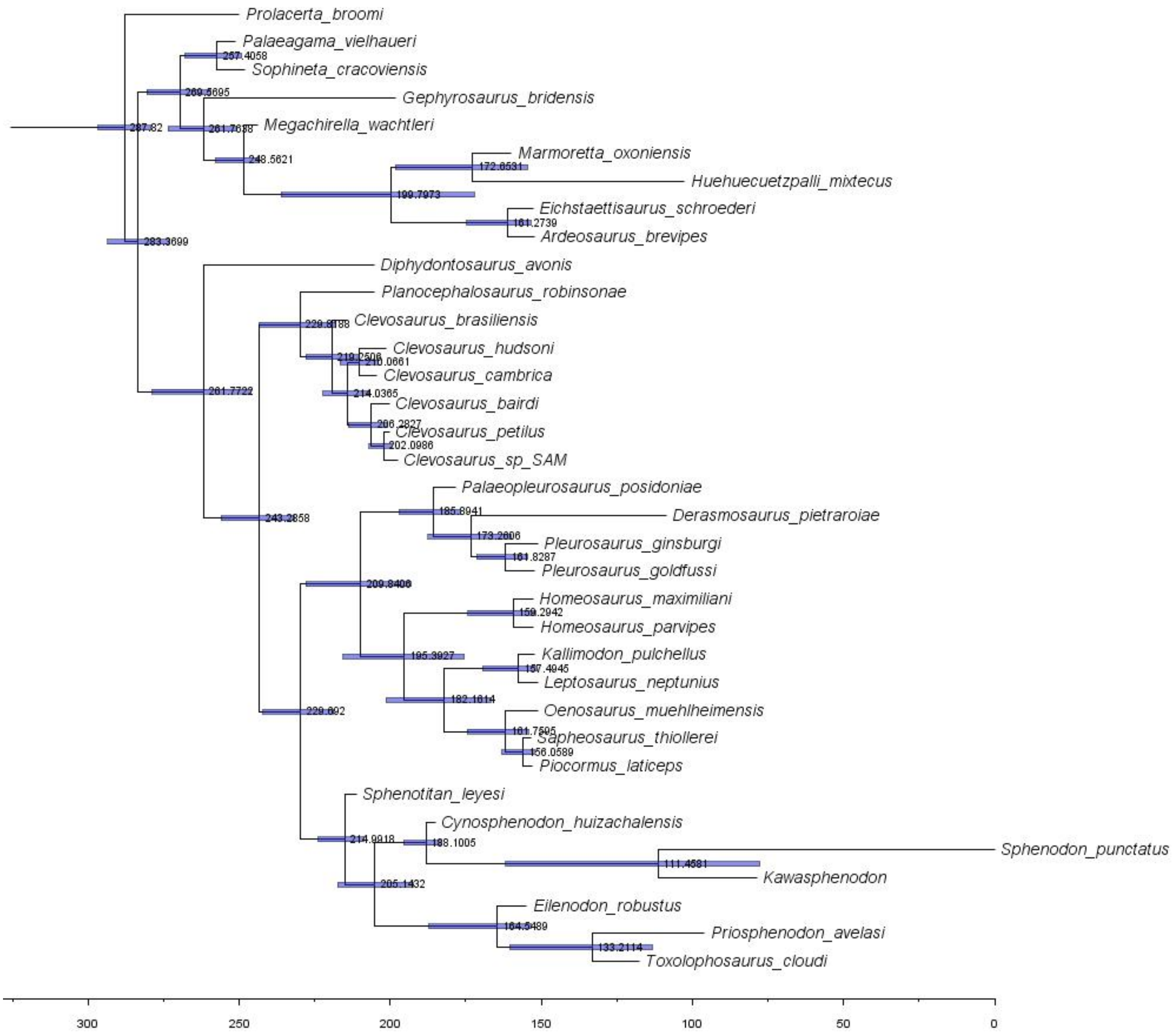

Supplementary Figure 7. Maximum compatibility tree (MCT) from partitioned morphological relaxed clock Bayesian inference analysis implementing the best performing model combination (TK02+DvNoSA+SFBD(s)2l+NoR) but with topology and base of the clock rate constrained to values from single partition tree (in Fig. 5 and Supplementary Fig. 3 and 4). Node values indicate median estimates for divergence times and node bars represent 95% HPD ranges.

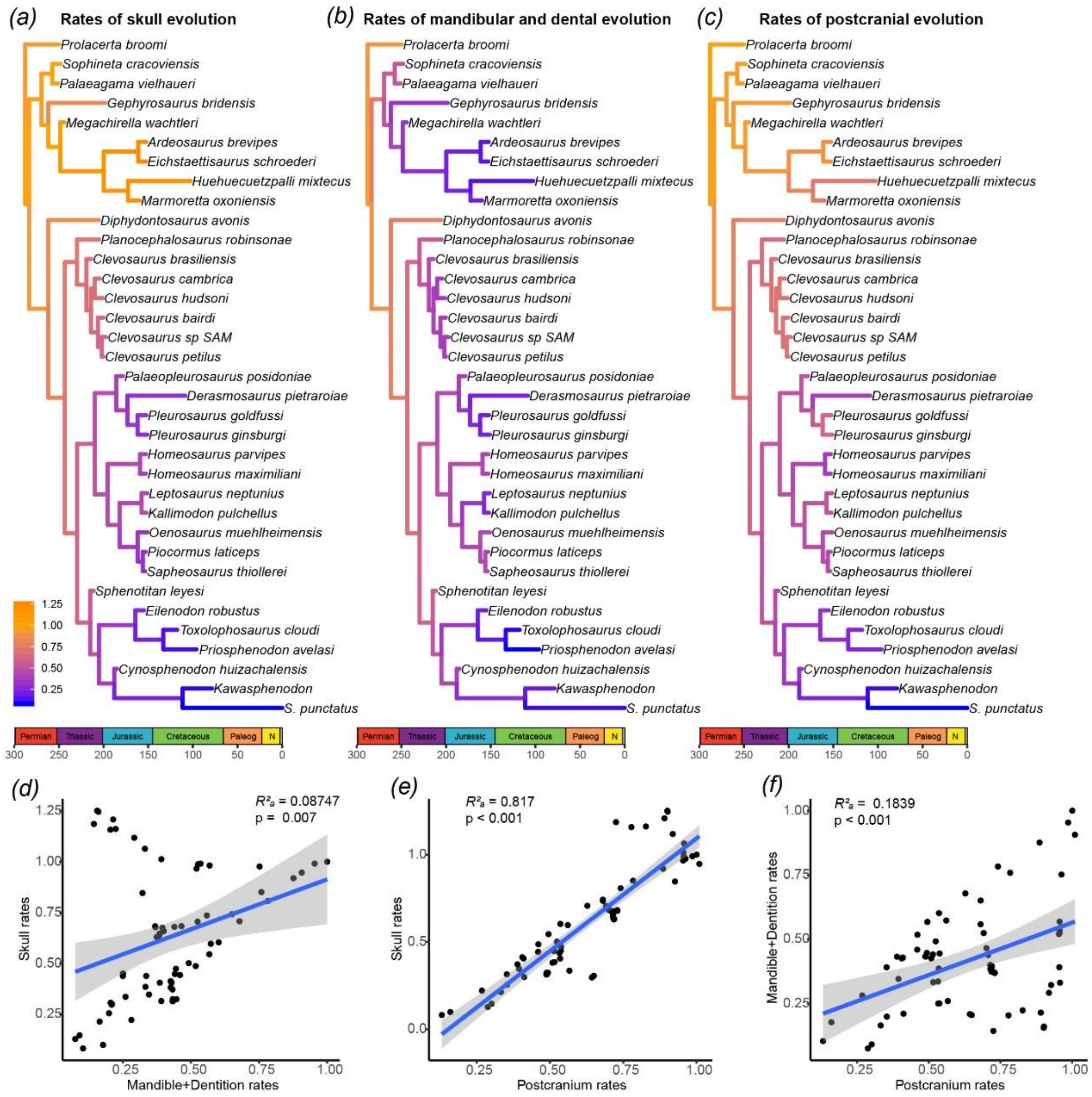

Supplementary Figure 8. Relative rates of morphological evolution for each morphological clock partition on the analysis imposing topological and background evolutionary rate constraints obtained from the best performing single morphological clock partition tree (in Fig. 5 and Supplementary Fig. 3 and 4). Branch colors and values indicate relative evolutionary rates. (a) rates of evolution for skull characters. (b) rates of evolution for mandibular and dental characters. (c) rates of evolution for postcranial characters. (d) linear regression between skull and mandibles+ dentition evolutionary rates. (e) linear regression between skull and postcranial evolutionary rates. (f) linear regression between mandible+ dentition and postcranial evolutionary rates.

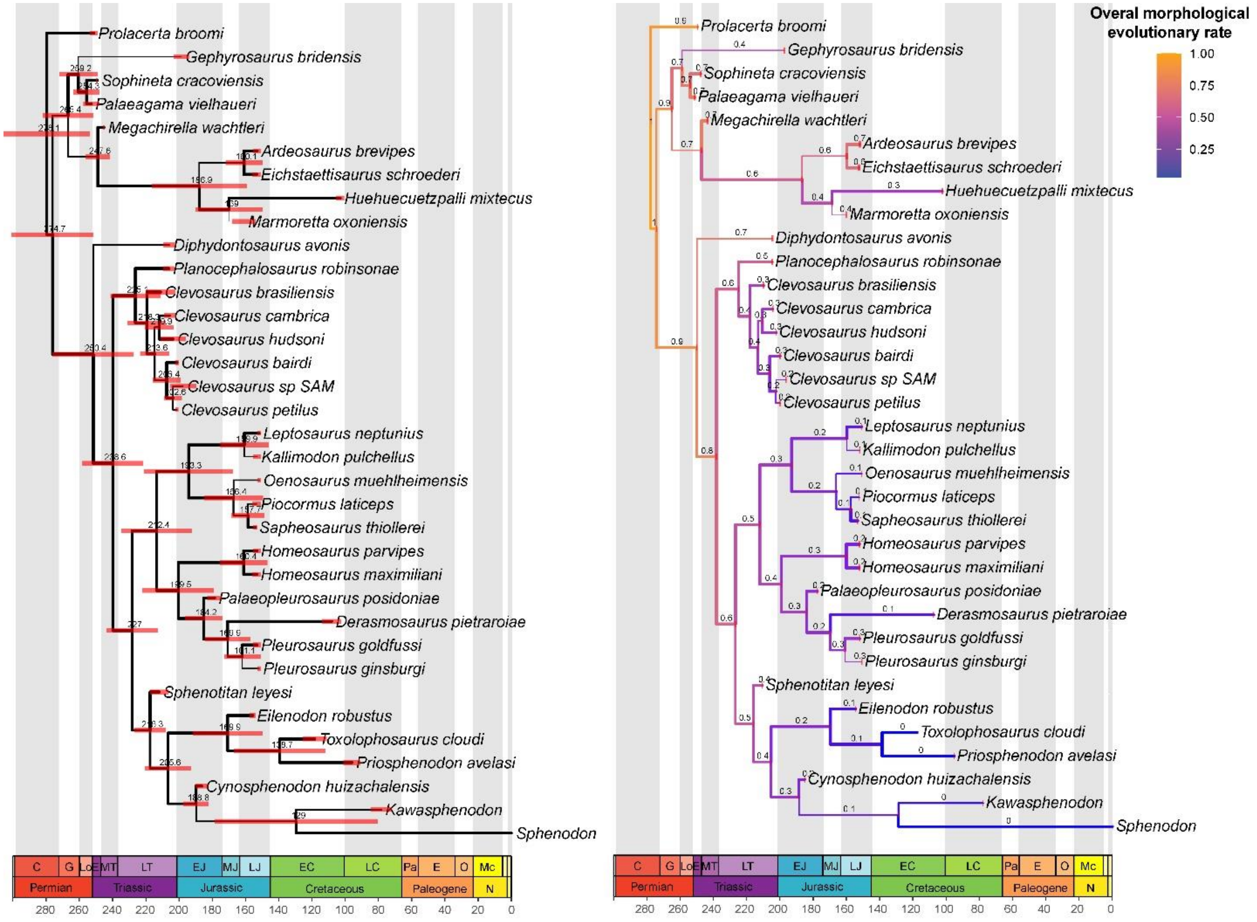

Supplementary Figure 9. Tree with the best performing tree and clock model combination with a single morphological clock partition (autocorrelated clock + no sampling of ancestors + maximizing diversity + two-time-slices skyline FBD) and asymmetric state frequencies for the model of character evolution. (a) median ages and 95% highest posterior density (HPD) intervals (red bars) for divergence times. Estimated median ages for the tips are in and will omitted for simplicity. (b) Relative rates of morphological evolution. Branch colors and values indicate relative evolutionary rates. Abbreviations: C, Cisuralian; E, Early; Eo, Eocene; G, Guadalupian; L, Late; Lo, Lopingian; M, Middle; Mc, Miocene; N, Neogene; O, Oligocene; Pa, Paleocene.

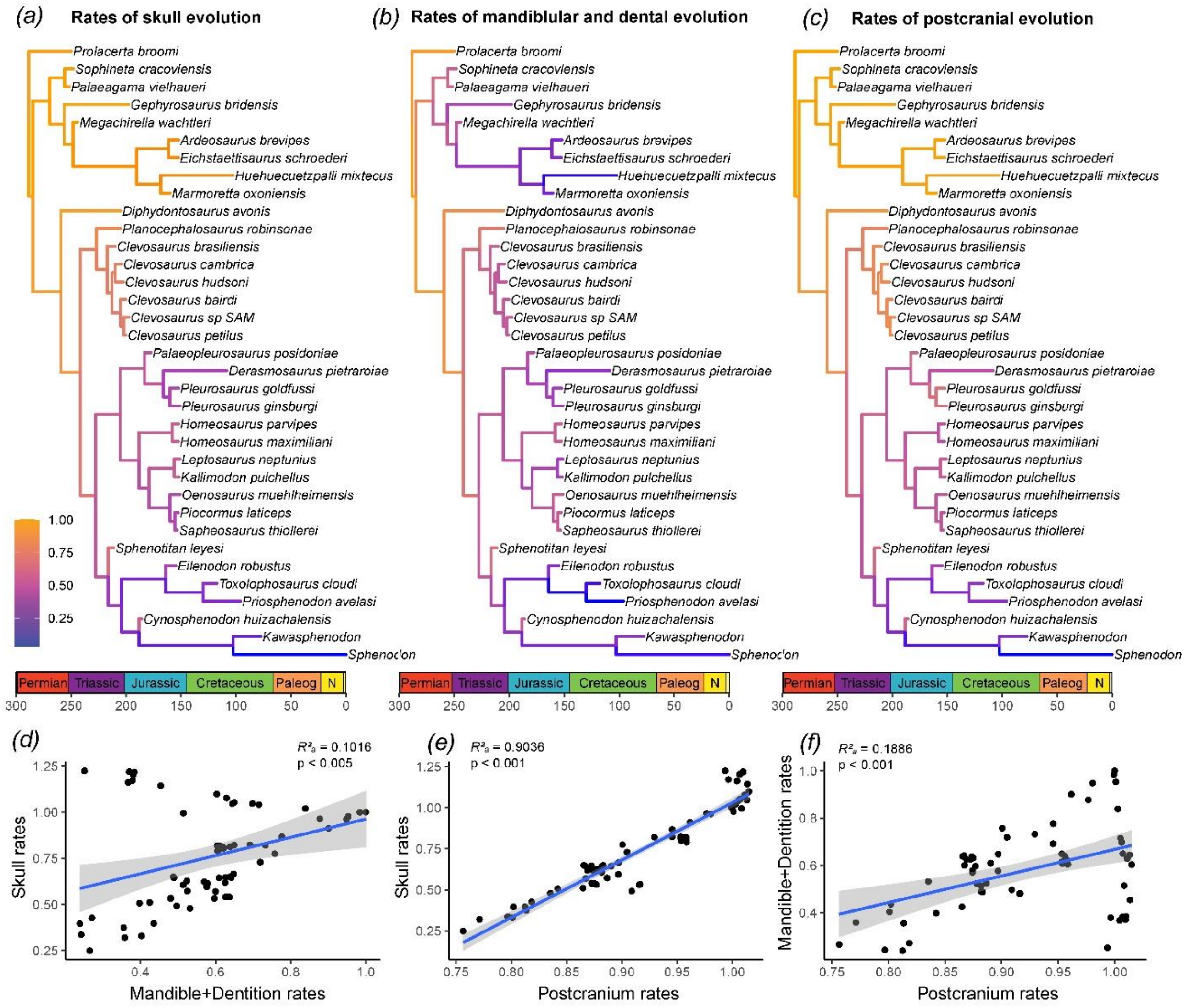

Supplementary Figure 10. Relative rates of morphological evolution for each morphological clock partition on the tree with the best performing tree and clock model combination with partitioned morphological clocks (autocorrelated clock + no sampling of ancestors + maximizing diversity + two-time-slices skyline FBD + truncated normal prior on the root age) and asymmetric state frequencies for the model of character evolution. Branch colors and values indicate relative evolutionary rates. (a) rates of evolution for skull characters. (b) rates of evolution for mandibular and dental characters. (c) rates of evolution for postcranial characters. (d) linear regression between skull and mandibles+ dentition evolutionary rates. (e) linear regression between skull and postcranial evolutionary rates. (f) linear regression between mandible+ dentition and postcranial evolutionary rates.
