# Supplementary material for: Sphenodontian phylogeny and the impact of model choice in Bayesian morphological clock estimates of divergence times and evolutionary rates
Source: BMC Biol. 2020 Dec 7;18:191. doi: 10.1186/s12915-020-00901-5 (PMC7720557; doi:10.1186/s12915-020-00901-5)
Supplement: Supplementary file 2 — Additional file 2. Text document containing list of sampled taxa, along with their occurrence data, stratigraphic interval, age, anatomical bibliography, and personally observed specimen numbers. [file 12915_2020_901_MOESM2_ESM.pdf]

Sampled taxa

Sphenodontian phylogeny and the Impact of Model Choice and Clock Partitioning in Bayesian Morphological Clock Estimates of Divergence Times and Evolutionary Rates

Tiago R. Simões, Michael W. Caldwell and Stephanie E. Pierce

Outgroup

Prolacerta broomi Parrington, 1935

Age. Induan-Olenekian, Early Triassic (Smith *et al.* 2012).

Horizon/Locality. *Lystrosaurus* zone, Beaufort Group, Karoo Supergroup—Harrismith District (Free State Province), Hueningkrans (Burgersdorp District, Eastern Cape Province), Fairydale and Tweefontein (Bethulie District, Free State Province), Rietport (Dewetsdorp District, Free State Province), Big Bank, Queen's Hill and Old Brickfield's Donga (Harrismith District, Free State Province), Barendskraal (Middleburg District, Eastern Cape Province) South Africa [(Gow 1974; Modesto & Sues 2004) and individual specimen labels]; possibly from Fremow Formation—Transantarctic Mountains, Antarctica (Colbert 1987).

Holotype. UMZC 2003.40 (high resolution pictures provided by F. Costa)

Observed referred materials. BPI/1/471, BPI/1/2675, BPI/1/2676, BPI/1/4504a &b, BP/1/5066, BPI/1/5375, BPI/1/5880

Main bibliography. Parrington (1935); Huene (1944); Camp (1945); Broom & Robinson (1948); Young (1948); Robinson (1967); Kuhn (1969); Wild (1973); Kuhn-Schnyder (1974); Gow (1974); Wild (1980); Evans (1984); Evans (1986); Colbert (1987); Modesto & Sues (2004); Müller (2004); Botha-Brink & Smith (2011).

Remarks. We follow here the designation of *Pricea longipes* Broom & Robinson, 1948 as a junior synonym of *Prolacerta broomi* (Gow 1974; Modesto & Sues 2004).

Early lepidosauromorphs

Palaegama vielhaueri Broom, 1926

Age. Middle Lopingian, Late Permian-middle Olenekian, Early Triassic (Smith *et al.* 2012).

Horizon/Locality. Top of *Daptocephalus* zone [= late *Cistecephalus* and *Dicynodon* Zones], or *Lystrosaurus* Zone, Beaufort Group, Karoo Supergroup—Kinira, Mount Frere District, Eastern Cape Province, South Africa (Broom 1926; Carroll 1975).

Holotype. MGM 3707 (observed).

Main bibliography. Broom (1926); Haughton (1929); Kuhn (1969); Carroll (1975); Carroll (1977); Estes (1983); Evans (1984); Carroll (1988); Carroll & Currie (1991).

Remarks. The identification of the squamosal and quadrate by Carroll (1975) are dubious, and thus considered to be only tentative. Additionally, the suture between the lacrimal and prefrontal

is difficult to distinguish and could be merely a crack. Nevertheless, the shape of the postfrontal, postorbital and jugal are considered to be just as provided by Carroll (1975).

Sophineta cracoviensis Evans & Borsuk-Bialynicka, 2009
Age. Early late Olenekian, Early Triassic (Shishkin & Sulej 2009),
Horizon/Locality. Czatkowice 1 Quarry, Kraków Region, Poland (Evans & Borsuk-Białynicka 2009).
Holotype. ZPAL RV/175 (observed)
Observed referred materials. ZPAL RV/10, ZPAL RV/13, ZPAL RV/226, ZPAL RV/227, ZPAL RV/228, ZPAL RV/232, ZPAL RV/233, ZPAL RV/234, ZPAL RV/431, ZPAL RV/236, ZPAL RV/246-247, ZPAL RV/248-249, ZPAL RV/506, ZPAL RV/746, ZPAL RV/747, ZPAL RV/748, ZPAL RV/749, ZPAL RV/974, ZPAL RV/1053, ZPAL RV/1054, ZPAL RV/1061, ZPAL RV/1089, ZPAL RV/1094, ZPAL RV/1101, ZPAL RV/1142, ZPAL RV/1145, ZPAL, ZPAL RV/1584, ZPAL RV/1121.
Main bibliography. Evans & Borsuk-Białynicka (2009)
Remarks. Remarks. The remains of this taxon are composed of isolated bones only. Upon personal observation, it could be detected that most of the skull elements fit into each other, thus demonstrating an anatomical connectivity between the referred specimens. The dental morphology also matches between the lower and upper jaws. However, the absence of anatomical connections between the preserved skull elements and the postcranium make the attribution of the postcranial material previously assigned to *S. cracoviensis* only tentative. For this reason, we chose to be conservative and only include data from the skull remains of this taxon.

Squamata

Megachirella wachtleri Renesto & Posenato, 2003
Age. Pelsonian, Anisian, Middle Triassic (Renesto & Bernardi 2014)
Horizon/Locality. Dont Formation, Braies Group—Monte Prà della Vacca, Braies/Prags Dolomites, Bolzano, Italy (Renesto & Posenato 2003).
Holotype. PZO628 (observed)
Main bibliography. Renesto & Posenato (2003); Renesto & Bernardi (2014), Simões *et al.* (2018).

Marmoretta oxoniensis Evans, 1991
Age. Late Bathonian, Middle Jurassic to Kimmeridgian, Late Jurassic (Evans & Kermack 1994)
Horizon/Locality. Kirtlington Mammal Bed, near the base of Forest Mable Formation (late Bathonian)—Old Cement Works Quarry, Kirtlington, Oxfordshire, United Kingdom; Kilmaluag Formation (Bathonian)—north side of Glen Scaladel (Cladach a'Ghlinne), Isle of Skye, Scotland, United Kingdom; Guimarota lignite mine, Leira, Portugal (Kimmeridgian) (Evans 1991; Evans & Kermack 1994; Waldman & Evans 1994).
Holotype. NHMUK R.12020.

Paratypes. NHMUK R.12025, NHMUK R.12026, NHMUK R.12027, NHMUK R.12028.
Observed referred materials. NHMUK 12400-12406, NMS G 1992.47.1, NMS G 1992.47.4, NMS G 1992.47.5.
Main bibliography. Evans (1991); Evans & Milner (1994); Waldman & Evans (1994); Benton & Spencer (1995); Evans & Waldman (1996).
Remarks. Semi-articulated and associated cranial and postcranial remains of *Marmoretta* from the Bathonian of the Isle of Skye, Scotland, have been used as means of anatomical comparison and attribution of some isolated remains to this taxon.

Huehuecuetzpalli mixtecus Reynoso, 1998
Age. Late Albian, Early Cretaceous (Benammi *et al.* 2006).
Horizon/Locality. Middle portion of the Tlayúa Formation—south of Tepexi de Rodríguez, State of Puebla, Mexico (Reynoso & Cruz 2014a).
Holotype. IGM 7389 (observed).
Paratype. IGM 4185**.**
Main bibliography. Reynoso (1998); Reynoso & Cruz (2014a).
Remarks. Personal observation of the holotype and paratype indicate a few differences to the description provided by Reynoso (1998). Among these, vertebrae on the pelvic region of the holotype are somewhat disarticulated and the anteriormost portion of their neural arches exposed with no clear indication of a zygosphene-zygantra system. On the skull, the left side of the parietal includes an elongate supratemporal process contacting the left squamosal, which is slightly displaced anteriorly. Between the squamosal and the supratemporal process of the parietal, a third distinct element is present, sutured to the parietal and elongate in shape. This corresponds to the supratemporal, which also occupies this same position and has this same overall shape in most squamates bearing those three elements. The supratemporal is less distinct on right side of the skull, but under UV light a partial suture is observed between it and the parietal. This suggests a partial fusion of the right element took place. Additionally, the presence of dorsal intercentra could not be confirmed. The holotype is preserved in dorsal view, preventing an assessment of the later condition, whereas the paratype shows the posterior dorsals in lateral view. Reynoso (1998) mentioned the presence of intercentra in the last presacrals (an inference that might have been made based on the paratype), but no clear structures between the dorsal centra could be established as intercentra. Therefore, this particular feature is treated herein as missing data

Eichstaettisaurus schroederi (Broili, 1938)
Age. Latest Kimmeridgian-early Tithonian, Late Jurassic (Schweigert 2007).
Horizon/Locality. Solnhofen Plattenkalk— Wintershof (near Eichstätt), Bavaria, Germany (Broili 1938).
Holotype. BSPG 1937 I 1 (observed)
Main bibliography. Broili (1938); Young (1948); Hoffstetter (1953); Kuhn (1958); Cocude-Michel (1961); Cocude-Michel (1963b); Hoffstetter (1964); Hoffstetter (1966); Estes (1983);

Evans (1993); Rieppel (1994); Evans *et al.* (2000); Evans *et al.* (2004); Daza *et al.* (2014); Simões *et al.* (2017).

Remarks. For a detailed recent account on the taxonomy, systematics, and re-assessment of the osteology of *E. schroederi* see Simões *et al.* (2017).

Ardeosaurus brevipes (Meyer, 1855)

Age. Latest Kimmeridgian-early Tithonian, Late Jurassic (Schweigert 2007).

Horizon/Locality. Solnhofen Plattenkalk—Eichstätt, Bavaria, Germany (Mateer 1982).

Holotype. Collection Hetzell, now lost (Mateer 1982; Estes 1983). Cast of holotype: NHMUK 38006 (observed).

Observed referred materials. BSPG 1923. I. 501.

Main bibliography. Meyer (1855); Meyer (1860); Zittel (1887); Nopcsa (1908); Camp (1923); Broili (1925); Hoffstetter (1953); Hoffstetter (1955b); Cocude-Michel (1963b); Hoffstetter (1964); Hoffstetter (1966); Estes (1983); Evans (1993); Rieppel (1994); Simões *et al.* (2017); Tałanda (2018).

Remarks. After the loss of the holotype of *A. brevipes*, a new specimen (PMU.R58) was discovered an described by Mateer (1982). However, PMU.R58 is currently lost as well (B. Kear, personal communication). Another specimen, previously assigned as *Ardeosaurus* cf. *digitatellus* (BSPG 1923. I. 501), displays several features that allow its assignment to *A. brevipes*. These include: impressions of skull roof osteoderms, a wider posterior margin of the parietals between the supratemporal processes (compared to the holotype of *A. digitatellus*), separate postorbital and posfrontal, and only three phalanges on the fifth pedal digit. Although preserved mostly as impressions, the level of detail preserved in the soft matrix allows the identification of individual bones and sutures, which highly contribute to increase the amount of information to be scored for this taxon in the present dataset.

Rhynchocephalia

Gephyrosaurus bridensis Evans, 1980

Age. Haettagian or Sinemurian, Lower Jurassic (Evans & Kermack 1994; Evans & Jones 2010; Whiteside *et al.* 2016).

Horizon/Locality. Pontalun and Pant quarries, and St. Bride's Island, South Glamorgan, Wales, United Kingdom (Evans 1980; Evans & Kermack 1994).

Holotype. NHMUK T.1503 (observed)

Observed referred materials. NHMUK T.722, NHMUK T.748, NHMUK T.752, NHMUK T.753, NHMUK T.755, NHMUK T.766, NHMUK T.769, NHMUK T.772, NHMUK T.782, NHMUK T.791, NHMUK T.856, NHMUK T.860, NHMUK T.865, NHMUK T.901, NHMUK T.907-910, NHMUK T.913-917, NHMUK T.937, NHMUK T.938, NHMUK T.940, NHMUK T.945-948, NHMUK T.950, NHMUK T.955-959, NHMUK T.1001, NHMUK T.1015, NHMUK T.1055, NHMUK T.1177, NHMUK T.1238-1264, NHMUK T.1428, NHMUK T.1450,

NHMUK T.1454, NHMUK T.1481, NHMUK T.1509, NHMUK T.1512, NHMUK T.1515, NHMUK T.1522, NHMUK T.1528, NHMUK T.1543, NHMUK T.1553, NHMUK T.1554, NHMUK T.1618, NHMUK T.1633, NHMUK T.1693, NHMUK T.1751, NHMUK T.1814, NHMUK T.1815, NHMUK T.1818-1833, NHMUK T.1845, NHMUK T.1847, NHMUK T.1849, NHMUK T.1853, NHMUK T.1855, NHMUK T.1863-1866, NHMUK T.1874, NHMUK T.1875, NHMUK T.1880, NHMUK T.1881, NHMUK T.1942, NHMUK T.1951, NHMUK T.1993, NHMUK T.1994, NHMUK T.2044, NHMUK T.2046, NHMUK T.2047, NHMUK T.2054, NHMUK T.2055, NHMUK T.2065, NHMUK T.2066, NHMUK T.2070, NHMUK T.2086, NHMUK T.2093, NHMUK T.2098, NHMUK T.2111, NHMUK T.2135, NHMUK T.2143, NHMUK T.2154, NHMUK T.2175, NHMUK T.2196, NHMUK T.2216, NHMUK T.2225, NHMUK T.2227, NHMUK T.2231, NHMUK T.2301, NHMUK T.2311, NHMUK T.2313, NHMUK T.2319, NHMUK T.2320, NHMUK T.2323, NHMUK T.2325, NHMUK T.2335, NHMUK T.2336, NHMUK T.2337, NHMUK T.2346-2352, NHMUK T.2357-2361, NHMUK T.232, NHMUK T.2639-2642, NHMUK T.3017-3022, NHMUK T.3113-3117, NHMUK T.3223

Main bibliography. Evans (1980); Evans (1981); Estes (1983); Evans (1984); Benton (1985); Evans (1985); Fraser & Benton (1989); Evans & Kermack (1994); Borsuk-Białynicka (1996); Wu (2003); Jones (2008); Evans & Jones (2010); Simões *et al.* (2016 [Suppl. Mat.]).

Remarks. See comments for the Late Triassic-Early Jurassic British faunas in the taxonomic sampling criteria above.

Diphydontosaurus avonis Whiteside, 1986

Age. Rhaetian, Late Triassic (Fraser 1994; Evans & Jones 2010).

Horizon/Locality. Tytherington and Cromhall quarries, South Gloucestershire, United Kingdom (Whiteside 1986).

Holotype. BU 23760 (observed).

Paratypes. BU 23763, BU 23764, BU 23842, BU 23787, BU 23789, BU 23785, BU 23781, BU 23790, BU 23780, BU 23782, BU 23783, BU 23784, BU 23772, 23776, BU 23768 BU 23774, BU 23986, BU 23778, BU 23777, BU 23761, BU 23762 (all observed).

Main bibliography. Whiteside (1986); Fraser & Benton (1989); Evans & Kermack (1994); Fraser (1994)**;** Borsuk-Białynicka (1996); Wu (2003); Jones (2008); Evans & Jones (2010); Simões *et al.* (2016 [Suppl. Mat.]).

Remarks. The published and available remains of this taxon are composed of isolated bones only. Upon personal observation, it could be detected that most of the skull elements fit into each other, thus demonstrating an anatomical connectivity between the referred specimens. The dental morphology also matches between the lower and upper jaws. However, the absence of anatomical connections between the preserved skull elements and the postcranium make the attribution of the postcranial material previously assigned to *D. avonis* only tentative. For this reason, we chose to be conservative and only include data from the skull remains of this taxon. We note that unpublished articulated specimens of *Diphydontosaurus* were observed by us at the

NHMUK, but they were not available for study as they are currently being scribed elsewhere (Marc Jones, pers. com.).

Planocephalosaurus robinsonae Fraser, 1982
Age. Rhaetian, Late Triassic (Fraser 1994; Evans & Jones 2010).
Horizon/Locality. Cromhall (old Slickstones) and Tytherington quarries, South Gloucestershire, United Kingdom (Fraser 1982; Evans & Kermack 1994).
Holotype. AUP 11061 (observed).
Observed referred materials AUP 11062-AUP 11081, AUP 11170-AUP 11185, NHMUK R9953-NHMUK R9976.
Main bibliography. Fraser (1982); Fraser & Walkden (1984); Fraser & Benton (1989); Evans & Kermack (1994); Fraser (1994)**;** Wu (2003); Jones (2008); Evans & Jones (2010); Simões *et al.* (2016 [Suppl. Mat.]).
Remarks. The remains of this taxon are composed of isolated bones only. Upon personal observation, it could be detected that most of the skull elements fit into each other, thus demonstrating an anatomical connectivity between the referred specimens. The dental morphology also matches between the lower and upper jaws. However, the absence of anatomical connections between the preserved skull elements and the postcranium make the attribution of the postcranial material previously assigned to *P. robinsonae* only tentative. For this reason, we chose to be conservative and only include data from the skull remains of this taxon.

Clevosaurus hudsoni (Swinton, 1939)
Age. Carnian-Rhaetian, Late Triassic (Fraser 1994; Evans & Jones 2010).
Horizon/Locality. Cromhall (old Slickstones) Quarry, South Gloucestershire, United Kingdom (Fraser 1988); same genus (possibly the same speies) from Tytherington, Emborough, and Pant quarries, southwest England and Wales, United Kingdom (Fraser 1988; Evans & Kermack 1994).
Holotype. NHMUK R5939 (syntypes).
Observed referred materials. NHMUK R604, NHMUK R605 (a,b,c), NHMUK R9249, UMZC T1264, UMZC T1265, UMZC T1266, UMZC T1267, UMZC T1268, UMZC T1269, UMZC T1270, UMZC T1271, UMCZ T 1272, UMCZ T 1273, UMCZ T 1274, UMCZ T 1275, UMCZ T 1276, UMCZ T 1277, UMCZ T 1279.
Main bibliography. Swinton (1939); Robinson (1973); Fraser (1988); Fraser & Benton (1989); Evans & Kermack (1994); Fraser (1994); Wu (2003); Jones (2008); Evans & Jones (2010); Simões *et al.* (2016 [Suppl. Mat.]).
Remarks. The articulated material that includes cranium and postcranium (UMZC T1271) as well as the articulated skulls (NHMUK R604, NHMUK R605, and UMZC T1269) was used as the main basis of comparison to establish which of the isolated bones can be confidentially associated to *Clevosaurus hudsoni*. See also comments for the Late Triassic-Early Jurassic British faunas in the taxonomic sampling criteria above.

Clevosaurus cambrica Keeble et al., 2018
Age. Rhaetian, Late Triassic (Keeble *et al.* 2018).
Horizon/Locality. Pant-y-ffynnon Quarry, near Bonvilston, Vale of Glamorgan, Wales, UK.
Holotype. NHMUK R5939 (syntypes).
Main bibliography. Keeble *et al.* (2018); Chambi-Trowell *et al.* (2019).
Remarks: Data scored from CT-scan data in Keeble *et al.* (2018) and Chambi-Trowell *et al.* (2019).

Clevosaurus brasiliensis Bonaparte and Sues, 2006
Age. Late Norian-Rhaetian, Late Triassic (Hsiou *et al.* 2015)
Horizon/Locality. Caturrita Formation— Linha São Luiz locality, Faxinal do Soturno Municipality, State of Rio Grande do Sul, Brazil (Hsiou *et al.* 2015).
Holotype. UFRGS-PV 0748 T (observed).
Main bibliography. Bonaparte & Sues (2006); Arantes *et al.* (2009); Hsiou *et al.* (2015); Romo-de-Vivar-Martínez & Soares (2015); Romo-de-Vivar-Martínez *et al.* (2017).

Clevosaurus bairdi Sues et al., 1994
Age. Hettangian, Early Jurassic (Sues *et al.* 1994).
Horizon/Locality. McCoy Brook Formation, Fundy Group— Wasson Bluff, near Parrsboro, Cumberland County, Nova Scotia, Canada (Sues *et al.* 1994).
Holotype. NSM 988GFl.1
Observed referred materials. MCZ 9105; MCZ 9106; MCZ 9110; MCZ 9112; MCZ 9113; MCZ 9114.
Main bibliography. Sues *et al.* (1994).

Clevosaurus petilus (Wu, 1994)
Age. Hettangian?, Early Jurassic (Wu 1994; Jones 2006).
Horizon/Locality. Dull Purplish Beds, Lufeng Formation —Yunnan Province, China (Wu 1994; Jones 2006).
Holotype. IVPP V-4007 (observed)
Observed referred materials. IVPP V-8272 (previous holotype of *C. mcgilli*), IVPP V-8273, IVPP V-8272 (previous holotype of *C. wangi*).
Main bibliography. Young (1982); Wu (1994); Sues *et al.* (1994); Jones (2006).
Remarks. Initially described by Young and published as *Dianosaurus petilus*, later redescribed and reclassified by Wu (1994) in the genus *Clevosaurus*. However, we agree with the interpretation of lack of diagnostic features to separate *C. wangi*, *C. mcgilli* and *C. petilus* (Sues *et al.* 1994; Jones 2006), and thus we follow Sues *et al.* (1994) and Hsiou *et al.* (2019) in considering all three Chinese clevosaurs as a single OTU.

***Clevosaurus* sp. SAM**
Age. Hettangian-Pliensbachian, Early Jurassic (Olsen & Galton 1984; Sues & Reisz 1995)

Horizon/Locality. Elliot or Clarens Formation, Stormberg Group—locality unknown (Sues & Reisz 1995).
Observed referred materials. SAM K7890.
Main bibliography. Sues & Reisz (1995).

Clevosaurus convalis
Age. Haettagian or Sinemurian, Lower Jurassic (Evans & Kermack 1994; Evans & Jones 2010; Whiteside *et al.* 2016).
Horizon/Locality. Pant quarry, South Glamorgan, Wales, United Kingdom (Säilä 2005)
Holotype. NHMUK R7530.
Main bibliography. Säilä (2005)

Homeosaurus maximiliani Meyer, 1847
Age. Latest Kimmeridgian-early Tithonian, Late Jurassic (Schweigert 2007)
Horizon/Locality. Solnhofen Plattenkalk—Kelheim and Wintershof, Bavaria, Germany [specimen identification labels and Cocude-Michel (1963a)].
Holotype. lost in 1944 in Munich—see Cocude-Michel (1963a).
Neotype: BSPG 1887 VI 502 (observed).
Observed referred materials. BSPG 1937-1-40.
Main bibliography. Meyer (1847); Wagner (1852); Meyer (1860); Meyer (1866); Boulenger (1891); Broili (1925); Barbour & Stetson (1929); Cocude-Michel (1963a); Kuhn (1969).
Remarks. The genus *Homeosaurus* is in need of revision, therefore only the neotype of *H. maximiliani* and another specimen housed at the BSPG collection (BSPG 1937-1-40.) that could be attributed to the same species are included herein as *H. maximialiani*. Cocude-Michel (1963a) also listed BSPG 1937-1-40 among the referred specimens of *H. maximialiani*. A revision and re-characterization of the genus might reveal additional specimens of *H. maximialiani* that were not studied herein. The localities provided herein are the ones where the specimens referred above come from.

Homeosaurus parvipes Cocude-Michel, 1963
Age. Latest Kimmeridgian-early Tithonian, Late Jurassic (Schweigert 2007).
Horizon/Locality. Solnhofen Plattenkalk—exact locality unknown, Bavaria, Germany (Cocude-Michel 1963a).
Holotype. MB.R.1007 (previously Berlin Rhy 1)
Main bibliography. Cocude-Michel (1963a).

Sapheosaurus thiollierei Meyer, 1850
Age. Latest Kimmeridgian, Late Jurassic (Bernier *et al.* 1984).
Horizon/Locality. Calcaire Lithographique—Cerin, Ain, France (Cocude-Michel 1963a).
Holotype. MNHNL 15672
Observed referred materials. MNHNL 15649, MNHNL 15645, MNHNL 15673.
Main bibliography. Hoffstetter (1955a); Cocude-Michel (1963a).

Remarks. Phylogenetic scoring for this taxon was based on high-resolution pictures (provided by S. Apesteguía) and Cocude-Michel (1963a).

Oenosaurus muehlheimensis Rauhut et al., 2012
Age. Early Tithonian, Late Jurassic (Rauhut *et al.* 2012).
Horizon/Locality. Mörnsheim Formation— Krautworst Naturstein quarry in Mühlheim near Mörnsheim, central Bavaria, Germany (Rauhut *et al.* 2012).
Holotype. BSPG 2009 I 23.
Main bibliography. Rauhut *et al.* (2012).

Piocormus laticeps Wagner, 1852
Age. Latest Kimmeridgian-Early Tithonian, Late Jurassic (Fabre *et al.* 1982; Schwarz-Wings *et al.* 2011).
Horizon/Locality. Tithonian lithographic limestone—Kelheim, Germany (Wagner 1852); Calcaires Blancs—Quarry situated near Aiguines village, Canjuers, Var, France (Fabre *et al.* 1982).
Holotype. Two plates forming part and counterpart described by Wagner (1852), now lost (Cocude-Michel 1963a).
Observed referred materials. MNHN CNJ 68.
Main bibliography. Wagner (1852), Hoffstetter (1955a); Cocude-Michel (1963a); Fabre *et al.* (1982).

Derasmosaurus pietraroiae Barbera & Macuglia, 1988
Age. Albian, Early Cretaceous (Cau *et al.* 2014).
Horizon/Locality. Pietraroia Plattenkalk— Le Cavere Quarry, Pietraroia, Benevento Province, Italy (Barbera & Macuglia 1988; Cau *et al.* 2014)
Holotype. MPN 541
Main bibliography. Barbera & Macuglia (1988); Cau *et al.* (2014).
Remarks. Phylogenetic scoring for this taxon was based on high-resolution pictures (provided by I. Paparella).

Palaeopleurosaurus posidoniae Carroll, 1985b
Age. Toarcian, Early Jurassic (Carroll 1985b).
Horizon/Locality. Posidonienschiefer, Schwarzjura, Lias Epsilon II1and2—P. Kirchmann Quarry, in Staatswald Ohmden, near Holzmaden, Germany (Carroll 1985b)
Holotype. SMN 50722 (observed)
Paratype. SMN 50721 (observed)
Observed referred materials. SMN 81774.
Main bibliography. Carroll (1985b)**;** Carroll & Wild (1994); Dupret (2004)**;** Jones (2008); Evans & Jones (2010).

Pleurosaurus goldfussi Meyer, 1831
Age. Latest Kimmeridgian-early Tithonian, Late Jurassic (Cocude-Michel 1963a; Dupret 2004; Schweigert 2007).
Horizon/Locality. Solnhofen Plattenkalk—Solnhofen, Eichstätt, Monheim, Sappenfeld, Dailing and Wintershof, Bavaria, Germany; Calcaires Blancs—Quarry situated near Aiguines village, Canjuers, Var, France; Calcaire Lithographique—Cerin, Ain, France. (Cocude-Michel 1963a; Reynoso 1996a; Dupret 2004).
Holotype. BSPG 1925 I 18 (observed)
Observed referred materials. BSPG 1978 I 7, MNHN 1983-4-CNJ 80.
Main bibliography. Meyer (1831); Münster (1839); Fitzinger (1843); Wagner (1860); Zittel (1887); Watson (1914); Broili (1926); Huene (1952); Hoffstetter (1955a); Cocude-Michel (1963a); Cocude-Michel (1967); Kuhn (1969); Carroll (1985a); Carroll (1985b); Carroll & Wild (1994); Dupret (2004); Jones (2008); Evans & Jones (2010).
Remarks. Specimen BSPG 1978 I 7 labeled as *P. ginsburgi* is assigned here to *P. goldfussi* based on the skull morphology. The specimen has a fused and relatively elongate postorbitofrontal, followed by an anteroposteriorly short squamosal. This condition is also seen in *P. goldfussi* (BSPG 1925 I 18) and differs from *P. ginsburgi* (BSPG 1977 XIX 40), which has separate postorbital and posfrontals, as well as a comparatively longer squamosal.

Pleurosaurus ginsburgi Fabre, 1974
Age. Lower Tithonian, Late Jurassic (Dupret 2004).
Horizon/Locality. Calcaires Blancs—Quarry situated near Aiguines village, Canjuers, Var, France (Fabre 1974; Dupret 2004)**.**
Holotype. MNHN 1983-4-CNJ 67 (observed).
Observed referred materials. BSPG 1977 XIX 40.
Main bibliography. Fabre (1974); Fabre (1981); Fabre *et al.* (1982); Carroll (1985a); Dupret (2004)**;** Evans & Jones (2010).

Kallimodon pulchellus Zittel, 1887
Age. Latest Kimmeridgian-early Tithonian, Late Jurassic (Schweigert 2007).
Horizon/Locality. Solnhofen Plattenkalk—Kelheim and Kupferberg, Bavaria, Germany [specimen identification labels and Cocude-Michel (1963a)].
Holotype. BSPG 1887 VI 1 (observed).
Observed referred materials. MB.R. 1008.1 (previously Berlin Rhy 2), MB.R. 1009.1-2 (previously Berlin Rhy 3), BSPG 1887 VI 2, BSPG 1922 I 15.
Main bibliography. Zittel (1887); Broili (1925); Cocude-Michel (1959); Cocude-Michel (1963a); Kuhn (1969); Fabre *et al.* (1982); Fraser & Benton (1989).
Remarks. As with *Homeosaurus*, the genus *Kallimodon* also needs revision as numerous specimens attributed to *K. pulchellus* display significant differences to the holotype, mostly based on the postcranium morphology. Additionaly, specimens labeled as belonging to other taxa

show no significant differences to the holotype of *K. pulchellus.* Only studied specimens that show no observable differences to the holotype are included herein as *K. pulchellus,* but a revision and re-characterization of the genus might reveal additional specimens of *K. pulchellus* that were not studied herein. The localities provided herein are the ones where the specimens referred above come from.

Leptosaurus neptunius Goldfuss, 1831
Age. Early Tithonian, Late Jurassic (Dupret 2004).
Horizon/Locality. Calcaires Blancs—Canjuers, Var, France (Cocude-Michel 1963a).
Holotype. "1305 of the collection in Bonn, Germany" (Cocude-Michel 1963a).
Observed referred materials. MNHN CNJ 72.
Remarks. This specimen has numerous similarities with the studied specimens of *Kallimodon pulchellus*. However, one notable difference is the presence of dorsolaterally oriented zygosphenes, observable throughout the dorsal series in this specimen, but absent in all studies specimens of *Kallimodon pulchellus*.

***Opisthias rarus* Gilmore, 1909**
Age. Kimmeridgian, Late Jurassic (Ikejiri 2005).
Horizon/Locality. Lower-middle Upper Morrison Formation—Quarry 9, Como Bluff, Albany County, Wyoming, USA (Gilmore 1909).
Holotype. NMNH 2860
Paratype. NMNH 2858
Observed referred materials. AMNH 14555.
Main bibliography. Gilmore (1909), Simpson (1926)
Remarks. The holotype of *Opisthias* is based on a dentary element only. Thus far, other dentary fragments have been found and associated to this genus, but only two maxillae have been referred to it: AMNH 14551 and YPM 13765. The latter has been described by Simpson (1926) along with new dentary elements. However, it is not mentioned by Simpson whether YPM 13765 was found in articulation or closely associated to the dentary elements. As the maxillary dentition in such taxa is commonly distinct from the dentary dentition, it is not possible to determine with clarity whether this maxilla actually belongs to *Opisthias*. The second maxilla referred to *Opisthias* (AMNH 14551) was found by one of us (TRS) in the AMNH collections. However, we could find no data indicating its potential association with dentary elements. Additionally, it possesses a quite distinct dental anatomy from the other referred maxilla (YPM 13765), possessing hatchling teeth and an alternate tooth series. Therefore, given the lack of clear data concerning those elements and their association with the diagnostic components of the genus (dentaries) and their conflicting anatomies, here we do not score data from the maxilla for this species.

Sphenotitan leyesi Martinez et al., 2013

Age. Norian, Late Triassic (Martínez *et al.* 2013).
Horizon/Locality. Upper layers of the Quebrada del Barro Formation, Marayes–El Carrizal Basin—'Balde de Leyes' fossil locality, San Juan Province, Argentina (Martínez *et al.* 2013).
Holotype. PVSJ 886
Main bibliography. Martínez *et al.* (2013).
Remarks: Taxon scorings based on Martínez *et al.* (2013).

Eilenodon robustus Rasmussen & Callsion, 1981
Age. Kimmeridgian, Late Jurassic (Kowallis *et al.* 1998; Trujillo & Kowallis 2015).
Horizon/Locality. Salt Wash Member, Morrison Formation— Fruita Paleontological Area, Mesa County, Colorado, USA (Rasmussen & Callison 1981).
Holotype. LACM 120462.
Remarks: Taxon scorings based on Rasmussen & Callison (1981).

***Toxolophosaurus cloudi* Olson, 1960**
Age. Aptian-earliest Albian (Decelles 1986; Condon 2000).
Horizon/Locality. Kootenai Formation— Silver Bow County, Southwestern Montana, USA (Olson 1960).
Holotype. FMNH UR619
Main bibliography. Olson (1960); Throckmorton et al. (1981).
Remarks: Taxon scorings based on Olson (1960); Throckmorton et al. (1981).

Priosphenodon avelasi Apesteguia & Novas, 2003.
Age. Cenomanian-Turonian, Late Cretaceous (Apesteguia & Novas 2003).
Horizon/Locality. Candeleros Formation, Neuquén Group—La Buitreta Quarry, Río Negro Province, Argentina (Leanza & Hugo 2001; Apesteguia & Novas 2003).
Holotype. MPCA 300 (observed).
Observed referred materials. MPCA 275, MPCA 293, MPCA 303, MPCA 304, MPCA 305, MPCA 316, MPCA 374.
Main bibliography. Apesteguia & Novas (2003); Simón & Kellner (2003); Jones (2008); Evans & Jones (2010); Apesteguia & Carballido (2014).
Remarks. *P. avelasi* and *Kaikaifilusaurus calvoi* are synonynoum taxa considering the published accounts on both taxa. Although *K. calvoi* has priority due to an earlier publication, this taxon has been considered as a nomen dubium by Apesteguia & Carballido (2014). For an account in the taxonomic dispute between *Kaikaifilusaurus calvoi* and *Priosphenodon avelasi*, see Apesteguia & Carballido (2014).

Cynosphenodon huizachalensis Reynoso, 1996
Age. Pliensbachian, Early Jurassic (Fastovsky *et al.* 2005; Reynoso & Cruz 2014b).
Horizon/Locality. Lower part of La Boca Formation—Huizachal Canyon, Municipio de Ciudad Victoria, Tamaulipas, Mexico (Reynoso 1996b).

Holotype. IGM 6652 (observed).
Observed referred materials. IGM 6653-IGM 6660.
Main bibliography. Reynoso (1996b); Reynoso (2003)**.**

Kawasphenodon Apesteguia, 2005
Age. Campanian-early Maastrichtian (Late Cretaceous) to early Palaeocene (Apesteguía 2005; Apesteguía *et al.* 2014).
Horizon/Locality. Los Alamitos Formation (Late Cretaceous)— Río Negro Province, Argentina; Upper part of the HansenMember of the Salamanca Formation (Palaeocene)—Punta Peligro locality, nearly 27 km north of Comodoro Rivadavia, Chubut province, Argentina (Apesteguía 2005; Apesteguía *et al.* 2014).
Holotype. IGM 7441
Main bibliography. Apesteguía (2005); Apesteguía *et al.* (2014).
Remarks. To reduce the total amount of missing data, we scored the two available species of this genus (*K. expectatus* and *K. peligrensis*) as a single taxonomic operational unit. Scores for this taxon were based on Apesteguía (2005); Apesteguía *et al.* (2014).

Ankylosphenodon pachyostosus **Reynoso, 2000**
Age. Albian (Early Cretaceous) (Reynoso 2000).
Horizon/Locality. Middle member, Tlayua Formation—Colonia Morelos, Tepexi de Rodriguez, Puebla, Mexico (Reynoso 1997; Reynoso 2000).
Holotype. IGM 7441 (observed).
Observed referred materials. IGM 7442, IGM 7443, IGM 7445, IGM 7446, IGM 7447.
Main bibliography. (Reynoso 2000)

Extant taxa

Sphenodon punctatus: MCZ R4702, FMNH 11113, FMNH 197942, FMNH 207433.

Institutional abbreviations:

AMNH – American Museum of Natural History, New York-NY, USA.
AUP – University of Aberdeen Paleontology collection
BSPG - Bayerische Staatssammlung für Paläontologie und historische Geologie, Munich, Germany
BU – University of Bristol, Bristol, UK.
FMNH – Field Museum of Natural History, Chicago, IL, USA.
IGM - Instituto de Geología, Universidad Nacional Autónoma de México
IVPP – Institute of Vertebrate Paleontology and Paleoanthropology, Beijing, China.
LACM : Natural History Museum of Los Angeles County, Los Angeles, California, USA.
MB – Museum fur Naturkunde, Berlin, Germany

MCZ – Museum of Comparative Zoology, Harvard University, Cambridge, MA, USA.
MGM - McGregor Museum – Kimberely, South Africa
MNHN – Muséum National d'Histoire Naturelle, Paris, France.
MNHNL: Museum National de Histoire Naturelle, Lyon.
MPN: Museo Paleontologico, Università di Napoli, Napoli, Italy.
NHMUK – Natural History Museum, London, UK.
NMNH – National Museum of Natural History, Washington, D.C., USA.
NMS – National Museum of Scotland, Edinburgh, United Kingdom
PMU – Paleontological Museum, Uppsala.
PVSJ: Museo de Ciencias Naturales, Universidad Nacional de San Juan, San Juan, Argentina
PZ – Museo Archeologico dell'Alto Adige, Bolzano (Bozen), Italy.
SAM – South Africa Museum (Iziko Museums), Cape Town, South Africa.
SMNS – Staatliches Museum fur Naturkunde, Stuttgart, Germany.
UMZC –Cambridge University Museum of Zoology, Cambridge, UK.
ZPAL - Institute of Paleobiology Polish Academy of Sciences, Warsaw, Poland

References

Apesteguía, S. 2005. A Late Campanian sphenodontid (Reptilia, Diapsida) from northern Patagonia. *Comptes Rendus Palevol*, **4**(8), 663-669.

Apesteguia, S. & Carballido, J. L. 2014. A new eilenodontine (Lepidosauria, Sphenodontidae) from the Lower Cretaceous of central Patagonia. *Journal of Vertebrate Paleontology*, **34**(2), 303-317.

Apesteguía, S., Gómez, R. O. & Rougier, G. W. 2014. The youngest South American rhynchocephalian, a survivor of the K/Pg extinction. *Proceedings of the Royal Society B: Biological Sciences*, **281**(1792), 20140811.

Apesteguia, S. & Novas, F. E. 2003. Large Cretaceous sphenodontian from Patagonia provides insight into lepidosaur evolution in Gondwana. *Nature*, **425**(6958), 609-612.

Arantes, B. d. A., Soares, M. B. & Schultz, C. L. 2009. Clevosaurus brasiliensis (Lepidosauria, Sphenodontia) do Triássico Superior do Rio Grande do Sul: Anatomia pós-craniana e relações filogenéticas. *Revista brasileira de paleontologia. Vol. 12, n. 1 (jan./abr. 2009), p. 43-54.*

Barbera, C. & Macuglia, L. 1988. Revisione dei tetrapodi del Cretacico inferiore di Pietraroia (Matese orientale, Benevento) appartenenti alla collezione Costa del Museo di Paleontologia dell'Università di Napoli. *Memorie della Societa Geologica Italiana*, **41**, 567-574.

Barbour, T. & Stetson, H. C. 1929. The squamation of *Homoeosaurus*. *Bulletin of the Museum of Comparative Zoology*, **69**(4), 97-104.

Benammi, M., Alvarado-Ortega, J. & Urrutia-Fucugauchi, J. 2006. Magnetostratigraphy of the lower cretaceous strata in Tlayúa Quarry, Tepexi de Rodriguez, state of Puebla, Mexico. *Earth, planets and space*, **58**(10), 1295-1302.

Benton, M. J. 1985. Classification and phylogeny of the diapsid reptiles. *Zoological Journal of the Linnean Society*, **84**(2), 97-164.

Benton, M. J. & Spencer, P. S. 1995. *Fossil Reptiles of Great Britain*. Springer Science & Business Media.

Bernier, P., Barale, G., Bourseau, J.-P., Buffetaut, E., Demathieu, G., Gaillard, C., Gall, J.-C. & Wenz, S. 1984. Decouverte de pistes de dinosaures sauteurs dans les calcaires lithographiques de Cerin (Kimmeridgien superieur, Ain, France) implications paleoecologiques. *Geobios*, **17**, 177-187.
Bonaparte, J. F. & Sues, H.-D. 2006. A new species of *Clevosaurus* (Lepidosauria: Rhynchocephalia) from the Upper Triassic of Rio Grande do Sul, Brazil. *Palaeontology*, **49**(4), 917-923.
Borsuk-Białynicka, M. 1996. The Late Cretaceous lizard *Pleurodontagama* and the origin of tooth permanency in Lepidosauria. *Acta Palaeontologica Polonica*, **41**, 231-252.
Botha-Brink, J. & Smith, R. M. H. 2011. Osteohistology of the Triassic archosauromorphs *Prolacerta*, *Proterosuchus*, *Euparkeria*, and *Erythrosuchus* from the Karoo Basin of South Africa. *Journal of Vertebrate Paleontology*, **31**(6), 1238-1254.
Boulenger, G. A. 1891. On British Remains of *Homœosaurus*, with Remarks on the Classification of the Rhynchocephalia. *Proceedings of the Zoological Society of London*, **59**(1), 167-172.
Broili, F. 1925. Beobachtungen an der Gattung *Homeosaurus* H. v. Meyer. *Sitzungsberichte Bayerische Akademie der Wissenschaften zu München Mathematisch-Naturwissenschaftliche Abteilung*, **1925**, 81-121.
Broili, F. 1926. Ueber ein neu entdecktes Exemplar von *Pleurosaurus goldfussi* H. von Meyer aus dem Malm Frankens. *Forschung und Fortschritt*, **2**, 105-107.
Broili, F. 1938. *Ein neuer Fund von ? Ardeosaurus H. v. Meyer*. S-B Bayerische Akademie der Wissenschaften zu München Math.-Naturw. Abt., München.
Broom, R. 1926. On a nearly complete skeleton of a new eosuchian reptile (*Palaeagama vielhaueri*, gen. et sp. nov.). *Proceedings of the Zoological Society of London*, **96**(2), 487-492.
Broom, R. & Robinson, J. T. 1948. Some Now Fossil Reptiles from the Karoo Beds of South Africa. *Proceedings of the Zoological Society of London*, **118**(2), 392-407.
Camp, C. 1923. Classification of the lizards. *Bulletin of the American Museum of Natural History*, **48**, 289-481.
Camp, C. 1945. *Prolacerta* and the protorosaurian reptiles. *American Journal of Science*, **243**(1), 17-32.
Carroll, R. L. 1975. Permo-Triassic 'lizards' from the Karroo. *Palaeontologia Africana*, **18**, 71-87.
Carroll, R. L. 1977. The origin of lizards. Pp. 1-28 in S.M. Andrews, R.S. Miles & A.D. Walker (eds) *Problems in Vertebrate Evolution*. Academic Press, London and New York.
Carroll, R. L. 1985a. Evolutionary constraints in aquatic diapsid reptiles. *Special Papers in Palaeontology*, **33**, 145-155.
Carroll, R. L. 1985b. A pleurosaur from the Lower Jurassic and the taxonomic position of the Sphenodontida. *Palaeontographica Abteilung A*, **189**(1-3), 1-28.
Carroll, R. L. 1988. Late Paleozoic and early Mesozoic lepidosauromorphs and their relation to lizard ancestry. Pp. 99-118 in R. Estes & G. Pregill (eds) *The phylogenetic relationships of the lizard families*. Stanford University Press, Stanford.
Carroll, R. L. & Currie, P. J. 1991. The early radiation of diapsid reptiles. Pp. 354-424 in H.-P. Schultze & L. Trueb (eds) *Origins of the higher groups of tetrapods: controversy and consensus*. Cornstock Publishing Associates, Ithaca and London.

Carroll, R. L. & Wild, R. 1994. Marine members of the Sphenodontia. Pp. 70-83 in N.C. Fraser & H.D. Sues (eds) *In the shadow of the dinosaurs: early Mesozoic tetrapods*. Cambridge University Press, New York.
Cau, A., Baiano, M. A. & Raia, P. 2014. A new sphenodontian (Reptilia, Lepidosauria) from the Lower Cretaceous of Southern Italy and the phylogenetic affinities of the Pietraroia Plattenkalk rhynchocephalians. *Cretaceous Research*, **49**(0), 172-180.
Chambi-Trowell, S. A. V., Whiteside, D. I. & Benton, M. J. 2019. Diversity in rhynchocephalian *Clevosaurus* skulls based on CT reconstruction of two Late Triassic species from Great Britain. *Acta Palaeontologica Polonica*, **64**, 10.4202/app.00569.02018.
Cocude-Michel, M. 1959. Le carpe de *Homeosaurus*, sphenodontide Jurassique. *Bulletin de la Societe Geologique de France*, **7**(3), 230-232.
Cocude-Michel, M. 1961. Les Sauriens des calcaires lithographiques de Bavière, d'âge portlandien inferieur. *Bulletin de la Societe Geologique de France*, **7**(6), 707-710.
Cocude-Michel, M. 1963a. Les Rhynchocephales et les Sauriens des calcaires lithographiques (Jurassique Superieur) d'Europe occidentale. *Nouvelles archives du Muséum d'Histoire Naturelle de Lyon*, **7**, 1-187.
Cocude-Michel, M. 1963b. *Les Rhynchocephales et les Sauriens des calcaires lithographiques (Jurassique Superieur) d'Europe occidentale*. Unpublished PhD thesis, Nouvelles archives du Muséum d'Histoire Naturelle de Lyon, 187 pp.
Cocude-Michel, M. 1967. Revision des Rhynchocephales de la collection du Musee Teyler de Haarlem (Pays-Bas) I. *Proceedings of the Koninklijke Nederlandse Akademie van Wetenschappen. Series B (Physicai Sciences)*, **34**, 538-546.
Colbert, E. H. 1987. The Triassic reptile *Prolacerta* in Antarctica. *American Museum Novitates*, **2882**, 1-19.
Condon, S. M. 2000. *Stratigraphic framework of Lower and Upper Cretaceous rocks in central and eastern Montana*. US Department of the Interior, US Geological Survey.
Daza, J. D., Bauer, A. M. & Snively, E. D. 2014. On the Fossil Record of the Gekkota. *The Anatomical Record*, **297**(3), 433-462.
Decelles, P. G. 1986. Sedimentation in a tectonically partitioned, nonmarine foreland basin: The Lower Cretaceous Kootenai Formation, southwestern Montana. *Geological Society of America Bulletin*, **97**(8), 911-931.
Dupret, V. 2004. The pleurosaurs: anatomy and phylogeny. *Revue de Paléobiologie, Genève,* **9**, 61-80.
Estes, R. 1983. *Handbuch der Paläoherpetologie: Sauria terrestria, Amphisbaenia, part 10A*. Gustav Fischer Verlag, Munich, 249 pp.
Evans, S. E. 1980. The skull of a new eosuchian reptile from the Lower Jurassic of South Wales. *Zoological Journal of the Linnean Society*, **70**(3), 203-264.
Evans, S. E. 1981. The postcranial skeleton of the Lower Jurassic eosuchian *Gephyrosaurus bridensis*. *Zoological Journal of the Linnean Society*, **73**(1), 81-116.
Evans, S. E. 1984. The classification of the Lepidosauria. *Zoological Journal of the Linnean Society*, **82**(1-2), 87-100.
Evans, S. E. 1985. Tooth replacement in the Lower Jurassic lepidosaur *Gephyrosaurus bridensis* *Neues Jahrbuch für Geologie und Paläontologie - Monatshefte*, **7**, 411-420.
Evans, S. E. 1986. The braincase of *Prolacerta broomi* (Reptilia, Triassic). *Neues Jahrbuch für Geologie und Paläontologie - Abhandlungen*, **173**, 181-200.

Evans, S. E. 1991. A new lizard-like reptile (Diapsida: Lepidosauromorpha) from the Middle Jurassic of England. *Zoological Journal of the Linnean Society*, **103**(4), 391-412.
Evans, S. E. 1993. Jurassic lizard assemblages. *Revue de Paléobiologie*, **7**, 55-65.
Evans, S. E. & Borsuk-Białynicka, M. 2009. A small lepidosauromorph reptile from the Early Triassic of Poland. *Palaeontologia Polonica*, **65**, 179-202.
Evans, S. E. & Jones, M. E. H. 2010. The Origin, Early History and Diversification of Lepidosauromorph Reptiles. Pp. 27-44 in S. Bandyopadhyay (ed) *New Aspects of Mesozoic Biodiversity*. Springer Berlin Heidelberg.
Evans, S. E. & Kermack, K. A. 1994. Assemblages of small tetrapods from the Early Jurassic of Britain. Pp. 271-283 in N.C. Fraser & H.-D. Sues (eds) *In the shadow of the dinosaurs: early Mesozoic tetrapods*. Cambridge University Press, New York.
Evans, S. E. & Milner, A. R. 1994. Middle Jurassic microvertebrate assemblages from the British Isles. Pp. 303-321 in N.C. Fraser & H.-D. Sues (eds) *In the shadow of the dinosaurs: early Mesozoic tetrapods*. Cambridge University Press, New York.
Evans, S. E., Raia, P. & Barbera, C. 2004. New lizards and rhynchocephalians from the Lower Cretaceous of southern Italy. *Acta Palaeontologica Polonica*, **49**(3), 393-408.
Evans, S. E., Ruiz, A. L. & Rey, J. 2000. A lizard from the Early Cretaceous (Berriasian-Valanginian) of Montsec, Catalonia, Spain. *Neues Jahrbuch fur Geologie und Palaontologie. Abhandlungen*, **215**(1), 1-15.
Evans, S. E. & Waldman, M. 1996. Small reptiles and amphibians from the Middle Jurassic of Skye, Scotland. *Museum of Northern Arizona Bulletin*, **60**, 219-226.
Fabre, J. 1974. Un squelette de *Pleurosaurus ginsburgi* nov. sp. (Rhynchocephalia) du Portlandien du Petit Plan de Canjuers (Var). *Comptes Rendus de l'Académie des Sciences de Paris, série D*, **278**(19), 2417-2420.
Fabre, J. 1981. *Les Rhynchocéphales et les Ptérosaures à Crête Pariétale du Kimmeridgien supérieur - Berriasien d'Europe Occidentale - Le gisement de Canjuers (Var - France) et ses Abords*. Éditions de la Fondation Singer-Polignac, Paris, 188 pp.
Fabre, J., Broni, F. d., Ginsburg, L. & Wenz, S. 1982. Les vertébrés du Berriasiende Canjuers (Var, France) et leur environnement. *Geobios*, **15**(6), 891-923.
Fastovsky, D. E., Hermes, O. D., Strater, N. H., Bowring, S. A., Clark, J. M., Montellano, M. & Rene, H. R. 2005. Pre–Late Jurassic, fossil-bearing volcanic and sedimentary red beds of Huizachal Canyon, Tamaulipas, Mexico. *Geological Society of America Special Papers*, **393**, 401-426.
Fitzinger, L. 1843. *Systema Reptilium*. Braumüller et Seidel, Vienna, 106 pp.
Fraser, N. C. 1982. A new rhynchocephalian from the British Upper Trias. *Palaeontology*, **25**(4), 709-725.
Fraser, N. C. 1988. The osteology and relationships of *Clevosaurus* (Reptilia: Sphenodontida). *Philosophical Transactions of the Royal Society of London, Series B: Biological Sciences*, **321**(1204), 125-178.
Fraser, N. C. 1994. Assemblages of small tetrapods from British Late Triassic fissure deposits. Pp. 214-226 in N.C. Fraser & H.D. Sues (eds) *In the shadow of the dinosaurs: early Mesozoic tetrapods*. Cambridge University Press, New York.
Fraser, N. C. & Benton, M. J. 1989. The Triassic reptiles *Brachyrhinodon* and *Polysphenodon* and the relationships of the sphenodontids. *Zoological Journal of the Linnean Society*, **96**(4), 413-445.

Fraser, N. C. & Walkden, G. M. 1984. The postcranial skeleton of the Upper Triassic sphenodontid *Planocephalosaurus robinsonae*. *Palaeontology,* **27**(3), 575-595.
Gilmore, C. W. 1909. A new rhynchocephalian reptile from the Jurassic of Wyoming, with notes on the fauna of" Quarry 9.". *Proceedings of the U.S. National Museum,* **37**(1698), 35-42.
Gow, C. E. 1974. *The morphology and relationships of Youngina capensis Broom and Prolacerta broomi Parrington*. Unpublished PhD thesis, University of the Witwatersrand, Johannesburg.
Haughton, S. H. 1929. Notes on the Karroo Reptilia from Madagascar. *Transactions of the Royal Society of South Africa,* **18**(2), 125-136.
Hoffstetter, R. 1953. Les Sauriens anté crétacés. *Bulletin du Museum National d'Histoire Naturelle,* **25**(3), 345-352.
Hoffstetter, R. 1955a. Rhynchocéphales. Pp. 556–576 in J. Piveteau (ed) *Traité de paléontologie*. Masson et Cie, Paris.
Hoffstetter, R. 1955b. Squamates de type moderne. *Traité de paléontologie,* **5**, 606-662.
Hoffstetter, R. 1964. Les Sauria du Jurassique supérieur et specialement les Gekkota de Bavie`re et de Mandchourie. *Senckenberger Biologisches,* **45**, 281-324.
Hoffstetter, R. 1966. A propos des genres *Ardeosaurus* et *Eichstaettisaurus* (Reptilia, Sauria, Gekkonoidea) du Jurassique Supérieur de Franconie. *Bulletin de la Societe Geologique de France,* **7**, 592-595.
Hsiou, A. S., De França, M. A. G. & Ferigolo, J. 2015. New data on the *Clevosaurus* (Sphenodontia: Clevosauridae) from the Upper Triassic of Southern Brazil. *PLoS ONE,* **10**(9), e0137523.
Hsiou, A. S., Nydam, R. L., Simões, T. R., Pretto, F. A., Onary, S., Martinelli, A. G., Liparini, A., Martínez, P. R. R. d. V., Soares, M. B., Schultz, C. L. & Caldwell, M. W. 2019. A new clevosaurid from the Triassic (Carnian) of Brazil and the rise of sphenodontians in Gondwana. *Scientific Reports,* **9**(1), 11821.
Huene, F. v. 1944. Die Zweiteilung des Reptilstammes. *Neues Jahrbuch für Mineralogie, Geologie und Paläontologie,* **88**, 427-440.
Huene, F. v. 1952. Revision der Gattung *Pleurosaurus* auf Grund neuer und alter Funde. *Palaeontographica Abteilung A,* 167-200.
Ikejiri, T. 2005. Distribution and biochronology of Camarasaurus (Dinosauria, Sauropoda) from the Jurassic Morrison Formation of the Rocky Mountain Region. Pp. 367-379*New Mexico Geological Society Field Conference Guidebook*.
Jones, M. E. H. 2006. The Early Jurassic clevosaurs from China (Diapsida: Lepidosauria). Pp. 548-561 in J.D. Harris, S.G. Lucas, J.A. Spielmann, M.G. Lockley, A.R.C. Milne & J.I. Kirkland (eds) *The Triassic-Jurassic terrestrial transition*. New Mexico Museum of Natural History, Albuquerque.
Jones, M. E. H. 2008. Skull shape and feeding strategy in *Sphenodon* and other Rhynchocephalia (Diapsida: Lepidosauria). *Journal of Morphology,* **269**(8), 945-966.
Keeble, E., Whiteside, D. I. & Benton, M. J. 2018. The terrestrial fauna of the Late Triassic Pant-y-ffynnon Quarry fissures, South Wales, UK and a new species of Clevosaurus (Lepidosauria: Rhynchocephalia). *Proceedings of the Geologists' Association,* **129**(2), 99-119.
Kowallis, B. J., Christiansen, E. H., Deino, A. L., Peterson, F., Turner, C. E., Kunk, M. J. & Obradovich, J. D. 1998. The age of the Morrison Formation. *Modern Geology,* **22**(1-4), 235-260.

Kuhn-Schnyder, E. 1974. Die Triasfauna der Tessiner Kalkalpen. *Neujahrsblätter - Naturforschende Gesellschaft in Zürich*, **1974**, 1-119.
Kuhn, O. 1958. Ein neuer Lacertilier aus dem fränkischen Lithographieschiefer. *Neues Jahrbuch für Geologie und Paläontologie, Monatshefte*, **1958**, 380-382.
Kuhn, O. 1969. *Handbuch der Paläoherpetologie-Part 9: Proganosauria, Bolosauria, Placodontia, Araeoscelidia, Trilophosauria, Weigeltisauria, Millerosauria, Rhynchocephalia, Protorosauria*. Verlag Dr. Friedrich Pfeil, Stuttgart.
Leanza, H. A. & Hugo, C. A. 2001. Cretaceous red beds from southern Neuquén Basin (Argentina): age, distribution and stratigraphic discontinuities. *Asociación Paleontológica Argentina, Publicación Especial*, **7**, 117-122.
Martínez, R. N., Apaldetti, C., Colombi, C. E., Praderio, A., Fernandez, E., Malnis, P. S., Correa, G. A., Abelin, D. & Alcober, O. 2013. A new sphenodontian (Lepidosauria: Rhynchocephalia) from the Late Triassic of Argentina and the early origin of the herbivore opisthodontians. *Proceedings of the Royal Society B: Biological Sciences*, **280**(1772), 20132057.
Mateer, N. 1982. Osteology of the Jurassic lizard *Ardeosaurus brevipes* (Meyer). *Palaeontology*, **25**(3), 461-469.
Meyer, H. v. 1831. Neue fossile Reptilien aus der Ordnung der Saurier. *Nova acta physico-medica Academiae Caesareae Leopoldino-Carolinae Naturae Curiosum*, **15**, 171-200.
Meyer, H. v. 1847. *Homoeosaurus maximiliani und Rhamphorhynchus (Pterodactylus) longicaudus: zwei fossile reptilien aus dem kalkschiefer von Solenhofen im Naturalienkabinet seiner baiserlich hoheit des Herzogs Maximilian von Leuchtenberg zu Eichstaedt*. Verlag der S. Schmerber'schen buchhandlung, Frankfurt.
Meyer, H. v. 1855. Briefliche Mitteilung an Prof. Bronn. *Neues Jahrbuch für Mineralogie, Geognosie, Geologie und Petrefaktenkunde*, **1855**, 326-337.
Meyer, H. v. 1860. *Zur Fauna der Vorwelt. Reptilien aus dem lithographischen Schiefer des Jura in Deutschland und Frankreich*. Verlag der S. Schmerbersche Buchhandlung, Frankfurt.
Meyer, H. v. 1866. *Homoeosaurus maximiliani* aus dem lithographischen Schiefer von Kelheim. *Palaeontographica*, **15**(2), 49-65.
Modesto, S. P. & Sues, H.-D. 2004. The skull of the Early Triassic archosauromorph reptile *Prolacerta broomi* and its phylogenetic significance. *Zoological Journal of the Linnean Society*, **140**(3), 335-351.
Müller, J. 2004. The relationships among diapsid reptiles and the influence of taxon selection. Pp. 379-408 in G. Arratia, M.V.H. Wilson & R. Cloutier (eds) *Recent advances in the origin and early radiation of vertebrates*. Verlag Dr. Friedrich Pfeil, Munich.
Münster, G. 1839. Ueber einige Petrefakten-Kunde in den lithographischen Schiefern von Baiern. *Neues Jahrbuch für Mineralogie, Geologie und Paläontologie*, 676-682.
Nopcsa, F. v. 1908. Zur kenntnis der fossilen Eidechsen. *Beiträge zur Paläontologie Österreich-Ungarns und des Orients.*, **21**, 33-62.
Olsen, P. E. & Galton, P. M. 1984. A review of the reptile and amphibian assemblages from the Stormberg of southern Africa, with special emphasis on the footprints and the age of the Stormberg.
Olson, E. C. 1960. A trilophosaurid reptile from the Kootenai Formation (Lower Cretaceous). *Journal of Paleontology*, 551-555.
Parrington, F. R. 1935. XVI.—On *Prolacerta broomi*, gen. et sp. n., and the origin of lizards. *Journal of Natural History Series 10*, **16**(92), 197-205.

Rasmussen, T. E. & Callison, G. 1981. A New Herbivorous Sphenodontid (Rhynchocephalia: Reptilia) from the Jurassic of Colorado. *Journal of Paleontology*, **55**(5), 1109-1116.
Rauhut, O. W., Heyng, A. M., López-Arbarello, A. & Hecker, A. 2012. A new rhynchocephalian from the Late Jurassic of Germany with a dentition that is unique amongst tetrapods. *PLoS ONE*, **7**(10), e46839.
Renesto, S. & Bernardi, M. 2014. Redescription and phylogenetic relationships of *Megachirella wachtleri* Renesto et Posenato, 2003 (Reptilia, Diapsida). *Paläontologische Zeitschrift*, **88**(2), 197-210.
Renesto, S. & Posenato, R. 2003. A new lepidosauromorph reptile from the Middle Triassic of the Dolomites (Northern Italy). *Rivista Italiana di Paleontologia e Stratigrafia*, **109**(3), 463-474.
Reynoso, V.-H. 1996a. *Early Cretaceous lepidosaurs (Reptilia: Diapsida) from Central Mexico and the phylogeny of lepidosauromorphs*. Unpublished PhD thesis, McGill University, 297 pp.
Reynoso, V.-H. 1996b. A Middle Jurassic Sphenodon-like sphenodontian (Diapsida: Lepidosauria) from Huizachal Canyon, Tamaulipas, Mexico. *Journal of Vertebrate Paleontology*, **16**(2), 210-221.
Reynoso, V.-H. 1997. A "Beaded" Sphenodontian (Diapsida: Lepidosauria) from the Early Cretaceous of Central Mexico. *Journal of Vertebrate Paleontology*, **17**(1), 52-59.
Reynoso, V.-H. 1998. *Huehuecuetzpalli mixtecus* gen. et sp. nov: a basal squamate (Reptilia) from the Early Cretaceous of Tepexi de Rodríguez, Central México. *Philosophical Transactions of the Royal Society of London B: Biological Sciences*, **353**(1367), 477-500.
Reynoso, V.-H. 2003. Growth patterns and ontogenetic variation of the teeth and jaws of the Middle Jurassic sphenodontian Cynosphenodon huizachalensis (Reptilia: Rhynchocephalia). *Canadian Journal of Earth Sciences*, **40**(4), 609-619.
Reynoso, V.-H. & Cruz, J. A. 2014a. Mesozoic Lepidosauromorphs of Mexico: a review and discussion of taxonomic assignments. Pp. 44-78 in H.E. Rivera-Sylva, K. Carpenter & E. Frey (eds) *Dinosaurs and Other Reptiles from the Mesozoic of Mexico*. Indiana University Press, Bloomington and Indianapolis.
Reynoso, V. H. 2000. An unusual aquatic sphenodontian (Reptilia: Diapsida) from the Tlayua Formation. *Journal of Paleontology*, **74**(1), 133-148.
Reynoso, V. H. & Cruz, J. A. 2014b. Mesozoic Lepidosauromorphs of Mexico: a review and discussion of taxonomic assignments. Pp. 44-78 in H.E. Rivera-Sylva, K. Carpenter & E. Frey (eds) *Dinosaurs and Other Reptiles from the Mesozoic of Mexico*. Indiana University Press, Bloomington and Indianapolis.
Rieppel, O. 1994. The Lepidosauromorpha: an overview with special emphasis on the Squamata. Pp. 23-37 in N.C. Fraser & H.D. Sues (eds) *In the shadow of the dinosaurs: early Mesozoic tetrapods*. Cambridge University Press, New York.
Robinson, P. L. 1967. The evolution of the Lacertilia. *Colloques Internationaux Centre national de la Recherche Scientifique (CNRS)*, **163**, 395-407.
Robinson, P. L. 1973. A problematic reptile from the British Upper Trias. *Journal of the Geological Society*, **129**(5), 457-479.
Romo-de-Vivar-Martínez, P. R., Martinelli, A. G., Paes Neto, V. D. & Soares, M. B. 2017. Evidence of osteomyelitis in the dentary of the late Triassic rhynchocephalian Clevosaurus brasiliensis (Lepidosauria: Rhynchocephalia) from southern Brazil and behavioural implications. *Historical Biology*, **29**(3), 320-327.

Romo-de-Vivar-Martínez, P. R. & Soares, M. B. 2015. Dentary Morphological Variation in *Clevosaurus brasiliensis* (Rhynchocephalia, Clevosauridae) from the Upper Triassic of Rio Grande do Sul, Brazil. *PLoS ONE*, **10**(3), e0119307.
Säilä, L. K. 2005. A new species of the sphenodontian reptile *Clevosaurus* from the Lower Jurassic of South Wales. *Palaeontology*, **48**(4), 817-831.
Schwarz-Wings, D., Klein, N., Neumann, C. & Resch, U. 2011. A new partial skeleton of Alligatorellus (Crocodyliformes) associated with echinoids from the Late Jurassic (Tithonian) lithographic limestone of Kelheim, S-Germany. *Fossil Record*, **14**(2), 195-205.
Schweigert, G. 2007. Ammonite biostratigraphy as a tool for dating Upper Jurassic lithographic limestones from South Germany–first results and open questions. *Neues Jahrbuch für Geologie und Paläontologie-Abhandlungen*, **245**(1), 117-125.
Shishkin, M. A. & Sulej, T. 2009. The Early Triassic temnospondyls of the Czatkowice 1 tetrapod assemblage. *Palaeontologia Polonica*, **65**(3), 77.
Simões, T. R., Caldwell, M. W., Nydam, R. L. & Jiménez-Huidobro, P. 2017. Osteology, phylogeny, and functional morphology of two Jurassic lizard species and the early evolution of scansoriality in geckoes. *Zoological Journal of the Linnean Society*, **180**(1), 216-241.
Simões, T. R., Caldwell, M. W., Tałanda, M., Bernardi, M., Palci, A., Vernygora, O., Bernardini, F., Mancini, L. & Nydam, R. L. 2018. The origin of squamates revealed by a Middle Triassic lizard from the Italian Alps. *Nature*, **557**(7707), 706-709.
Simões, T. R., Funston, G. F., Vafaeian, B., Nydam, R. L., Doschak, M. R. & Caldwell, M. W. 2016. Reacquisition of the lower temporal bar in sexually dimorphic fossil lizards provides a rare case of convergent evolution. *Scientific Reports*, **6**, 24087.
Simón, M. E. & Kellner, A. W. A. 2003. New Sphenodontid (Lepidosauria, Rhynchocephalia, Eilenodontinae) from the Candeleros Formation, Cenomanian of Patagonia, Argentina:(with 12 Figures). *Boletim do Museu Nacional, Nova série, Geologia*, **68**, 1-12.
Simpson, G. G. 1926. American terrestrial Rhynchocephalia. *American Journal of Science*, **67**, 12-16.
Smith, R., Rubidge, B., Van der Walt, M. & Chinsamy-Turan, A. 2012. Therapsid biodiversity patterns and paleoenvironments of the Karoo Basin, South Africa. Pp. 30-62*Forerunners of Mammals: Radiation, Histology, Biology*. Indiana University Press, Indianapolis.
Sues, H.-D. & Reisz, R. R. 1995. First record of the early Mesozoic sphenodontian *Clevosaurus* (Lepidosauria: Rhynchocephalia) from the Southern Hemisphere. *Journal of Paleontology*, **69**(1), 123-126.
Sues, H.-D., Shubin, N. H. & Olsen, P. E. 1994. A new sphenodontian (Lepidosauria: Rhynchocephalia) from the McCoy Brook Formation (Lower Jurassic) of Nova Scotia, Canada. *Journal of Vertebrate Paleontology*, **14**(3), 327-340.
Swinton, W. E. 1939. A new Triassic Rhynchocephalian from Gloucestershire. *Journal of Natural History*, **4**(24), 591-594.
Tałanda, M. 2018. An exceptionally preserved Jurassic skink suggests lizard diversification preceded fragmentation of Pangaea. *Palaeontology*.
Throckmorton, G. S., Hopson, J. A. & Parks, P. 1981. A Redescription of *Toxolophosaurus cloudi* Olson, a Lower Cretaceous Herbivorous Sphenodontid Reptile. *Journal of Paleontology*, **55**(3), 586-597.
Trujillo, K. & Kowallis, B. 2015. Recalibrated legacy 40Ar/39Ar ages for the Upper Jurassic Morrison Formation, Western Interior, USA. *Geology of the Intermountain West*, **2**, 1-8.

Wagner, A. 1852. Neu-aufgefundene Saurier, Überreste aus dem lithographischen Schiefern und dem obern Jurakalke. *Abhandlungen Bayerische Akademie der Wissenschaften zu München Mathematisch-Physikalischen Klasse*, **3**(6), 661-710.
Wagner, J. A. 1860. Vergleichung der urweltlichen Fauna des lithographischen Schiefers von Cirin mit den gleichnamigen Ablagerungen im Frankischen Jura. *Gelehrten Anzeiger der königlich bayerischen Akademie der Wissenschaften*, **48**, 390-391.
Waldman, M. & Evans, S. E. 1994. Lepidosauromorph reptiles from the Middle Jurassic of Skye. *Zoological Journal of the Linnean Society*, **112**, 135-150.
Watson, D. M. S. 1914. *Pleurosaurus* and the homologies of the bones of the temporal region of the lizard's skull. *Annals and Magazine of Natural History*, **14**(79), 84-95.
Whiteside, D. I. 1986. The head skeleton of the Rhaetian sphenodontid *Diphydontosaurus avonis* gen. et sp. nov. and the modernizing of a living fossil. *Philosophical Transactions of the Royal Society of London, Series B: Biological Sciences*, **312**(1156), 379-430.
Whiteside, D. I., Duffin, C. J., Gill, P. G., Marshall, J. E. & Benton, M. J. 2016. The Late Triassic and Early Jurassic fissure faunas from Bristol and South Wales: stratigraphy and setting. *Palaeontologia Polonica*, **67**, 257-287.
Wild, R. 1973. Die Triasfauna der Tessiner Kalkalpen XXIII *Tanystropheus longobardicus* (Bassani)(Neue Ergebnisse). *Schweizerische Paläontologische Abhandlungen*, **95**, 1-162.
Wild, R. 1980. Die Triasfauna der Tessiner Kalkalpen. XXIV. Neue Funde von *Tanystropheus* (Reptilia, Squamata). *Schweizerischen Paläontologischen Abhandlungen*, **102**, 1-31.
Wu, X.-C. 1994. Late Triassic-Early Jurassic sphenodontians from China and the phylogeny of the Sphenodontia. Pp. 38-69 in N.C. Fraser & H.D. Sues (eds) *In the shadow of the dinosaurs: early Mesozoic tetrapods*. Cambridge University Press, Cambridge
Wu, X.-C. 2003. Functional morphology of the temporal region in the Rhynchocephalia. *Canadian Journal of Earth Sciences*, **40**(4), 589-607.
Young, C. C. 1948. A review of Lepidosauria from China. *American Journal of Science*, **246**(11), 711-719.
Young, C. C. 1982. A new fossil reptile from the Lufeng Basin. *Science Press*, 36-37.
Zittel, K. 1887. Handbuch der Palaeontologie I. Abtheilung, Palaeozoologie III. Band., Pisces, Amphibia, Reptilia, Aves. Druck und Verlag Von R. Oldenbourg.
