# Supplementary material for: Sphenodontian phylogeny and the impact of model choice in Bayesian morphological clock estimates of divergence times and evolutionary rates
Source: BMC Biol. 2020 Dec 7;18:191. doi: 10.1186/s12915-020-00901-5 (PMC7720557; doi:10.1186/s12915-020-00901-5)
Supplement: Supplementary file 3 — Additional file 3. Text document including phylogenetic morphological characters list with individual character descriptions. [file 12915_2020_901_MOESM3_ESM.pdf]

Character list

Sphenodontian phylogeny and the Impact of Model Choice and Clock Partitioning in Bayesian Morphological Clock Estimates of Divergence Times and Evolutionary Rates

Tiago R. Simões, Michael W. Caldwell and Stephanie E. Pierce

Abbreviations: A14 (Apesteguia & Carballido 2014); AGR12 (Apesteguía *et al.* 2012); AN03 (Apesteguia & Novas 2003); B85 (Benton 1985); CoN06 (Conrad & Norell 2006); Co08 (Conrad 2008); DBC93 (de Braga & Carroll 1993); DBR97 (de Braga & Rieppel 1997); E88 (Estes *et al.* 1988); Ev88 (Evans 1988); FB89 (Fraser & Benton 1989); G88a (Gauthier *et al.* 1988a); G88b (Gauthier *et al.* 1988b); G12 (Gauthier *et al.* 2012); J94 (Juul 1994); LC00 (Lee & Caldwell 2000); Lee97 (Lee 1997); Lee98 (Lee 1998); Lo12 (Longrich *et al.* 2012); LR95 (Laurin & Reisz 1995); Mo99 (Motani 1999); MS04 (Modesto & Sues 2004); P86 (Pregill *et al.* 1986); R94 (Rieppel 1994); RD03 (Reisz & Dilkes 2003); Re96 (Reynoso 1996); RZ00 (Rieppel & Zaher 2000); S18 (Simões *et al.* 2018); Su94 (Sues *et al.* 1994); W94 (Wu 1994).

Cranium

1. Premaxillae, fusion: unfused (0)/ fused (1) (B85, Ch. Y1).

2. Premaxillae, posterodorsal process: absent (0)/ present (1) (Ev88, Ch. G1—modified).

3. Maxilla, lateral view, premaxillary process: present (0); absent (1) (Su94, W94, Re96, AN03—modified)

4. Maxilla, shape of posterior end: tapering posteriorly (0); dorsoventrally broad (1) (W94, Re96, AN03, A14, Ch. 8).

5. Nasals, foramina: absent (0)/ present (1) (S18, Ch. 28)

6. Nasals, ventrolateral process: absent (0)/ present (1) (G12, Ch. 22, Fig. Ch. 22 therein).

7. Nasals, anterior margin, anteromedial process: absent (0)/ present (1) (NEW).
Remarks: This process extends anteriorly and in parallel to the nasal process of the premaxilla. Its development contributes to an anteriorly concave margin of the nasal as observed in *Homeosaurus maximiliani* and *Kallimodon pulchellus*.

8. Lacrimal: present (0); absent (1) (E88, Ch. 28).

9. Lacrimal duct, posterior opening on skull surface, position: posteriorly (0)/ laterally (1) (RD03, Ch. 19—modified).

10. Jugal, posteroventral process: absent (0); present (1) (Su94, W94, Re96, AN03—modified herein).
Remarks: Previous states relating to the length of the posteroventral process of the jugal have been lumped into a single "present" state herein. This jugal process is quite variable intraspecifically, especially due to ontogeny in a variety of lepidosaurs, such as in the early rhynchocephalians *Planocephalosaurus*, *Clevosaurus*, and *Diphyodontosaurus* (Fraser 1982; Whiteside 1986; Fraser 1988).

11. Jugal, posteroventral process, orientation: directed posteriorly (0); directed laterally (1) (NEW).
Remarks: The posteroventral process of the jugal forms the majority or all of the lower temporal bar in rhynchocephalians. Whereas in some taxa this process is directed posteriorly and relatively straight as compared to the maxilla, in other taxa (e.g. *Sphenodon*) it is directed laterally, creating the larger lower temporal fenestra and more space for the adductor chamber. This character can only be assessed on articulated specimens.

12. Prefrontal crest: absent (0)/ present (1) (G12, Ch. 130).

13. Prefrontal, ornamentation on bone surface: absent (0)/ tubercles (1) (AN03, A14, Ch. 11—modified)

14. Postorbitals, fusion to postfrontal: unfused (0)/ fused (1) (E88, Ch. 14).

15. Postorbital, deep ventrolateral concavity: absent (0); present (1) (AN03, A14, Ch. 13—modified).

16. Postorbitals, dorsal margin, position relative to postfrontal: laterally (0)/ posteriorly (1)/ anteriorly (2) (S18, Ch. 45).

17. Postfrontals, medial forking: absent (0)/ present (1) (E88, Ch. 13)

18. Postfrontals, parietal process: absent (0)/ present (1) (E88, Ch. 13—modified).
Remarks: When the postfrontal parietal process is absent, the postfrontal acquires a lunate-like appearance.

19. Postfrontals, medial margin, position, relative to parietal: ventral (0)/ dorsal (1)/ lateral (2)/ anterior (3) (G12, Ch. 65—modified).
Remarks: The conservative relationship of the contact between the parietal and postfrontal across a wide range of clades and sampled taxa justifies the codification of the position of the postfrontal using the parietal as a landmark.

20. Squamosals, anterior process, lateral surface, facet for postorbital: absent (0)/ parabolic (1)/ half-parabolic (S18, Ch. 61—modified).
Remarks: Absent refers to a smooth lateral surface of the squamosal. A parabolic shape facet for the postorbital creates an elongate lateral concavity into which the posterior process of the postorbital inserts. When both the squamosal and postorbital are in articulation in state “1”, it creates the impression that the anterior process of the squamosal is anteriorly bifid. In state “2”, the facet has a half-parabolic shape for the posterior process of the postorbital, located on the dorsolateral margin of the anterior process of the squamosal, as seen in *Clevosaurus hudsoni*.

21. Squamosals, dorsal process: absent (0)/ present (1) (E88, Ch. 34—modified).

22. Squamosals, anteroventral process: absent (0)/ present (1) (S18, Ch. 50).
Remarks: The squamosal of diapsids can be tetraradiate in many early forms and rhynchocephalians (Benton 1985). One of the processes creating the tetraradiate condition is the anteroventrally directed process, which braces the quadrate (and the quadratojugal, when present) anteriorly, as seen in most rhynchocephalians.

23. Supratemporals: absent (0)/ present (1) (B85, Ch. J3).

24. Quadratojugals: present (0)/ absent (1)/ (G88a, Ch. 12).

25. Quadratojugals, anterior extension: present (0)/ absent (1) (G88a, Ch. 9)
Remarks: In lepidosaurs, when the quadratojugal is present, the main body of the quadratojugal contacts the quadrate, but its anterior extension (such as the one observed in many archosauriforms) is absent.

26. Frontals, fusion to each other: separated (0); fused (1) (B85, Ch. Y1).

27. Frontals, parietal tabs: absent (0)/ present (1) (E88, Ch. 10).
Remarks: Among some early diapsids and rhynchocephalians, each frontal possesses a process overlying the parietal laterally.

28. Parietals, fusion to each other: separated (0)/ fused (1) (B85, Ch. Y1).

29. Parietals, nuchal fossa: absent (0)/ present (1) (G12, Ch. 94—modified, Fig. Ch. 94 therein).
Remarks: The parietal nuchal fossa is a thin bony plate projecting posteriorly from the parietal posterior margin and is distinct from the main body of the parietal.

30. Parietals, posteromedial (= postparietal) process: absent (0)/ present (1) (G12, Ch. 95, Fig. Ch. 95 therein).

31. Parietals, parietal table, shape: margins ventrally directed, sagittal crest present (0)/ margins ventrally directed, without sagittal crest (1)/ margins laterally directed (2) (Lee98, Ch. 35 and G88b, Ch.19).

32. Parietal, posterior margin, shape: curved anteriorly (0)/ curved posteriorly (1)/ straight (2) (W94, Re96, AN03, A14, Ch. 18—re-phrased).

Palatoquadrate

33. Quadrates, posterior emargination: absent (0)/ present (1) (B85, Ch. B6).

34. Quadrates, quadrate conch: absent (0)/ present (1) (B85, Ch. Y11).
Remarks: The tympanic membrane attachment to the quadratojugal (or the quadrate when the quadratojugal is lost) creates a reinforced attachment site (the tympanic crest) that always occurs in conjunction with a thinner and medially projecting bony flange that forms by the quadrate conch. Therefore, the absence/presence of a quadrate conch, and the absence/presence of a tympanic crest, are dependent characters among the studied taxa herein, and the quadrate conch is scored in this dataset to avoid redundancy. Another important consideration is that the tympanic crest of most squamates is positioned on the lateral margin of the quadrate, whereas in most rhynchocephalians this crest actually belongs to the fused quadratojugal. This topological difference seems to be a consequence of the loss of the quadratojugal in squamates, transferring the attachment site of the tympanum from the quadratojugal to the quadrate. Therefore, this topological difference regarding the composition of the tympanic crest is dependent upon the loss of the quadratojugal itself (already considered under character 24), and thus it is not a separate and independent character. Accordingly, we do not treat the composition of the tympanic crest as a distinct character in order to avoid redundancy.

35. Quadrates, suprastapedial process: absent (0)/ present (1) (DBC93, Ch. 40).

36. Quadrate foramen: present (0)/ absent (1) (G88a, Ch. 21).

Remarks: The quadrate foramen is considered to be only the small foramen piercing the quadrate bone itself, usually near the base of the quadrate posterior pillar. This is not to be confounded with the opening between the quadrate and the quadratojugal (Simões *et al.* 2018)—see also character: quadratojugal foramen.

37. Quadrates, cephalic condyle, notch for the squamosal: absent (0)/ present (1) (Ev88, Ch. L9—modified).

Palate

38. Vomers, ventral surface, midline crest (=longitudinal ridge): absent (0)/ present (1) (G12, Ch. 222—modified; Fig. in Ch. 222 therein).
Remarks: The right and left crests lie adjacent to, and butt against, each other on the midline, sometimes diverging anteriorly.

39. Palatine, shape of posterior end: tapers posteriorly (0); widens posteriorly (1) (Su94, W94, Re96, AN03, A14, Ch. 22).

40. Pterygoids, arcuate flange: absent (0)/ present (1) (LR95, Ch. 42).

41. Pterygoids, transverse processes, flange: absent (0)/ present (1) (G12, Ch. 266, Fig. in Ch. 266 therein)
Remarks: The acquisition of the pterygoid flange on its transverse process has been considered as one of the distinguishing feature of reptiles (Carroll 1969).

42. Ectopterygoids, lateral process: absent (0)/ present (1) (G12, Ch. 283—modified, Fig. in Ch. 283 therein).

Braincase

43. Supraoccipital, medial ascending process: absent (0)/ present (1) (RZ00, Ch. 232; Fig. in G12, Ch. 297)
Remarks: Usually referred to as the processus ascendens of the synotic tectum in squamates (Oelrich 1956), this character reflects the ossification of this medially placed and dorsally ascending process that, in many squamates, contacts the parietal ventrally.

44. Supraoccipital, sagittal crest: absent (0)/ present (1) (LR95, Ch. 55).

45. Basioccipital, ventral aspect, shape, concavity: single (0)/ divided (1)/ absent (2) (S18, Ch. 134).
Remarks: This concavity for axial musculature can be either single or divided. Since both conditions are seen as distinct from each other as they are from the absent condition (not hierarchically nested and being mutually exclusive) they are all included as part of the same character instead of being split into contingent characters.

46. Basisphenoid (or fused parabasisphenoid), ventral aspect, shape, concavity: single (0)/ divided (1)/ absent (2) (LR95, Ch. 50).
Remarks: As above.

47. Prootics, alar crest: absent (0)/ present (1) (E88, Ch. 49—modified; Fig. in G12, Ch. 305).
Remarks: The alar crest is a thin bony flange projecting anteriorly on prootics, located just anterior to the anterior semicircular canals (Oelrich 1956; Rieppel 1984). The alar crest is absent in most iguanians, but it is common among other squamate lineages.

Mandibles

48. Dentary, symphysis, ventral margin, mentonian process: absent (0); present (1) (AN03, A14, Ch. 33-modified herein).
Remarks: The mental (or mentonian) process of the dentary is a ventral expansion of the symphysial regions, which is observed within most rhynchocephalians (Apesteguía 2008).

49. Dentary symphysis, symphysial spur: absent (0); present (1) (AN03, A14, Ch. 36-modified herein).
Remarks: The symphysial spur is an edentulous anteriorly directed projection of the dentary (Apesteguía 2008). This character has been transformed to code for the absence and presence of the symphysial spur by combining the states that tried to account for the length of the spur into the present state. Without direct measurements to account for variations in length and the continuous nature of the spur length, the scoring of the spur length becomes subjective and arbitrary. The spur length is also quite prone to ontogenetic variation.

50. Dentary, symphysis, shape: circular (0); eliptical (1) (B85, Re96, AN03—re-phrased herein)

51. Dentaries, anterior end, symphysial articulatory facet, position: on dorsal margin only (0)/ on dorsal and ventral margins (1)/ on ventral margin only (2) (Lo12, Ch. 612).

52. Dentaries, anterior end, split by Meckelian canal: absent (0)/ present (1) (S18, Ch. 167).

53. Dentaries, subdental shelf: present (0)/ absent (1) (E88, Ch. 59).

54. Dentaries, dorsal margin, contact, with ventral margin in medial view: absent (0)/ present (1) (E88, Ch. 55—modified).
Remarks: The degree of contact between both margins along the length of the dentary is a variable with a continuous range of variation that we do not attempt to code herein. Only the absence/presence of that contact is considered for this character. Because the contact between both margins results mostly from a ventral expansion of the subdental crest of the dentary in all of the observed taxa, we consider this feature to be primarily homologous among the sampled species.

55. Dentaries, coronoid process, dorsal expansion: absent (0)/ present (1) (E88, Ch. 60—modified).Remarks: A dorsal expansion of the coronoid process is observed within lepidosaurs, butting against the lateral margin of the dorsal process of the coronoid bone (e,g, sphenodontians).

56. Dentaries, posteroventral process: absent (0)/ present (1) (G88a, Ch. 66—modified).
Remarks: In most reptiles, only the coronoid (posterodorsal) process of the dentary is present. In lepidosaurs (mostly squamates), a posteroventral process occurs (as in *Huehuecuetzpalli*, acrodontans, xantusiids, among others) below the level of the anterior surangular foramen.

57. Splenials: absent (0)/ present (1) (B85, Ch. Z12).

58. Surangulars, posterior surangular foramen: absent (0)/ present (1) (MS04, Ch.146).

59. Surangulars, coronoid process: absent (0)/ present (1) (G88a, Ch. 69, Fig. in G12, Ch. 400).
Remarks: The surangular may have a dorsal contribution to the coronoid eminence of the mandible, with variable degrees of contribution of the coronoid dorsal process.

60. Articular, glenoid cavity, central ridge: absent (0); present (1) (AN03 A14, Ch. 38—recoded).
Remarks: A central ridge on the glenoid articulatory facet of the articular bone fits into a ventral groove or concavity between the condyles of the quadrate-quadratojugal complex and is usually inferred as an adaptation for proal motion of the lower jaw.

61. Articulars, retroarticular process: absent (0)/ present (1) (G88b, Ch. 104).

62. Articular, retroarticular process, orientation: caudally directed (0); dorsally directed (1) (Su94, W94, Re96, AN03, A14, Ch. 40—modified).

63. Coronoids, dorsal process: absent (0)/ present (1) (B85, Ch. J7—modified, Fig. in G12, Ch. 386).

64. Coronoids, anterolateral (=labial) process: absent (0)/ present (1) (E88, Ch. 68—modified).

65. Coronoids, anteromedial process: present (0)/ absent (1) (LC00, Ch. 138, Fig. in G12, Ch. 391).
Remarks: Variable occurrence within rhynchocephalians. Some taxa still possess a distinct anteromedial process projecting ventrally to the dentigerous portion of the posterior end of the dentary, whereas other taxa retain only the dorsal process of the coronoid bone (medially to the coronoid process of the dentary).

66. Coronoids, posterodorsomedial process: present (0)/ absent (1) (NEW).
Remarks: Present in some squamates and early lepidosaurs, but absent in most rhynchocephalians.

67. Coronoid, posteroventromedial process: present (0)/ absent (1) (G12, Ch. 393, Fig. in Ch. 393 therein).
Remarks: Present in some squamates and early lepidosaurs, but absent in most rhynchocephalians.

Dentition

68. Vomers, teeth: absent (0)/ present (1) (G88b, Ch. 120).

69. Palatine teeth, number of tooth rows: one (0); two (1); three (2) (Su94, W94, Re96, AN03, A14, Ch. 52—Recoded).

70. Palatine teeth, longitudinal fusion into a single dentigerous element: unfused (0)/ fused (1) (NEW).
Remarks: In some taxa, all palatine teeth within a single row become fused into a single dentigerous longitudinally elongate structure, such as observed in *Kallimodon*.

71. Palatine teeth, lateral flanges: absent (0); present (1) (FB89, Re96, AN03— modified).
Remarks: Observed among opisthodontids, such as *Sphenotitan* and *Priosphenodon*.

72. Pterygoids, teeth: absent (0)/ present (1) (B85, Ch. L3 and E88, Ch. 83—modified).

73. Pterygoid teeth, number of tooth rows: one (0)/ two (1)/ three (2)/ four (3) (Su94, W94, Re96, AN03, A14, Ch. 55).

74. Marginal dentition, posterior teeth series, tooth replacement: present (0); absent (1) (P86, Ch. 26—modified).

75. Marginal dentition, hatchling teeth in adults: absent (0); present (1) (B85, Re96, AN03 A14, Ch. 44—recoded).

76. Marginal dentition, alternating teeth series: absent (0)/ present (1) (S18, Ch. 218).
Remarks: In *Sphenodon* there are five generations of teeth, the first three occurring in the embryo. The fourth and fifth generations occur after hatching and are termed successional teeth (or replacement teeth). Each of these successional teeth replace two or more of the teeth from the preceding generation (Harrison 1901). Some of the hatchling dentition is retained with the larger successional teeth on the maxilla, creating a pattern of alternating teeth anteriorly. This alternating tooth pattern is also observed in adults of most fossil sphenodontians. This character is inapplicable when hatchling teeth are absent.

77. Marginal dentition, longitudinal fusion into a single dentigerous element: unfused (0)/ fused (1) (NEW).
Remarks: All marginal teeth become fused into a single dentigerous longitudinally elongate structure, such as is observed in *Oenosaurus* and *Saphaeosaurus*.

78. Marginal dentition, posterior teeth series, position, placement relative to jaw bone apical margin (= crista dorsalis): lingually (0); apicolingually (1); apically (2) (S18, Ch. 210—re-phrased).
Remarks: The classical categories of "tooth attachment", such as acrodonty, pleurodonty and thecodonty usually mix a combination of distinct features, such as tooth position on the jaws, ankylosis, and mode of replacement. However, tooth ankylosis to its surrounding tissue of attachment may occur in reptiles in combination with different kinds of tooth topologies and replacement modes. For instance, teeth set in four-sided sockets may or may not be ankylosed to alveolar bone, suggesting both are independent characters. Therefore, here we divide the classical tooth attachment classifications into its different properties that seem to vary independently in at least part of the sampled taxa: tooth position on the jaw bone (relative to the jaws labial wall apical margins), tooth ankylosis, tooth delimitation (e.g. three-sided vs four-sided sockets), and presence or absence of replacement. In the present character, tooth position can be defined as: lingual to the dentary/maxillary labial wall; apically on the labial wall (sitting entirely on the crest forming the apex of the labial wall of the tooth bearing bones), such as in chamaeleonids, some priscagamids (e.g. *Mimeosaurus crassus*), and most sphenodontians (e.g.

Kallimodon, Pleurosaurus and *Sphenodon*); or apicolingually, in which part of the tooth base lies apically to the dorsal crest, but they also extend lingually to it, such as in many agamids and priscagamids (Borsuk-Białynicka & Moody 1984; Borsuk-Białynicka 1996; Evans *et al.* 2002; Simões *et al.* 2015), a condition previously described as "pleuroacrodonty" (Evans *et al.* 2002; Simões *et al.* 2015). See more in Bertin *et al.* (2018).

79. Marginal dentition, posterior teeth series, ankylosis to crista dorsalis (apex of labial wall) of dentary: absent (0)/ present (1) (S18, Ch. 211—re-phrased)

80. Marginal dentition, posterior teeth series, delimitation by tooth bearing bone: by a labial wall only (0)/ by a three-sided socket (1)/ by a four-sided socket (2)/ by a lingual and labial wall only (3) (S18, Ch. 212—re-phrased).
Remarks: See comments above for character #78 The three-sided socket condition occurs when interdental ridges are present connecting to the labial wall of the jaws. The four-sided socket condition occurs when the teeth are fully enclosed inside a socket or alveolus on the jaws. When teeth are at the apex of the labial wall, instead of medially to it, this character is scored as inapplicable.

81. Premaxillary teeth, general organization in adults: discrete teeth (0)/ merged into a chisel-like structure (1) (Su94, W94, Re96, AN03).
Remarks: Early evolving rhynchocephalians retain the premaxillary dentition composed of individually discernible teeth, as well as some species of *Clevosaurus*. However, most sphenodontians have all of their premaxillary dentition fused into a single enlarged and ventrally projecting tooth.

82. Maxillary teeth, posterior teeth series, posteromedial flanges: absent (0)/ present (1) (Su94, W94, Re96, AN03, A14, Ch. 50—modified).
Remarks: Occurs in several taxa with labiolingually compressed maxillary teeth, including clevosauruids, *Kallimodon*, *Saphaeosaurus* and pleurosaurids.

83. Maxillary teeth, posterior teeth series, anterior flange: absent (0)/ present (1) (AN03 A14, Ch. 51).

84. Dentary, anterior teeth series, caniniform successional tooth: absent (0)/ present (1) (NEW).
Remarks: A caniniform successional tooth occurs on the anteriormost section of the dentary in taxa such as *Cynosphenodon* and *Sphenodon*.

85. Dentary teeth, anterior teeth series, position relative to the jaw apical margin (dentary dorsal crest or maxillary ventral crest): lingual (0)/ apical (1)/ apicolingual (2) (G88a, Ch. 75 – modified).

86. Dentary teeth, posterior teeth series, shape of basal cross section: circular (0)/ labiolingually compressed (1)/ mesiodistally compressed (2)/ quadrangular (3) (NEW).
Remarks: Early rhynchocephalians possessed conic (peglike) marginal dentitions with a circular basal cross section. Later forms, such as clevosauruids, *Kallimodon*, *Saphaeosaurus* and pleurosaurids possessed labiolingually compressed teeth, and other forms, such as opisthodontids, possessed mesiodistally compressed teeth. *Sphenodon* possesses a quadrangular basal cross section of the posterior tooth series (clearly defined four sides), which is also observed in *Kawasphenodon* and *Opisthias* (Throckmorton *et al.* 1981; Apesteguía *et al.* 2014). In the latter state (quadrangular bases), whereas some of the teeth have equal sided quadrangular bases, some of the teeth in the posterior series may be slightly compressed labiolingually or mesiodistally. However, even in such instances, four clearly distinct sides are still discernible and form the perimeter of the tooth bases, which is considered here a qualitative difference to the features scored under state 1 (with narrow and curved mesial and distal borders) and state 2 (with narrow and curved lingual and labial borders)

87. Dentary teeth, posterior teeth series, concave anteriorly: absent (0)/ present (1) (S18, Ch. 209).
Remarks: This character is the result of the development of laterally projecting ridges, both lingually and labially, sometimes termed "anteromedial" and "anterolateral" ridges or flanges (Throckmorton *et al.* 1981; Apesteguia & Carballido 2014; Apesteguía *et al.* 2014; Jones *et al.* 2018). The development of those ridges, which are usually as thick as the central cusp [as they composed of extremely thick enamel (Throckmorton *et al.* 1981; Jones *et al.* 2018)], gives the posterior tooth series an anteriorly concave appearance when observed in lateral view, or in occlusal view (if there is some degree of apical tooth wear)—see also Fig. 4 in Throckmorton *et al.* (1981). This character is applicable only when teeth are mesiodistally compressed or has a quadrangular base, as it is logically impossible on teeth that are conical or labiolingually compressed. Observed in several Mesozoic taxa occurring in South and North America, such as *Toxolophosaurus* and *Eilenodon*.

88. Dentary teeth, posterior teeth series, anterior flanges: absent (0)/ present (1) (NEW).
Remarks: Very thin projections from the central cusp, lying on the longitudinal median axis of the tooth row. See potential variations below.

89. Dentary teeth, posterior teeth series, anterior flanges, orientation: anterolaterally (0)/ anteriorly (1)/ anteromedially (2) (NEW).

Remarks: The present character considers variations on the orientation of the anterior flange, as defined in character #88 only. Such an anterior flange may be oriented anteriorly (as in *Kallimodon, Homeosaurus* and pleurosaurids), or anterolaterally, in which the posteriormost portion of each tooth is hidden in lateral view by the anterior tip of the flange from each posteriorly succeeding tooth (as in clevosaurids and *Cynosphenodon*). An anteromedial orientation of the anterior flange has not been observed on the taxa sampled for this data set, but we provide it as third possible state for this character for potential future findings and analyses. Importantly, this character is conditional on the presence of an anteriorly directed flange (i.e. lying on the longitudinal median axis of the tooth row), and it is therefore not homologous to the "anterolateral" and "anteromedial" crests—laterally oriented and more robust dental projections composed of extremely thick enamel of taxa like *Opisthias*, *Toxolophosaurus* and *Eilenodon* (Throckmorton *et al.* 1981; Jones *et al.* 2018)— which confer the anteriorly concave aspect of the posterior additional teeth of those taxa.

90. Dentary teeth, posterior teeth series, posterior flanges: absent (0)/ present (1) (NEW).
Remarks: A posteriorly directed flange is rarely observed among rhynchocephalians, occurring, for example, in *Sphenodon punctatus*.

91. Dentary teeth, posterior teeth series,, labial surface, enamel ornamentation: smooth (0)/ striae (1)/ grooves (2) (AN03 A14, Ch. 58—re-phrased)

92. Dentary teeth, posterior teeth series, distal(=posterior) surface, groove,: absent (0)/ present (1) (A14, Ch. 72—recoded).

Postcranium

Vertebrae

Atlas-Axis

93. Atlas, pleurocentrum, fusion to axis: unfused (0)/ fused (1) (G88b, Ch. 133).
Remarks: Within Reptilia, the atlas centrum fuses to the axis in birds and lepidosaurs, forming the odontoid process of the axis (Hoffstetter & Gasc 1969).

94. Axis, ribs: present (0)/ absent (1) (S18, Ch. 248).

Postaxial vertebrae: Presacral/precloacal pleurocentra

95. Presacral pleurocentra, orientation of centrum: amphicoelous (0)/ procoelous (1)/ platycoelous (2) (G88a, Ch.84—modified).

96. Presacral pleurocentra, notochord, persistent in adults: present (0)/ absent (1) (B85, Ch. C6; Fig. 34 (Hoffstetter & Gasc 1969)).
Remarks: Although persistent notochords are most commonly seen in taxa with amphicoelic vertebrae, this character undergoes variation independent of the orientation of the pleurocentra. For instance, most geckos have amphicoelic vertebrae with notochordal canals, but geckos with procoelic vertebrae, such as *Sphaerodactylus parkeri*, also have a persistent notochord (Holder 1960).

97. Presacral pleurocentra, midventral crest, cervical vertebrae: absent (0)/ present (1) (G88b, Ch. 139; Fig. 37 (Hoffstetter & Gasc 1969)).

98. Presacral pleurocentra, midventral crest, posterior dorsal vertebrae: absent (0)/ present (1) (Ev88, Ch. B2; Fig. 34 (Hoffstetter & Gasc 1969)).

99. Sacral vertebrae, number: zero (0)/ one (1)/ two (2)/ three (3)/ four (4) (G88b, Ch. 141—modified).

100. Caudal vertebrae, autotomic septum: absent (0)/ present (1) (P86, Ch. 52; Fig. 52 (Hoffstetter & Gasc 1969))

Intercentra

101. Intercentra, on anteriormost caudal, shape: wedge-like elements (0)/ modified into chevron elements (1) (S18, Ch. 241).
Remarks: The anteriormost caudals that bear intercentra may have intercentra that differ in shape from the more posteriorly located intercentra, suggesting this region corresponds to a different domain in the caudal series. If any variation in the anterior caudal series occurs, this should be observed in the first caudal. Therefore, we score the condition for the anterior caudal intercentra based on the anteriormost caudal. Such variation is observed, for instance, in *Sphenodon*, which has wedge-like intercentra in the anteriormost caudals followed posteriorly by the intercentra forming chevron elements (forming the haemal arches)

102. Neural arches, presacral vertebrae, zygosphenes: absent (0)/ present (1) (LC00, Ch. 186; Fig. 41 (Hoffstetter & Gasc 1969)).

103. Neural arches and pleurocentrum, diapophysis, anterior dorsal vertebrae, fusion to parapophysis: absent (0)/ present (1) (S18, Ch. 250).

104. Neural arches and pleurocentrum, posterior dorsals, synapophyses, shape: elongated (0)/ circular (1) (NEW)
Remarks: Whereas the synapophyses in sphenodontians have an elongated articulatory facet, the latter is circular in most squamates.

Ribs

105. Presacral ribs, uncinate processes, anterior dorsals: absent (0)/ present (1) (G88b, Ch. L17).
Remarks: Neomorph cartilages that calcify in *Sphenodon* and crocodiles, and ossify in birds (Gauthier et al. 1988b), as well as within some marine reptiles.

106. Sacral ribs, distal forking: absent (0)/ on first sacral rib only (1)/ on first and second sacral ribs (2)/ on second sacral rib only (3) (Lee98, Ch. 189—modified)

107. Sacral/Cloacal ribs, fusion to pleurocentra: unfused (0)/ fused (1) (G88a, Ch. 87—modified).

108. Presternum, mineralized: absent (0)/ present (1) (B85, Ch. R5; Fig. 1.2 (Russell & Bauer 2008)).

109. Mineralized poststernal inscriptional ribs: absent (0)/ present (1) (E88, Ch. 110; Fig. 2 (Etheridge 1965)).
Remarks: Also termed postxiphisternal ribs, these are present posterior to the last presternal ribs and connected distally to the dorsal ribs in many instances. Alternatively, inscriptional ribs may occurs as "free" ribs, when not attaching to the dorsal ribs, such as in *Chalarodon* (Etheridge 1965). The latter condition is observed in many squamates and within rhynchocephalians (e.g. *Sphenodon punctatus* and *Kallimodon* pulchellus), but absent in observed specimens of pleurosaurs and *Clevosaurus hudsoni*) We only scored for mineralized inscriptional ribs, since cartilaginous ones may be incorrectly scored as absent in fossils.

Girdles

110. Scapula, posterior emargination: absent (0)/ present (1) (S18, Ch. 307)

111. Scapula, anterior emargination: absent (0)/ present (1) (E88, Ch. 111)
Remarks: In squamates, the anterior margin is not truly emarginated. In most squamates the anterior margin is relatively straight or convex. Iguanians may bear a scapular ray (see above), but the margin to which it connects to is also straight. Some rhynchocephalians have a true emargination, with the anterior margin being concave anteriorly and is not homologous to the scapular "emargination" or fenestra to which the scapular ray contributes in some squamates.

112. Procoracoid, coracoid emargination: absent (0)/ anterior emargination (1)/ anterior and posterior emarginations (2) (P86, Ch. 56 and 57; Fig. 1.2, 1.3 and 1.5 (Russell & Bauer 2008))
Remarks: The anterior coracoid emargination is separated from the scapulocoracoid emargination dorsally by the first (anterior) coracoid ray and the posterior coracoid emargination is separated from an anterior coracoid emargination by the second (posterior) coracoid ray. The posterior emargination only occurs if the anterior one is also present; thus they are part of the same transformation series and logically nested.

113. Clavicles, secondary dorsal curvature anteriorly: absent (0)/ present (1) (E88, Ch. 116; Fig. in G12, Ch. 502).

114. Ilia, anterior pubic process: absent (0)/ present (1) (B85, Ch. J12).
Remarks: The anterior extension of the ilium dorsal to the pubis is present in some early reptiles (e.g. *Hovasaurus* and *Acerosodontosaurus*) and most squamates, but this process is absent in most other reptilian lineages.

115. Ilia, anterior (=preacetabular) process: absent (0)/ present (1) (Lee97, Ch. 132; Fig. 1.14 (Russell & Bauer 2008)).
Remarks: Process located on the iliac blade or the anterior extension of the ilium (character #114)

116. Pubes, obturator foramen: absent (0)/ complete foramen (1)/ notch (2) (S18, Ch. 333).

117. Ischia, ischiadic tuberosity: absent (0)/ present (1) (J94, Ch. 10—modified; Fig. 1.14 (Russell & Bauer 2008)).

Limbs

118. Humeri, ectepicondyle foramen: absent (0)/ groove (1)/ notch (2)/ complete foramen (3) (G88b, Ch. 162 and 163—modified).

119. Humeri, entepicondyle foramen: absent (0)/ opening dorsally only (1)/ opening ventrally only (2)/ fully open ventrally and dorsally (3) (S18, Ch. 341).
Remarks: Most reptiles with an entepicondylar foramen display it on the dorsal surface of the distal end of the humerus, state “1”. However, some sphenodontians develop this foramen ventrally only, and in sauropterygians and early reptiles there is a full opening connecting the dorsal and ventral sides of the humerus. The character states herein do not occur in conjunction, thus being mutually exclusive and being better coded as different character states within a single character instead of split into contingently coded characters.

120. Humeri, expanded radial condyle (= capitelum): present (0)/ absent (1) (S18, Ch. 344).

121. Humeri, secondary ossification of epiphyses: absent (0)/ present (1) (B85, Ch. X1—modified)
Remarks: Observed in most squamates and rhynchocephalians.

122. Radia, distal epiphysis, styloid process: absent (0)/ present (1) (G88a, Ch. 99; Fig. 9 in G88a).
Remarks: Observable in ventral and medial aspect in articulation with the radiale. This feature may not be seen in fossil taxa with the epiphyses not preserved (in which case they are scored as missing data).

123. Ulnae, distal epiphysis, expansion: absent (0)/ present (1) (B85, Ch. X3; Fig. 9 in G88a).
Remarks: A distally expanded or "ball-like" distal epiphysis of the ulna is observed within squamates. The presence of a proximal concavity on the ulnare of lizards is dependent upon the presence of a distal ball-like distal epiphysis of the ulna and the formation of a ball-socket articulation. Therefore, a character on the proximal concavity on the ulnare is not included in the present dataset.

124. Pisiform: absent (0)/ present (1) (Mo99, Ch. 67)
Remarks: Postaxial element, positioned ventrally to the ulnare in squamates and other reptiles.

125. Distal carpal 1: present (0)/ absent (1) (G88a, Ch. 103; Fig. 4c and d in G88a).
Remarks: The element that partially occupies the position of the first distal carpal in lizards represents the medial centrale (Russell & Bauer 2008) [lateral central *sensu* (Romer 1956)], resulting from the fusion of the first carpal to the first metacarpal, and a slight shift in position of the medial centrale (Carroll 1977; Gauthier *et al.* 1988a). This fusion usually results in an enlarged epiphysis of the first metacarpal (which also occupies part of the position of the first carpal) followed by a reduced number of anterior carpal elements. Because the underlying developmental process of fusion, this cannot be assessed in the vast majority of sampled taxa, the wording of the present character reflects the absence of DC1 only. Nevertheless, among all taxa studied herein the absence of a distinct DC1 always seemed to reflect the fusion of the latter to the first metacarpal (by possessing an expanded proximal epiphysis on MC I) Therefore, all taxa scored with the absent condition herein are primarily homologous based on similarity, likely all due to the fusion of the DC1 to the first metacarpal.

126. Astragalus and calcaneum: as totally separate elements (0)/ fused (1) (B85, Ch. X10).

127. Distal tarsal 2: present (0)/ absent (1) (Ev88, Ch. L20).
Remarks: Absent in squamates.

128. Distal tarsal 4, proximal peg: present (0)/ absent (1) (Ev88, Ch. J1; Fig. 1.20 (Russell & Bauer 2008)) .
Remarks: This proximal peg is better observed in ventral aspect on the tarsus of lizards in articulation with the astragalocalcaneum. The proximal peg is responsible for the mesotarsal articulation in lizards and is also observed in other reptilian groups. The presence of a distomesial articulatory surface on the astragalocalcaneum that articulates with this proximal peg is dependent upon this character, and therefore it is not included as a character here to avoid redundancy.

129. Metatarsal 5, hooked: absent (0)/ present (1) (B85, Ch. C14; Fig. 1.20 (Russell & Bauer 2008))
Remarks: Benton (1985) described this character originally as a non-lepidosaur type of hooked fifth metatarsal, such as lacking the plantar tubercle observed in lepidosaurs. However, the latter feature is placed herein as a distinct character.

130. Metatarsal 5, plantar tubercle: absent (0)/ present (1) (DBRe96, Ch. 156; Fig. 1.20 (Russell & Bauer 2008)).

131. Gastralia: absent (0)/ present (1) (G88a, Ch. 136).
Remarks: These are dermal ossifications that should not be confused with the inscriptional ribs (of endochondral ossification) present in lizards, sometimes erroneously referred to as “abdominal ribs”, “gastralia”, “parasternal chevrons” or “parasternal ribs” (Etheridge 1965).

References

Apesteguía, S. 2008. *Esfenodontes (Reptilia, Lepidosauria) del Cretácico Superior de Patagonia: anatomía y filogenia*. Unpublished PhD thesis, Universidad Nacional de La Plata, 535 pp.
Apesteguia, S. & Carballido, J. L. 2014. A new eilenodontine (Lepidosauria, Sphenodontidae) from the Lower Cretaceous of central Patagonia. *Journal of Vertebrate Paleontology*, **34**(2), 303-317.
Apesteguía, S., Gómez, R. O. & Rougier, G. W. 2012. A basal sphenodontian (Lepidosauria) from the Jurassic of Patagonia: new insights on the phylogeny and biogeography of Gondwanan rhynchocephalians. *Zoological Journal of the Linnean Society*, **166**(2), 342-360.
Apesteguía, S., Gómez, R. O. & Rougier, G. W. 2014. The youngest South American rhynchocephalian, a survivor of the K/Pg extinction. *Proceedings of the Royal Society B: Biological Sciences*, **281**(1792), 20140811.
Apesteguia, S. & Novas, F. E. 2003. Large Cretaceous sphenodontian from Patagonia provides insight into lepidosaur evolution in Gondwana. *Nature*, **425**(6958), 609-612.
Benton, M. J. 1985. Classification and phylogeny of the diapsid reptiles. *Zoological Journal of the Linnean Society*, **84**(2), 97-164.

Bertin, T. J., Thivichon-Prince, B., LeBlanc, A. R., Caldwell, M. W. & Viriot, L. 2018. Current perspectives on tooth implantation, attachment, and replacement in Amniota. *Frontiers in Physiology*, **9**.
Borsuk-Białynicka, M. 1996. The Late Cretaceous lizard *Pleurodontagama* and the origin of tooth permanency in Lepidosauria. *Acta Palaeontologica Polonica*, **41**, 231-252.
Borsuk-Białynicka, M. & Moody, S. M. 1984. Priscagaminae. A new subfamily of the Agamidae (Sauria) from the Late Cretaceous of the Gobi Desert. *Acta Palaeontologica Polonica*, **29**(1-2), 51-81.
Carroll, R. L. 1969. Problems of the origin of reptiles. *Biological Reviews*, **44**(3), 393-431.
Carroll, R. L. 1977. The origin of lizards. Pp. 1-28 in S.M. Andrews, R.S. Miles & A.D. Walker (eds) *Problems in Vertebrate Evolution*. Academic Press, London and New York.
Conrad, J. L. 2008. Phylogeny and systematics of Squamata (Reptilia) based on morphology. *Bulletin of the American Museum of Natural History*, **310**, 1-182.
Conrad, J. L. & Norell, M. A. 2006. High-resolution X-ray computed tomography of an Early Cretaceous gekkonomorph (Squamata) from Öösh (Övörkhangai; Mongolia). *Historical Biology*, **18**(4), 405-431.
de Braga, M. & Carroll, R. L. 1993. The origin of mosasaurs as a model of macroevolutionary patterns and processes. *Evolutionary Biology*, **27**, 245-322.
de Braga, M. & Rieppel, O. 1997. Reptile phylogeny and the interrelationships of turtles. *Zoological Journal of the Linnean Society*, **120**, 281 - 354.
Estes, R., de Queiroz, K. & Gauthier, J. A. 1988. Phylogenetic relationships within Squamata. Pp. 119-281 in R. Estes & G. Pregill (eds) *Phylogenetic relationships of the lizard families*. Stanford University Press, Stanford.
Etheridge, R. 1965. The Abdominal Skeleton of Lizards in the Family Iguanidae. *Herpetologica*, **21**(3), 161-168.
Evans, S. E. 1988. The early history and relationships of the Diapsida. Pp. 221-260 in M.J. Benton (ed) *The phylogeny and classification of the tetrapods*. Clarendon Press, Oxford.
Evans, S. E., Prasad, G. V. R. & Manhas, B. K. 2002. Fossil lizards from the Jurassic Kota Formation of India. *Journal of Vertebrate Paleontology*, **22**(2), 299-312.
Fraser, N. C. 1982. A new rhynchocephalian from the British Upper Trias. *Palaeontology*, **25**(4), 709-725.
Fraser, N. C. 1988. The Osteology and Relationships of *Clevosaurus* (Reptilia: Sphenodontida). *Philosophical Transactions of the Royal Society of London, Series B: Biological Sciences*, **321**(1204), 125-178.
Fraser, N. C. & Benton, M. J. 1989. The Triassic reptiles *Brachyrhinodon* and *Polysphenodon* and the relationships of the sphenodontids. *Zoological Journal of the Linnean Society*, **96**(4), 413-445.
Gauthier, J. A., Estes, R. & de Queiroz, K. 1988a. A phylogenetic analysis of Lepidosauromorpha. Pp. 15-98 in R. Estes & G. Pregill (eds) *Phylogenetic relationships of the lizard families*. Stanford University Press, Stanford.
Gauthier, J. A., Kearney, M., Maisano, J. A., Rieppel, O. & Behlke, A. D. B. 2012. Assembling the squamate tree of life: perspectives from the phenotype and the fossil record. *Bulletin of the Peabody Museum of Natural History*, **53**(1), 3-308.
Gauthier, J. A., Kluge, A. G. & Rowe, T. 1988b. Amniote Phylogeny and the Importance of Fossils. *Cladistics*, **4**(2), 105-209.

Harrison, H. S. 1901. The development and succession of teeth in *Hatteria punctata*. *Quarterly Journal of Microscopical Science*, **44**, 161-213.
Hoffstetter, R. & Gasc, J.-P. 1969. Vertebrae and ribs of modern reptiles. Pp. 201-310 in C. Gans, A.d.A. Bellairs & T.S. Parsons (eds) *Biology of the Reptilia*. Academic Press, London and New York.
Holder, L. A. 1960. The comparative morphology of the axial skeleton in the Australian Gekkonidae. *Journal of the Linnean Society of London, Zoology*, **44**(297), 300-335.
Jones, M. E., Lucas, P. W., Tucker, A. S., Watson, A. P., Sertich, J. J., Foster, J. R., Williams, R., Garbe, U., Bevitt, J. J. & Salvemini, F. 2018. Neutron scanning reveals unexpected complexity in the enamel thickness of an herbivorous Jurassic reptile. *Journal of The Royal Society Interface*, **15**(143), 20180039.
Juul, L. 1994. The phylogeny of basal archosaurs. *Palaeontologia Africana*, **31**, 1-38.
Laurin, M. & Reisz, R. R. 1995. A reevaluation of early amniote phylogeny. *Zoological Journal of the Linnean Society*, **113**(2), 165-223.
Lee, M. S. & Caldwell, M. W. 2000. *Adriosaurus* and the affinities of mosasaurs, dolichosaurs and snakes. *Journal of Paleontology*, **74**(5), 915-937.
Lee, M. S. Y. 1997. The phylogeny of varanoid lizards and the affinities of snakes. *Philosophical Transactions of the Royal Society of London, Series B: Biological Sciences*, **352**(1349), 53-91.
Lee, M. S. Y. 1998. Convergent evolution and character correlation in burrowing reptiles: towards a resolution of squamate relationships. *Biological Journal of the Linnean Society*, **65**(4), 369-453.
Longrich, N. R., Bhullar, B.-A. S. & Gauthier, J. A. 2012. Mass extinction of lizards and snakes at the Cretaceous–Paleogene boundary. *Proceedings of the National Academy of Sciences*.
Modesto, S. P. & Sues, H.-D. 2004. The skull of the Early Triassic archosauromorph reptile *Prolacerta broomi* and its phylogenetic significance. *Zoological Journal of the Linnean Society*, **140**(3), 335-351.
Motani, R. 1999. Phylogeny of the Ichthyopterygia. *Journal of Vertebrate Paleontology,* **19**, 473 - 496.
Oelrich, T. M. 1956. The anatomy of the head of *Ctenosaura pectinata* (Iguanidae). *Miscellaneous Publications - Museum of Zoology, University of Michigan*, **94**, 1-122.
Pregill, G. K., Gauthier, J. A. & Greene, H. W. 1986. The evolution of helodermatid squamates with description of a new taxon and an overview of Varanoidea. *Transactions of the San Diego Society of Natural History*, **21**, 167-202.
Reisz, R. R. & Dilkes, D. W. 2003. *Archaeovenator hamiltonensis*, a new varanopid (Synapsida: Eupelycosauria) from the Upper Carboniferous of Kansas. *Canadian Journal of Earth Sciences*, **40**(4), 667-678.
Reynoso, V.-H. 1996. *Early Cretaceous lepidosaurs (Reptilia: Diapsida) from Central Mexico and the phylogeny of lepidosauromorphs*. Unpublished PhD thesis, McGill University, 297 pp.
Rieppel, O. 1984. The cranial morphology of the fossorial lizard genus *Dibamus* with a consideration of its phylogenetic relationships. *Journal of Zoology*, **204**(3), 289-327.
Rieppel, O. 1994. Osteology of *Simosaurus gaillardoti* and the relationships of stem-group Sauropterygia *Fieldiana (Geology)*, **28**, 1-85.

Rieppel, O. & Zaher, H. 2000. The intramandibular joint in squamates: and the phylogenetic relationships of the fossil snake *Pachyrhachis problematicus* Haas. *Fieldiana (Geology - New Series)*, **43**, 1-69.
Romer, A. S. 1956. *Osteology of the Reptiles*. University of Chicago Press, Chicago.
Russell, A. P. & Bauer, A. M. 2008. The appendicular locomotor apparatus of *Sphenodon* and normal-limbed squamates. Pp. 1-465 in C. Gans, A. Gaunt & K. Adler (eds) *Biology of the Reptilia*. Society for the Study of Amphibians and Reptiles, Ithaca, NY.
Simões, T. R., Caldwell, M. W., Tałanda, M., Bernardi, M., Palci, A., Vernygora, O., Bernardini, F., Mancini, L. & Nydam, R. L. 2018. The origin of squamates revealed by a Middle Triassic lizard from the Italian Alps. *Nature*, **557**(7707), 706-709.
Simões, T. R., Wilner, E., Caldwell, M. W., Weinschütz, L. C. & Kellner, A. W. A. 2015. A stem acrodontan lizard in the Cretaceous of Brazil revises early lizard evolution in Gondwana. *Nature Communications*, **6**(8149), 9149.
Sues, H.-D., Shubin, N. H. & Olsen, P. E. 1994. A new sphenodontian (Lepidosauria: Rhynchocephalia) from the McCoy Brook Formation (Lower Jurassic) of Nova Scotia, Canada. *Journal of Vertebrate Paleontology*, **14**(3), 327-340.
Throckmorton, G. S., Hopson, J. A. & Parks, P. 1981. A Redescription of *Toxolophosaurus cloudi* Olson, a Lower Cretaceous Herbivorous Sphenodontid Reptile. *Journal of Paleontology*, **55**(3), 586-597.
Whiteside, D. I. 1986. The Head Skeleton of the Rhaetian Sphenodontid *Diphydontosaurus avonis* gen. et sp. nov. and the Modernizing of a Living Fossil. *Philosophical Transactions of the Royal Society of London, Series B: Biological Sciences*, **312**(1156), 379-430.
Wu, X.-C. 1994. Late Triassic-Early Jurassic sphenodontians from China and the phylogeny of the Sphenodontia. Pp. 38-69 in N.C. Fraser & H.D. Sues (eds) *In the shadow of the dinosaurs: early Mesozoic tetrapods*. Cambridge University Press, Cambridge
