# Supplementary figures and images for: Sphenodontian phylogeny and the impact of model choice in Bayesian morphological clock estimates of divergence times and evolutionary rates
Source: BMC Biol. 2020 Dec 7;18:191. doi: 10.1186/s12915-020-00901-5 (PMC7720557; doi:10.1186/s12915-020-00901-5)

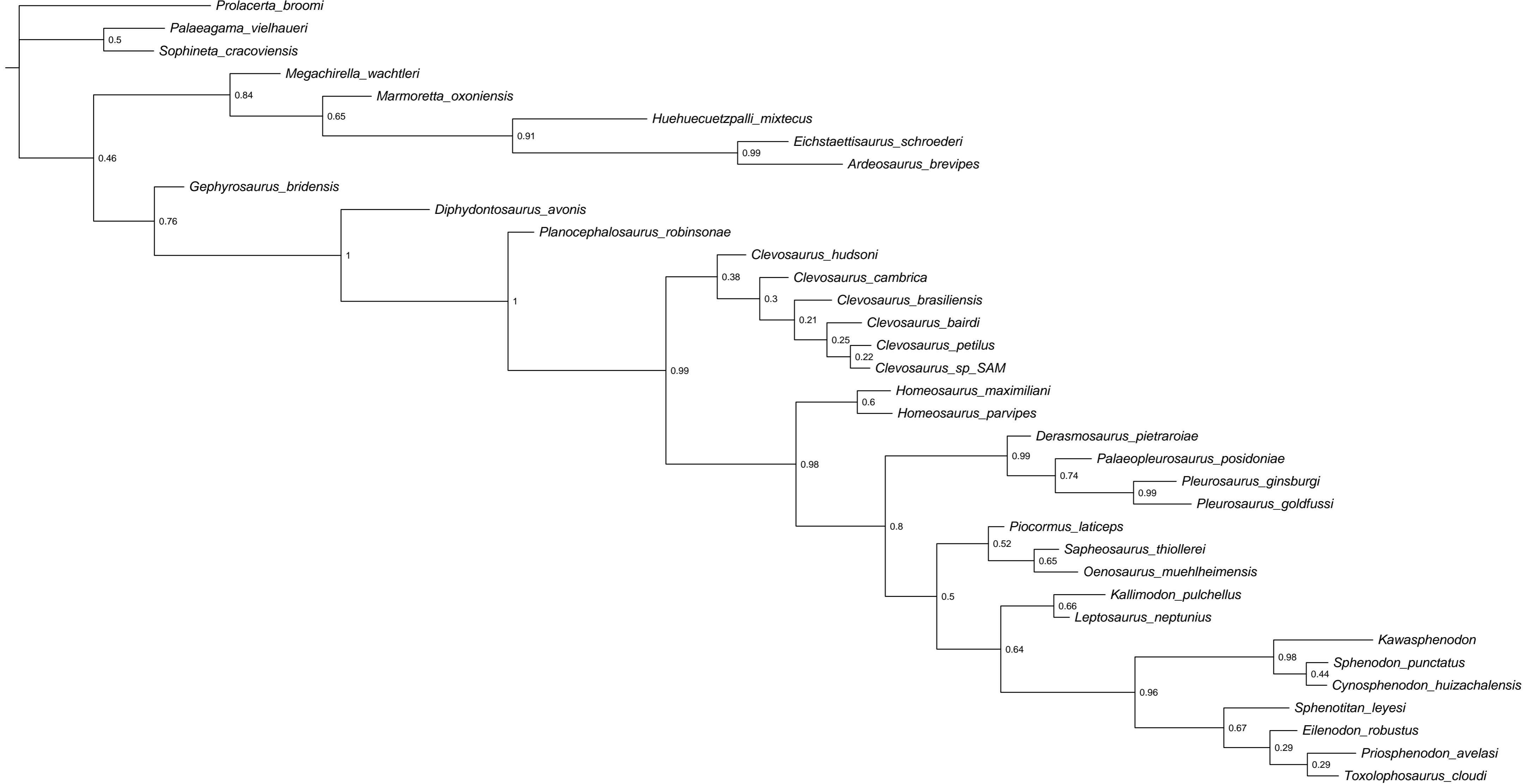

0.2

Supplement: Supplementary file 6 — Additional file 6. Input files including the dataset and all necessary coding (see Mr. Bayes blocks) to reproduce the analyses. [file 12915_2020_901_MOESM6_ESM.zip › InputFiles&OutputTrees/Bayes/Bayes_AllCompCon.t.con.pdf]

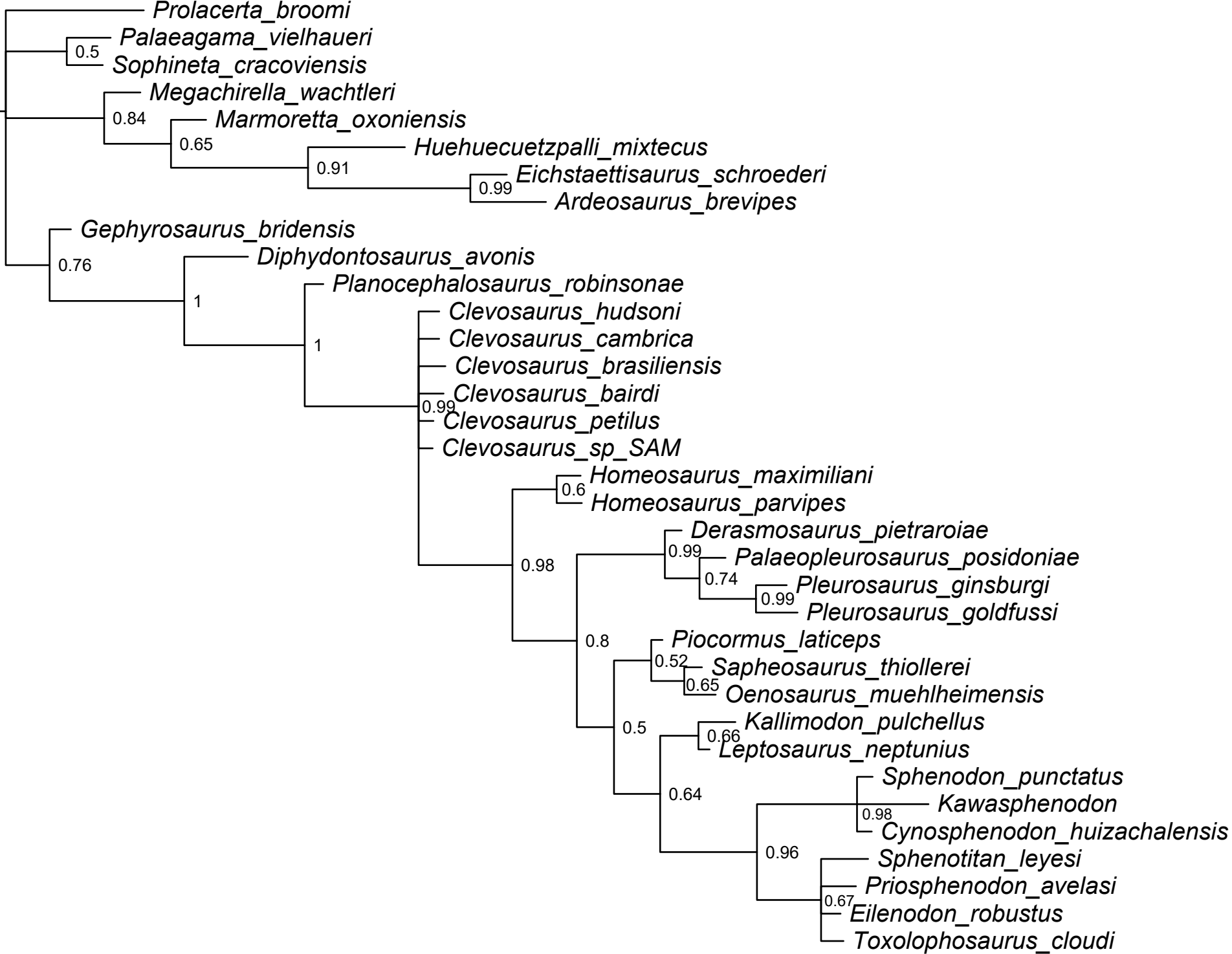

0.2

Supplement: Supplementary file 6 — Additional file 6. Input files including the dataset and all necessary coding (see Mr. Bayes blocks) to reproduce the analyses. [file 12915_2020_901_MOESM6_ESM.zip › InputFiles&OutputTrees/Bayes/Bayes_MRC.t.con.pdf]

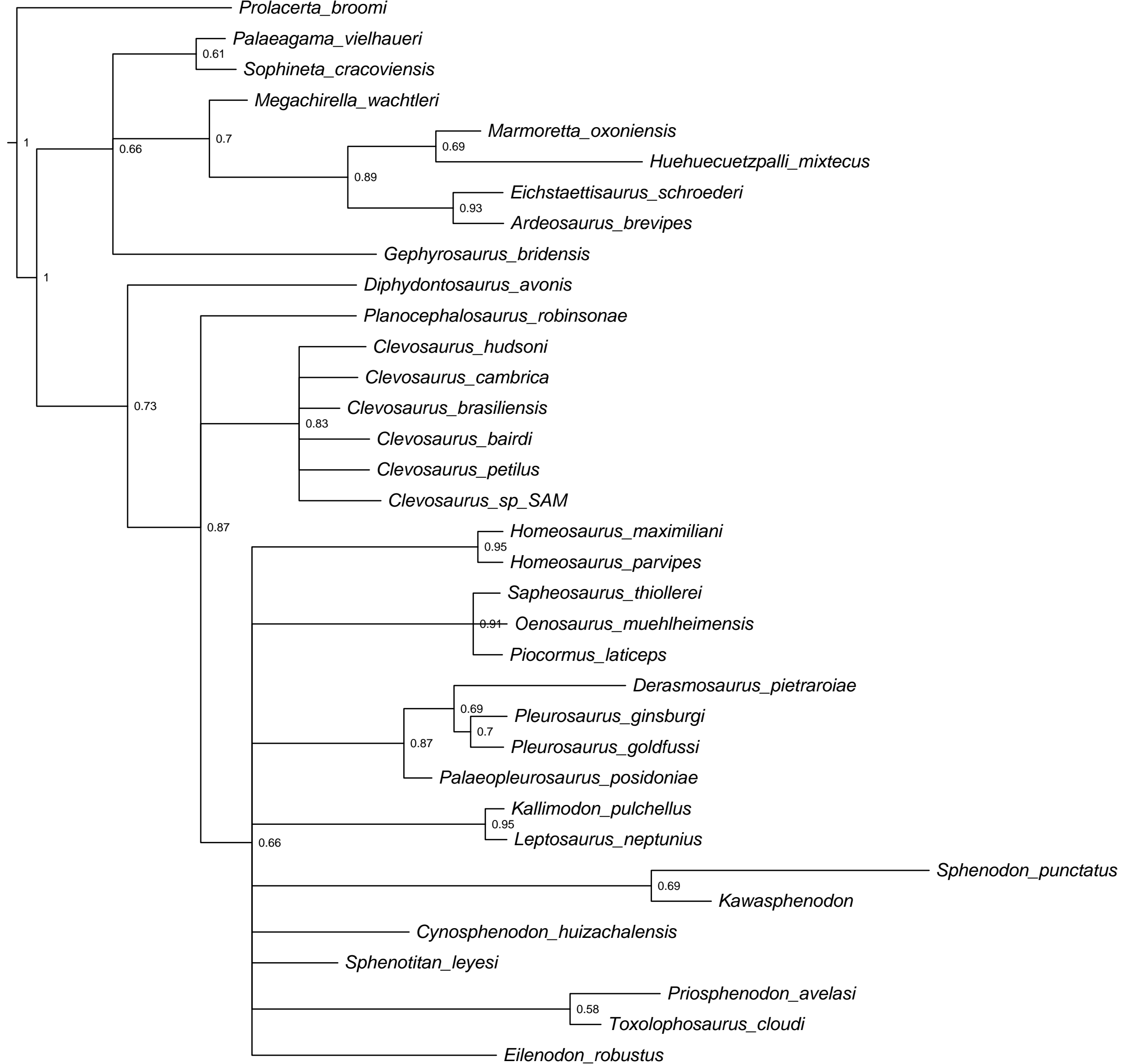

40.0

Supplement: Supplementary file 6 — Additional file 6. Input files including the dataset and all necessary coding (see Mr. Bayes blocks) to reproduce the analyses. [file 12915_2020_901_MOESM6_ESM.zip › InputFiles&OutputTrees/BayesCalibrated/Diversity/BayesCal_IGR_ln_p1_60G_Dv/BayesCal_IGR_ln_p1_Dv_MRC.t.con.tre.pdf]

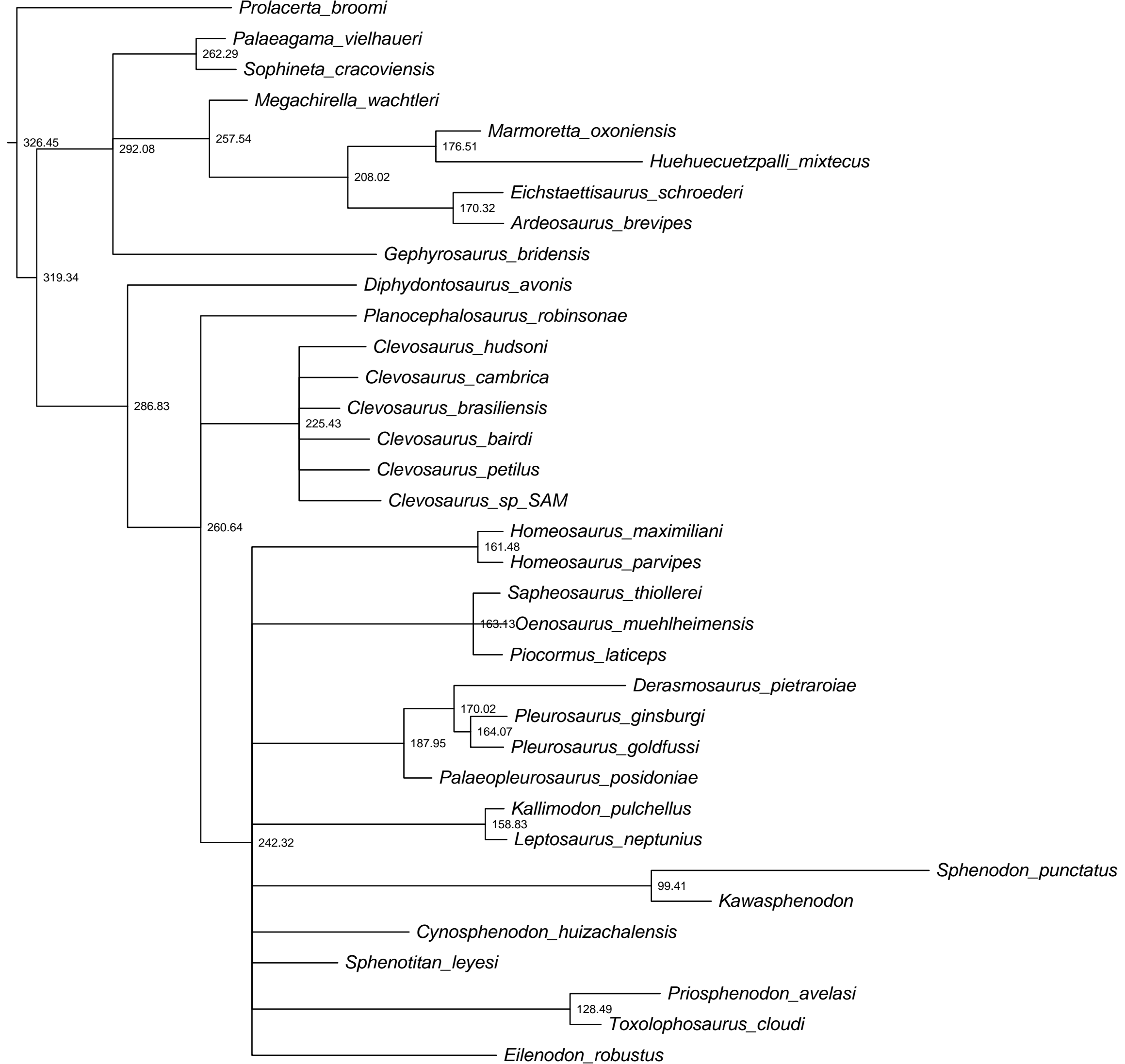

40.0

Supplement: Supplementary file 6 — Additional file 6. Input files including the dataset and all necessary coding (see Mr. Bayes blocks) to reproduce the analyses. [file 12915_2020_901_MOESM6_ESM.zip › InputFiles&OutputTrees/BayesCalibrated/Diversity/BayesCal_IGR_ln_p1_60G_Dv/BayesCal_IGR_ln_p1_Dv_MRC.t.con.tre_Age.pdf]

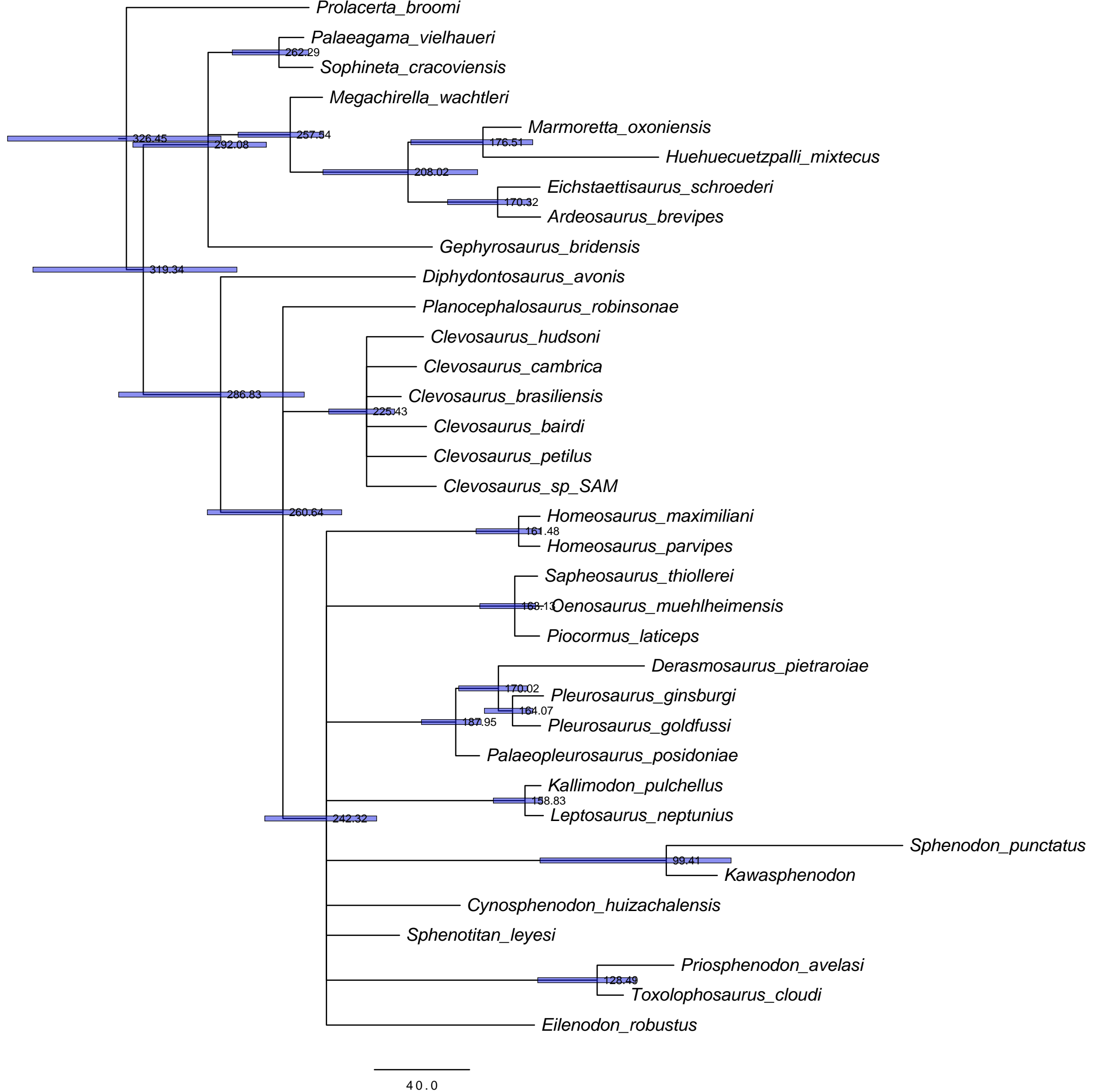

Supplement: Supplementary file 6 — Additional file 6. Input files including the dataset and all necessary coding (see Mr. Bayes blocks) to reproduce the analyses. [file 12915_2020_901_MOESM6_ESM.zip › InputFiles&OutputTrees/BayesCalibrated/Diversity/BayesCal_IGR_ln_p1_60G_Dv/BayesCal_IGR_ln_p1_Dv_MRC.t.con.tre_AgeBars.pdf]

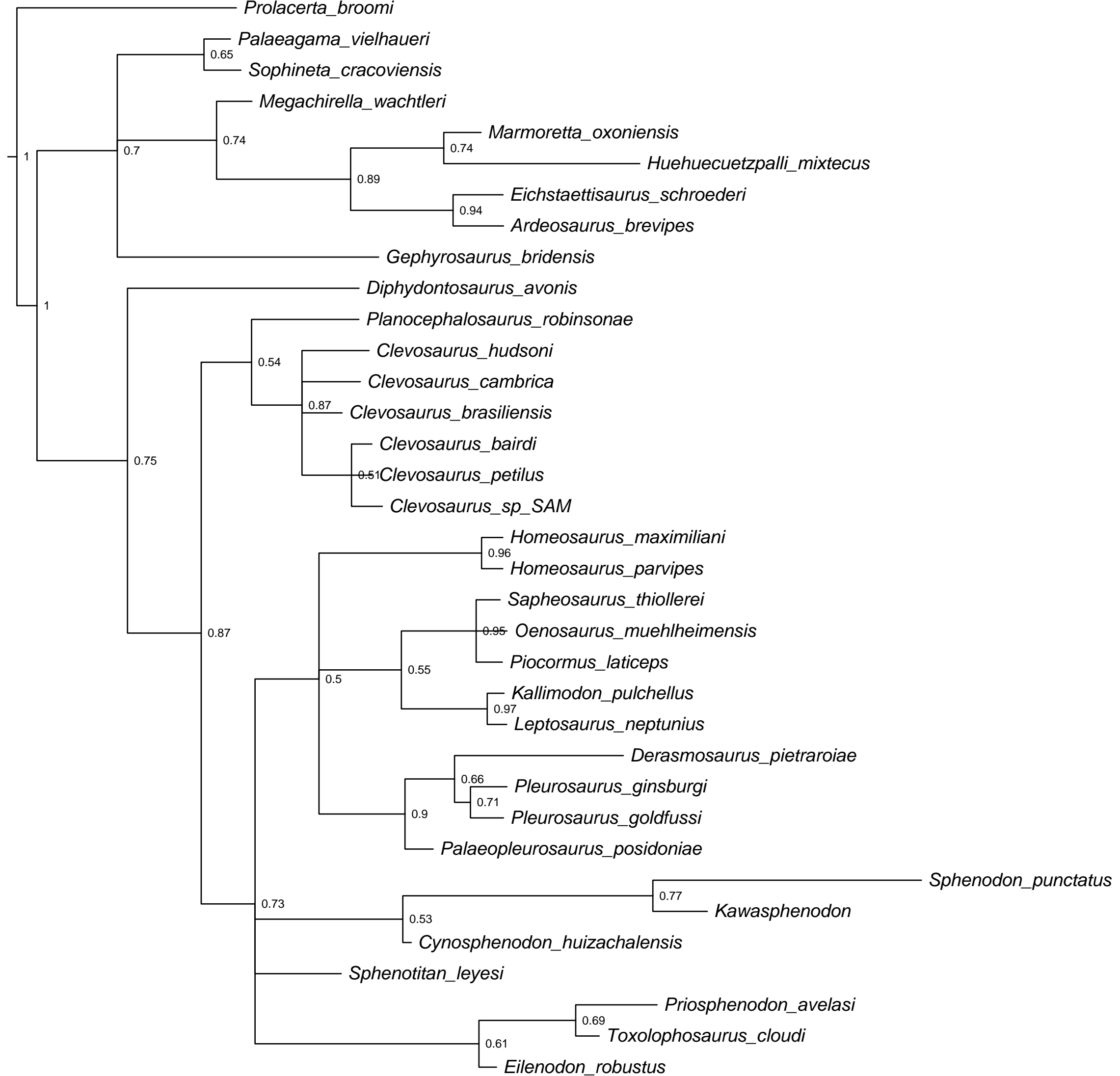

Supplement: Supplementary file 6 — Additional file 6. Input files including the dataset and all necessary coding (see Mr. Bayes blocks) to reproduce the analyses. [file 12915_2020_901_MOESM6_ESM.zip › InputFiles&OutputTrees/BayesCalibrated/Diversity/BayesCal_TK02_ln_p1_60G_Dv/BayesCal_TK02_ln_p1_Dv_MRC.t.con.tre.pdf]

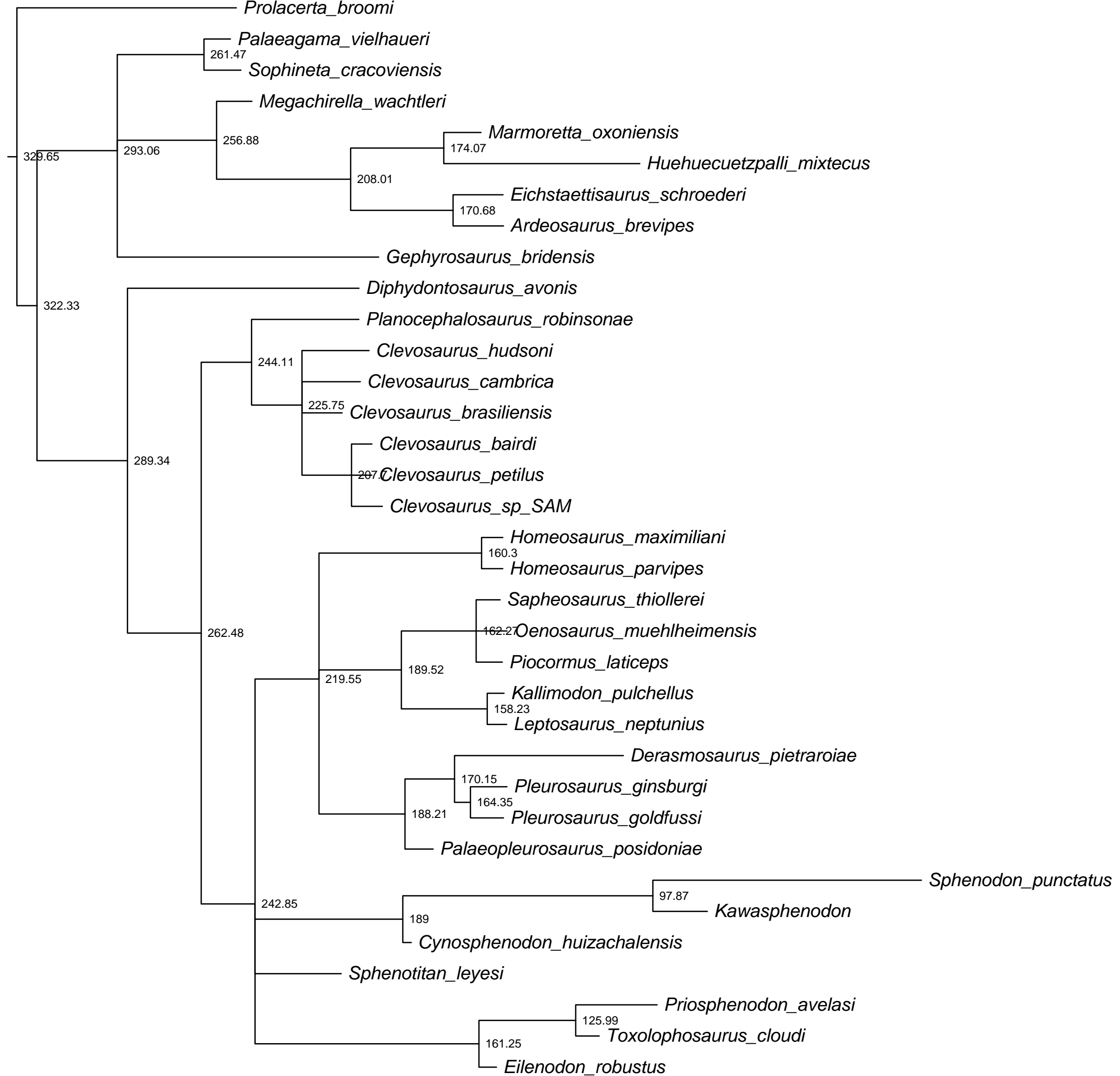

Supplement: Supplementary file 6 — Additional file 6. Input files including the dataset and all necessary coding (see Mr. Bayes blocks) to reproduce the analyses. [file 12915_2020_901_MOESM6_ESM.zip › InputFiles&OutputTrees/BayesCalibrated/Diversity/BayesCal_TK02_ln_p1_60G_Dv/BayesCal_TK02_ln_p1_Dv_MRC.t.con.tre_Age.pdf]

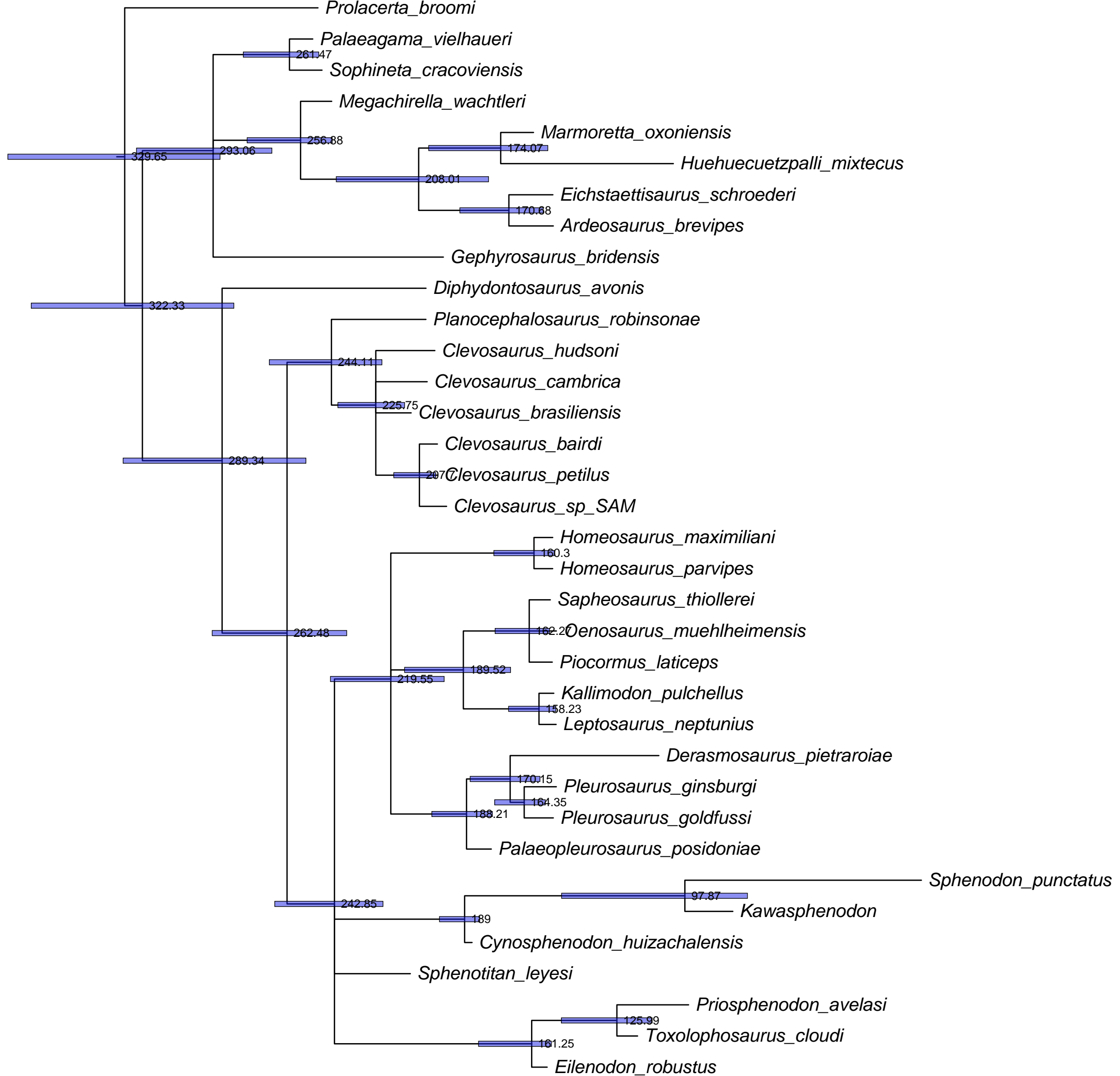

40.0

Supplement: Supplementary file 6 — Additional file 6. Input files including the dataset and all necessary coding (see Mr. Bayes blocks) to reproduce the analyses. [file 12915_2020_901_MOESM6_ESM.zip › InputFiles&OutputTrees/BayesCalibrated/Diversity/BayesCal_TK02_ln_p1_60G_Dv/BayesCal_TK02_ln_p1_Dv_MRC.t.con.tre_AgeBars.pdf]

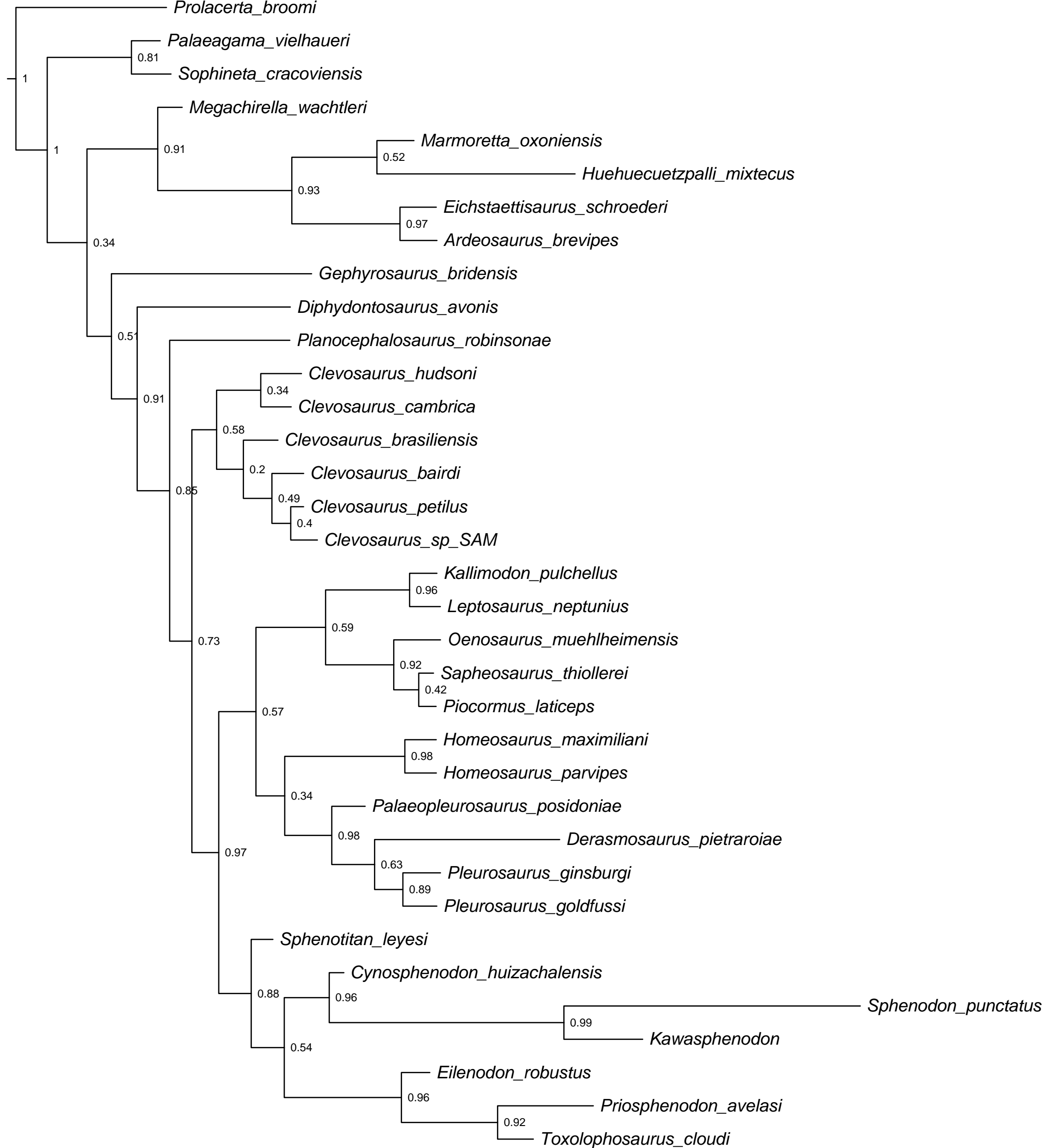

Supplement: Supplementary file 6 — Additional file 6. Input files including the dataset and all necessary coding (see Mr. Bayes blocks) to reproduce the analyses. [file 12915_2020_901_MOESM6_ESM.zip › InputFiles&OutputTrees/BayesCalibrated/Diversity(NoSA)/BayesCal_IGR_ln_p1_60G_DvNoSA/BayesCal_IGR_ln_p1_DvNoSA_AllCom.t.con.tre.pdf]

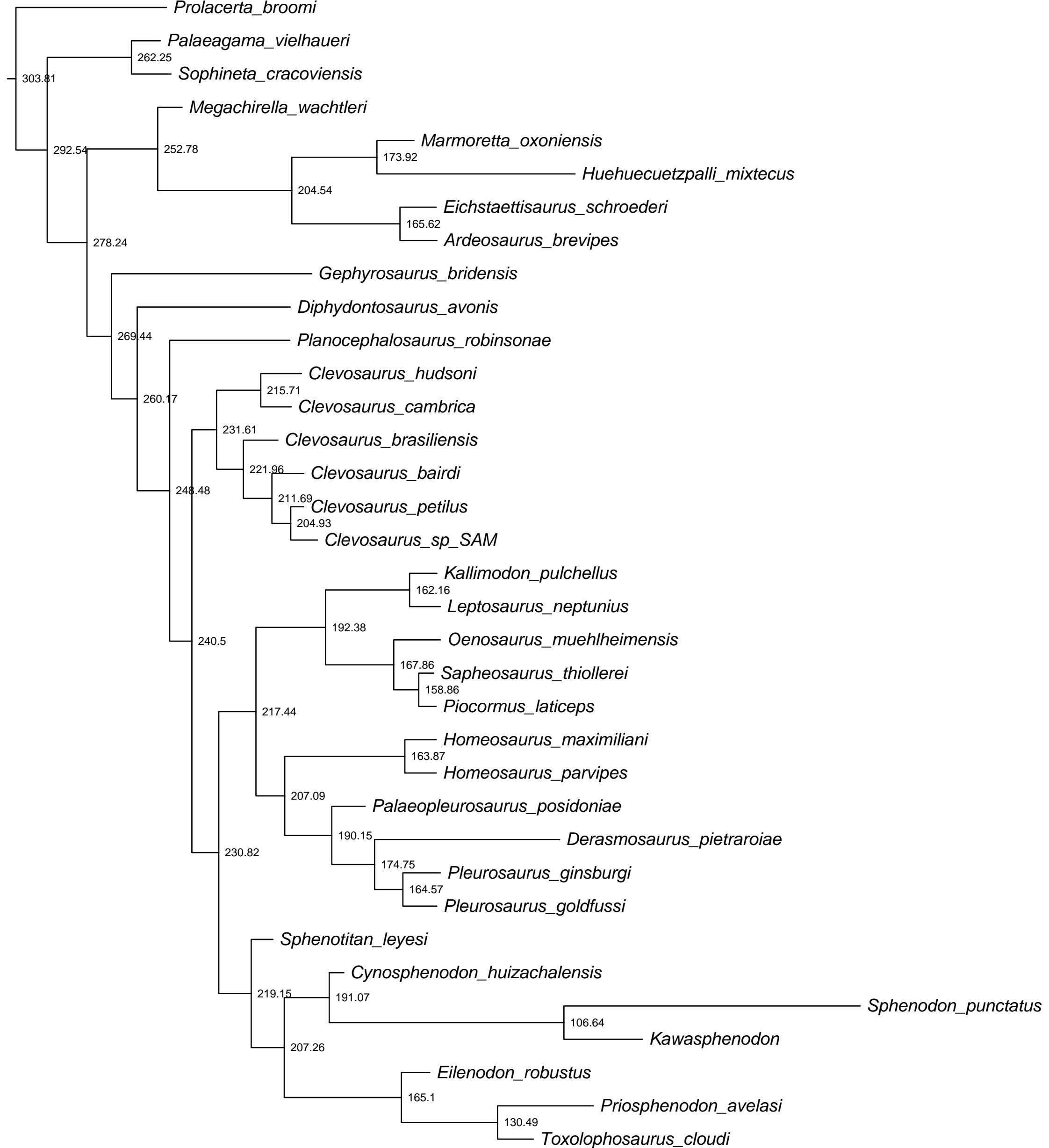

40.0

Supplement: Supplementary file 6 — Additional file 6. Input files including the dataset and all necessary coding (see Mr. Bayes blocks) to reproduce the analyses. [file 12915_2020_901_MOESM6_ESM.zip › InputFiles&OutputTrees/BayesCalibrated/Diversity(NoSA)/BayesCal_IGR_ln_p1_60G_DvNoSA/BayesCal_IGR_ln_p1_DvNoSA_AllCom.t.con.tre_Age.pdf]

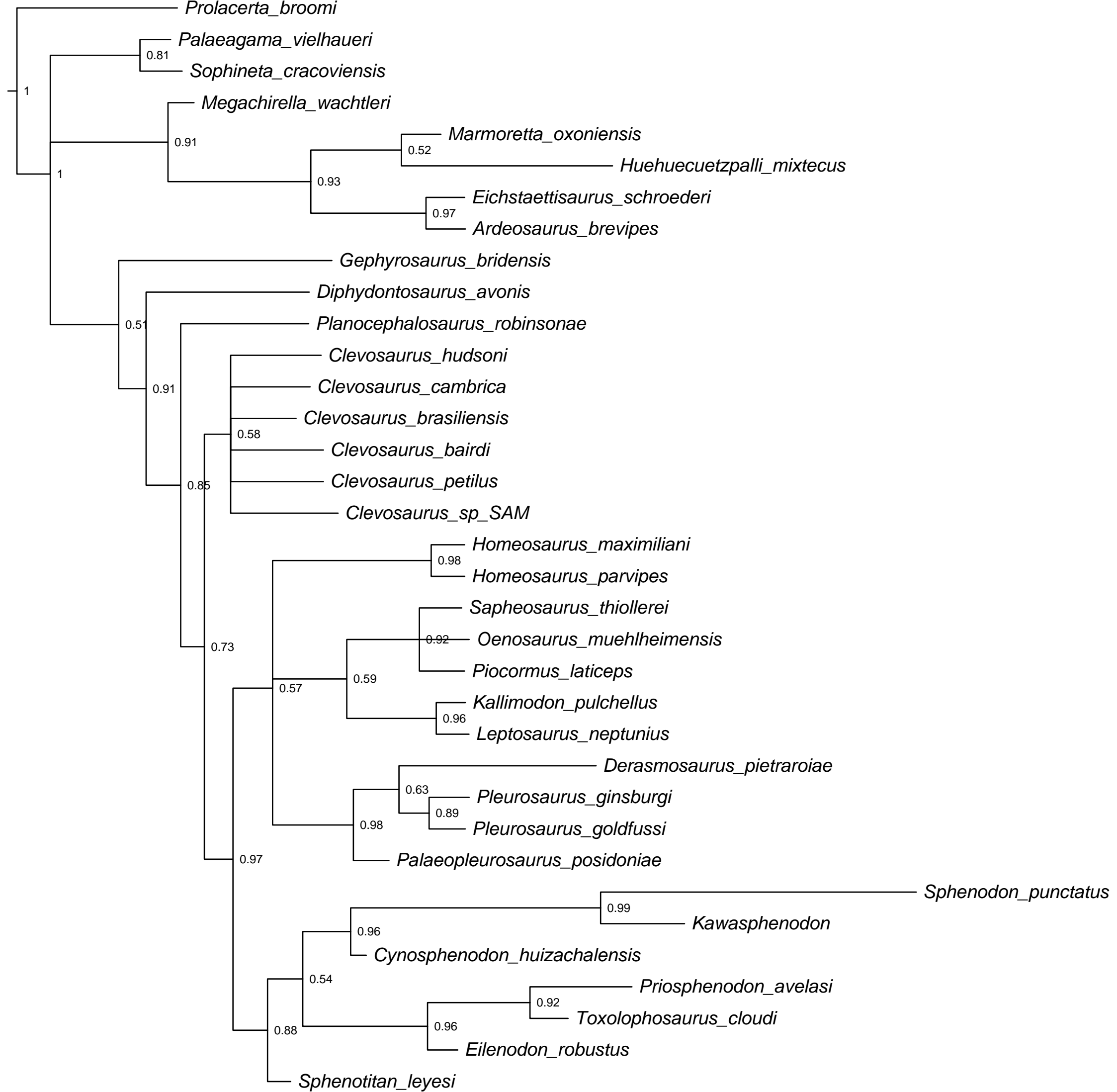

40.0

Supplement: Supplementary file 6 — Additional file 6. Input files including the dataset and all necessary coding (see Mr. Bayes blocks) to reproduce the analyses. [file 12915_2020_901_MOESM6_ESM.zip › InputFiles&OutputTrees/BayesCalibrated/Diversity(NoSA)/BayesCal_IGR_ln_p1_60G_DvNoSA/BayesCal_IGR_ln_p1_DvNoSA_MRC.t.con.tre.pdf]

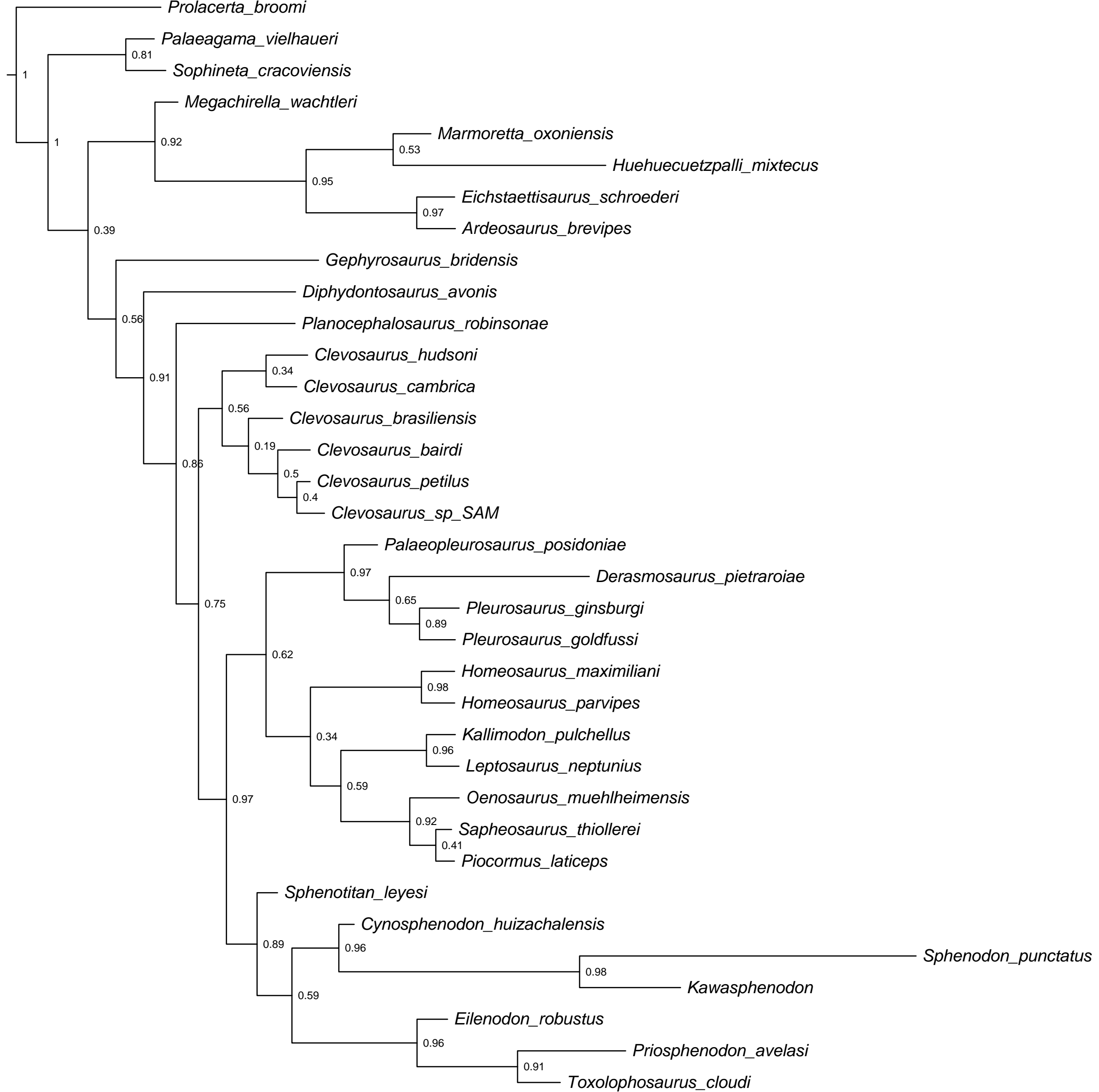

30.0

Supplement: Supplementary file 6 — Additional file 6. Input files including the dataset and all necessary coding (see Mr. Bayes blocks) to reproduce the analyses. [file 12915_2020_901_MOESM6_ESM.zip › InputFiles&OutputTrees/BayesCalibrated/Diversity(NoSA)/BayesCal_IGR_ln_p1_60G_DvNoSA_SFBD(s)2_2l/BayesCal_IGR_ln_p1_DvNoSA_SFBD%28s%292_2l_AllCom.t.con.tre.pdf]

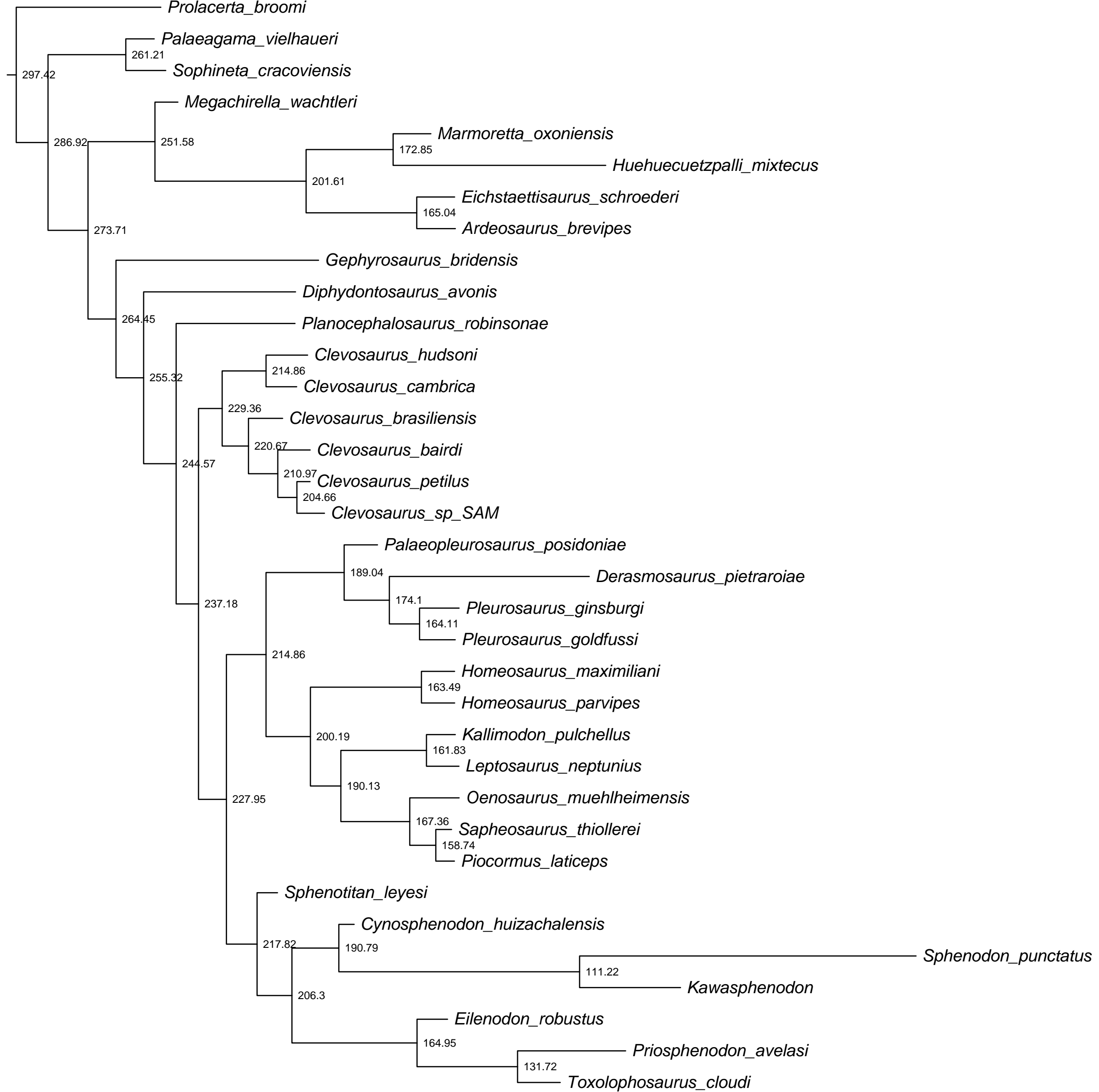

30.0

Supplement: Supplementary file 6 — Additional file 6. Input files including the dataset and all necessary coding (see Mr. Bayes blocks) to reproduce the analyses. [file 12915_2020_901_MOESM6_ESM.zip › InputFiles&OutputTrees/BayesCalibrated/Diversity(NoSA)/BayesCal_IGR_ln_p1_60G_DvNoSA_SFBD(s)2_2l/BayesCal_IGR_ln_p1_DvNoSA_SFBD%28s%292_2l_AllCom.t.con.tre_Age.pdf]

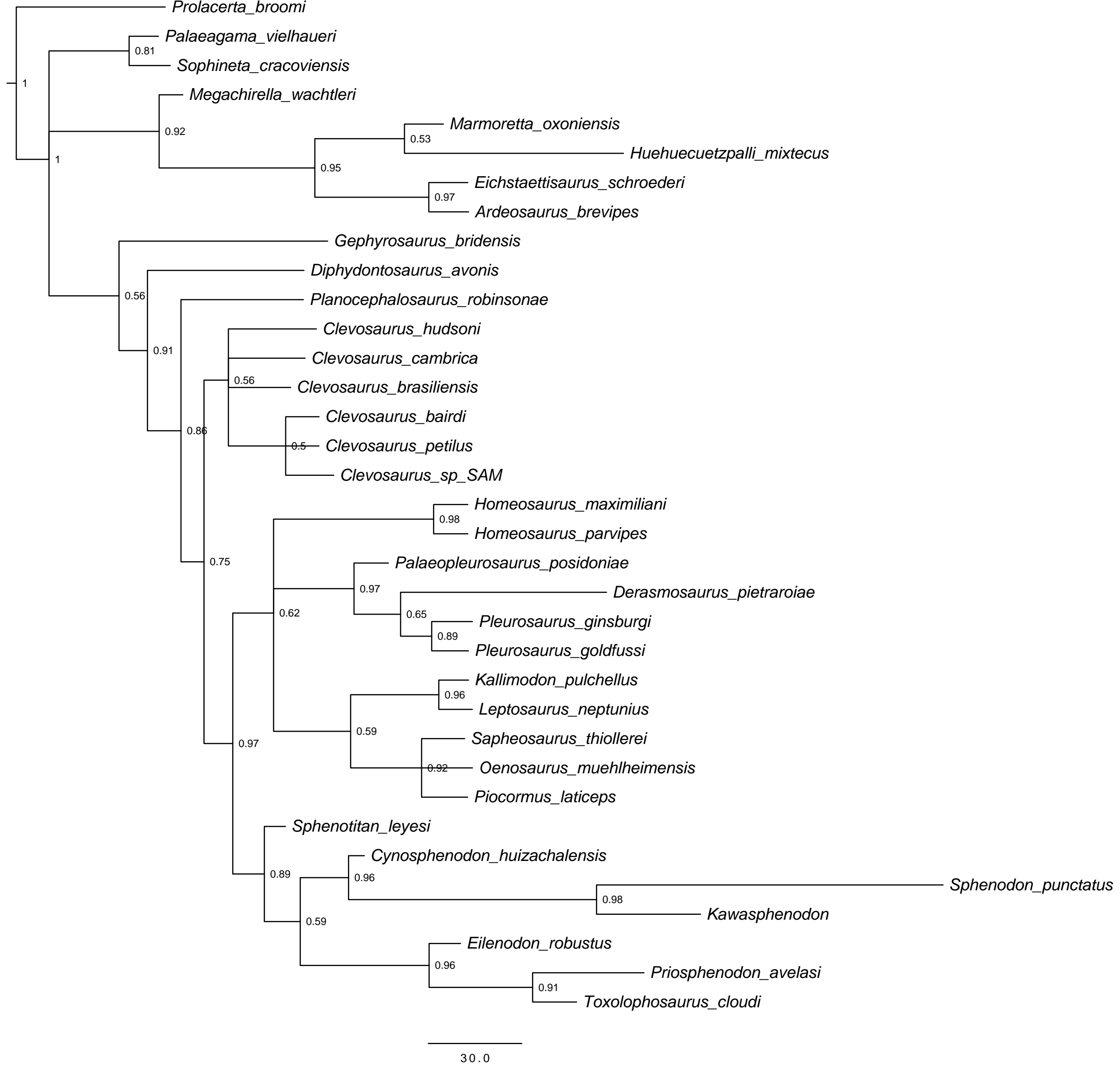

Supplement: Supplementary file 6 — Additional file 6. Input files including the dataset and all necessary coding (see Mr. Bayes blocks) to reproduce the analyses. [file 12915_2020_901_MOESM6_ESM.zip › InputFiles&OutputTrees/BayesCalibrated/Diversity(NoSA)/BayesCal_IGR_ln_p1_60G_DvNoSA_SFBD(s)2_2l/BayesCal_IGR_ln_p1_DvNoSA_SFBD%28s%292_2l_MRC.t.con.tre.pdf]

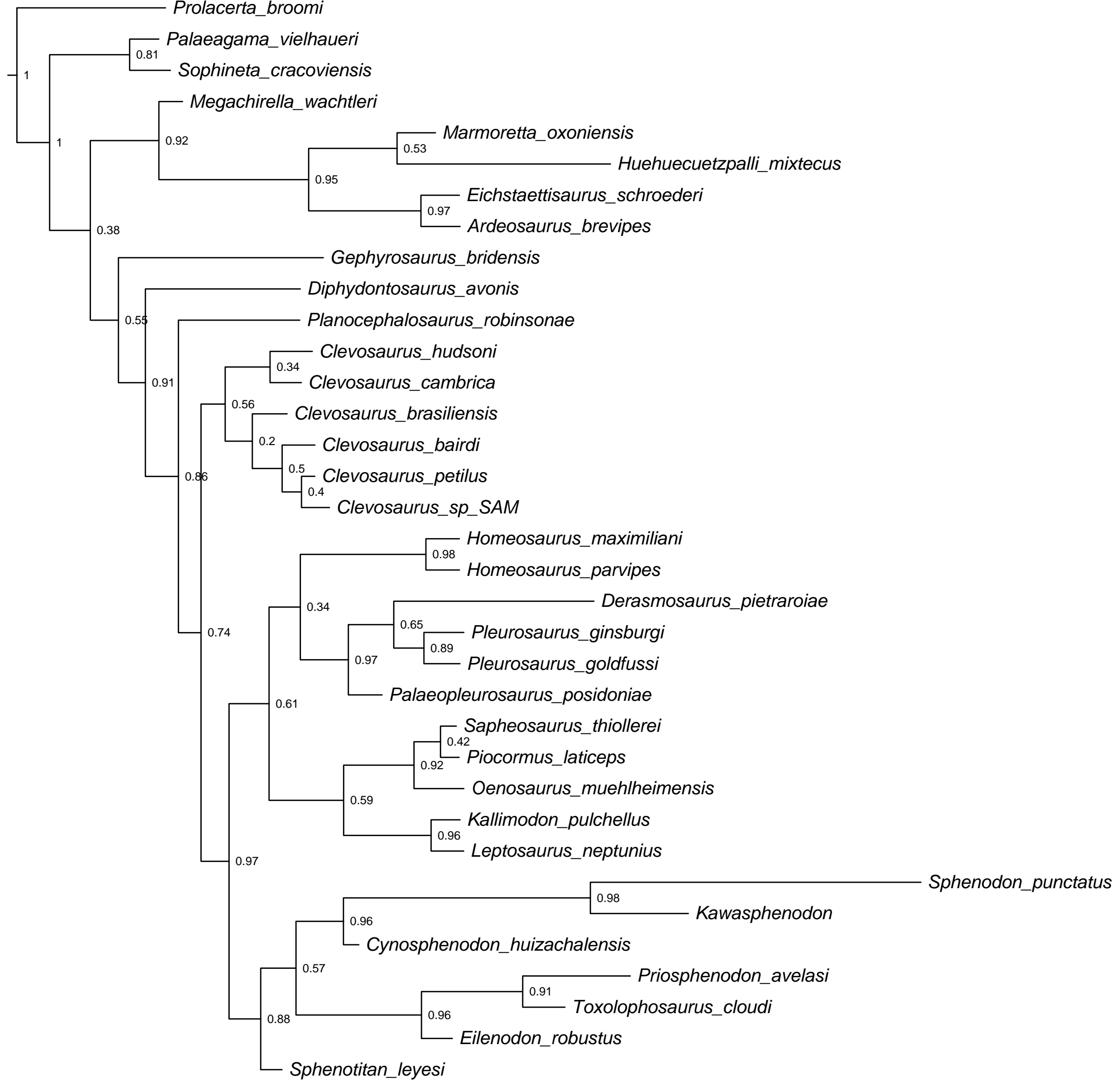

Supplement: Supplementary file 6 — Additional file 6. Input files including the dataset and all necessary coding (see Mr. Bayes blocks) to reproduce the analyses. [file 12915_2020_901_MOESM6_ESM.zip › InputFiles&OutputTrees/BayesCalibrated/Diversity(NoSA)/BayesCal_IGR_ln_p1_60G_DvNoSA_SFBD(s)2_3l/BayesCal_IGR_ln_p1_DvNoSA_SFBD%28s%292_3l_AllCom.t.con.tre.pdf]

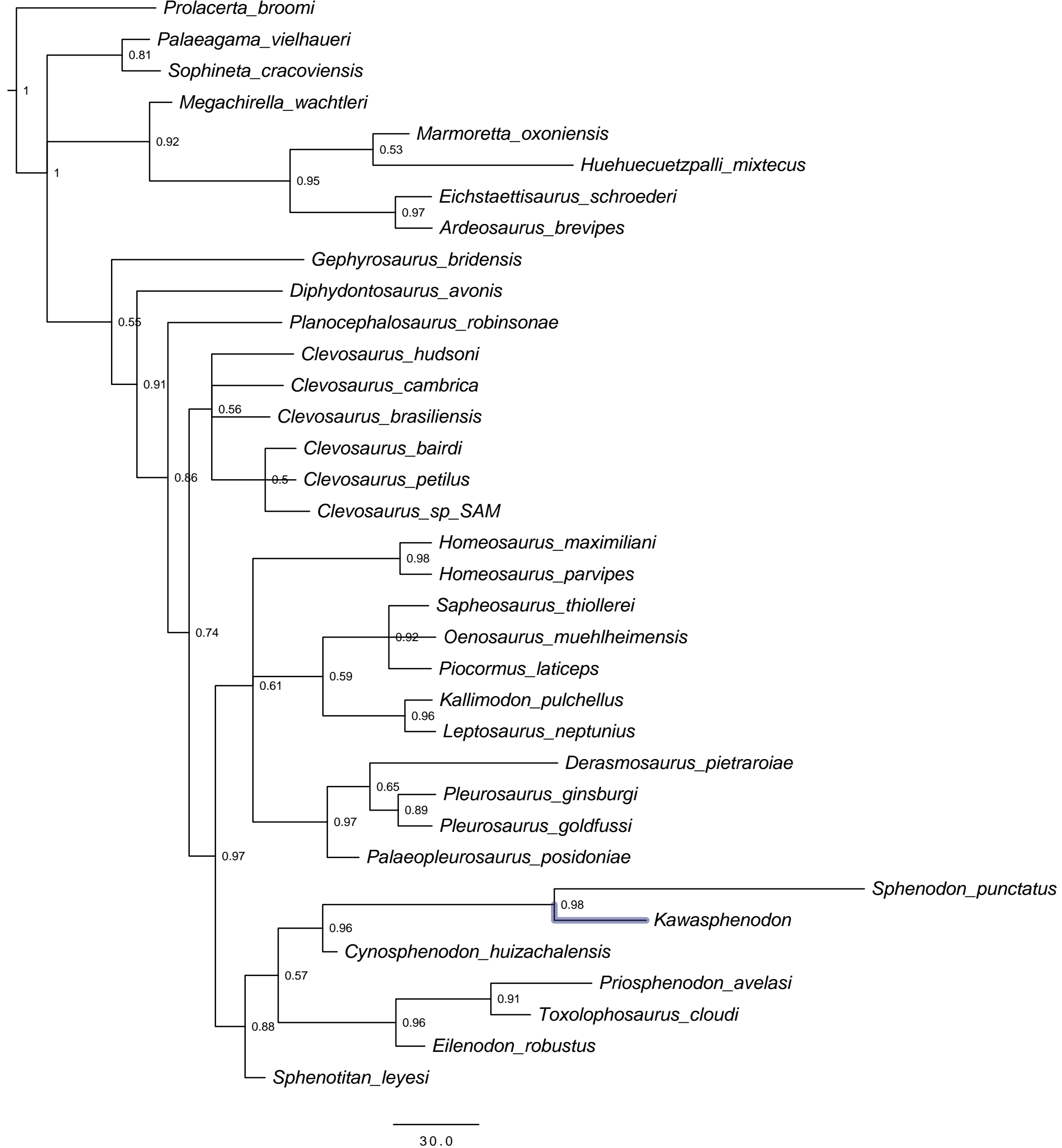

Supplement: Supplementary file 6 — Additional file 6. Input files including the dataset and all necessary coding (see Mr. Bayes blocks) to reproduce the analyses. [file 12915_2020_901_MOESM6_ESM.zip › InputFiles&OutputTrees/BayesCalibrated/Diversity(NoSA)/BayesCal_IGR_ln_p1_60G_DvNoSA_SFBD(s)2_3l/BayesCal_IGR_ln_p1_DvNoSA_SFBD%28s%292_3l_MRC.t.con.tre.pdf]

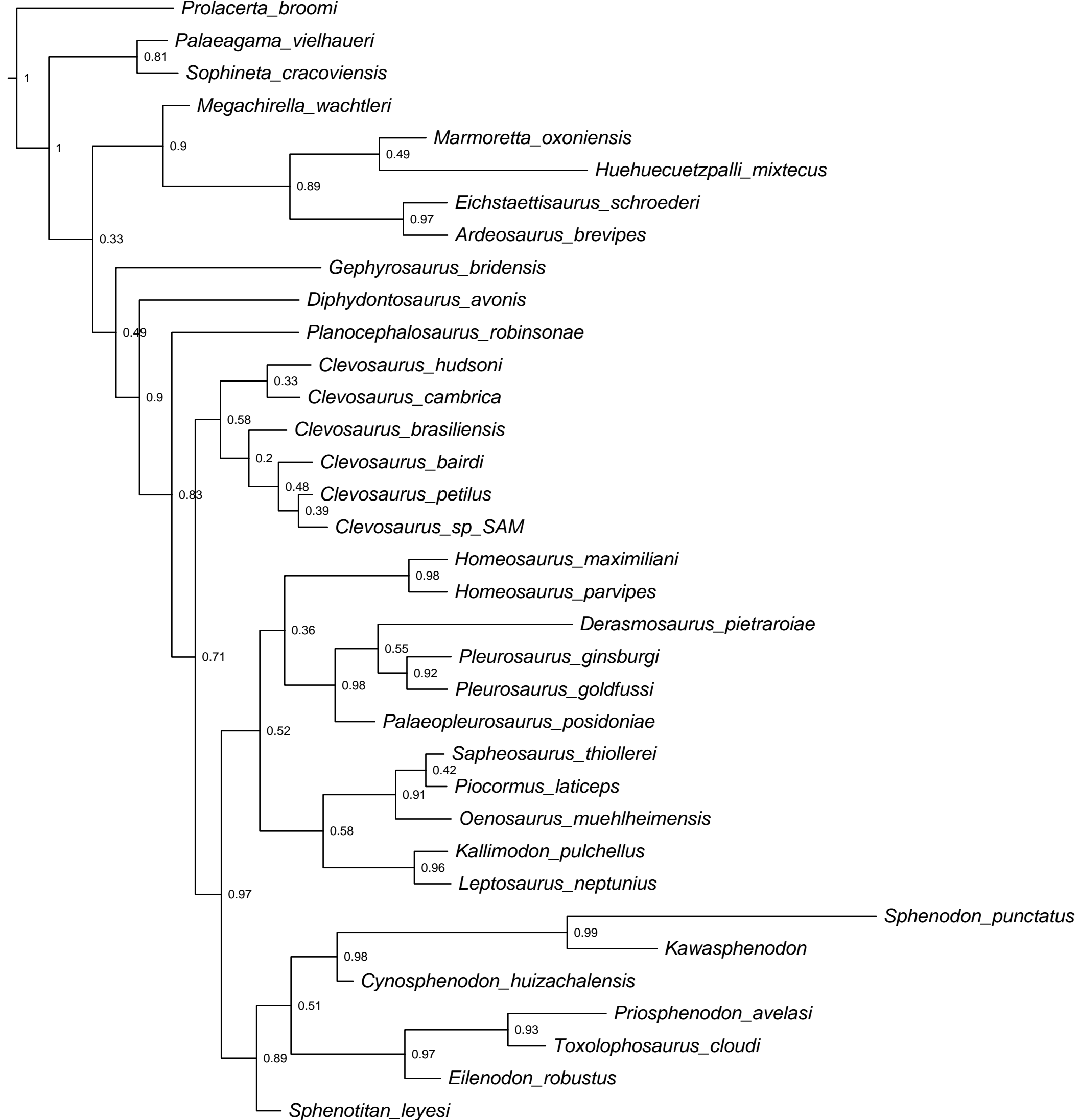

Supplement: Supplementary file 6 — Additional file 6. Input files including the dataset and all necessary coding (see Mr. Bayes blocks) to reproduce the analyses. [file 12915_2020_901_MOESM6_ESM.zip › InputFiles&OutputTrees/BayesCalibrated/Diversity(NoSA)/BayesCal_IGR_ln_p1_60G_DvNoSA_SFBD(sdr)2_2l/BayesCal_IGR_ln_p1_DvNoSA_SFBD%28sdr%292_2l_AllCom.t.con.tre.pdf]

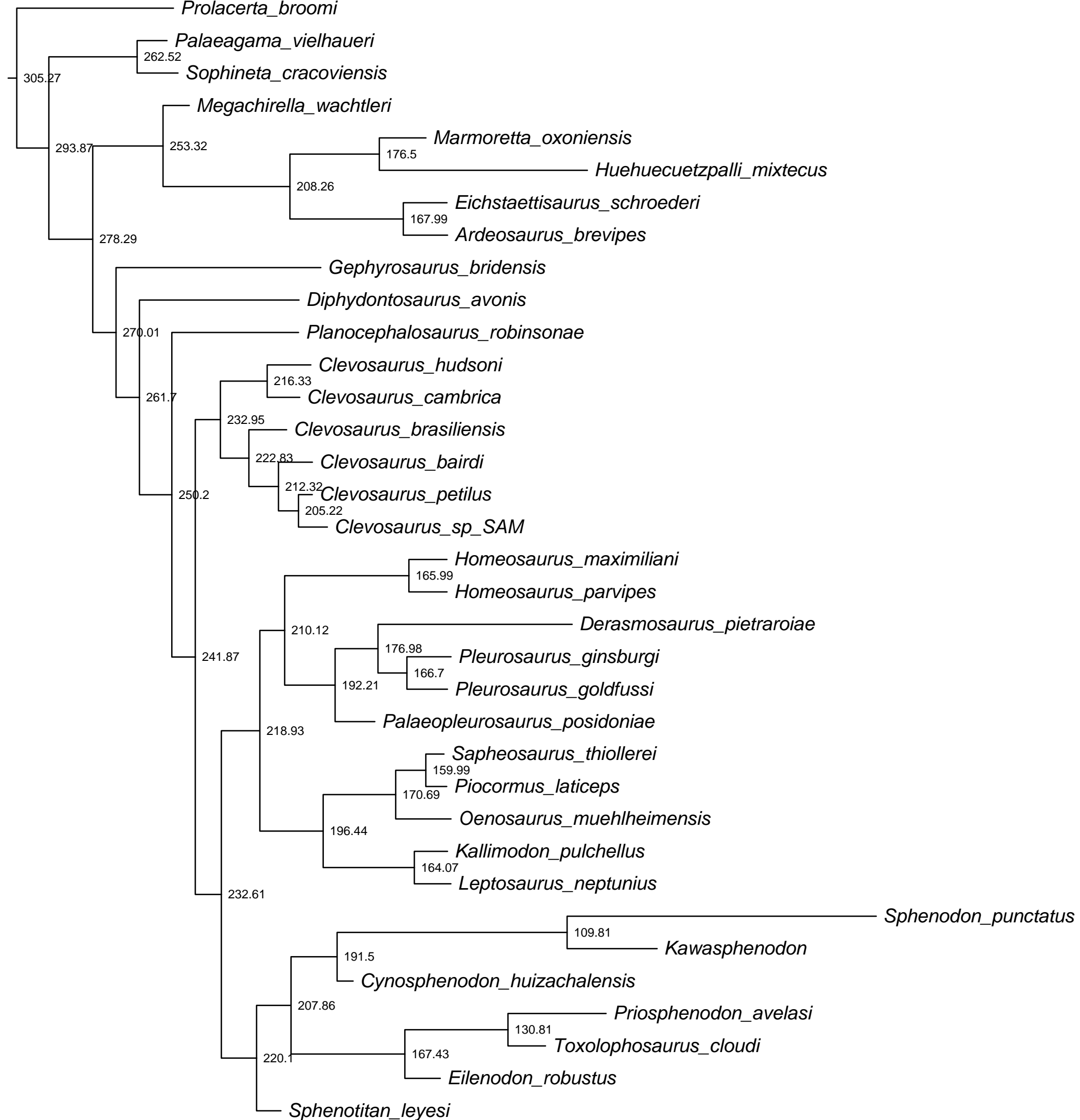

Supplement: Supplementary file 6 — Additional file 6. Input files including the dataset and all necessary coding (see Mr. Bayes blocks) to reproduce the analyses. [file 12915_2020_901_MOESM6_ESM.zip › InputFiles&OutputTrees/BayesCalibrated/Diversity(NoSA)/BayesCal_IGR_ln_p1_60G_DvNoSA_SFBD(sdr)2_2l/BayesCal_IGR_ln_p1_DvNoSA_SFBD%28sdr%292_2l_AllCom.t.con.tre_Age.pdf]

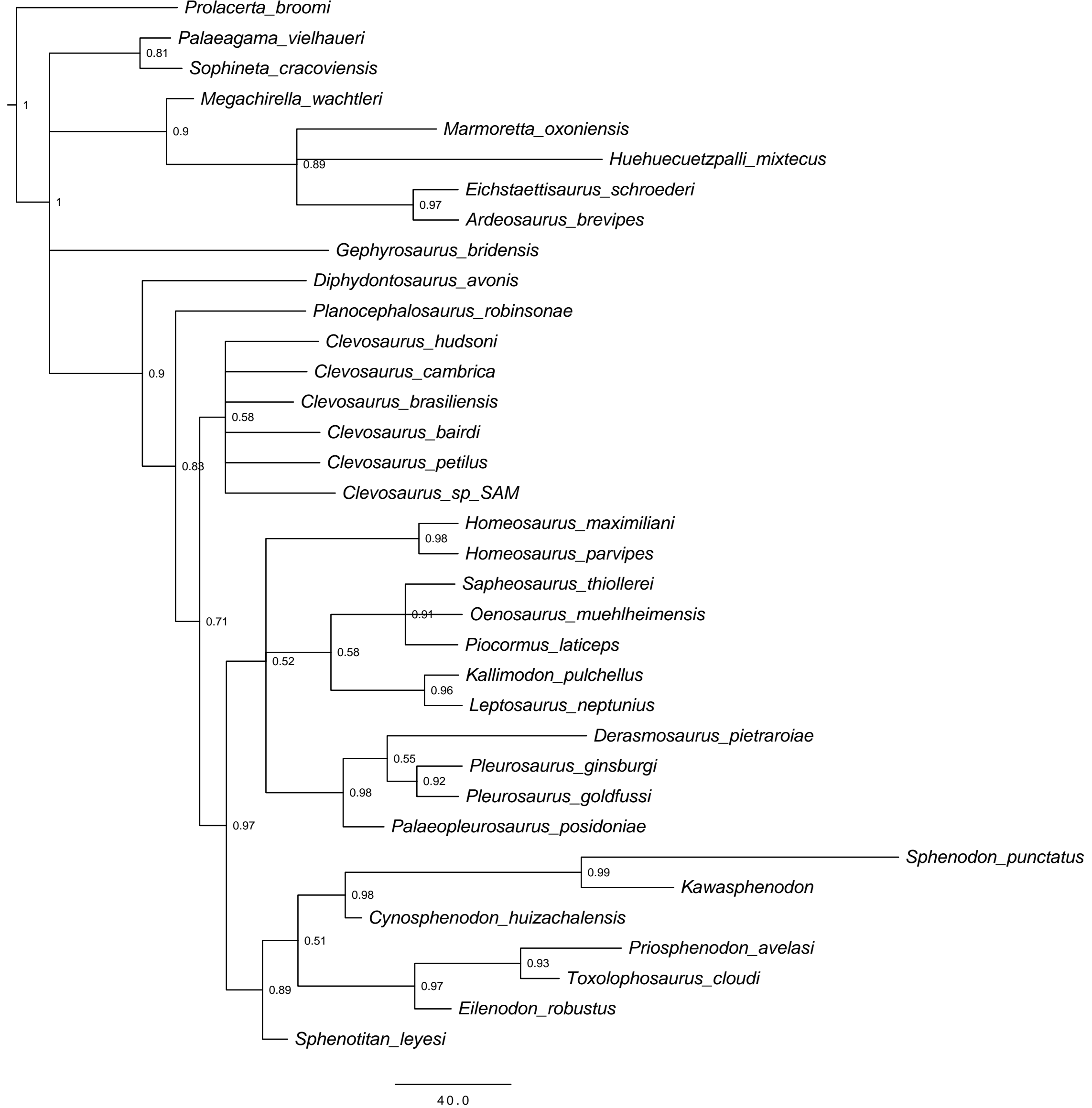

Supplement: Supplementary file 6 — Additional file 6. Input files including the dataset and all necessary coding (see Mr. Bayes blocks) to reproduce the analyses. [file 12915_2020_901_MOESM6_ESM.zip › InputFiles&OutputTrees/BayesCalibrated/Diversity(NoSA)/BayesCal_IGR_ln_p1_60G_DvNoSA_SFBD(sdr)2_2l/BayesCal_IGR_ln_p1_DvNoSA_SFBD%28sdr%292_2l_MRC.t.con.tre.pdf]

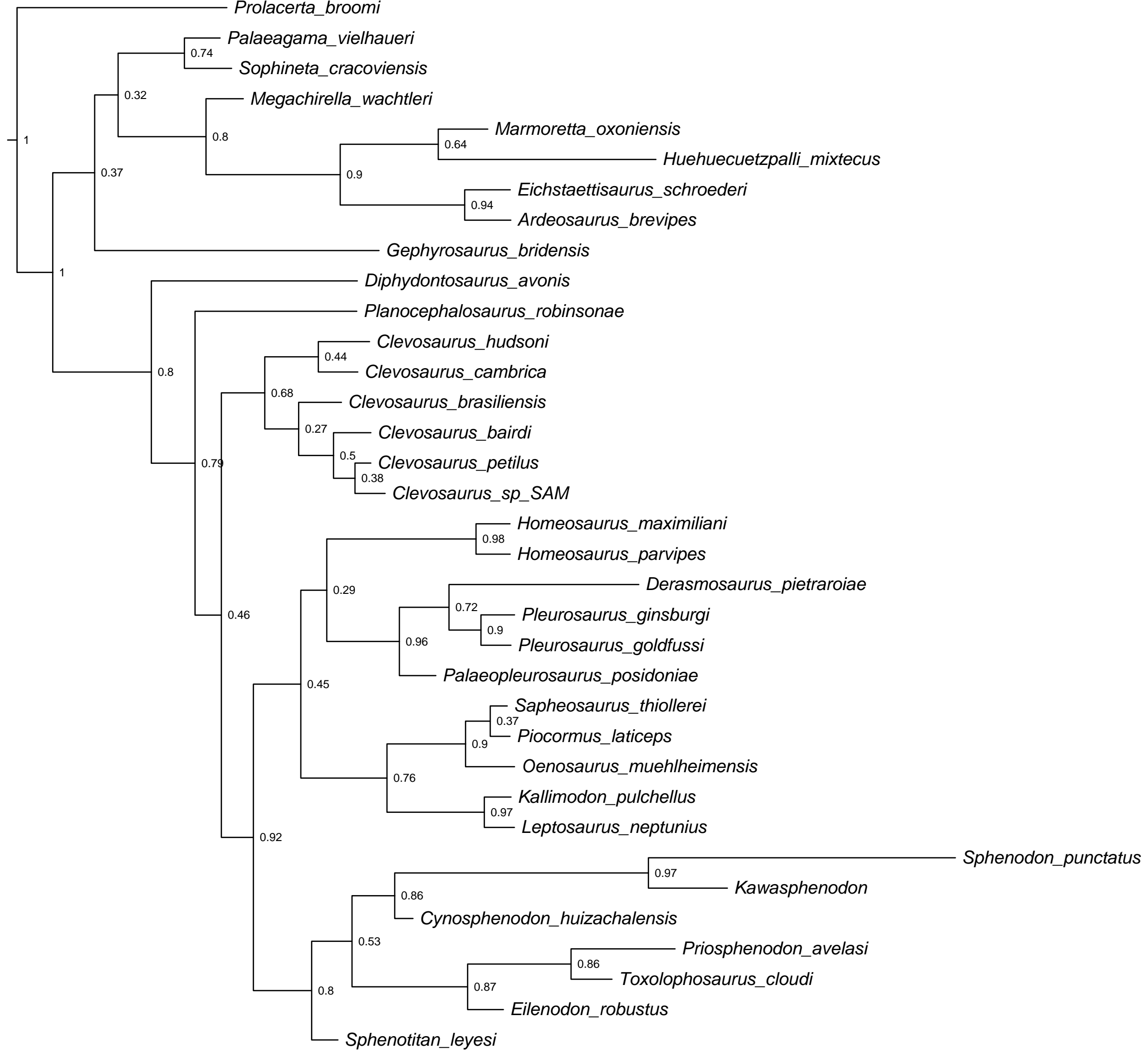

40.0

Supplement: Supplementary file 6 — Additional file 6. Input files including the dataset and all necessary coding (see Mr. Bayes blocks) to reproduce the analyses. [file 12915_2020_901_MOESM6_ESM.zip › InputFiles&OutputTrees/BayesCalibrated/Diversity(NoSA)/BayesCal_IGR_ln_p3_60G_DvNoSA/BayesCal_IGR_ln_p3_DvNoSA_AllCom.t.con.tre.pdf]

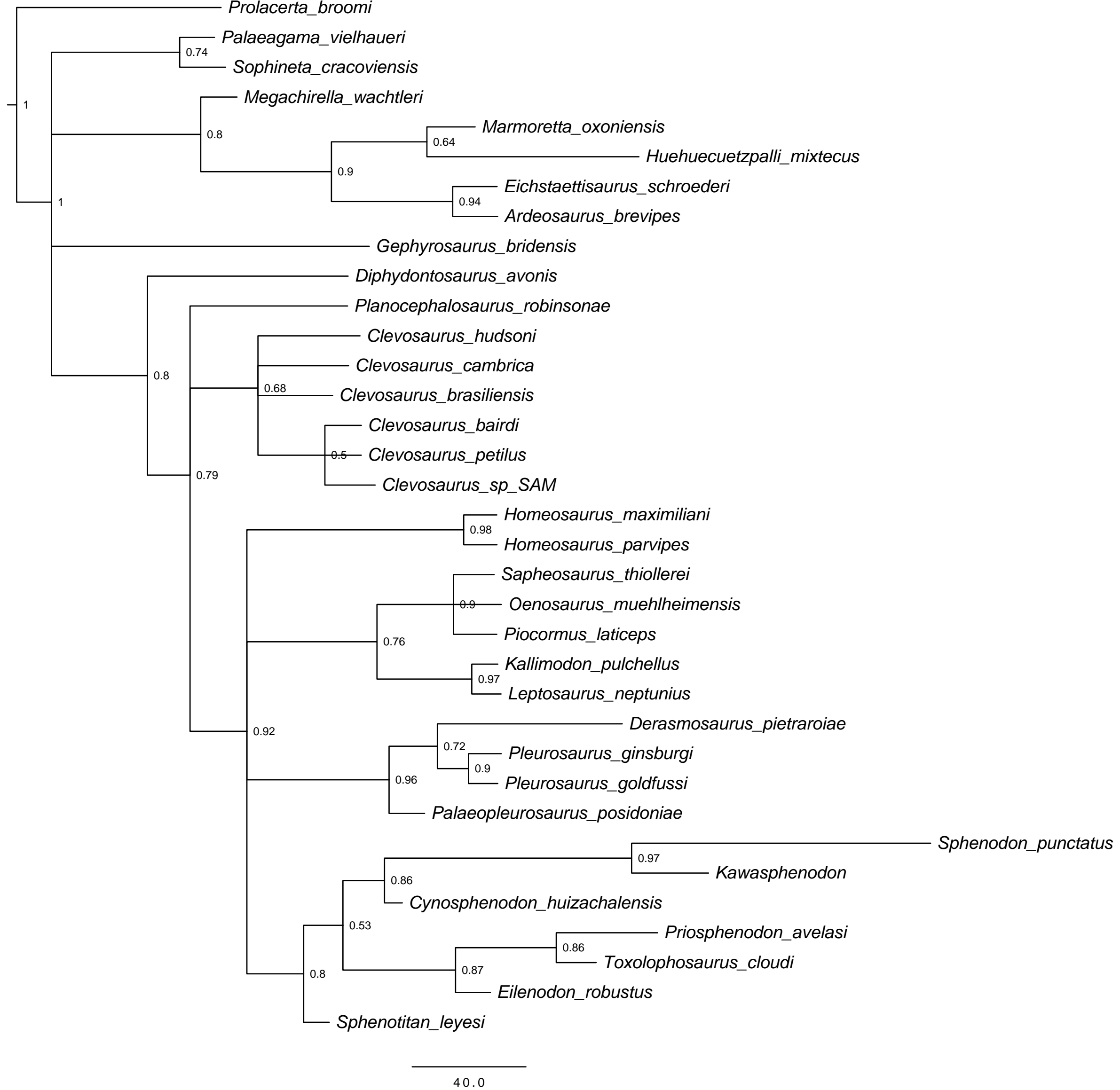

Supplement: Supplementary file 6 — Additional file 6. Input files including the dataset and all necessary coding (see Mr. Bayes blocks) to reproduce the analyses. [file 12915_2020_901_MOESM6_ESM.zip › InputFiles&OutputTrees/BayesCalibrated/Diversity(NoSA)/BayesCal_IGR_ln_p3_60G_DvNoSA/BayesCal_IGR_ln_p3_DvNoSA_MRC.t.con.tre.pdf]

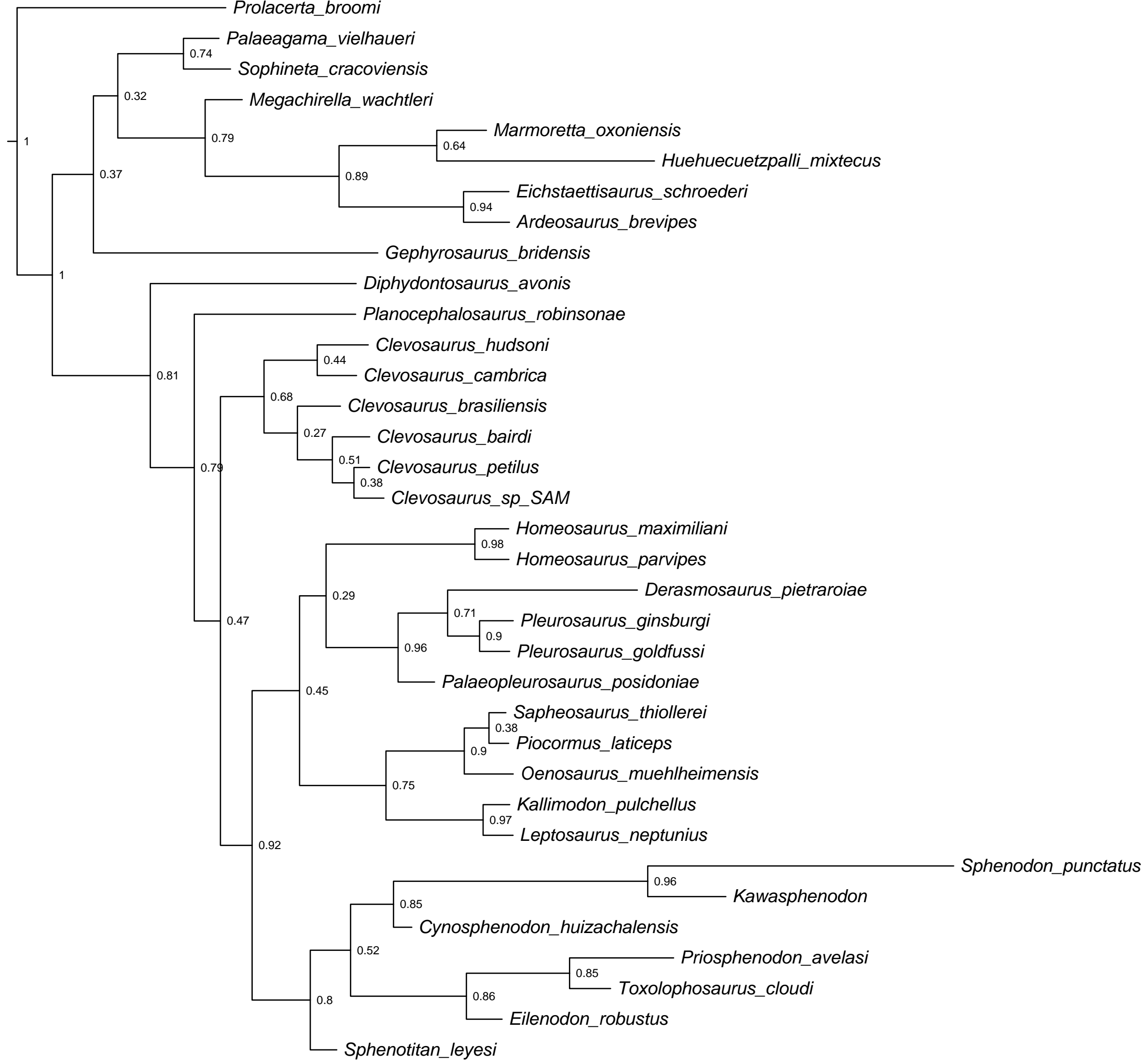

40.0

Supplement: Supplementary file 6 — Additional file 6. Input files including the dataset and all necessary coding (see Mr. Bayes blocks) to reproduce the analyses. [file 12915_2020_901_MOESM6_ESM.zip › InputFiles&OutputTrees/BayesCalibrated/Diversity(NoSA)/BayesCal_IGR_ln_p3_StartTr_3per_60G_DvNoSA/BayesCal_IGR_ln_p3_StartTR_3per_DvNoSA_AllCom.t.con.tre.pdf]

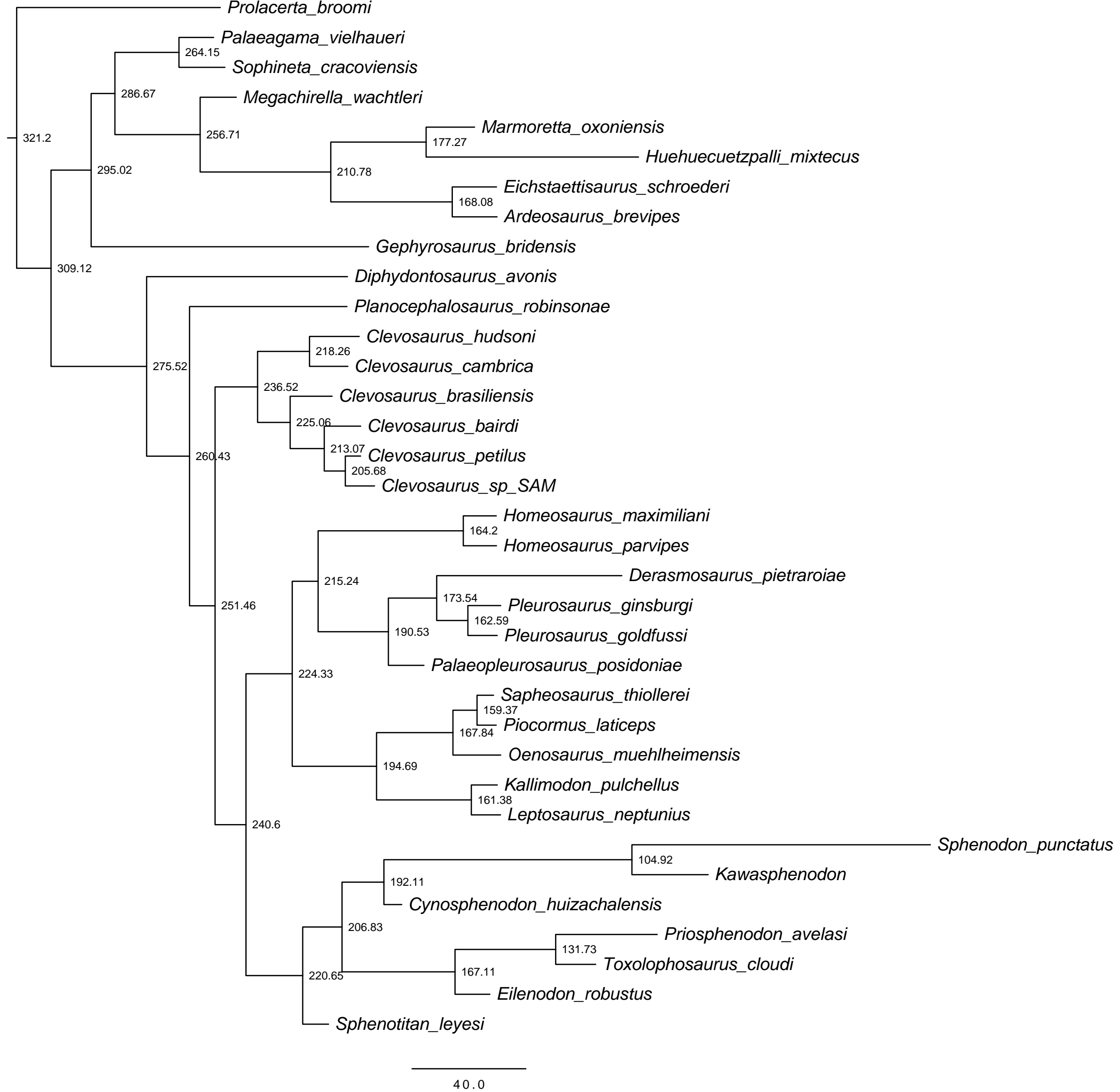

Supplement: Supplementary file 6 — Additional file 6. Input files including the dataset and all necessary coding (see Mr. Bayes blocks) to reproduce the analyses. [file 12915_2020_901_MOESM6_ESM.zip › InputFiles&OutputTrees/BayesCalibrated/Diversity(NoSA)/BayesCal_IGR_ln_p3_StartTr_3per_60G_DvNoSA/BayesCal_IGR_ln_p3_StartTR_3per_DvNoSA_AllCom.t.con.tre_Age.pdf]

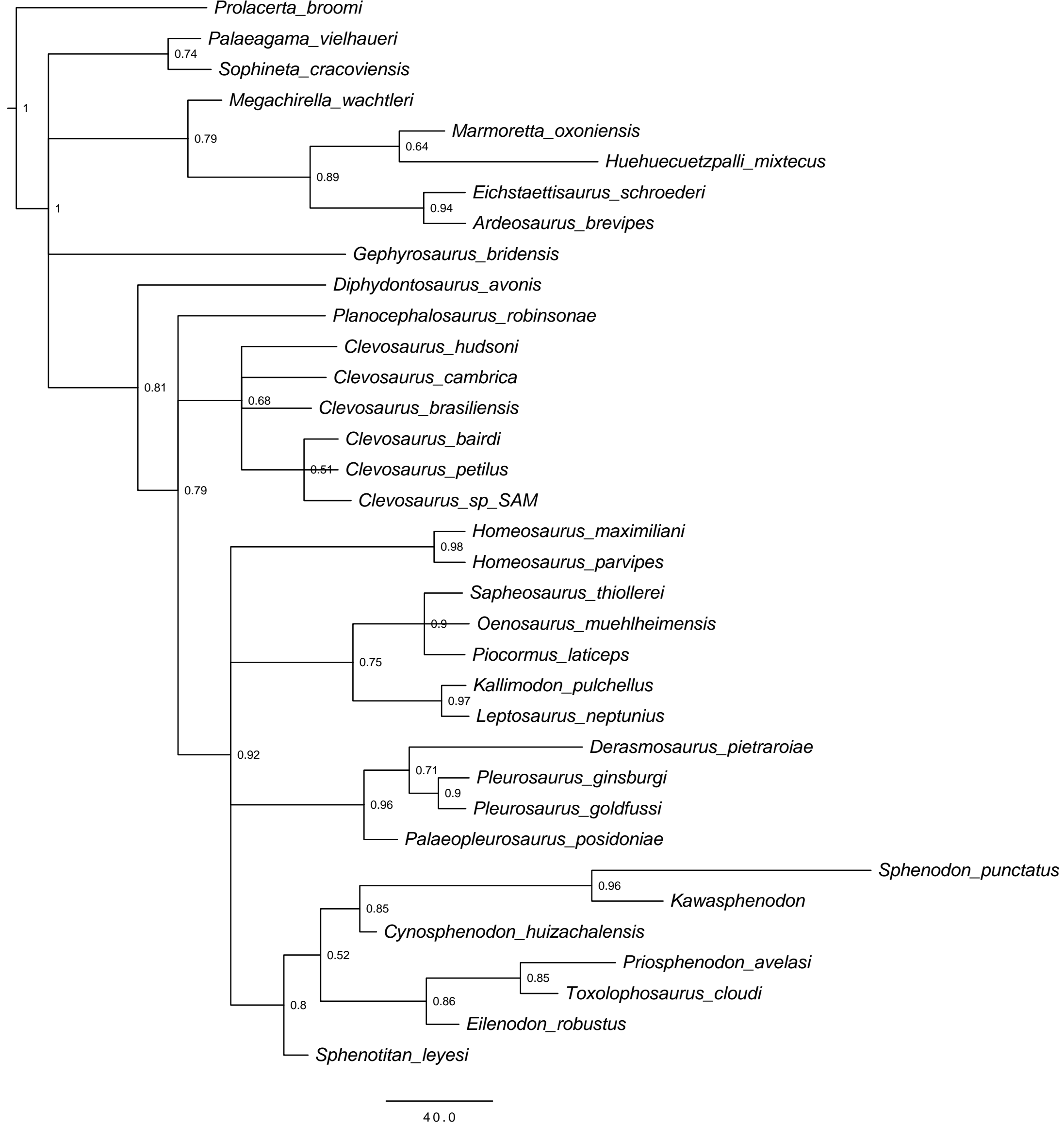

Supplement: Supplementary file 6 — Additional file 6. Input files including the dataset and all necessary coding (see Mr. Bayes blocks) to reproduce the analyses. [file 12915_2020_901_MOESM6_ESM.zip › InputFiles&OutputTrees/BayesCalibrated/Diversity(NoSA)/BayesCal_IGR_ln_p3_StartTr_3per_60G_DvNoSA/BayesCal_IGR_ln_p3_StartTR_3per_DvNoSA_MRC.t.con.tre.pdf]

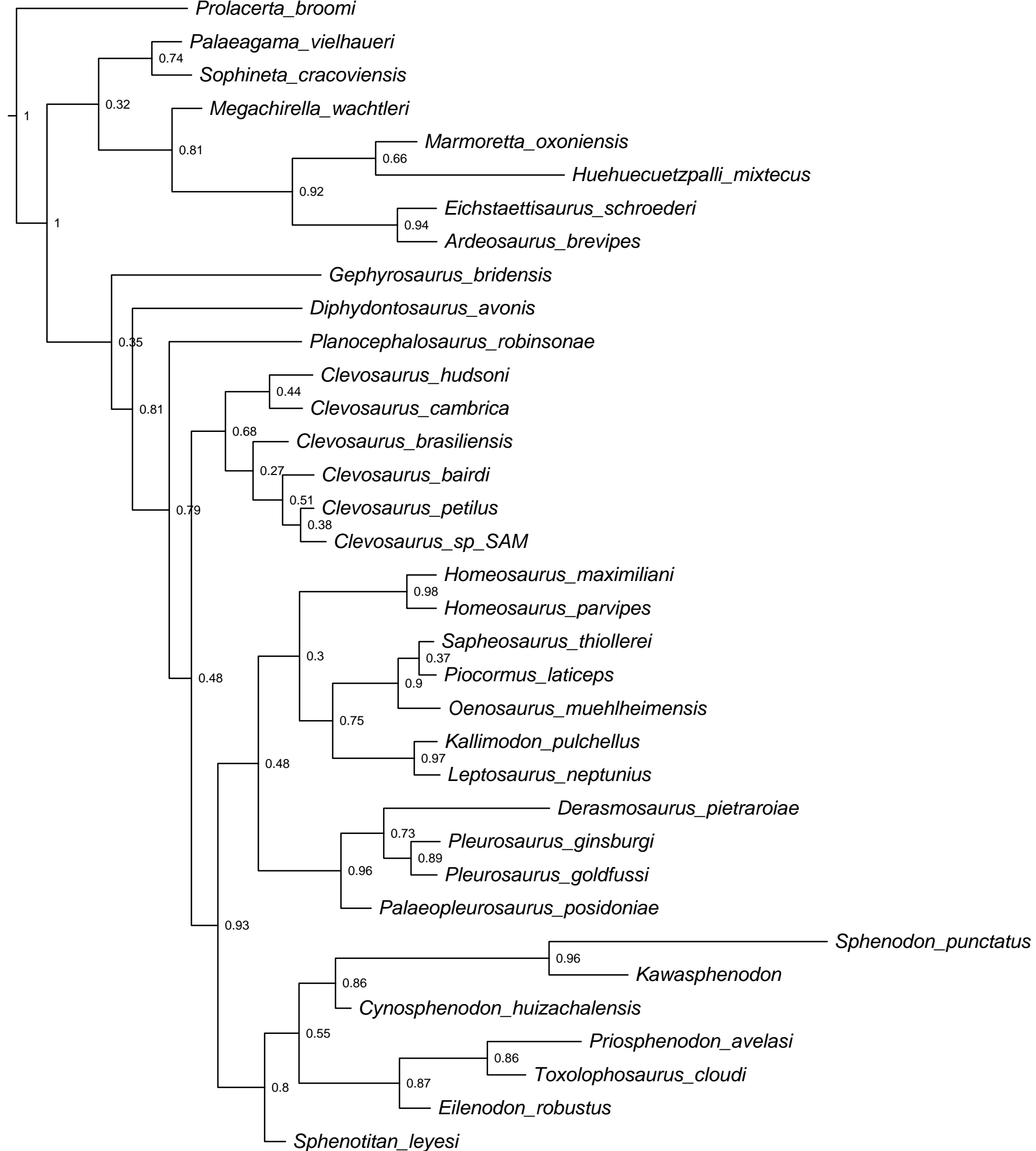

Supplement: Supplementary file 6 — Additional file 6. Input files including the dataset and all necessary coding (see Mr. Bayes blocks) to reproduce the analyses. [file 12915_2020_901_MOESM6_ESM.zip › InputFiles&OutputTrees/BayesCalibrated/Diversity(NoSA)/BayesCal_IGR_ln_p3_StartTr_3per_60G_DvNoSA_SFBD(s)2_2l/BayesCal_IGR_ln_p3_StartTR_3per_DvNoSA_SFBD2_AllCom.t.con.tre.pdf]

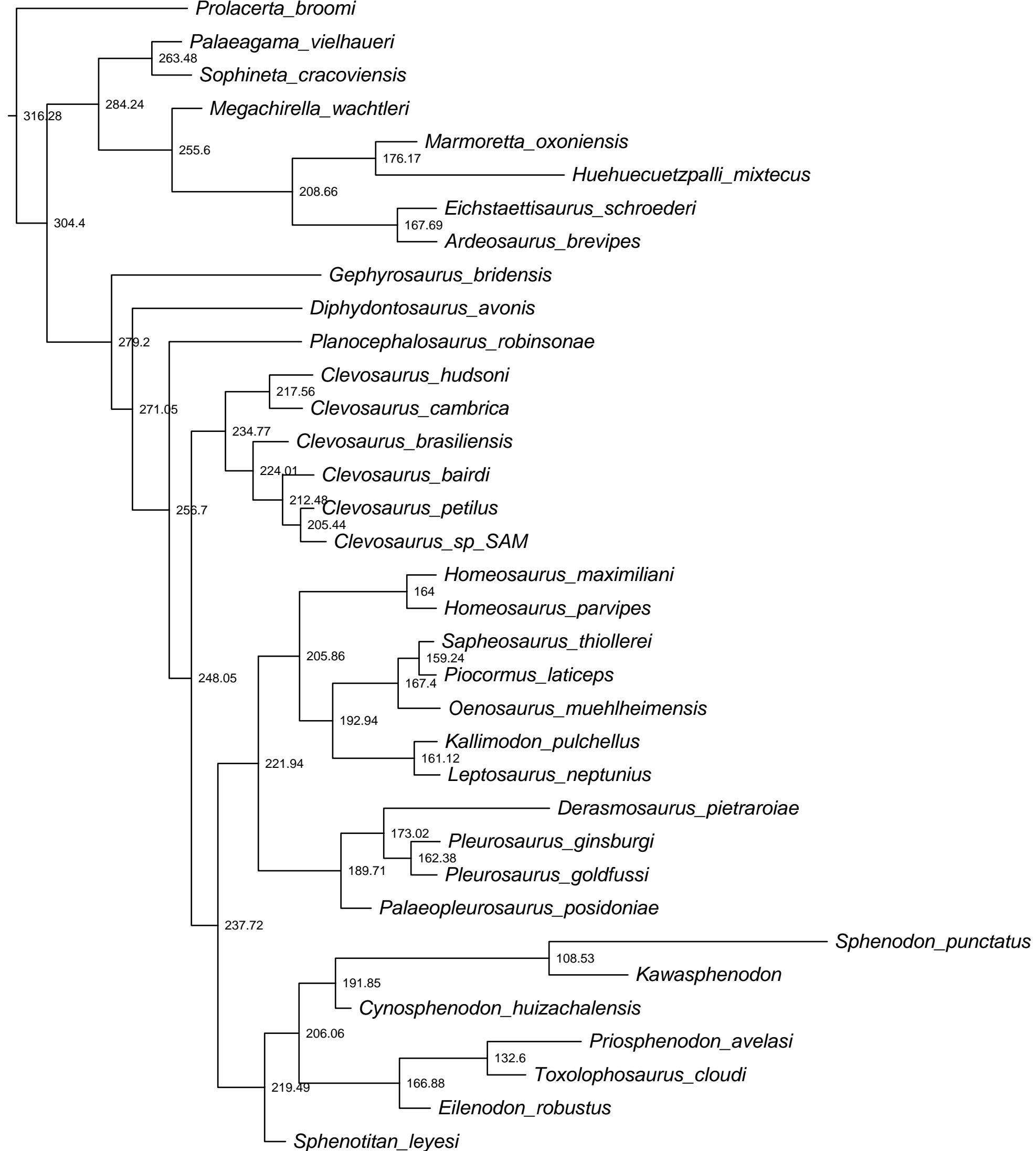

Supplement: Supplementary file 6 — Additional file 6. Input files including the dataset and all necessary coding (see Mr. Bayes blocks) to reproduce the analyses. [file 12915_2020_901_MOESM6_ESM.zip › InputFiles&OutputTrees/BayesCalibrated/Diversity(NoSA)/BayesCal_IGR_ln_p3_StartTr_3per_60G_DvNoSA_SFBD(s)2_2l/BayesCal_IGR_ln_p3_StartTR_3per_DvNoSA_SFBD2_AllCom.t.con.tre_Age.pdf]

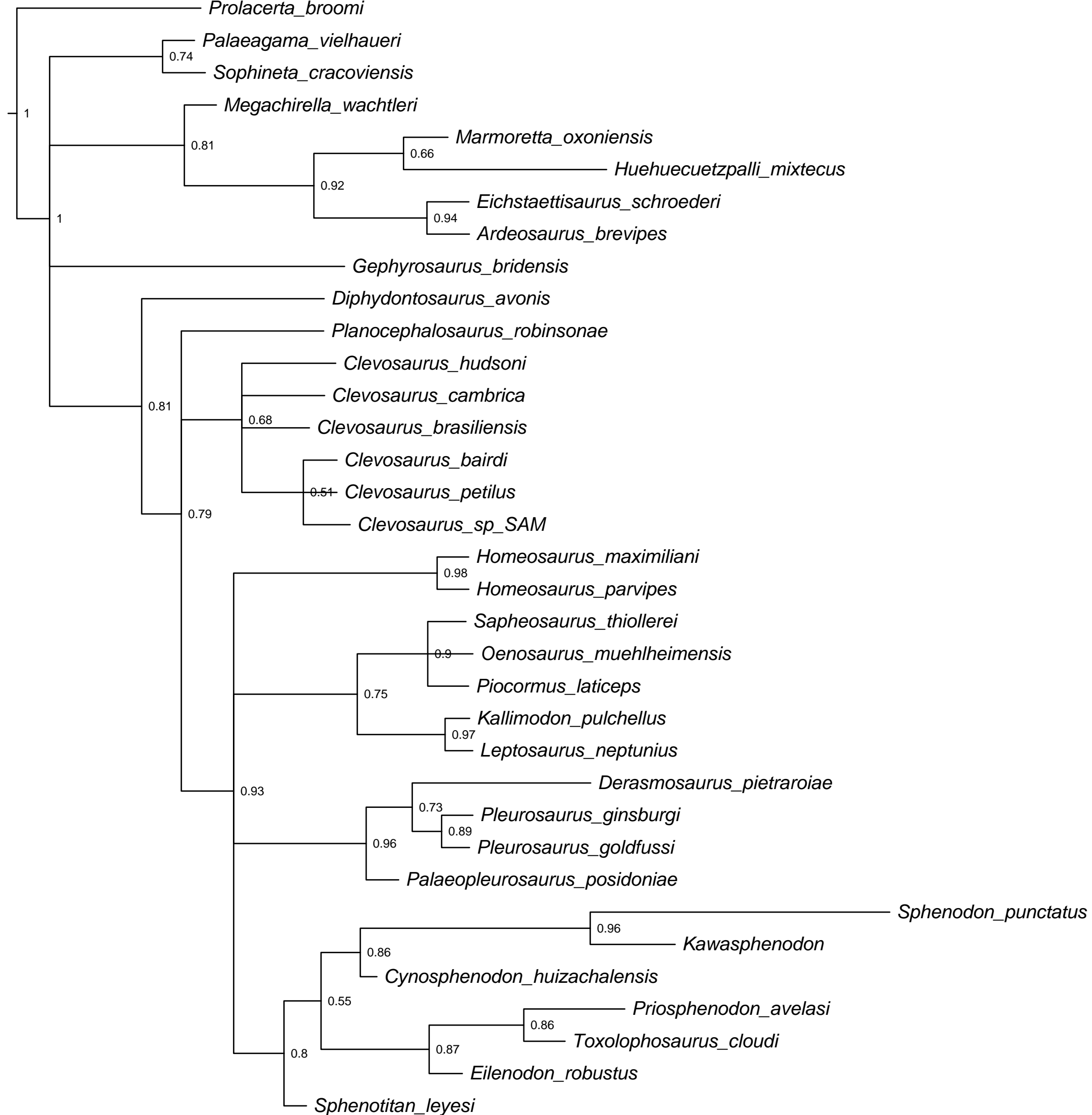

Supplement: Supplementary file 6 — Additional file 6. Input files including the dataset and all necessary coding (see Mr. Bayes blocks) to reproduce the analyses. [file 12915_2020_901_MOESM6_ESM.zip › InputFiles&OutputTrees/BayesCalibrated/Diversity(NoSA)/BayesCal_IGR_ln_p3_StartTr_3per_60G_DvNoSA_SFBD(s)2_2l/BayesCal_IGR_ln_p3_StartTR_3per_DvNoSA_SFBD2_MRC.t.con.tre.pdf]

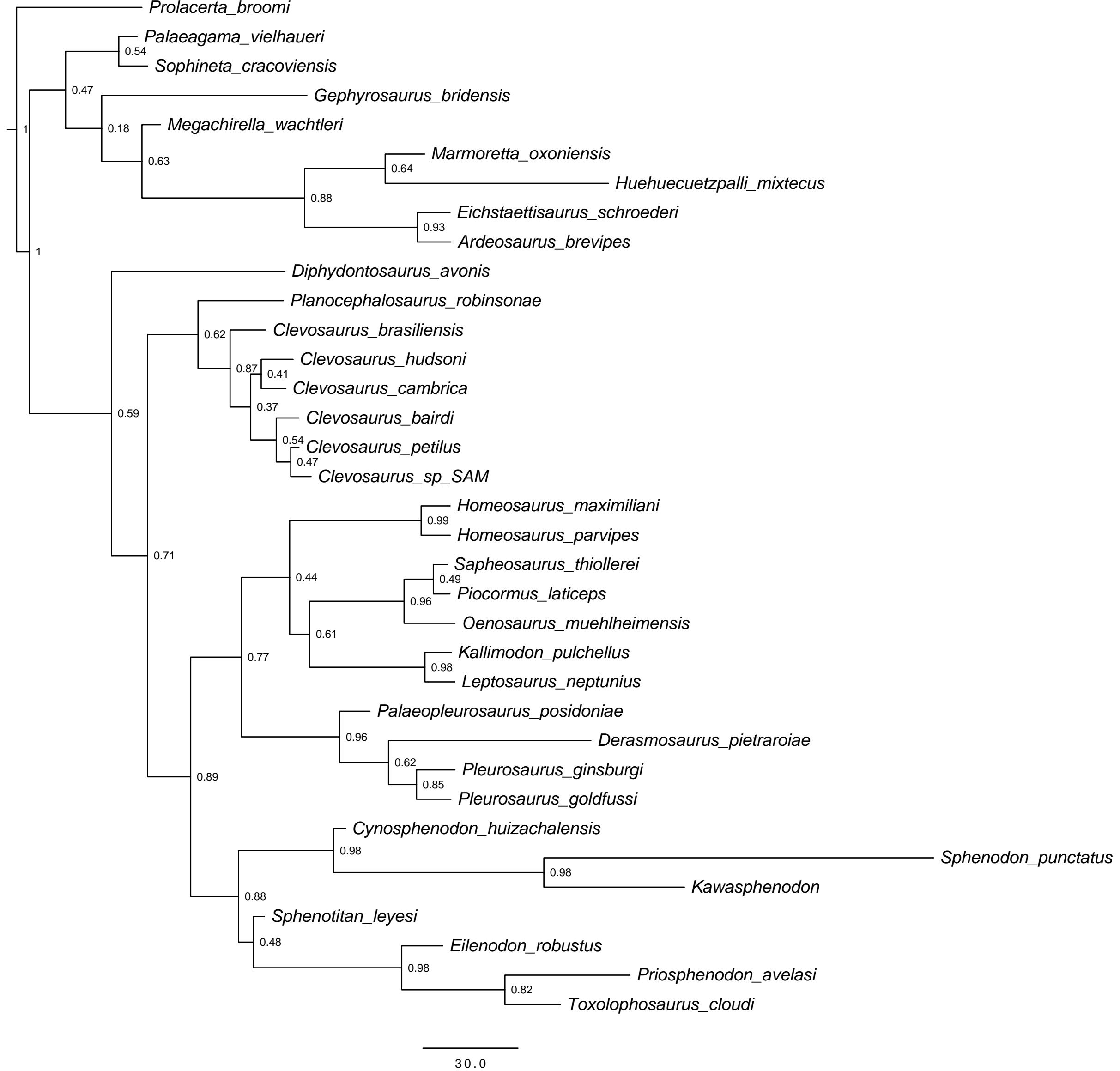

Supplement: Supplementary file 6 — Additional file 6. Input files including the dataset and all necessary coding (see Mr. Bayes blocks) to reproduce the analyses. [file 12915_2020_901_MOESM6_ESM.zip › InputFiles&OutputTrees/BayesCalibrated/Diversity(NoSA)/BayesCal_TK02_ln_p1_60G_DvNoSA/BayesCal_TK02_ln_p1_DvNoSA_AllCom.t.con.tre.pdf]

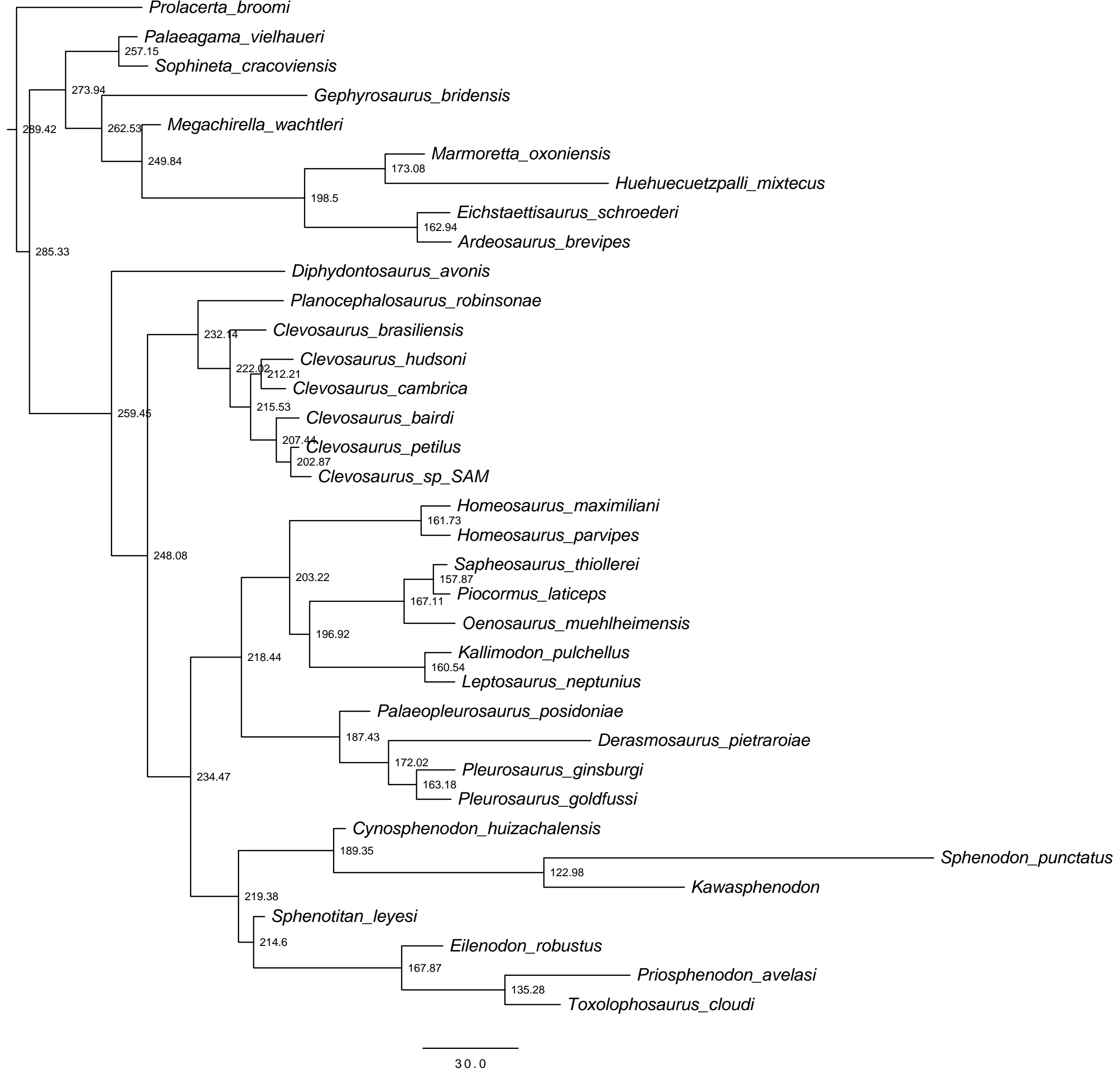

Supplement: Supplementary file 6 — Additional file 6. Input files including the dataset and all necessary coding (see Mr. Bayes blocks) to reproduce the analyses. [file 12915_2020_901_MOESM6_ESM.zip › InputFiles&OutputTrees/BayesCalibrated/Diversity(NoSA)/BayesCal_TK02_ln_p1_60G_DvNoSA/BayesCal_TK02_ln_p1_DvNoSA_AllCom.t.con.tre_Age.pdf]

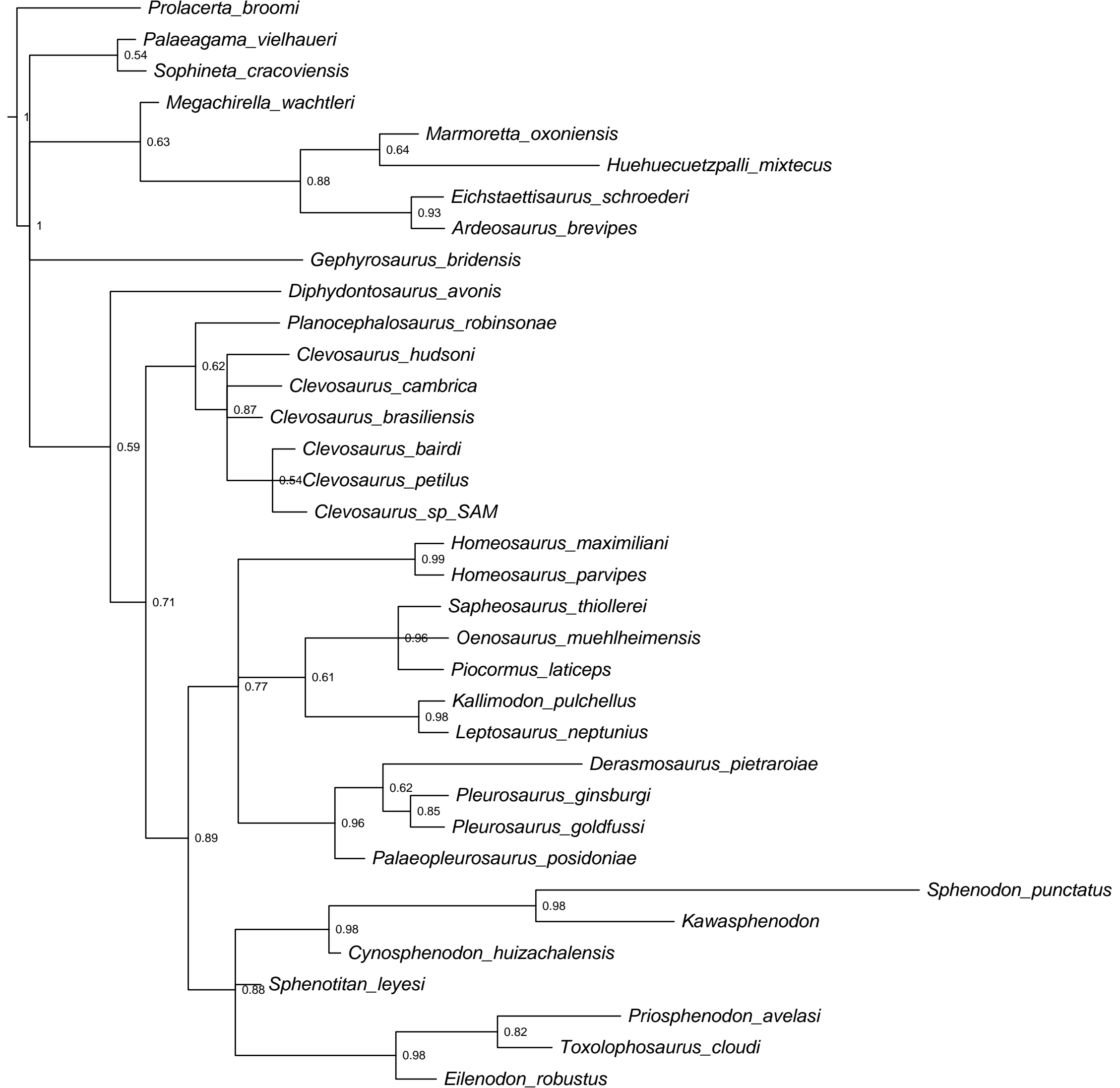

Supplement: Supplementary file 6 — Additional file 6. Input files including the dataset and all necessary coding (see Mr. Bayes blocks) to reproduce the analyses. [file 12915_2020_901_MOESM6_ESM.zip › InputFiles&OutputTrees/BayesCalibrated/Diversity(NoSA)/BayesCal_TK02_ln_p1_60G_DvNoSA/BayesCal_TK02_ln_p1_DvNoSA_MRC.t.con.tre.pdf]

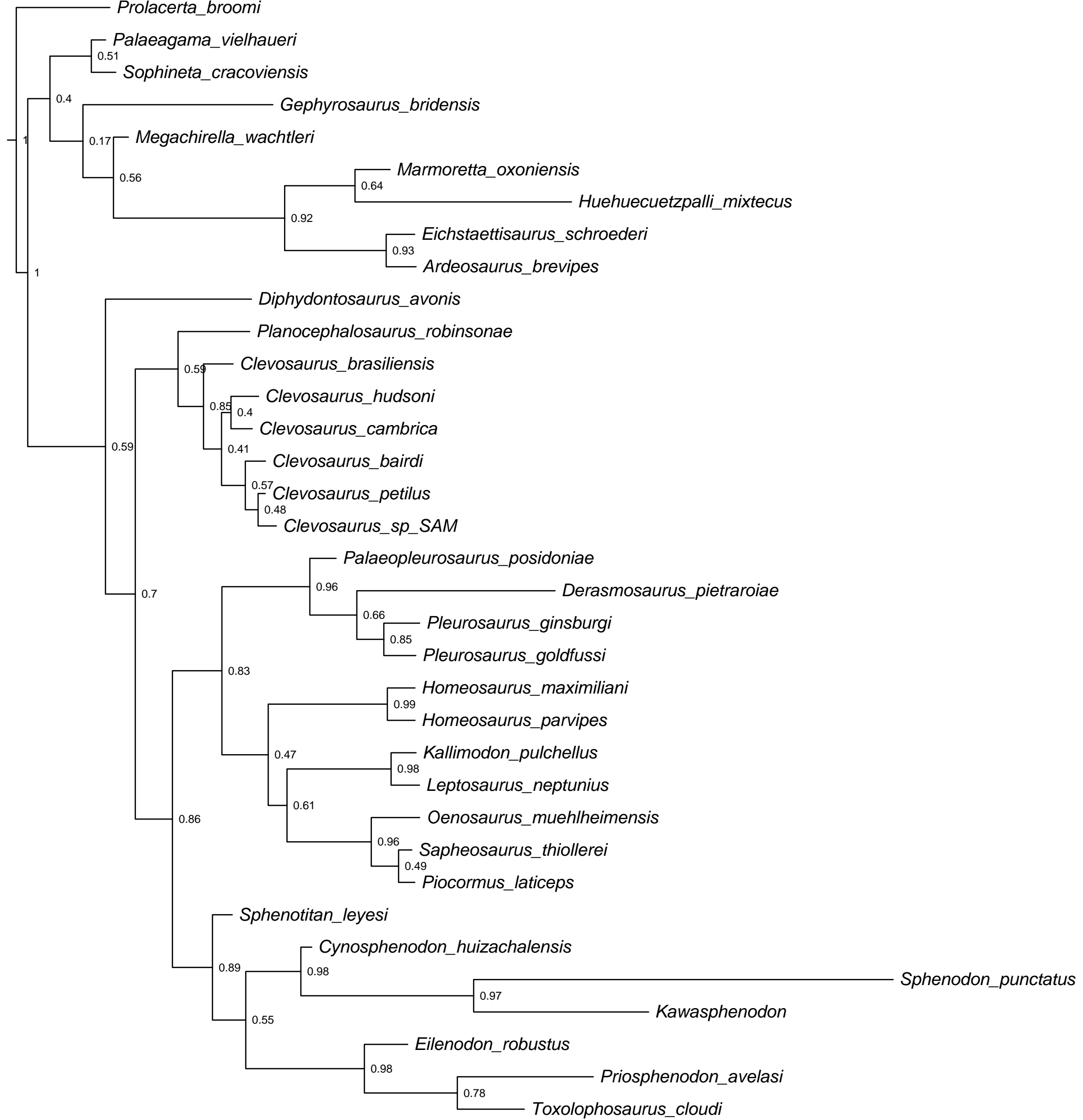

Supplement: Supplementary file 6 — Additional file 6. Input files including the dataset and all necessary coding (see Mr. Bayes blocks) to reproduce the analyses. [file 12915_2020_901_MOESM6_ESM.zip › InputFiles&OutputTrees/BayesCalibrated/Diversity(NoSA)/BayesCal_TK02_ln_p1_60G_DvNoSA_SFBD(s)2_2l/BayesCal_TK02_ln_p1_DvNoSA_SFBD2_AllCom.t.con.tre.pdf]

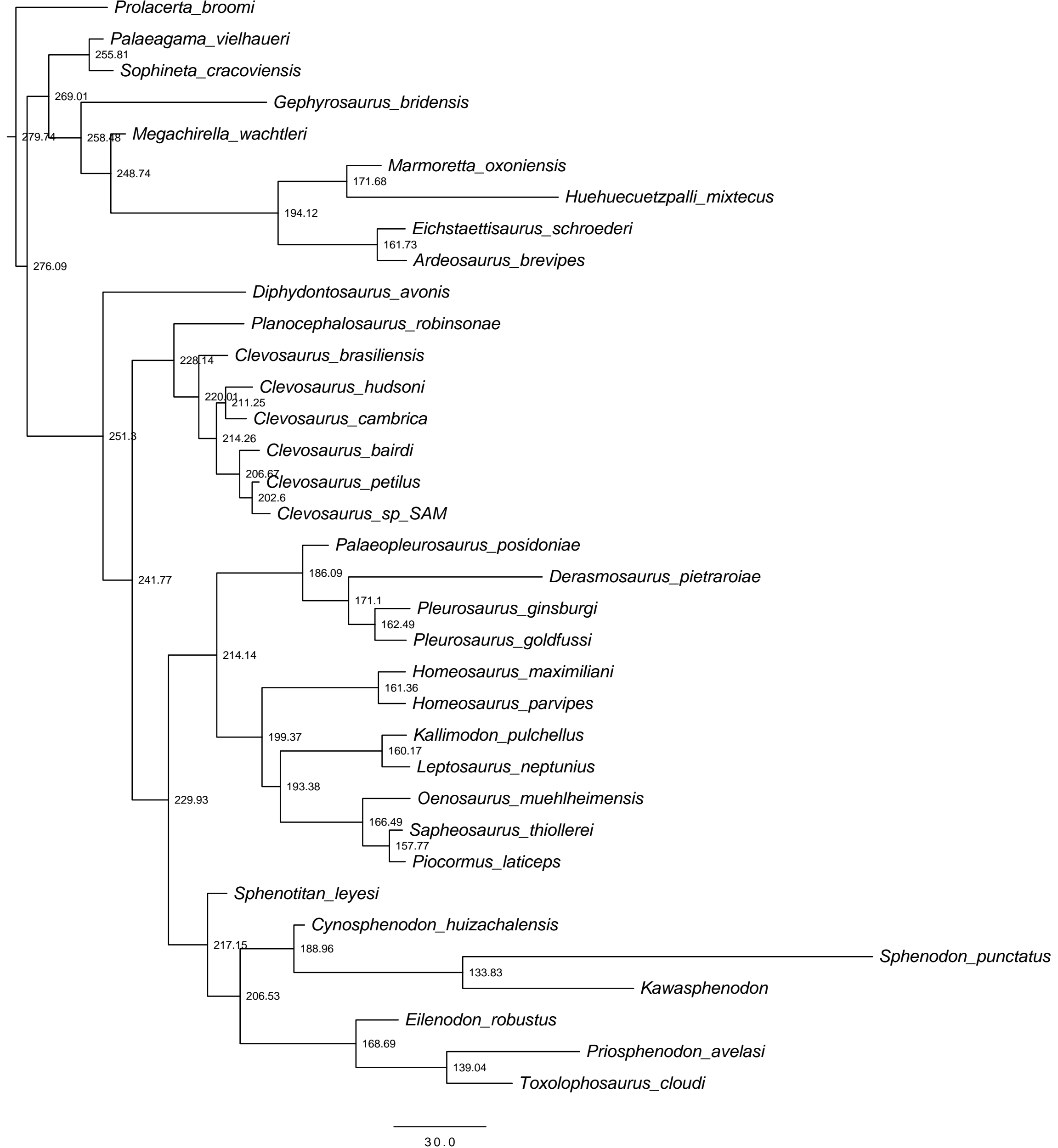

Supplement: Supplementary file 6 — Additional file 6. Input files including the dataset and all necessary coding (see Mr. Bayes blocks) to reproduce the analyses. [file 12915_2020_901_MOESM6_ESM.zip › InputFiles&OutputTrees/BayesCalibrated/Diversity(NoSA)/BayesCal_TK02_ln_p1_60G_DvNoSA_SFBD(s)2_2l/BayesCal_TK02_ln_p1_DvNoSA_SFBD2_AllCom.t.con.tre_Age.pdf]

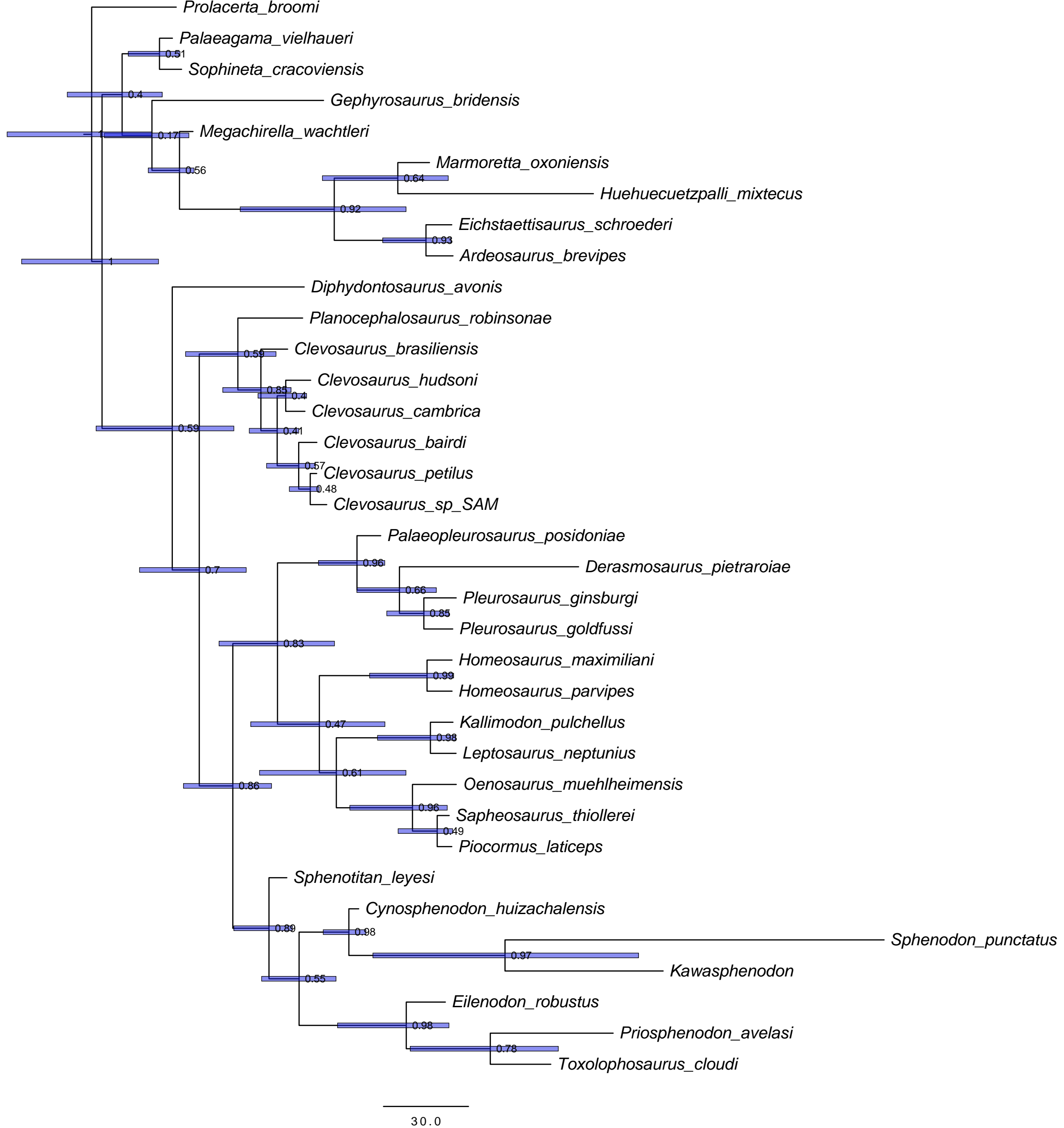

Supplement: Supplementary file 6 — Additional file 6. Input files including the dataset and all necessary coding (see Mr. Bayes blocks) to reproduce the analyses. [file 12915_2020_901_MOESM6_ESM.zip › InputFiles&OutputTrees/BayesCalibrated/Diversity(NoSA)/BayesCal_TK02_ln_p1_60G_DvNoSA_SFBD(s)2_2l/BayesCal_TK02_ln_p1_DvNoSA_SFBD2_AllCom.t.con.tre_AgeBars.pdf]

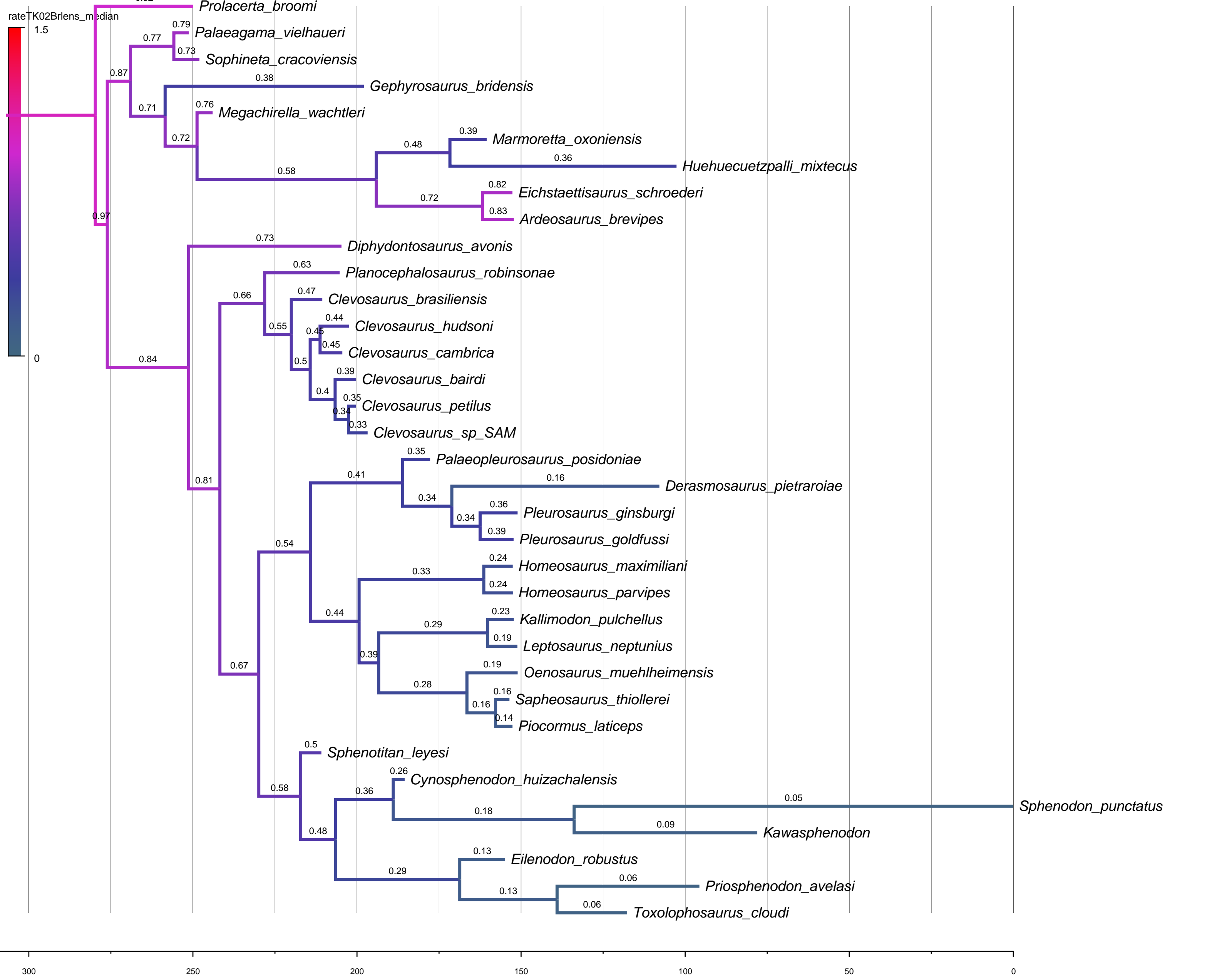

Supplement: Supplementary file 6 — Additional file 6. Input files including the dataset and all necessary coding (see Mr. Bayes blocks) to reproduce the analyses. [file 12915_2020_901_MOESM6_ESM.zip › InputFiles&OutputTrees/BayesCalibrated/Diversity(NoSA)/BayesCal_TK02_ln_p1_60G_DvNoSA_SFBD(s)2_2l/BayesCal_TK02_ln_p1_DvNoSA_SFBD2_AllCom.t.con.tre_Rate.pdf]

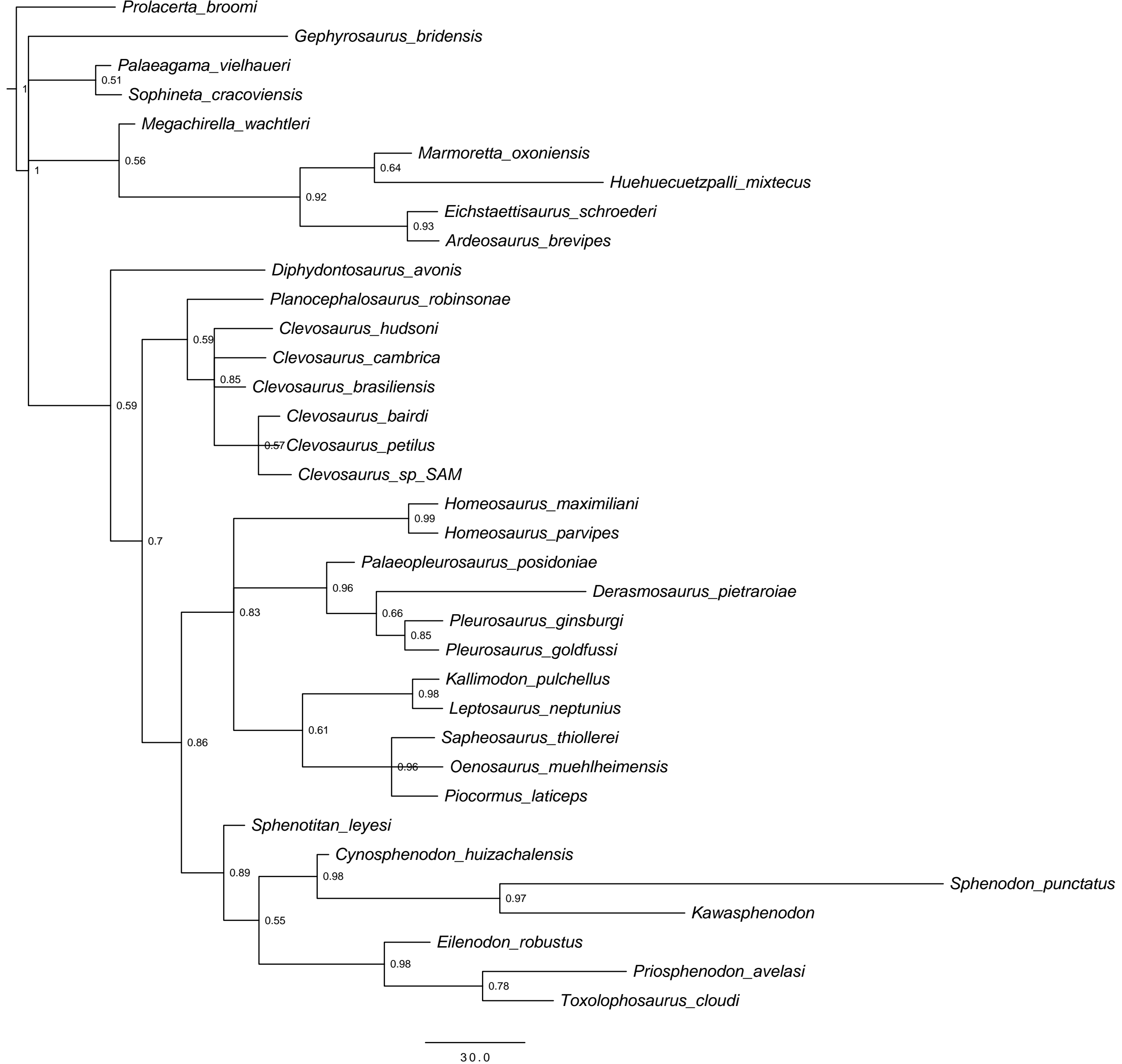

Supplement: Supplementary file 6 — Additional file 6. Input files including the dataset and all necessary coding (see Mr. Bayes blocks) to reproduce the analyses. [file 12915_2020_901_MOESM6_ESM.zip › InputFiles&OutputTrees/BayesCalibrated/Diversity(NoSA)/BayesCal_TK02_ln_p1_60G_DvNoSA_SFBD(s)2_2l/BayesCal_TK02_ln_p1_DvNoSA_SFBD2_MRC.t.con.tre.pdf]

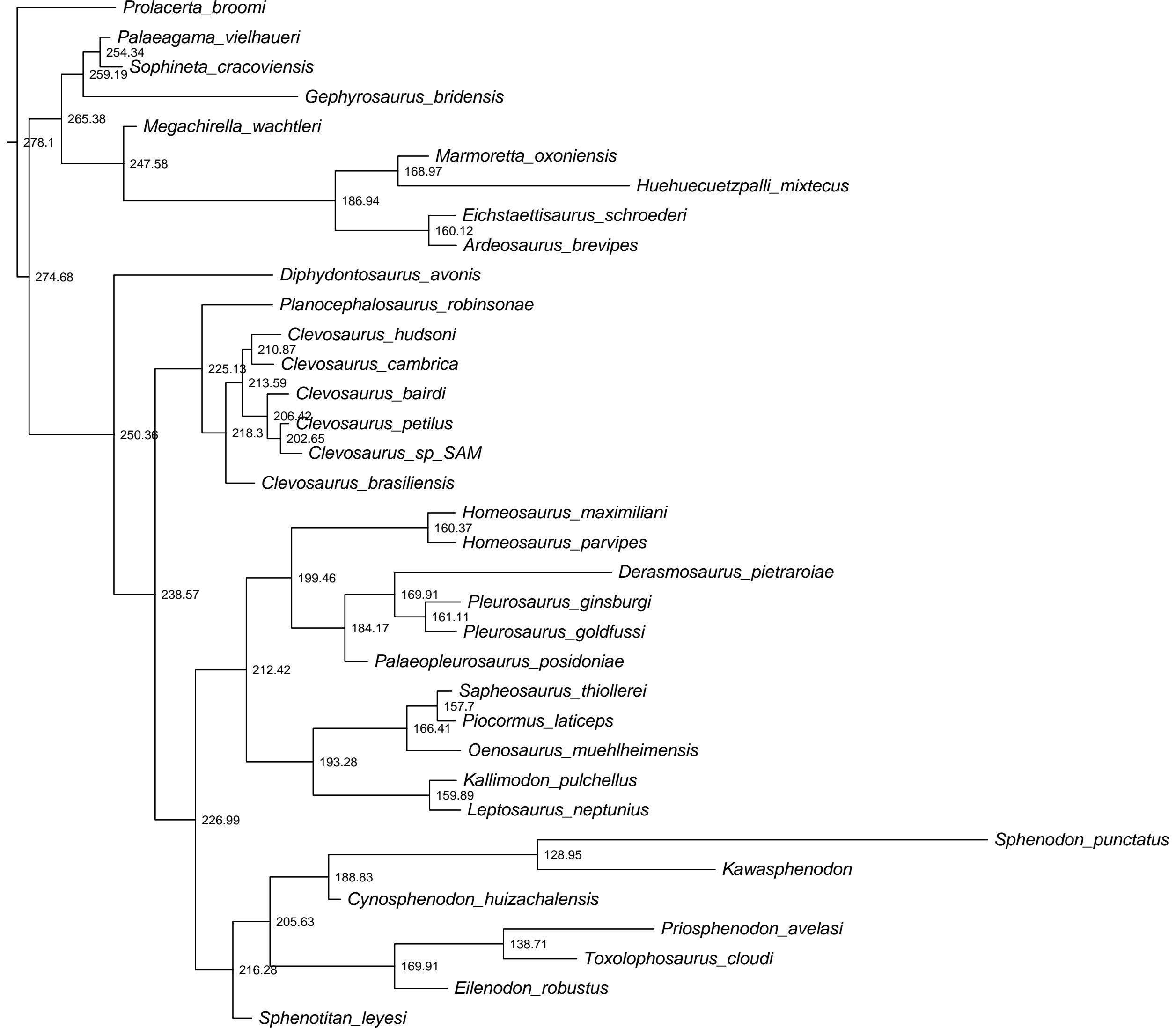

30.0

Supplement: Supplementary file 6 — Additional file 6. Input files including the dataset and all necessary coding (see Mr. Bayes blocks) to reproduce the analyses. [file 12915_2020_901_MOESM6_ESM.zip › InputFiles&OutputTrees/BayesCalibrated/Diversity(NoSA)/BayesCal_TK02_ln_p1_60G_DvNoSA_SFBD(s)2_2l_Asym/BayesCal_TK02_ln_p1_DvNoSA_SFBD2_Asym_AllCom.t.con.tre_Ages.pdf]

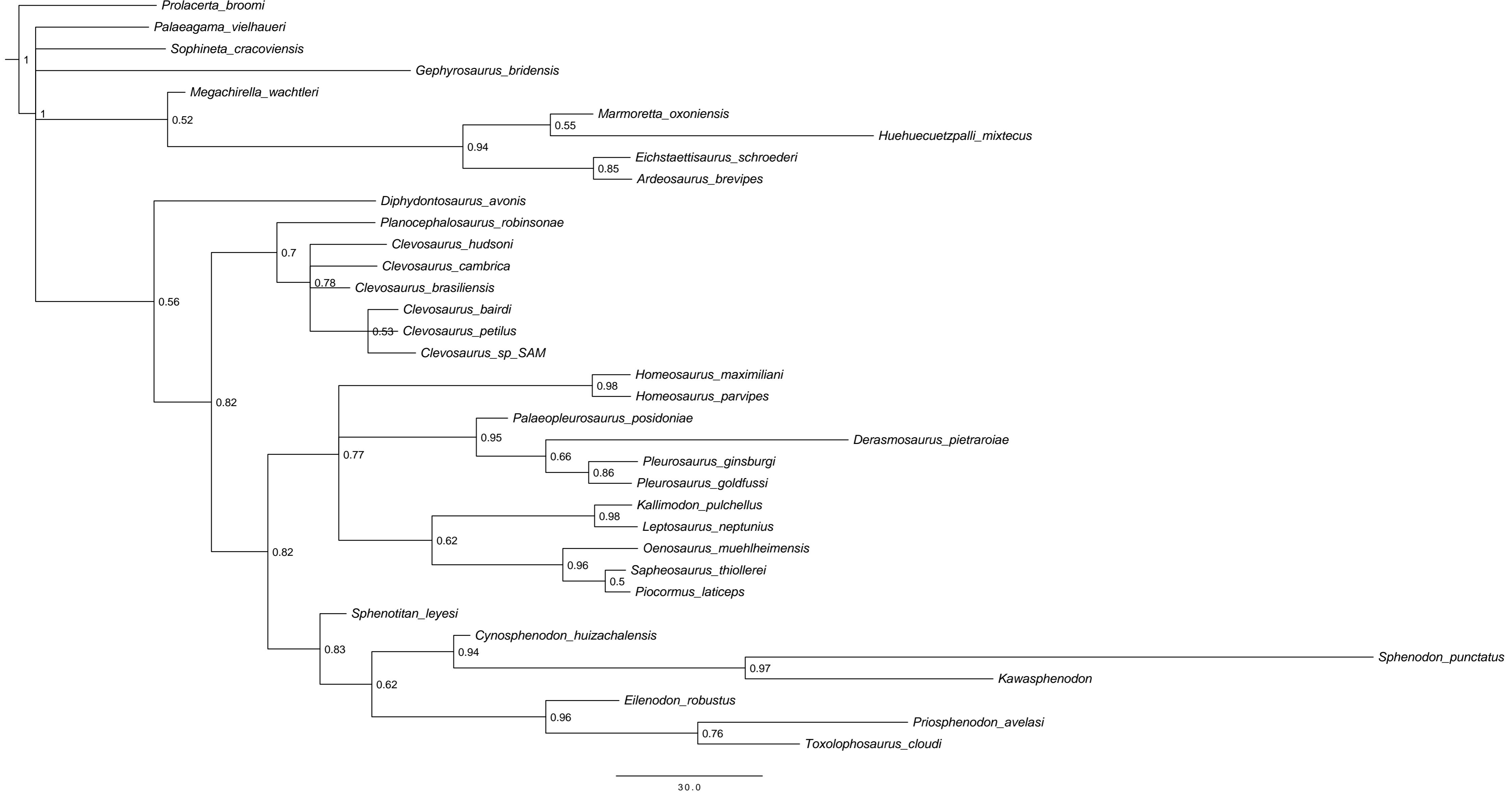

Supplement: Supplementary file 6 — Additional file 6. Input files including the dataset and all necessary coding (see Mr. Bayes blocks) to reproduce the analyses. [file 12915_2020_901_MOESM6_ESM.zip › InputFiles&OutputTrees/BayesCalibrated/Diversity(NoSA)/BayesCal_TK02_ln_p1_60G_DvNoSA_SFBD(s)2_2l_Asym/BayesCal_TK02_ln_p1_DvNoSA_SFBD2_Asym_MRC.t.con.tre.pdf]

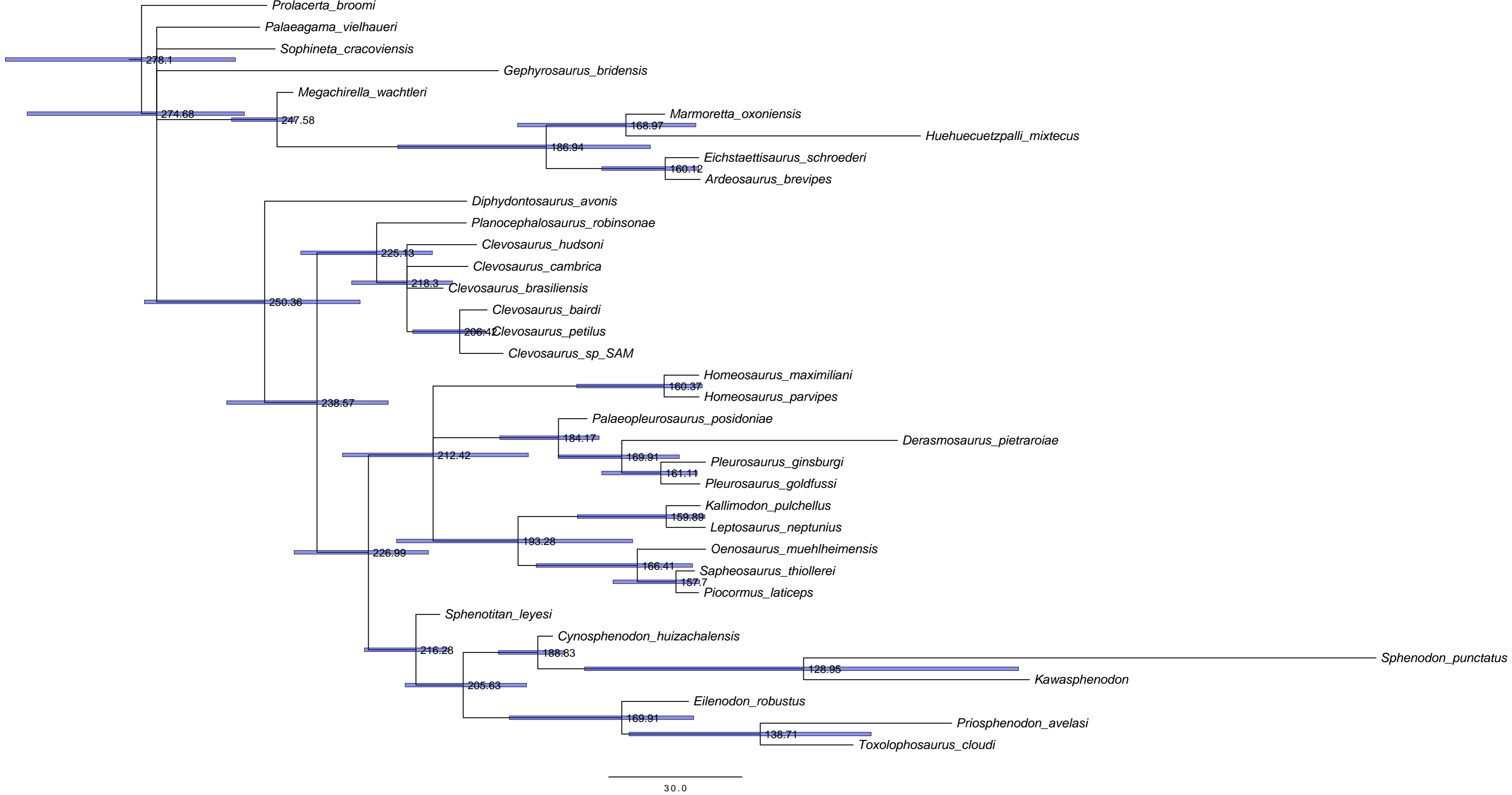

Supplement: Supplementary file 6 — Additional file 6. Input files including the dataset and all necessary coding (see Mr. Bayes blocks) to reproduce the analyses. [file 12915_2020_901_MOESM6_ESM.zip › InputFiles&OutputTrees/BayesCalibrated/Diversity(NoSA)/BayesCal_TK02_ln_p1_60G_DvNoSA_SFBD(s)2_2l_Asym/BayesCal_TK02_ln_p1_DvNoSA_SFBD2_Asym_MRC.t.con.tre_AgeBars.pdf]

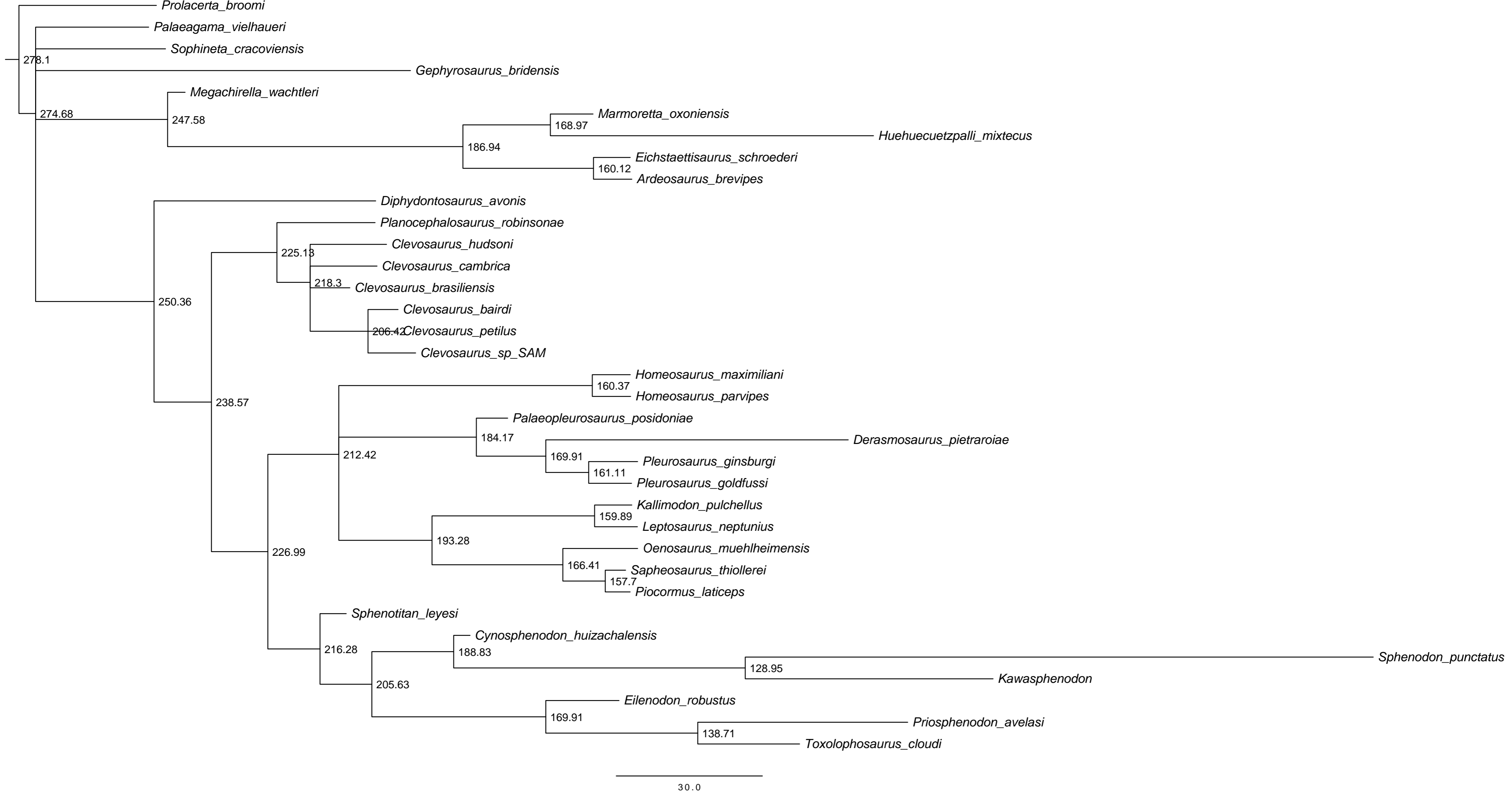

Supplement: Supplementary file 6 — Additional file 6. Input files including the dataset and all necessary coding (see Mr. Bayes blocks) to reproduce the analyses. [file 12915_2020_901_MOESM6_ESM.zip › InputFiles&OutputTrees/BayesCalibrated/Diversity(NoSA)/BayesCal_TK02_ln_p1_60G_DvNoSA_SFBD(s)2_2l_Asym/BayesCal_TK02_ln_p1_DvNoSA_SFBD2_Asym_MRC.t.con.tre_Ages.pdf]

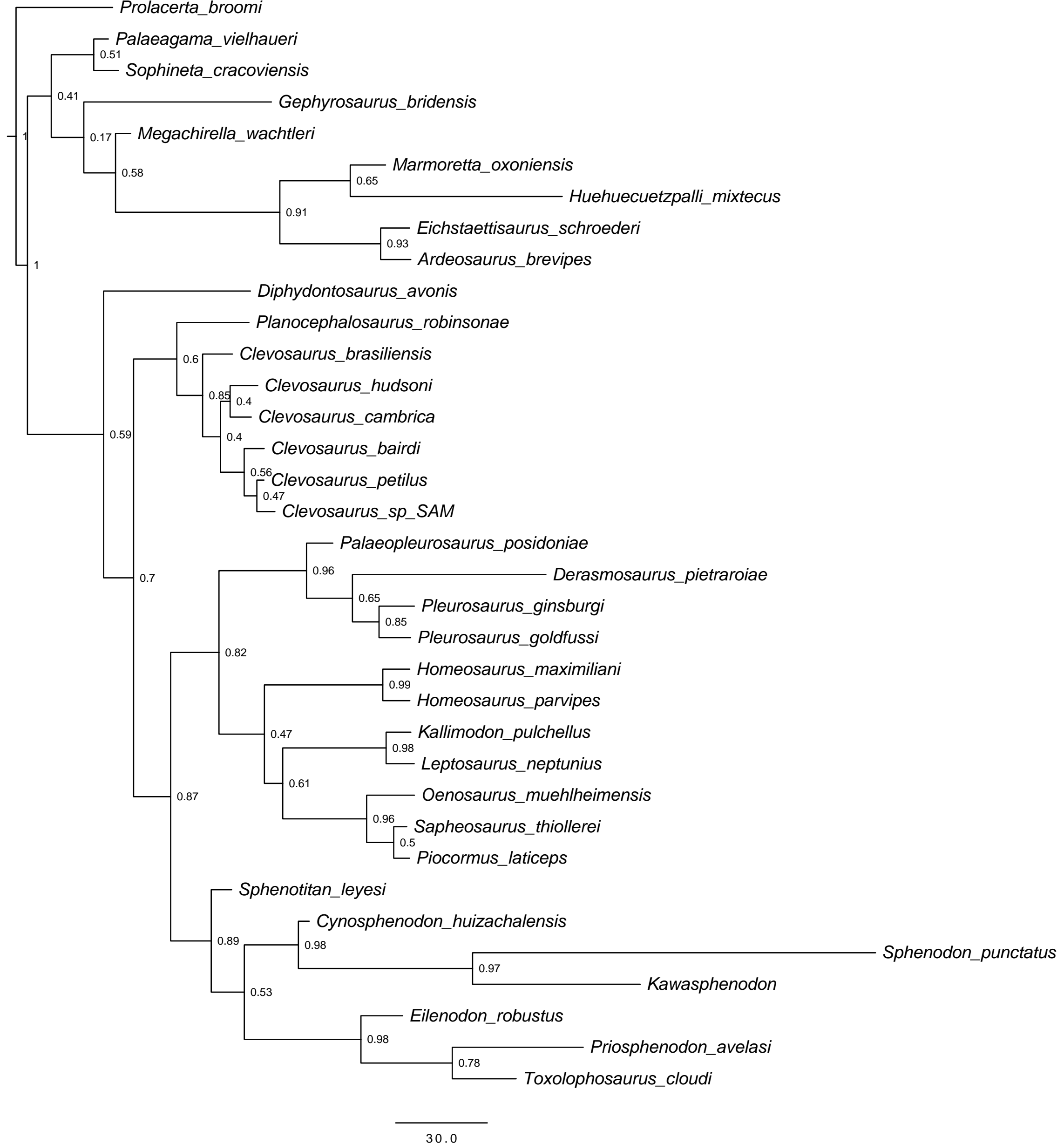

Supplement: Supplementary file 6 — Additional file 6. Input files including the dataset and all necessary coding (see Mr. Bayes blocks) to reproduce the analyses. [file 12915_2020_901_MOESM6_ESM.zip › InputFiles&OutputTrees/BayesCalibrated/Diversity(NoSA)/BayesCal_TK02_ln_p1_60G_DvNoSA_SFBD(s)2_3l/BayesCal_TK02_ln_p1_DvNoSA_SFBD3_AllCom.t.con.tre.pdf]

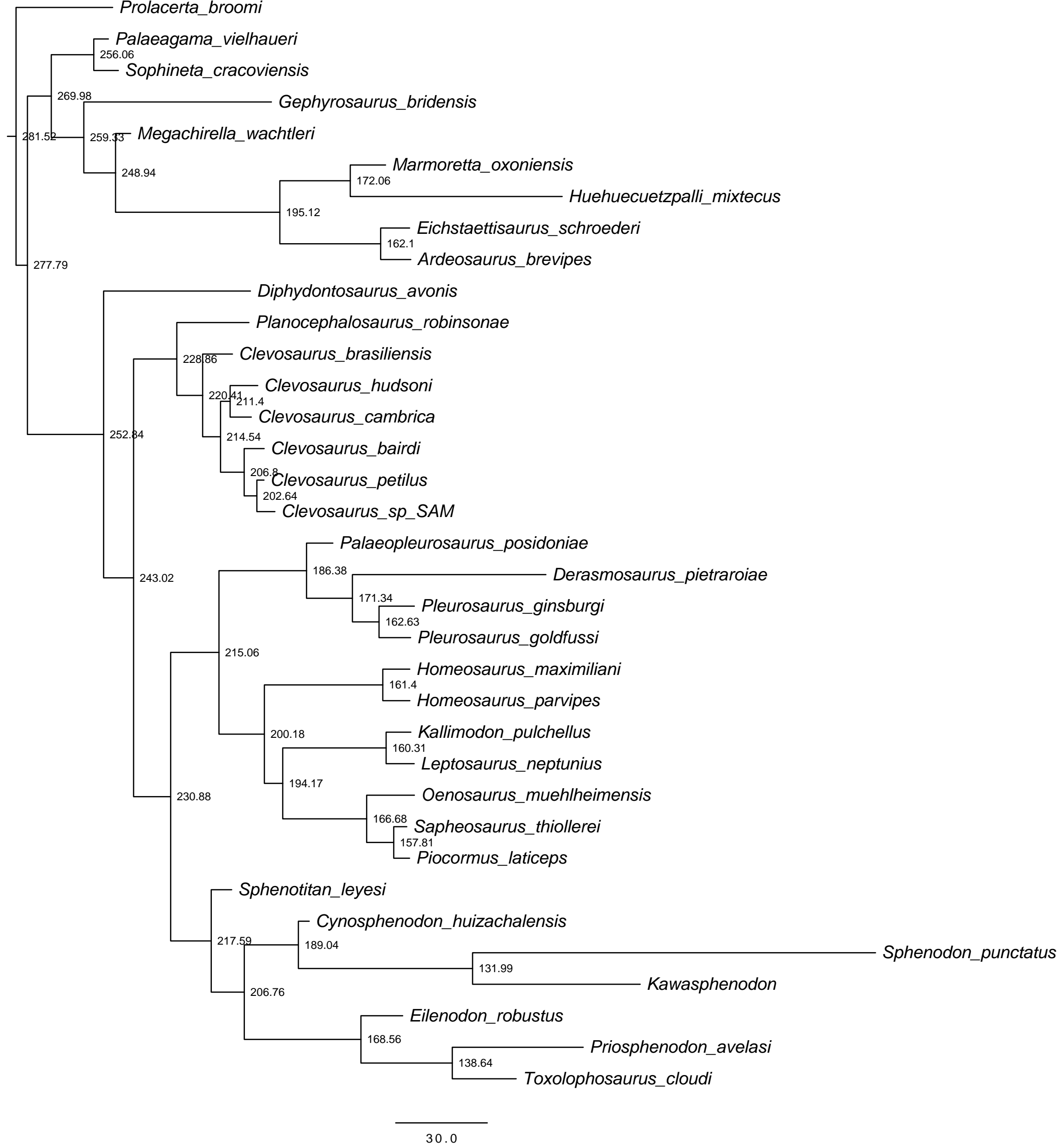

Supplement: Supplementary file 6 — Additional file 6. Input files including the dataset and all necessary coding (see Mr. Bayes blocks) to reproduce the analyses. [file 12915_2020_901_MOESM6_ESM.zip › InputFiles&OutputTrees/BayesCalibrated/Diversity(NoSA)/BayesCal_TK02_ln_p1_60G_DvNoSA_SFBD(s)2_3l/BayesCal_TK02_ln_p1_DvNoSA_SFBD3_AllCom.t.con.tre_Age.pdf]

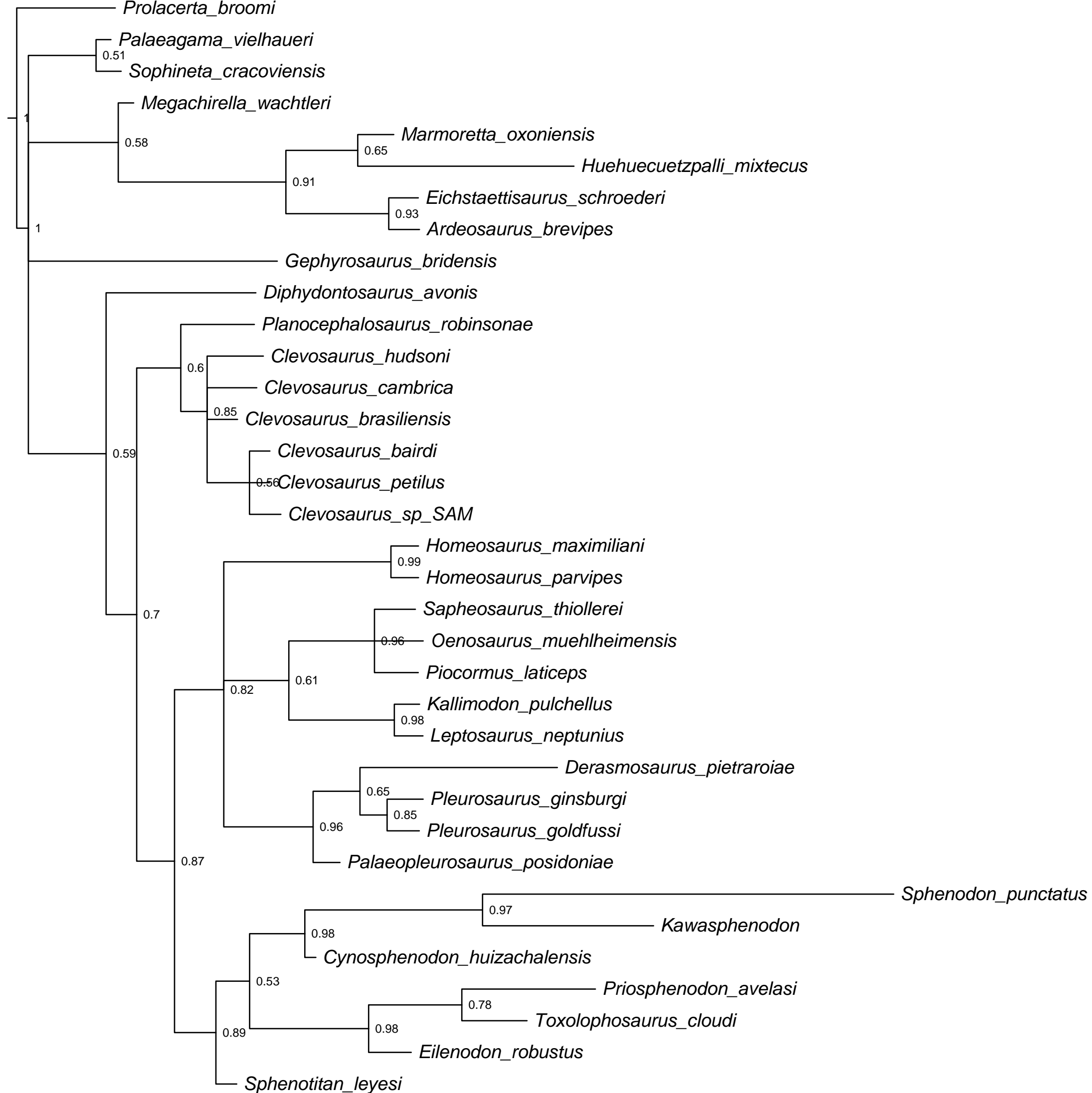

Supplement: Supplementary file 6 — Additional file 6. Input files including the dataset and all necessary coding (see Mr. Bayes blocks) to reproduce the analyses. [file 12915_2020_901_MOESM6_ESM.zip › InputFiles&OutputTrees/BayesCalibrated/Diversity(NoSA)/BayesCal_TK02_ln_p1_60G_DvNoSA_SFBD(s)2_3l/BayesCal_TK02_ln_p1_DvNoSA_SFBD3_MRC.t.con.tre.pdf]

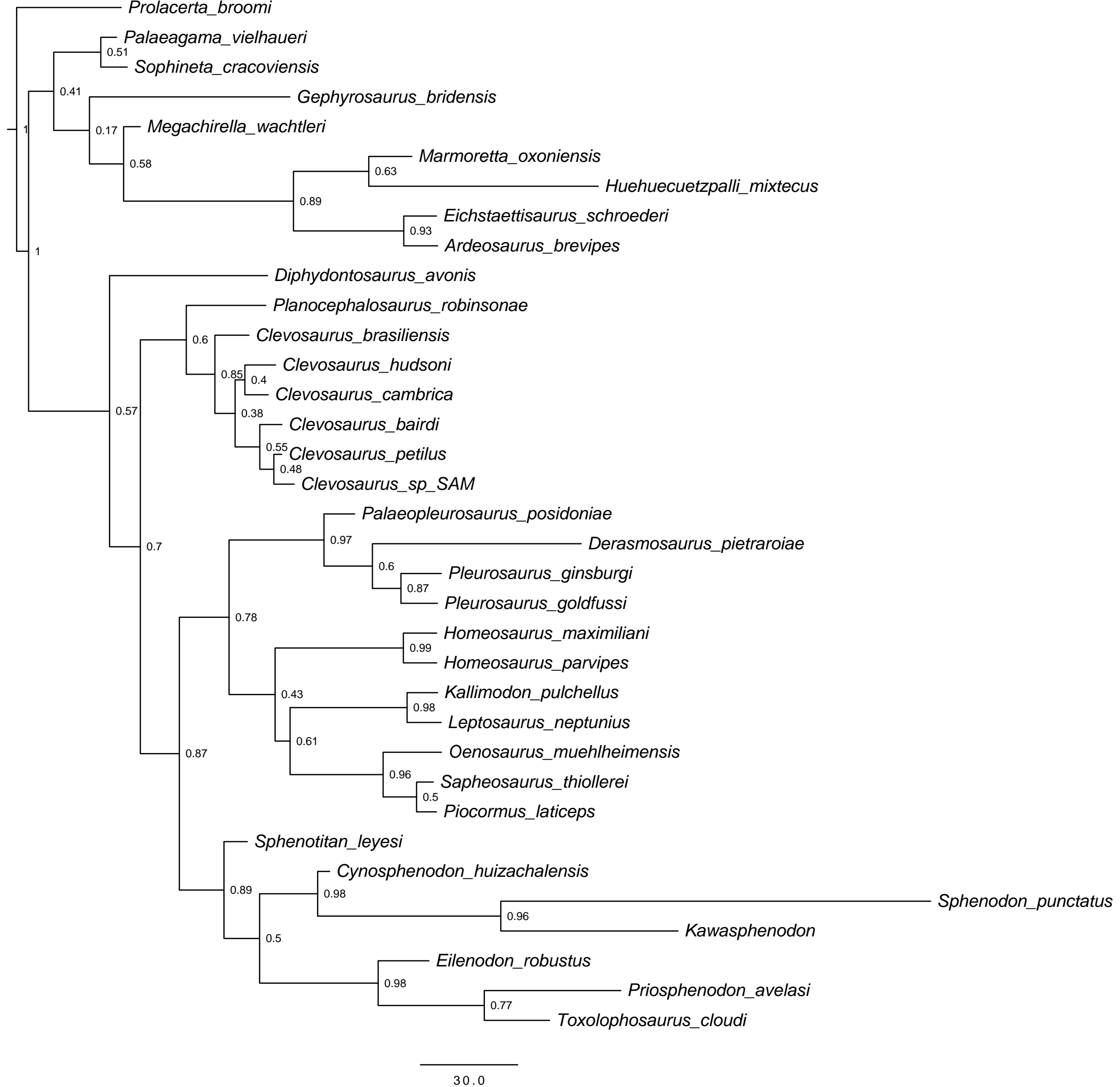

Supplement: Supplementary file 6 — Additional file 6. Input files including the dataset and all necessary coding (see Mr. Bayes blocks) to reproduce the analyses. [file 12915_2020_901_MOESM6_ESM.zip › InputFiles&OutputTrees/BayesCalibrated/Diversity(NoSA)/BayesCal_TK02_ln_p1_60G_DvNoSA_SFBD(sdr)2_2l/BayesCal_TK02_ln_p1_DvNoSA_SFBD2%28sdr%292l_AllCom.t.con.tre.pdf]

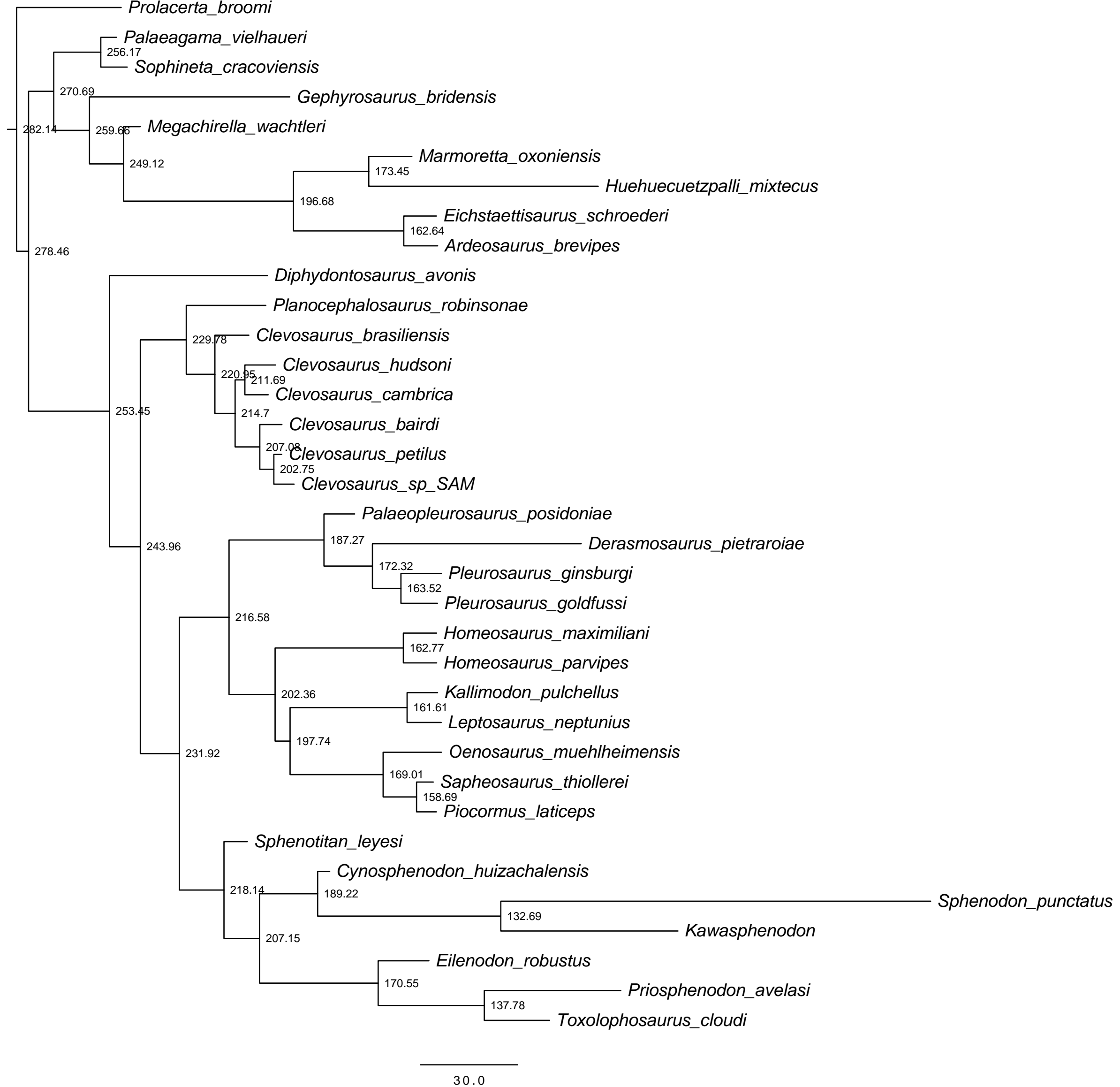

Supplement: Supplementary file 6 — Additional file 6. Input files including the dataset and all necessary coding (see Mr. Bayes blocks) to reproduce the analyses. [file 12915_2020_901_MOESM6_ESM.zip › InputFiles&OutputTrees/BayesCalibrated/Diversity(NoSA)/BayesCal_TK02_ln_p1_60G_DvNoSA_SFBD(sdr)2_2l/BayesCal_TK02_ln_p1_DvNoSA_SFBD2%28sdr%292l_AllCom.t.con.tre_Age.pdf]

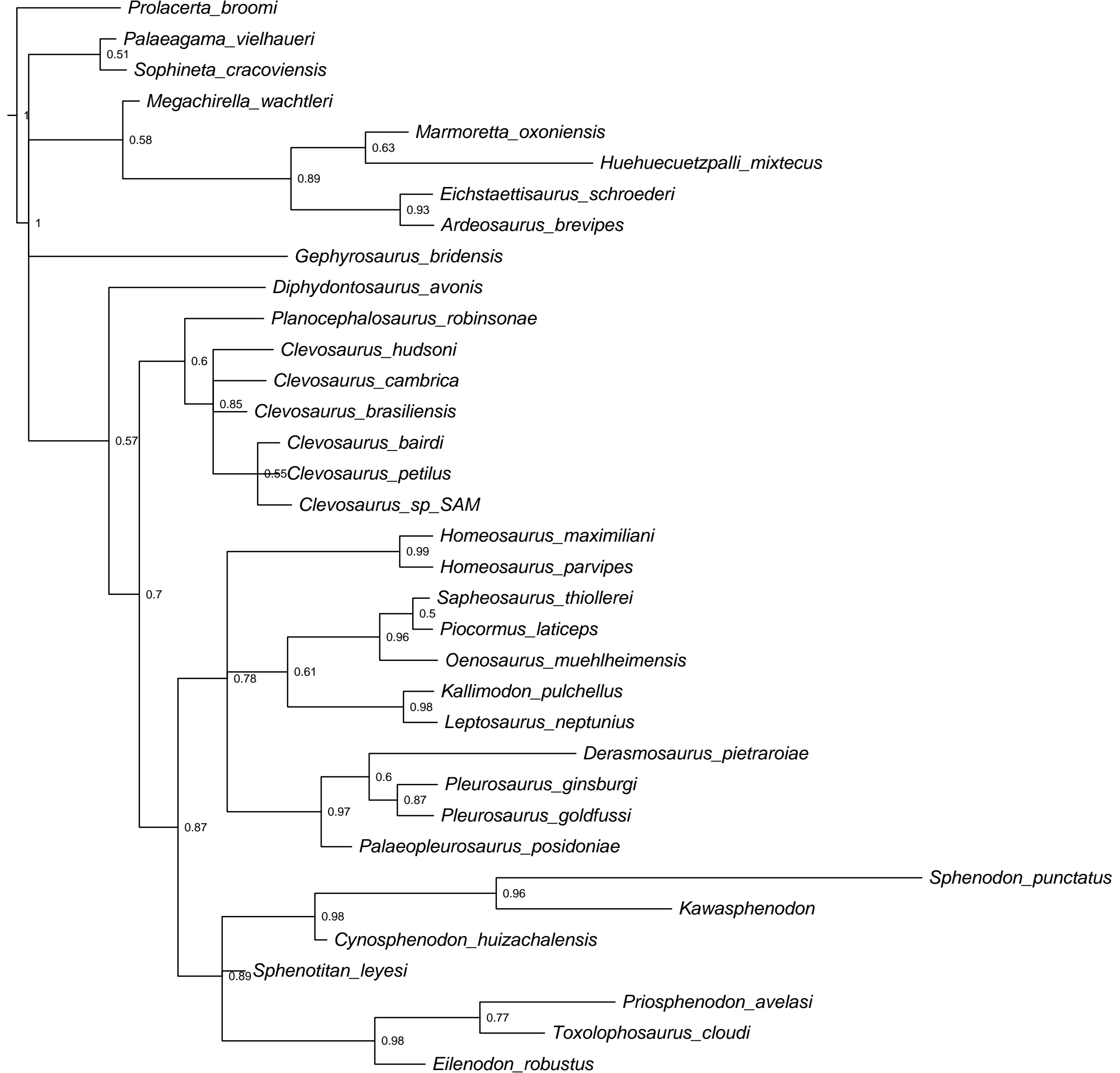

30.0

Supplement: Supplementary file 6 — Additional file 6. Input files including the dataset and all necessary coding (see Mr. Bayes blocks) to reproduce the analyses. [file 12915_2020_901_MOESM6_ESM.zip › InputFiles&OutputTrees/BayesCalibrated/Diversity(NoSA)/BayesCal_TK02_ln_p1_60G_DvNoSA_SFBD(sdr)2_2l/BayesCal_TK02_ln_p1_DvNoSA_SFBD2%28sdr%292l_MRC.t.con.tre.pdf]

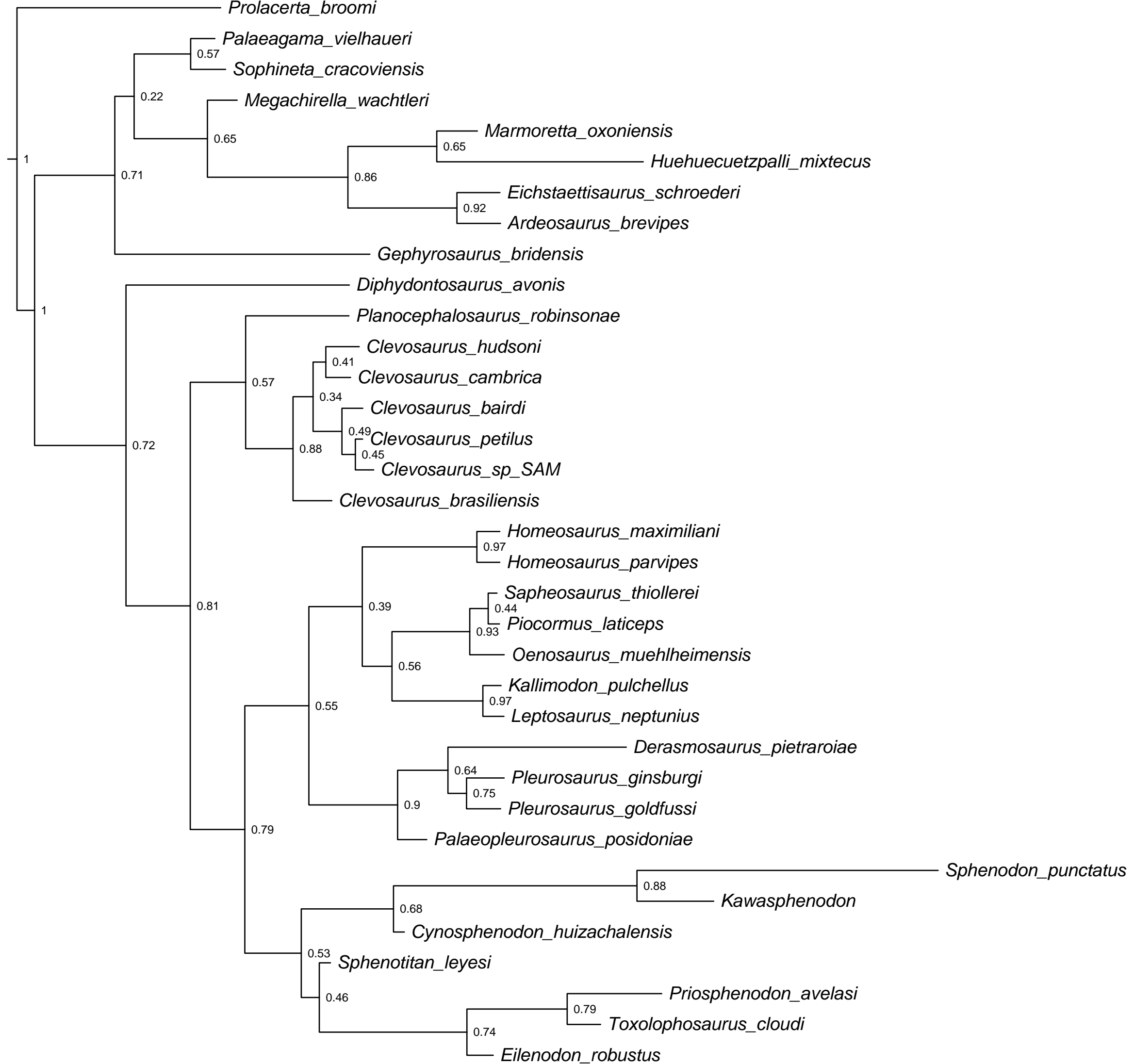

40.0

Supplement: Supplementary file 6 — Additional file 6. Input files including the dataset and all necessary coding (see Mr. Bayes blocks) to reproduce the analyses. [file 12915_2020_901_MOESM6_ESM.zip › InputFiles&OutputTrees/BayesCalibrated/Diversity(NoSA)/BayesCal_TK02_ln_p3_60G_DvNoSA/BayesCal_TK02_ln_p3_DvNoSA_AllCom.t.con.tre.pdf]

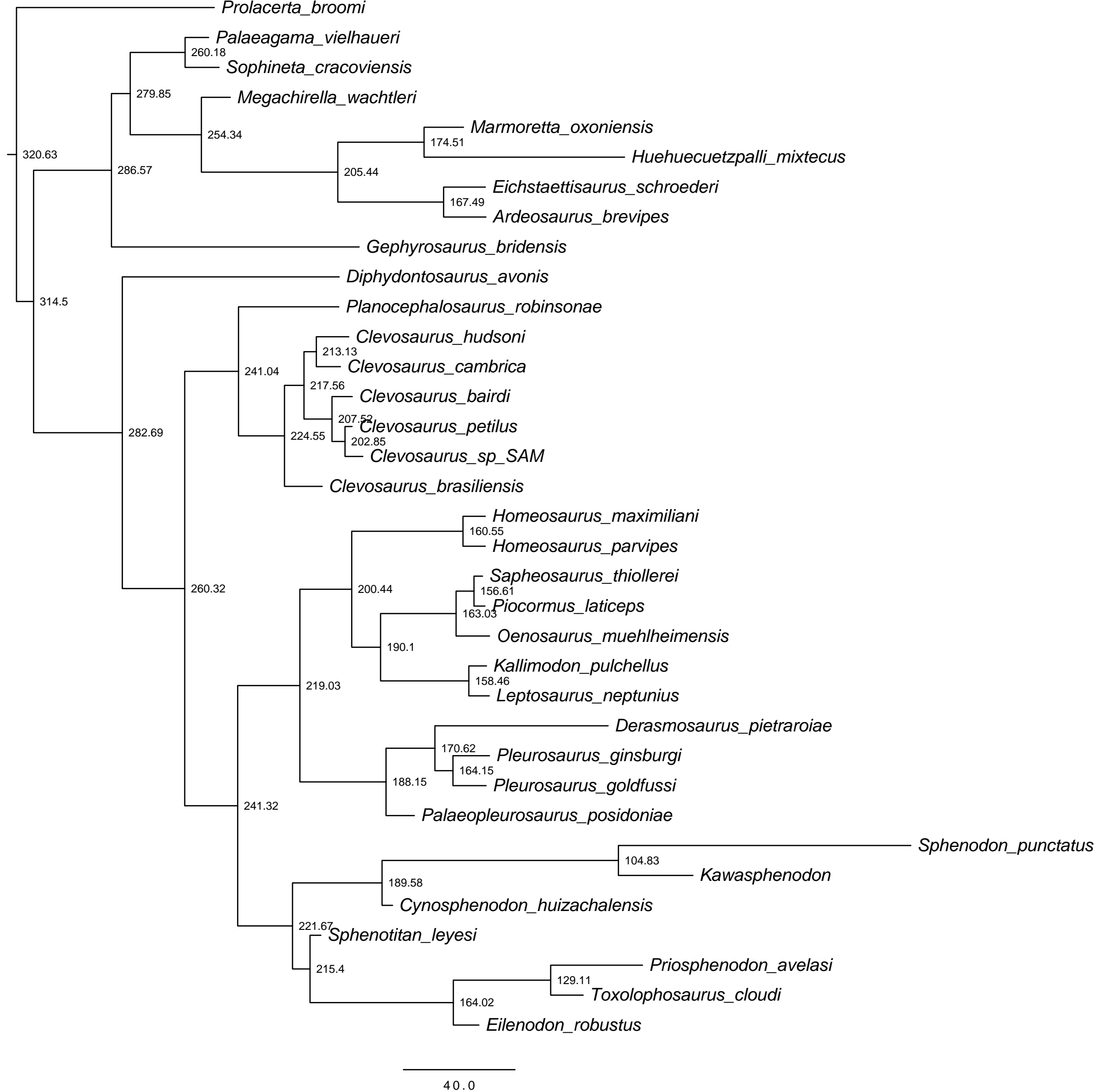

Supplement: Supplementary file 6 — Additional file 6. Input files including the dataset and all necessary coding (see Mr. Bayes blocks) to reproduce the analyses. [file 12915_2020_901_MOESM6_ESM.zip › InputFiles&OutputTrees/BayesCalibrated/Diversity(NoSA)/BayesCal_TK02_ln_p3_60G_DvNoSA/BayesCal_TK02_ln_p3_DvNoSA_AllCom.t.con.tre_Ages.pdf]

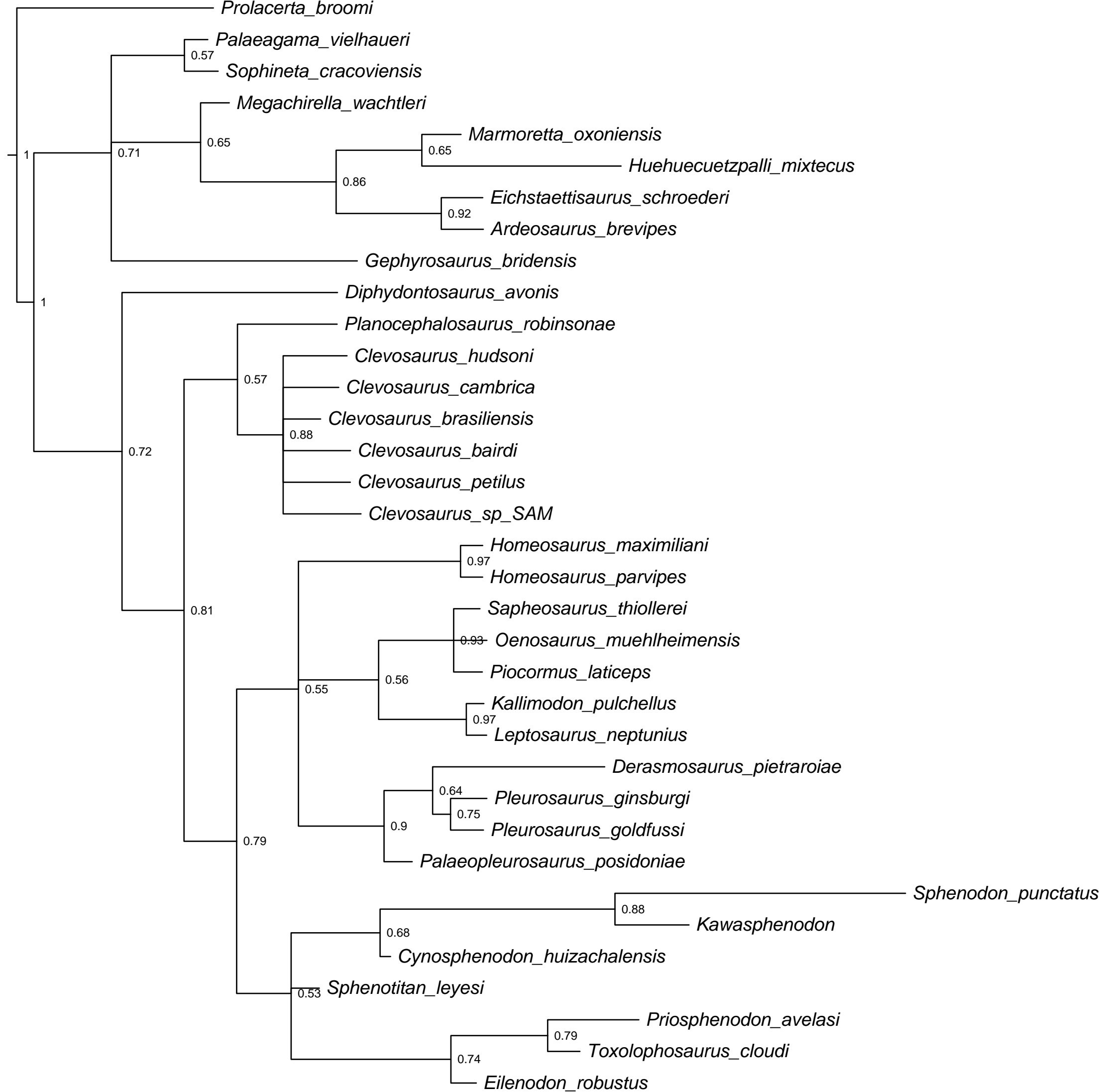

Supplement: Supplementary file 6 — Additional file 6. Input files including the dataset and all necessary coding (see Mr. Bayes blocks) to reproduce the analyses. [file 12915_2020_901_MOESM6_ESM.zip › InputFiles&OutputTrees/BayesCalibrated/Diversity(NoSA)/BayesCal_TK02_ln_p3_60G_DvNoSA/BayesCal_TK02_ln_p3_DvNoSA_MRC.t.con.tre.pdf]

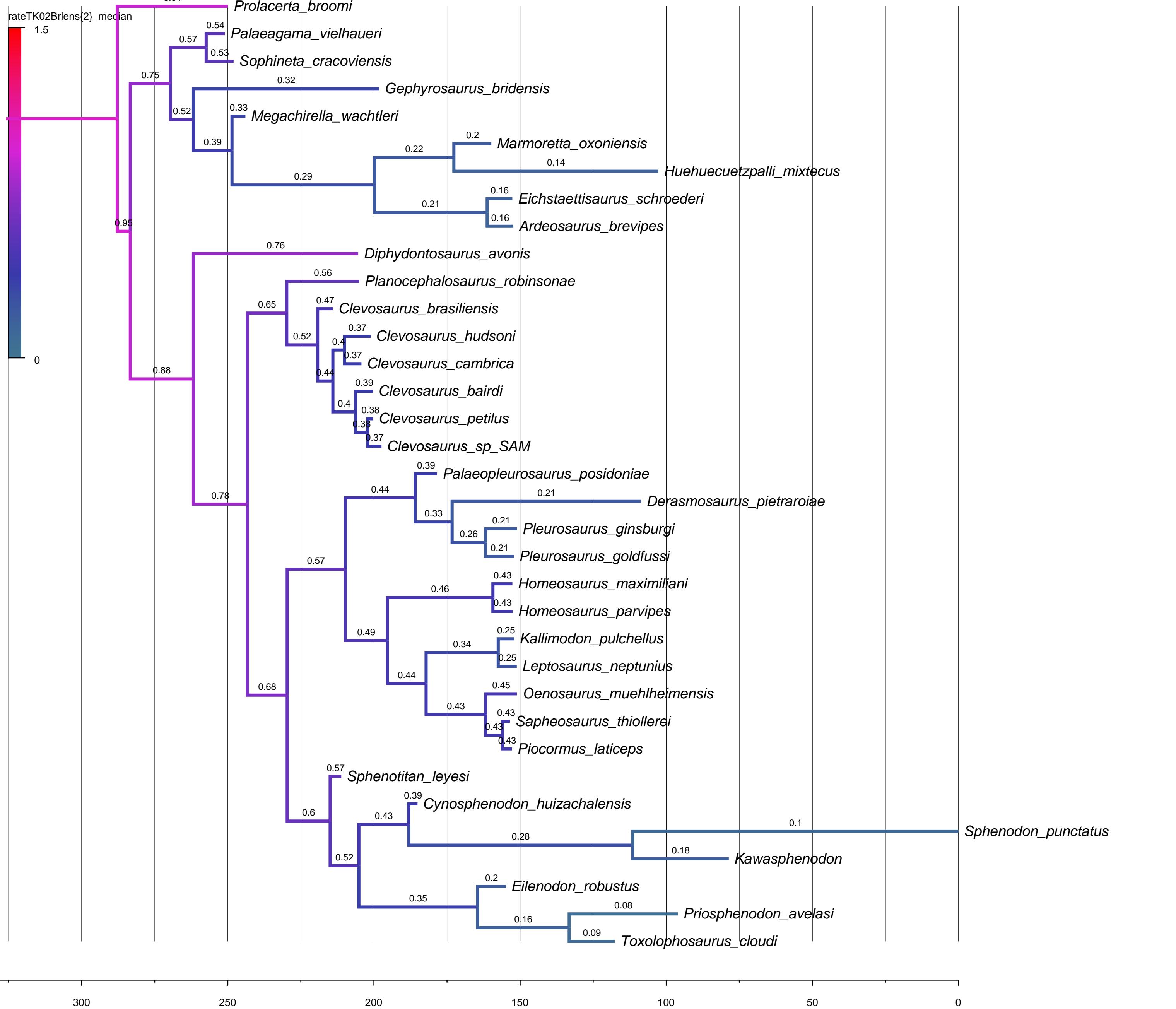

Supplement: Supplementary file 6 — Additional file 6. Input files including the dataset and all necessary coding (see Mr. Bayes blocks) to reproduce the analyses. [file 12915_2020_901_MOESM6_ESM.zip › InputFiles&OutputTrees/BayesCalibrated/Diversity(NoSA)/BayesCal_TK02_ln_p3_60G_DvNoSA_NoR_Rate+TopCons/BayesCal_TK02_ln_p3_DvNoSA_NoR_Rate%2BTopCons_AllCom.t.con.tre_RateMandTeeth.pdf]

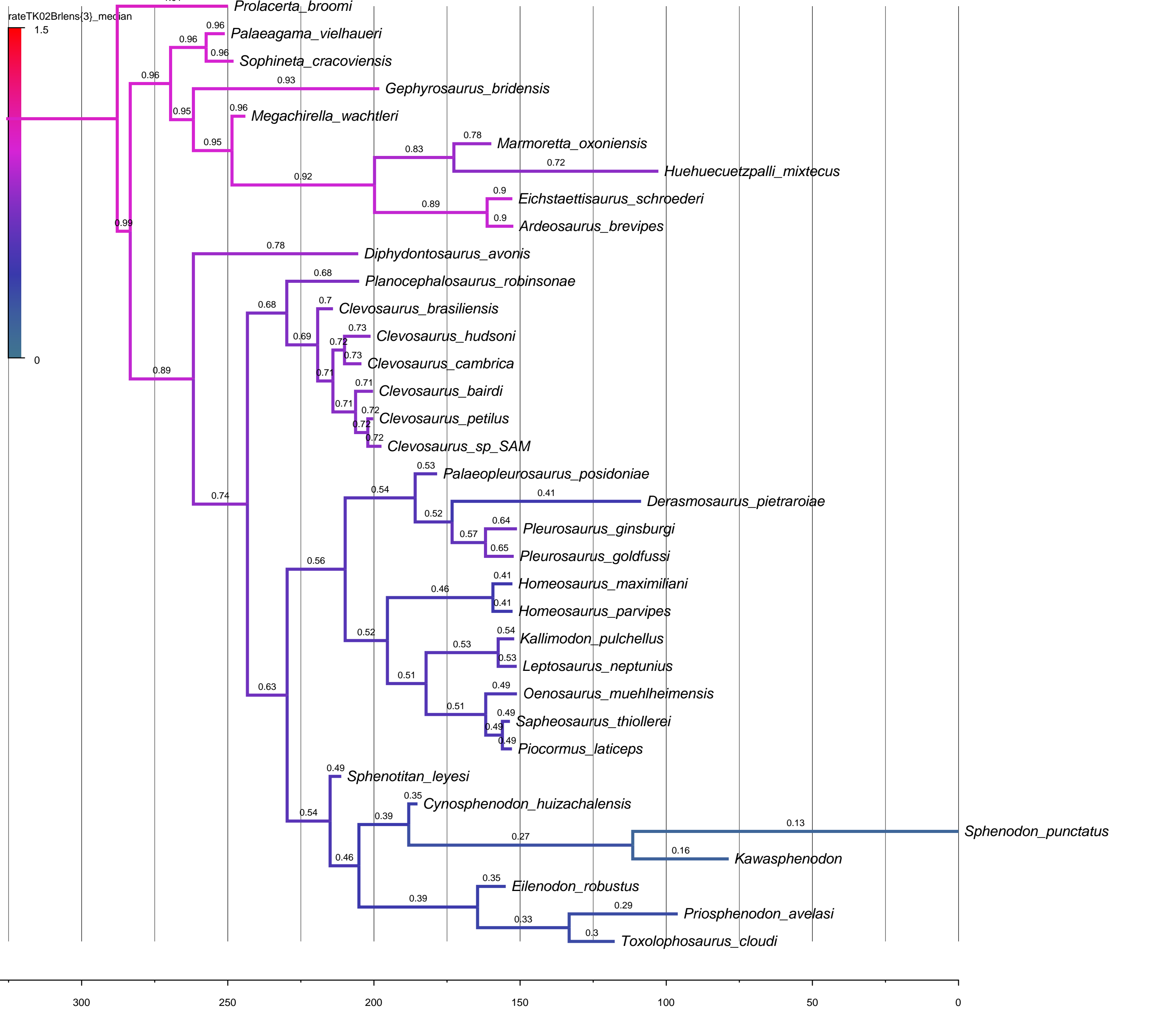

Supplement: Supplementary file 6 — Additional file 6. Input files including the dataset and all necessary coding (see Mr. Bayes blocks) to reproduce the analyses. [file 12915_2020_901_MOESM6_ESM.zip › InputFiles&OutputTrees/BayesCalibrated/Diversity(NoSA)/BayesCal_TK02_ln_p3_60G_DvNoSA_NoR_Rate+TopCons/BayesCal_TK02_ln_p3_DvNoSA_NoR_Rate%2BTopCons_AllCom.t.con.tre_RatePostCran.pdf]

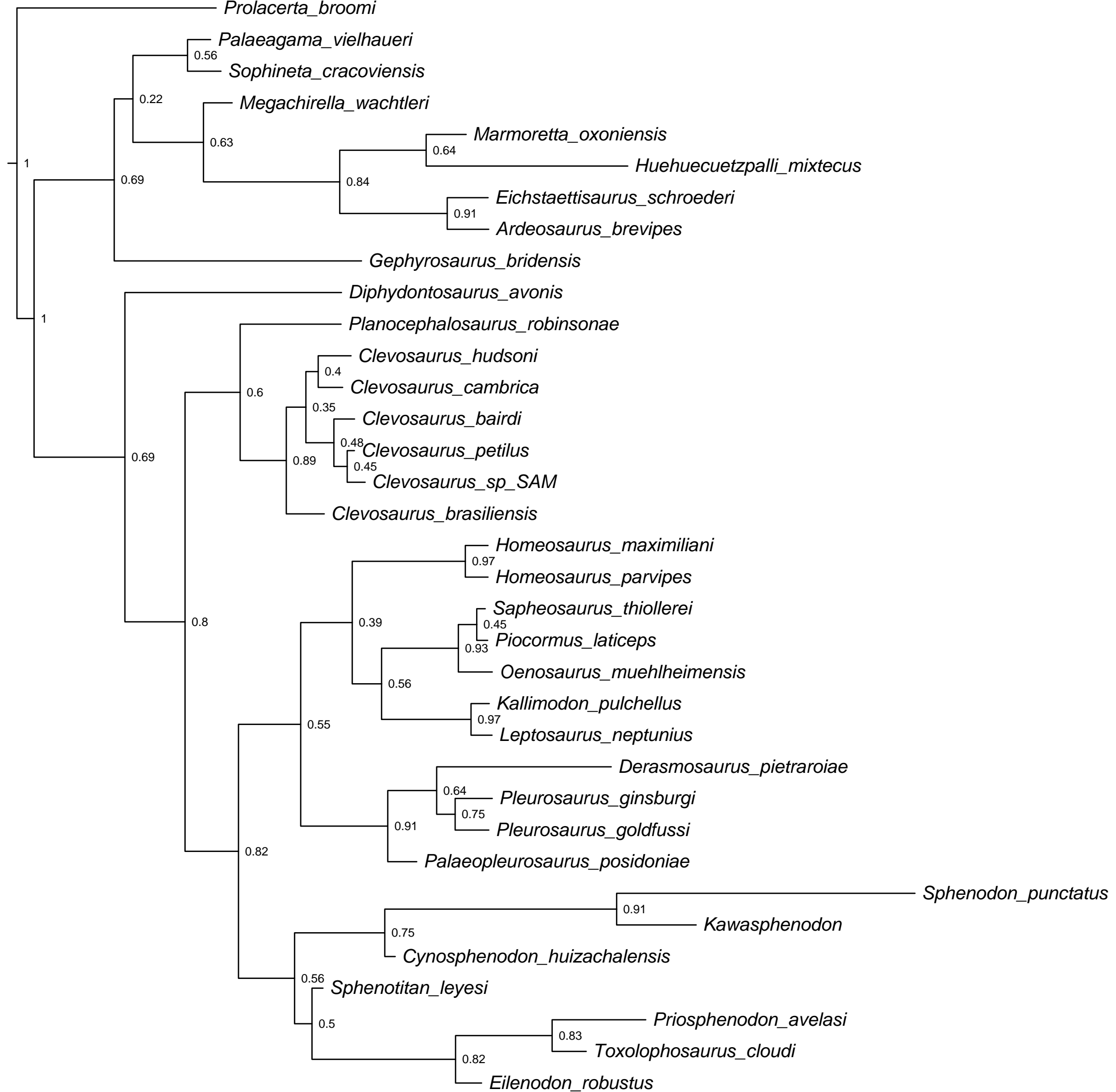

40.0

Supplement: Supplementary file 6 — Additional file 6. Input files including the dataset and all necessary coding (see Mr. Bayes blocks) to reproduce the analyses. [file 12915_2020_901_MOESM6_ESM.zip › InputFiles&OutputTrees/BayesCalibrated/Diversity(NoSA)/BayesCal_TK02_ln_p3_StartTr_3per_60G_DvNoSA/BayesCal_TK02_ln_p3_StartTr_3per_DvNoSA_AllCom.t.con.tre.pdf]

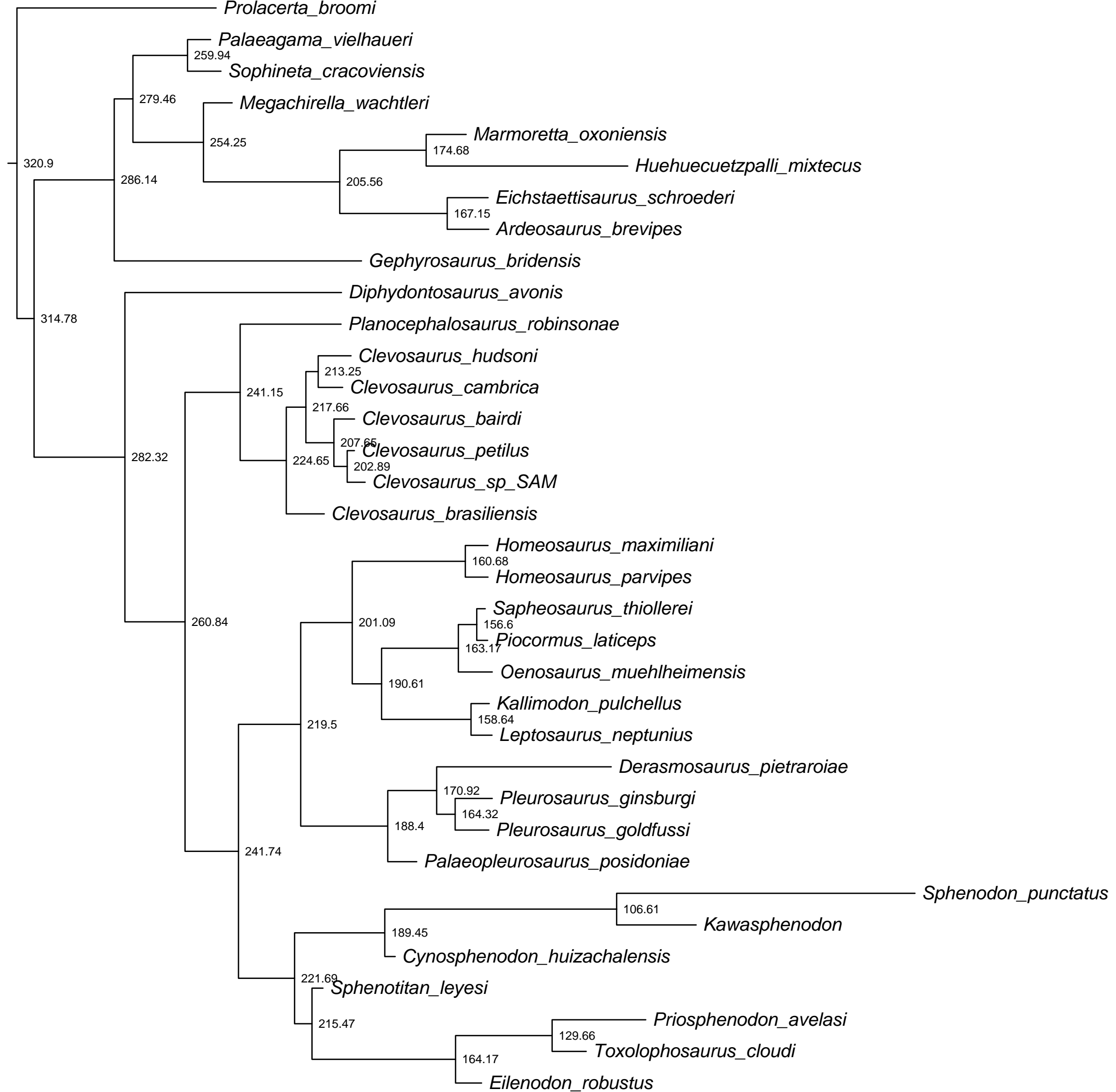

40.0

Supplement: Supplementary file 6 — Additional file 6. Input files including the dataset and all necessary coding (see Mr. Bayes blocks) to reproduce the analyses. [file 12915_2020_901_MOESM6_ESM.zip › InputFiles&OutputTrees/BayesCalibrated/Diversity(NoSA)/BayesCal_TK02_ln_p3_StartTr_3per_60G_DvNoSA/BayesCal_TK02_ln_p3_StartTr_3per_DvNoSA_AllCom.t.con.tre_Age.pdf]

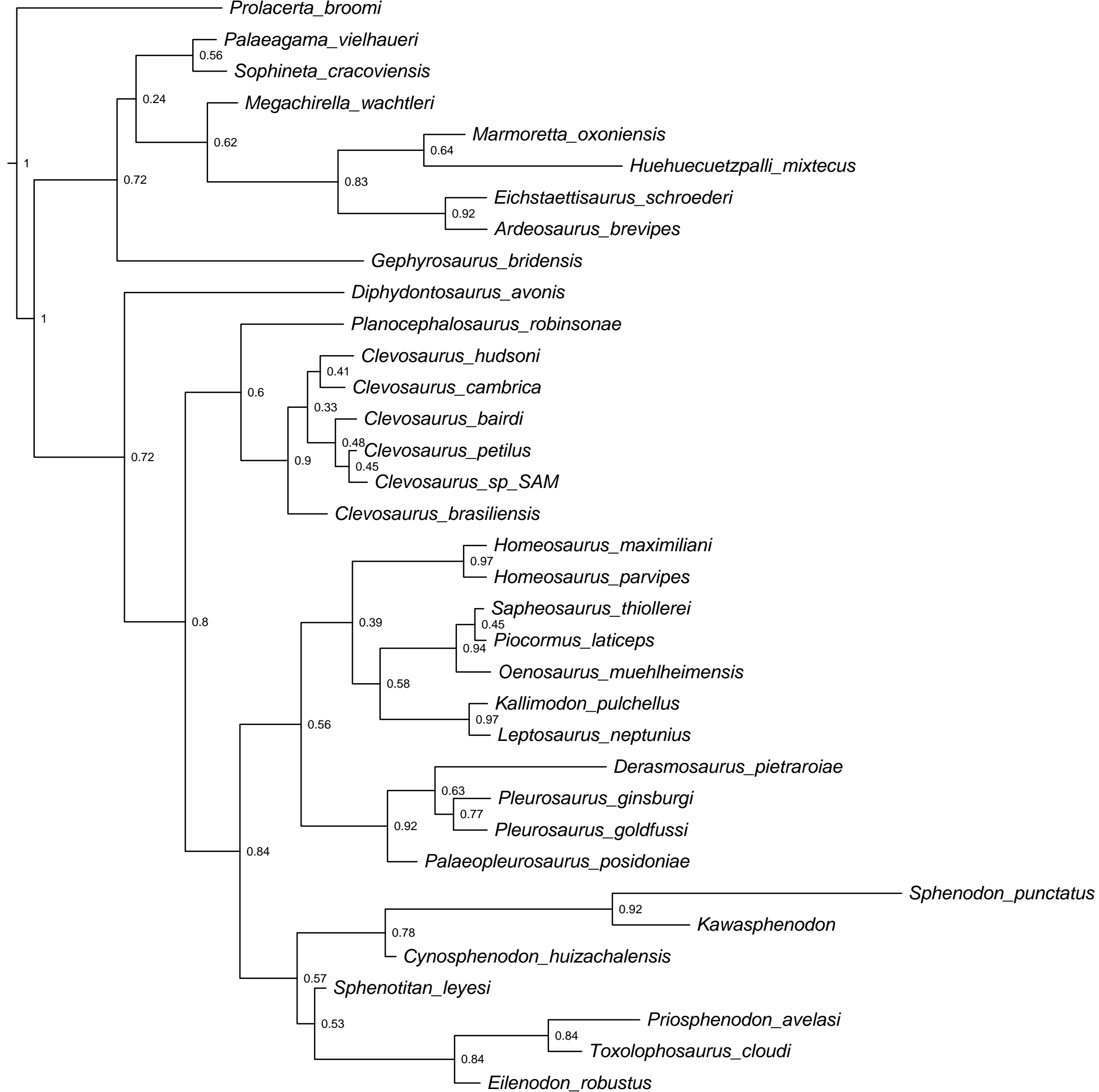

40.0

Supplement: Supplementary file 6 — Additional file 6. Input files including the dataset and all necessary coding (see Mr. Bayes blocks) to reproduce the analyses. [file 12915_2020_901_MOESM6_ESM.zip › InputFiles&OutputTrees/BayesCalibrated/Diversity(NoSA)/BayesCal_TK02_ln_p3_StartTr_3per_60G_DvNoSA_LExct/BayesCal_TK02_ln_p3_StartTr_3per_DvNoSA_AllCom.t.con.tre.pdf]

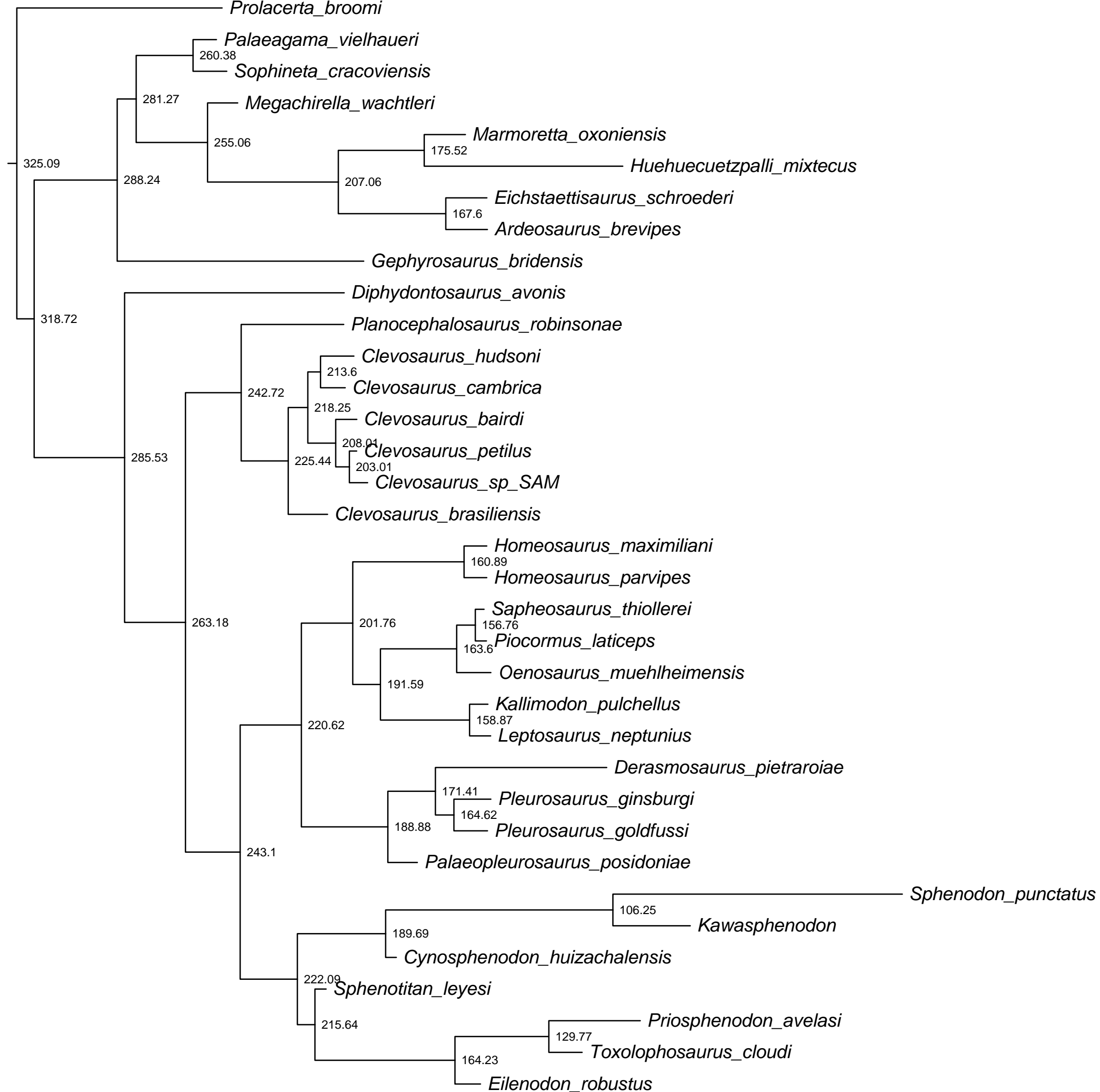

40.0

Supplement: Supplementary file 6 — Additional file 6. Input files including the dataset and all necessary coding (see Mr. Bayes blocks) to reproduce the analyses. [file 12915_2020_901_MOESM6_ESM.zip › InputFiles&OutputTrees/BayesCalibrated/Diversity(NoSA)/BayesCal_TK02_ln_p3_StartTr_3per_60G_DvNoSA_LExct/BayesCal_TK02_ln_p3_StartTr_3per_DvNoSA_AllCom.t.con.tre_Age.pdf]

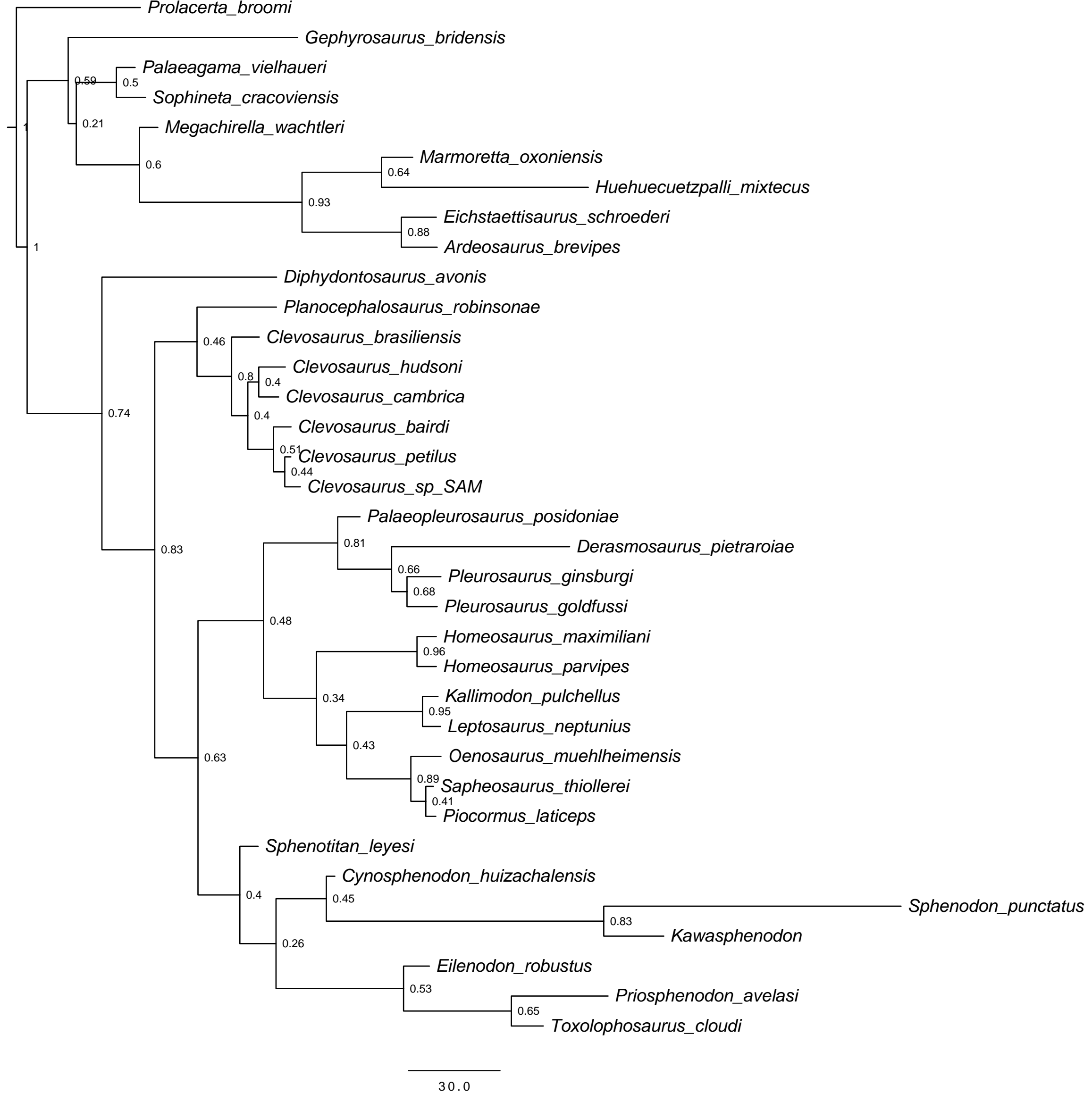

Supplement: Supplementary file 6 — Additional file 6. Input files including the dataset and all necessary coding (see Mr. Bayes blocks) to reproduce the analyses. [file 12915_2020_901_MOESM6_ESM.zip › InputFiles&OutputTrees/BayesCalibrated/Diversity(NoSA)/BayesCal_TK02_ln_p3_StartTr_3per_60G_DvNoSA_NoR/BayesCal_TK02_ln_p3_StartTr_3per_DvNoSA_AllCom.t.con.tre.pdf]

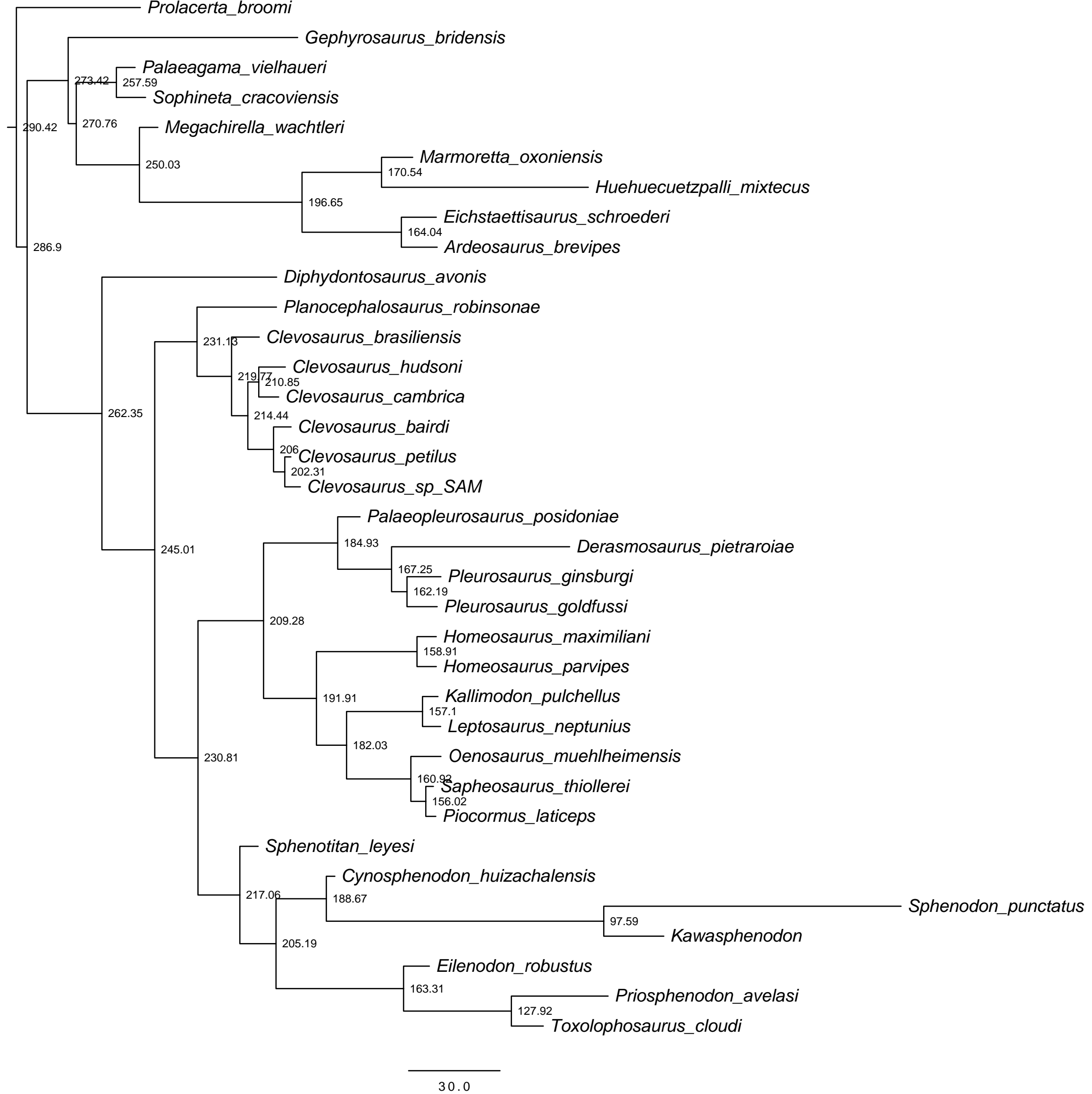

Supplement: Supplementary file 6 — Additional file 6. Input files including the dataset and all necessary coding (see Mr. Bayes blocks) to reproduce the analyses. [file 12915_2020_901_MOESM6_ESM.zip › InputFiles&OutputTrees/BayesCalibrated/Diversity(NoSA)/BayesCal_TK02_ln_p3_StartTr_3per_60G_DvNoSA_NoR/BayesCal_TK02_ln_p3_StartTr_3per_DvNoSA_AllCom.t.con.tre_Age.pdf]

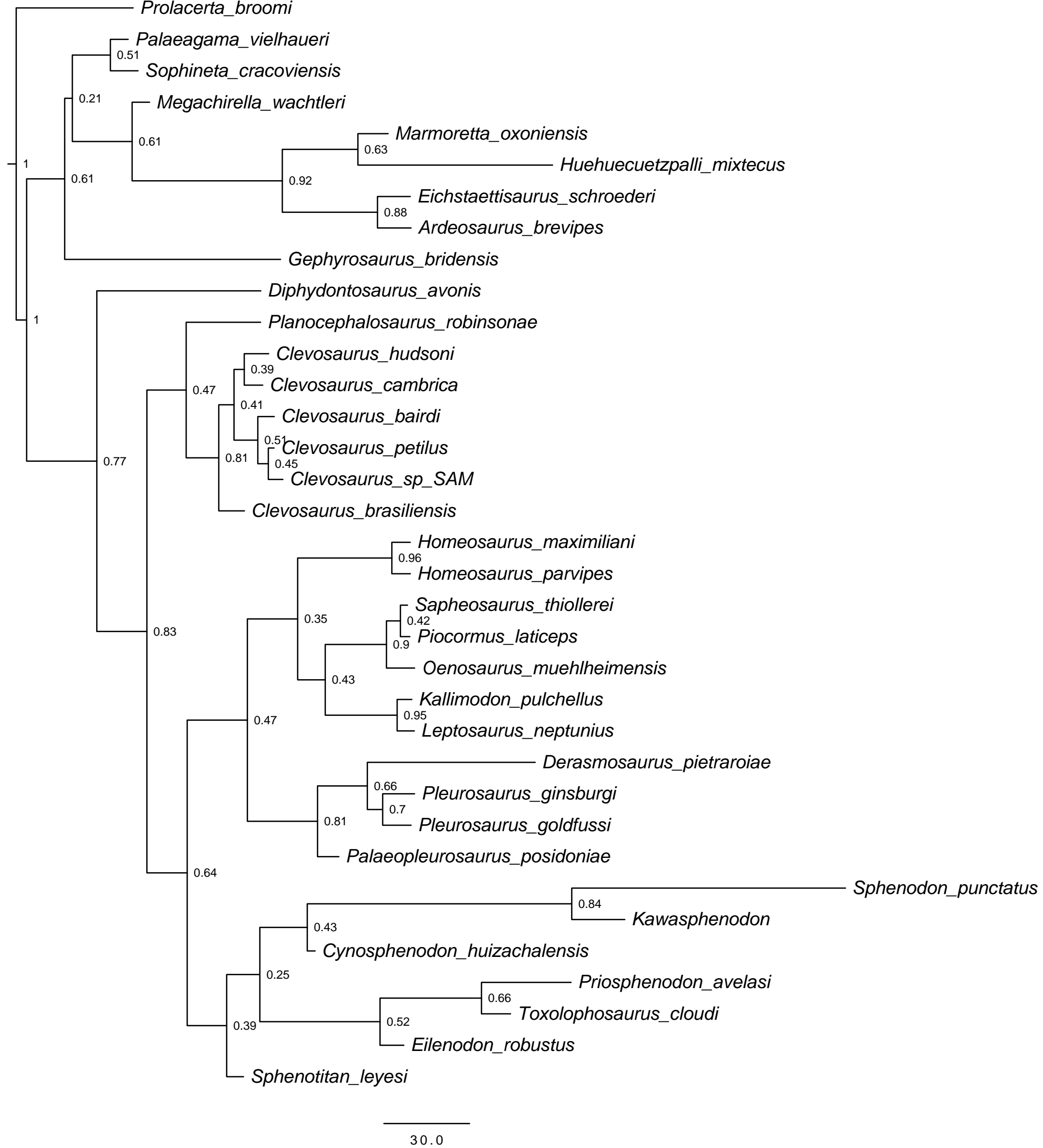

Supplement: Supplementary file 6 — Additional file 6. Input files including the dataset and all necessary coding (see Mr. Bayes blocks) to reproduce the analyses. [file 12915_2020_901_MOESM6_ESM.zip › InputFiles&OutputTrees/BayesCalibrated/Diversity(NoSA)/BayesCal_TK02_ln_p3_StartTr_3per_60G_DvNoSA_NoR_LExct/BayesCal_TK02_ln_p3_StartTr_3per_DvNoSA_NoR_LExct_AllCom.t.con.tre.pdf]

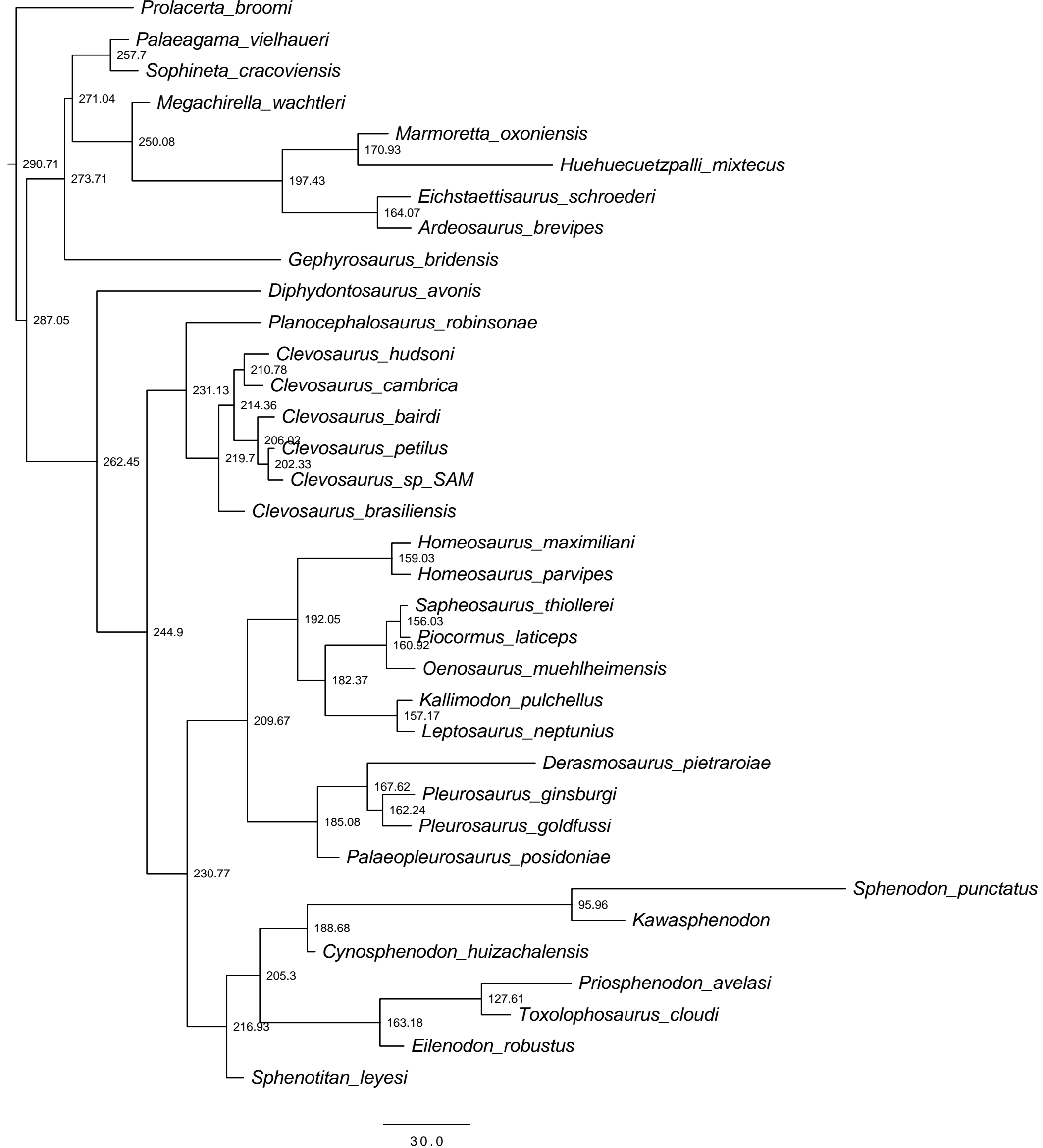

Supplement: Supplementary file 6 — Additional file 6. Input files including the dataset and all necessary coding (see Mr. Bayes blocks) to reproduce the analyses. [file 12915_2020_901_MOESM6_ESM.zip › InputFiles&OutputTrees/BayesCalibrated/Diversity(NoSA)/BayesCal_TK02_ln_p3_StartTr_3per_60G_DvNoSA_NoR_LExct/BayesCal_TK02_ln_p3_StartTr_3per_DvNoSA_NoR_LExct_AllCom.t.con.tre_Age.pdf]

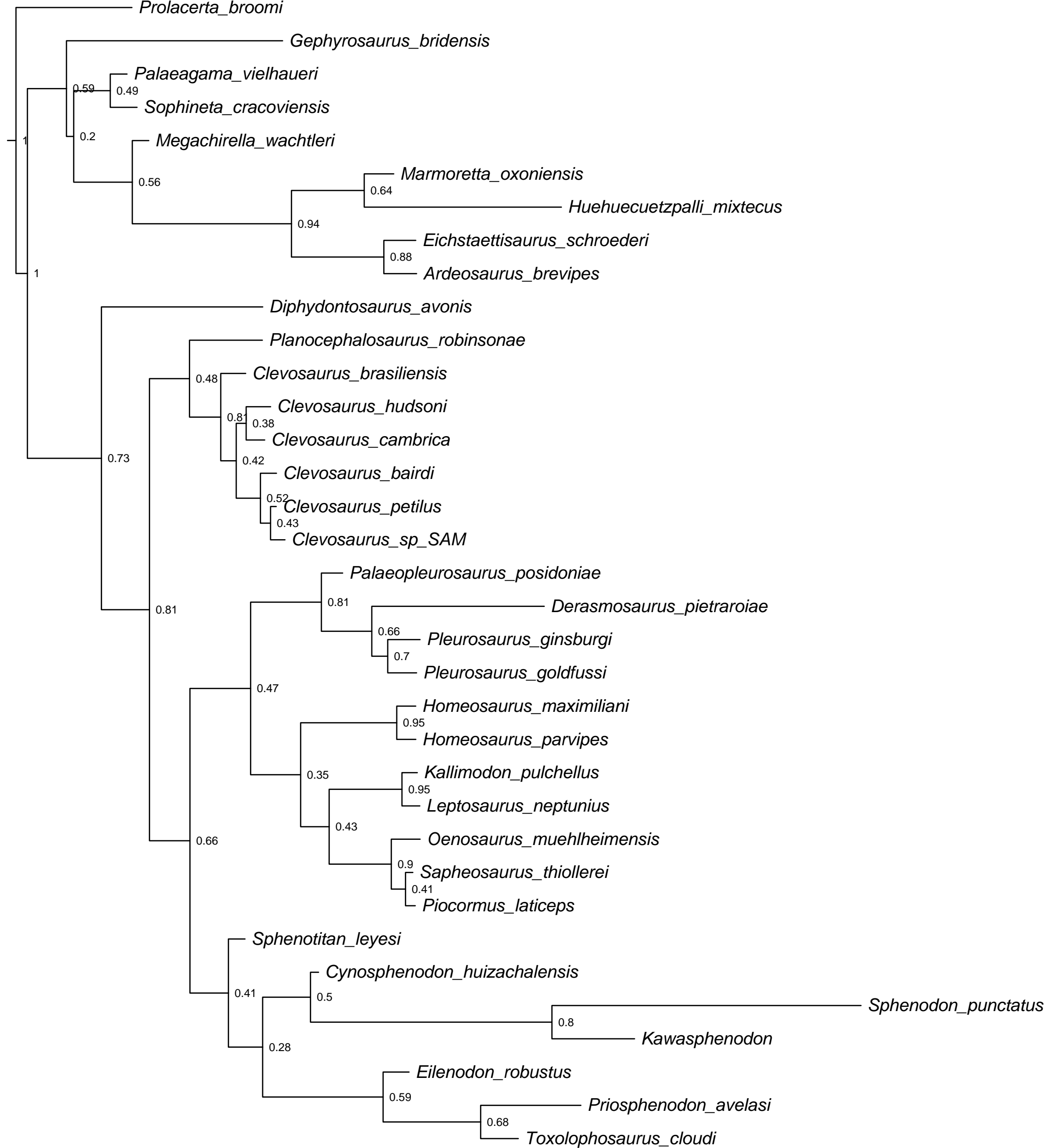

Supplement: Supplementary file 6 — Additional file 6. Input files including the dataset and all necessary coding (see Mr. Bayes blocks) to reproduce the analyses. [file 12915_2020_901_MOESM6_ESM.zip › InputFiles&OutputTrees/BayesCalibrated/Diversity(NoSA)/BayesCal_TK02_ln_p3_StartTr_3per_60G_DvNoSA_SFBD(s)2l_NoR/BayesCal_TK02_ln_p3_StartTr_3per_DvNoSA_SFBD2_AllCom.t.con.tre.pdf]

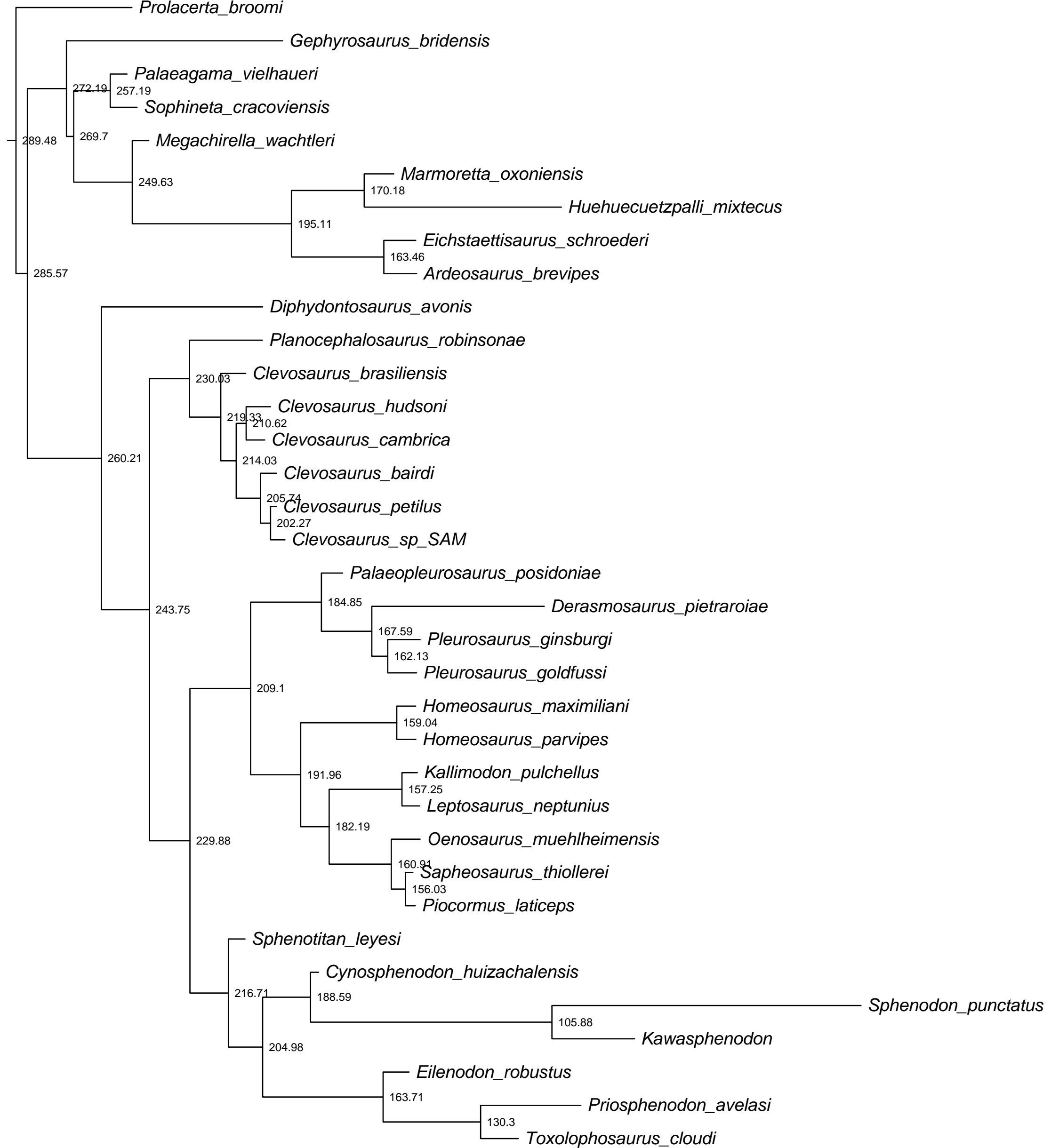

30.0

Supplement: Supplementary file 6 — Additional file 6. Input files including the dataset and all necessary coding (see Mr. Bayes blocks) to reproduce the analyses. [file 12915_2020_901_MOESM6_ESM.zip › InputFiles&OutputTrees/BayesCalibrated/Diversity(NoSA)/BayesCal_TK02_ln_p3_StartTr_3per_60G_DvNoSA_SFBD(s)2l_NoR/BayesCal_TK02_ln_p3_StartTr_3per_DvNoSA_SFBD2_AllCom.t.con.tre_Age.pdf]

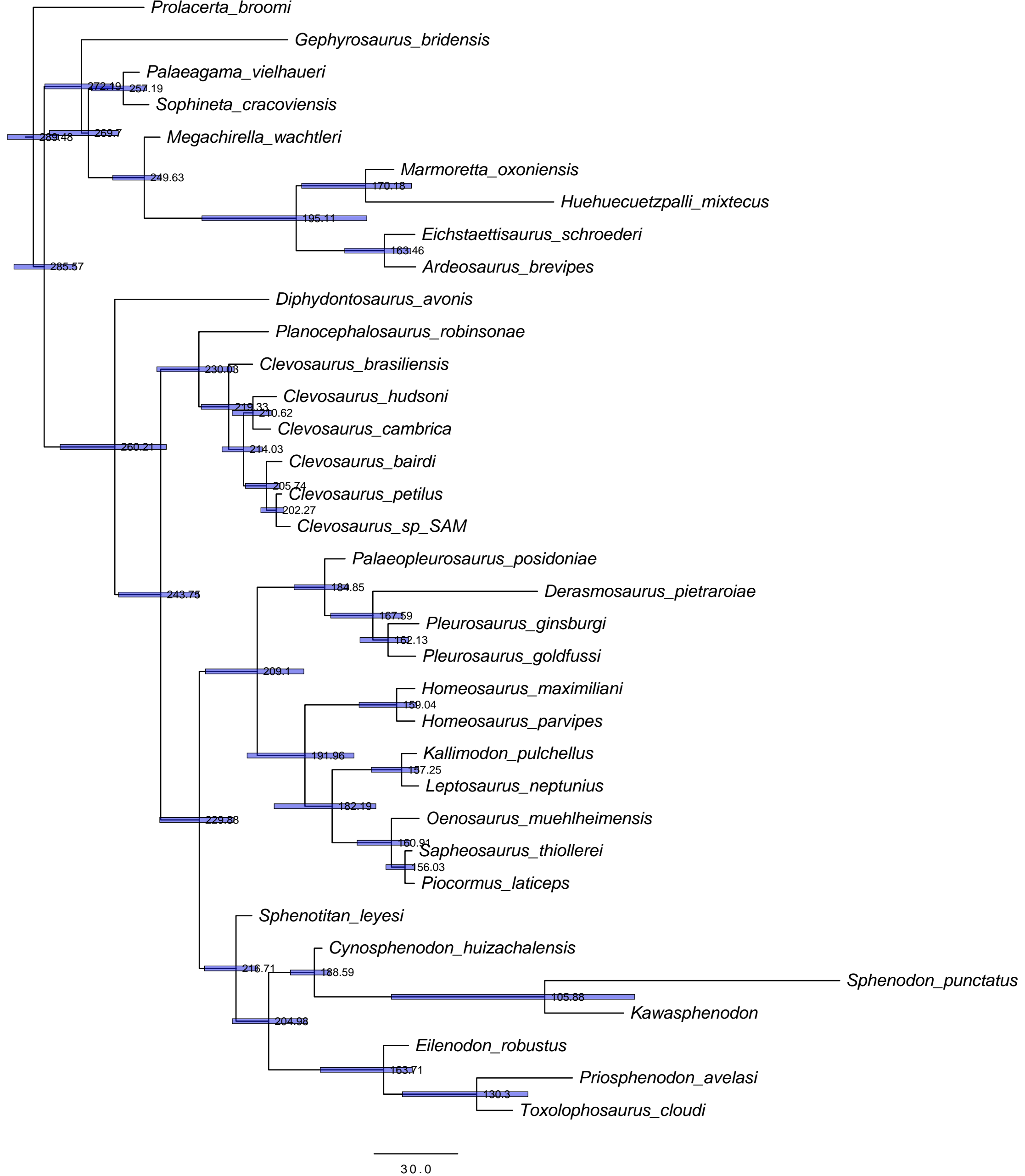

Supplement: Supplementary file 6 — Additional file 6. Input files including the dataset and all necessary coding (see Mr. Bayes blocks) to reproduce the analyses. [file 12915_2020_901_MOESM6_ESM.zip › InputFiles&OutputTrees/BayesCalibrated/Diversity(NoSA)/BayesCal_TK02_ln_p3_StartTr_3per_60G_DvNoSA_SFBD(s)2l_NoR/BayesCal_TK02_ln_p3_StartTr_3per_DvNoSA_SFBD2_AllCom.t.con.tre_AgeBars.pdf]

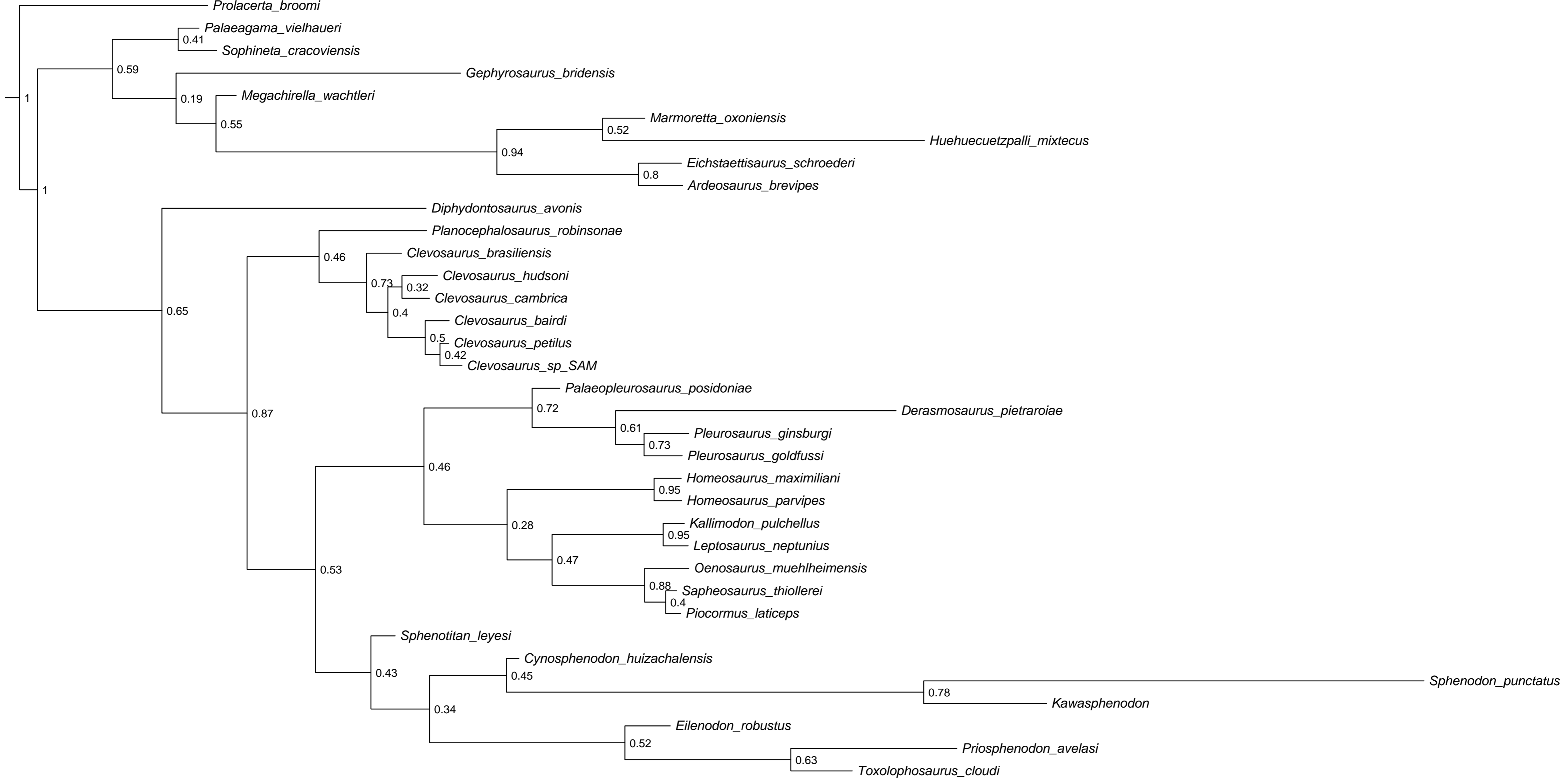

Supplement: Supplementary file 6 — Additional file 6. Input files including the dataset and all necessary coding (see Mr. Bayes blocks) to reproduce the analyses. [file 12915_2020_901_MOESM6_ESM.zip › InputFiles&OutputTrees/BayesCalibrated/Diversity(NoSA)/BayesCal_TK02_ln_p3_StartTr_3per_60G_DvNoSA_SFBD(s)2l_NoR_Asym/BayesCal_TK02_ln_p3_StartTr_3per_DvNoSA_SFBD2_Asym_AllCom.t.con.tre.pdf]

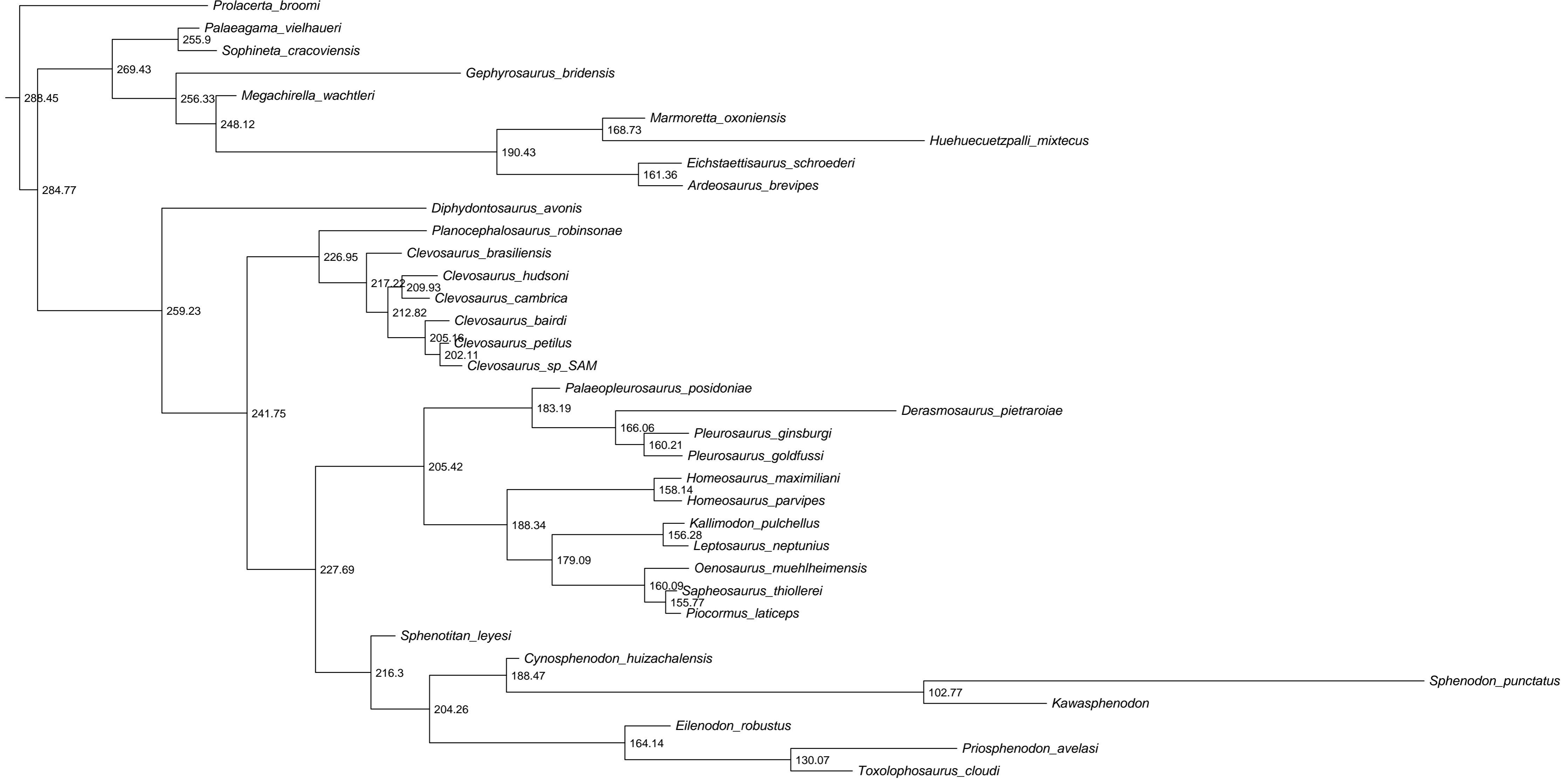

30.0

Supplement: Supplementary file 6 — Additional file 6. Input files including the dataset and all necessary coding (see Mr. Bayes blocks) to reproduce the analyses. [file 12915_2020_901_MOESM6_ESM.zip › InputFiles&OutputTrees/BayesCalibrated/Diversity(NoSA)/BayesCal_TK02_ln_p3_StartTr_3per_60G_DvNoSA_SFBD(s)2l_NoR_Asym/BayesCal_TK02_ln_p3_StartTr_3per_DvNoSA_SFBD2_Asym_AllCom.t.con.tre_Ages.pdf]

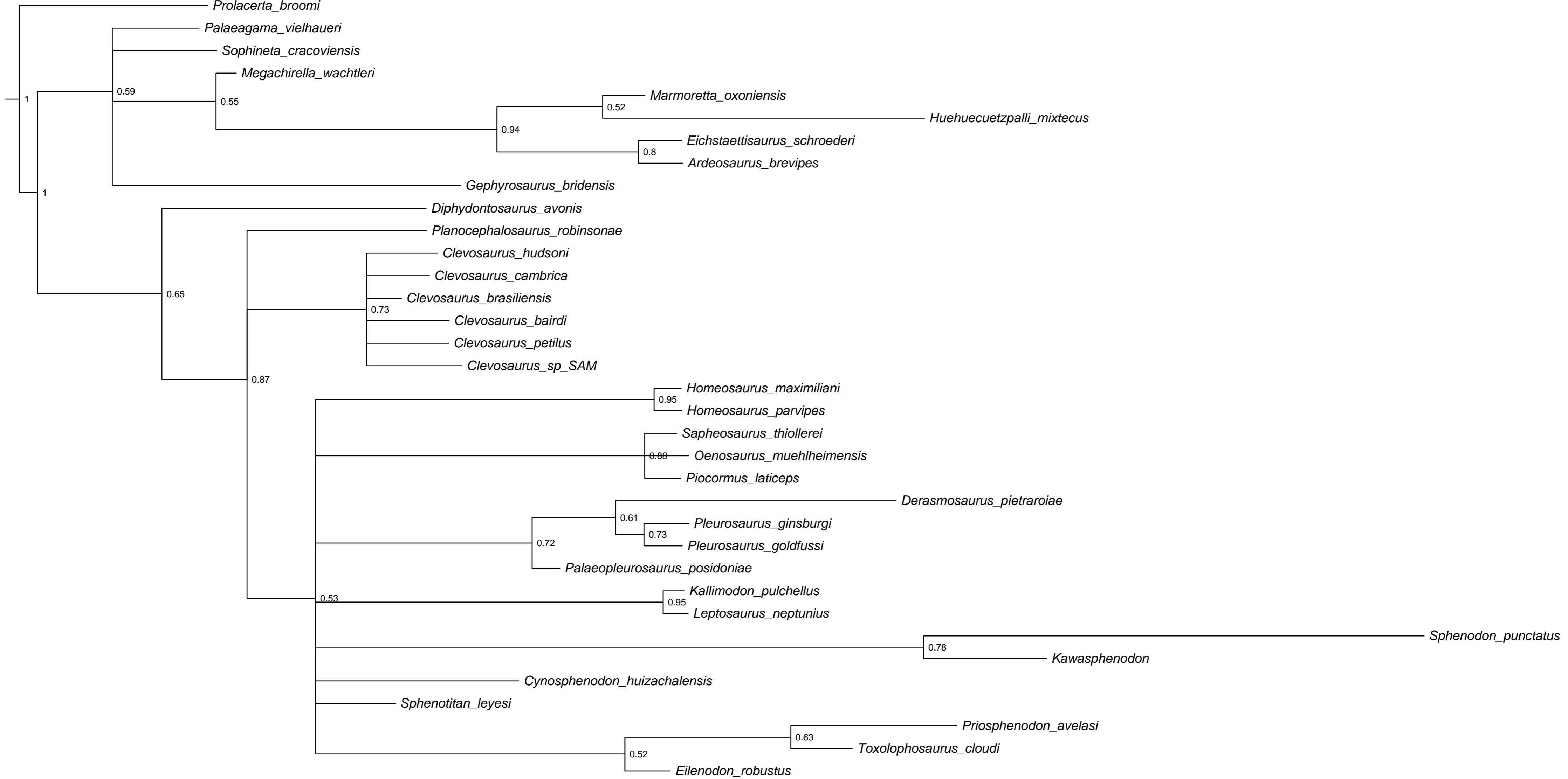

Supplement: Supplementary file 6 — Additional file 6. Input files including the dataset and all necessary coding (see Mr. Bayes blocks) to reproduce the analyses. [file 12915_2020_901_MOESM6_ESM.zip › InputFiles&OutputTrees/BayesCalibrated/Diversity(NoSA)/BayesCal_TK02_ln_p3_StartTr_3per_60G_DvNoSA_SFBD(s)2l_NoR_Asym/BayesCal_TK02_ln_p3_StartTr_3per_DvNoSA_SFBD2_Asym_MRC.t.con.tre.pdf]

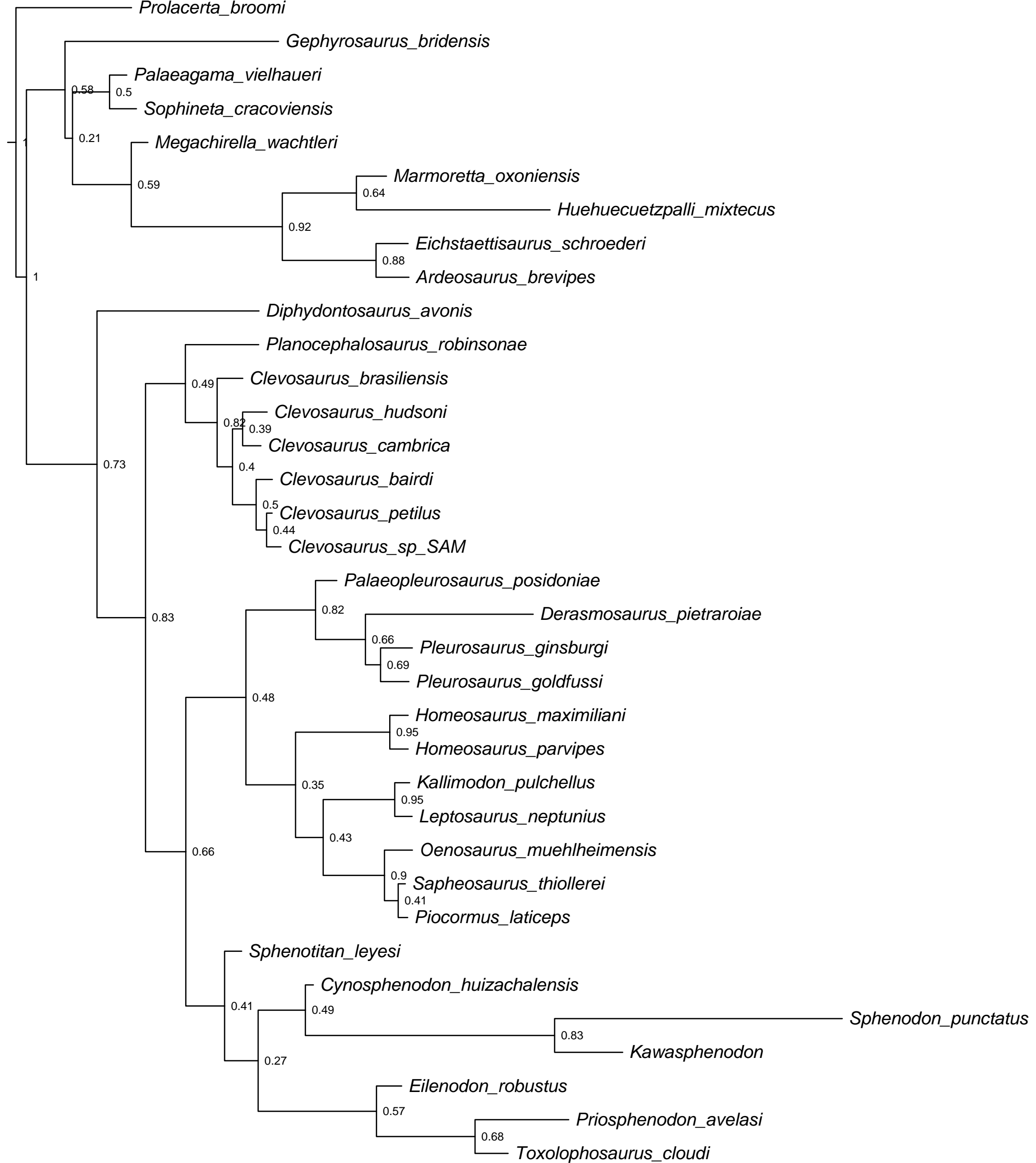

30.0

Supplement: Supplementary file 6 — Additional file 6. Input files including the dataset and all necessary coding (see Mr. Bayes blocks) to reproduce the analyses. [file 12915_2020_901_MOESM6_ESM.zip › InputFiles&OutputTrees/BayesCalibrated/Diversity(NoSA)/BayesCal_TK02_ln_p3_StartTr_3per_60G_DvNoSA_SFBD(s)2l_NoR_LExct/BayesCal_TK02_ln_p3_StartTr_3per_DvNoSA_SFBD2_NoR_LExct_AllCom.t.con.tre.pdf]

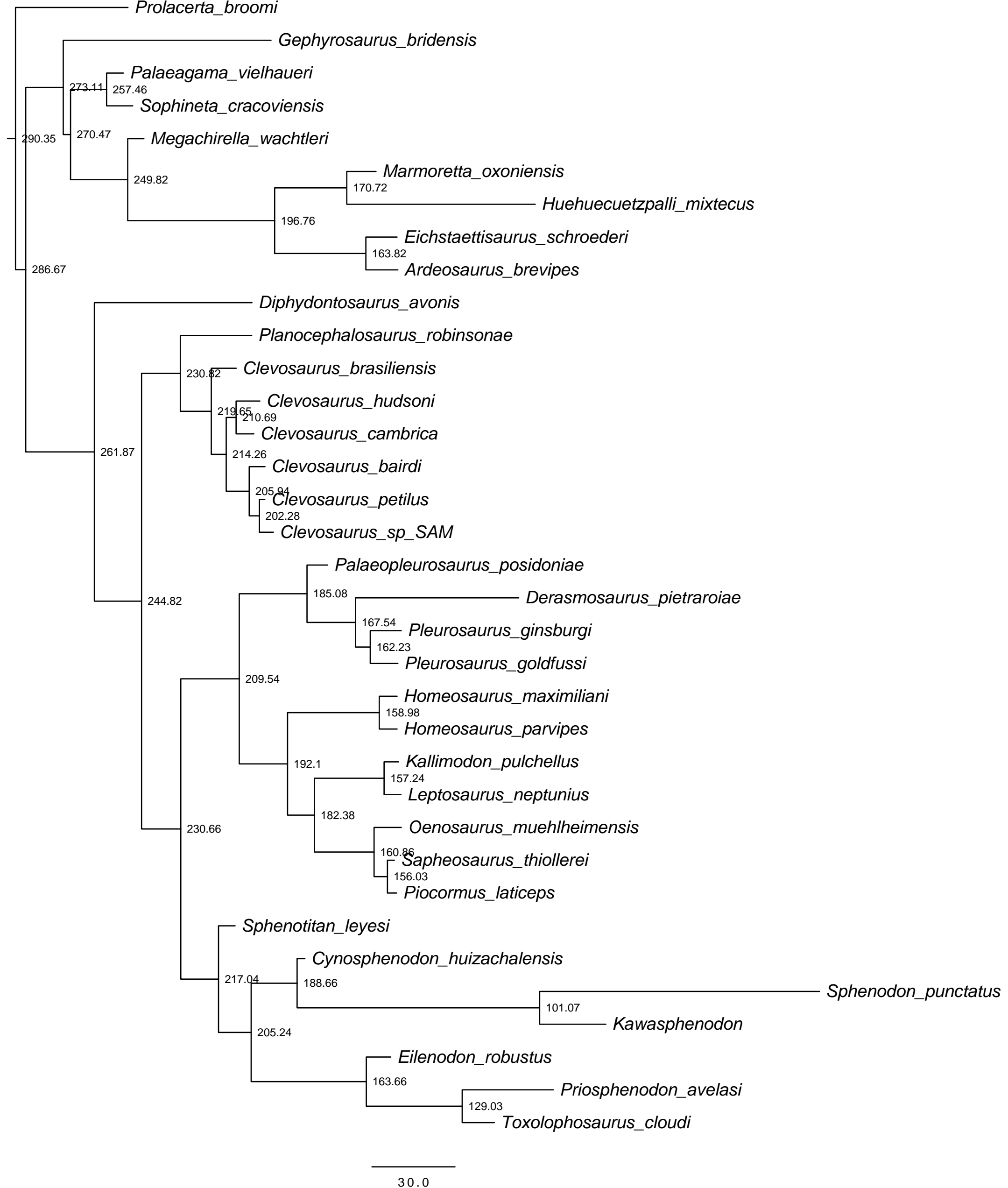

Supplement: Supplementary file 6 — Additional file 6. Input files including the dataset and all necessary coding (see Mr. Bayes blocks) to reproduce the analyses. [file 12915_2020_901_MOESM6_ESM.zip › InputFiles&OutputTrees/BayesCalibrated/Diversity(NoSA)/BayesCal_TK02_ln_p3_StartTr_3per_60G_DvNoSA_SFBD(s)2l_NoR_LExct/BayesCal_TK02_ln_p3_StartTr_3per_DvNoSA_SFBD2_NoR_LExct_AllCom.t.con.tre_Age.pdf]

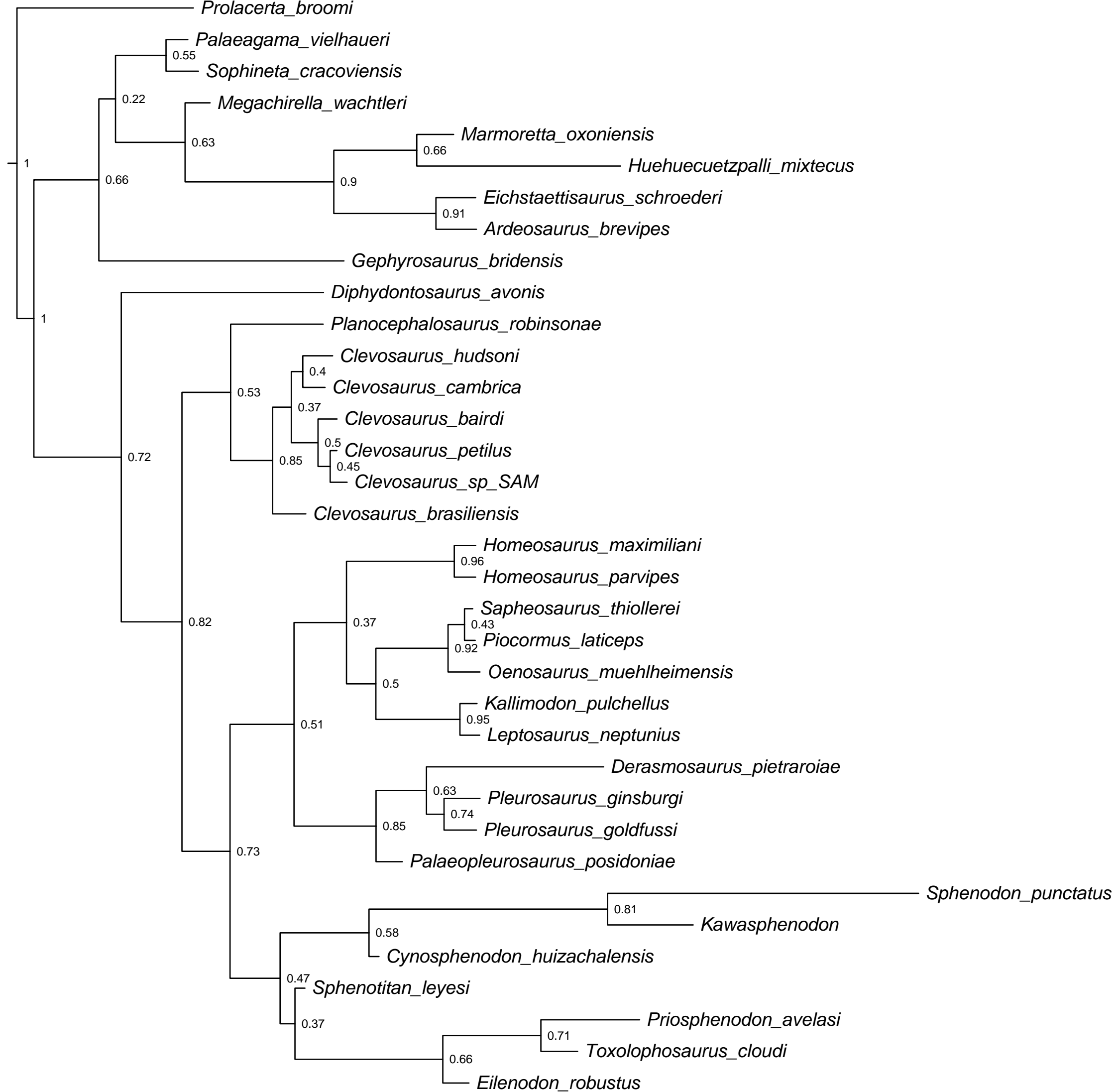

40.0

Supplement: Supplementary file 6 — Additional file 6. Input files including the dataset and all necessary coding (see Mr. Bayes blocks) to reproduce the analyses. [file 12915_2020_901_MOESM6_ESM.zip › InputFiles&OutputTrees/BayesCalibrated/Diversity(NoSA)/BayesCal_TK02_ln_p3_StartTr_3per_60G_DvNoSA_SFBD(s)2_2l/BayesCal_TK02_ln_p3_StartTr_3per_DvNoSA_SFBD2_AllCom.t.con.tre.pdf]

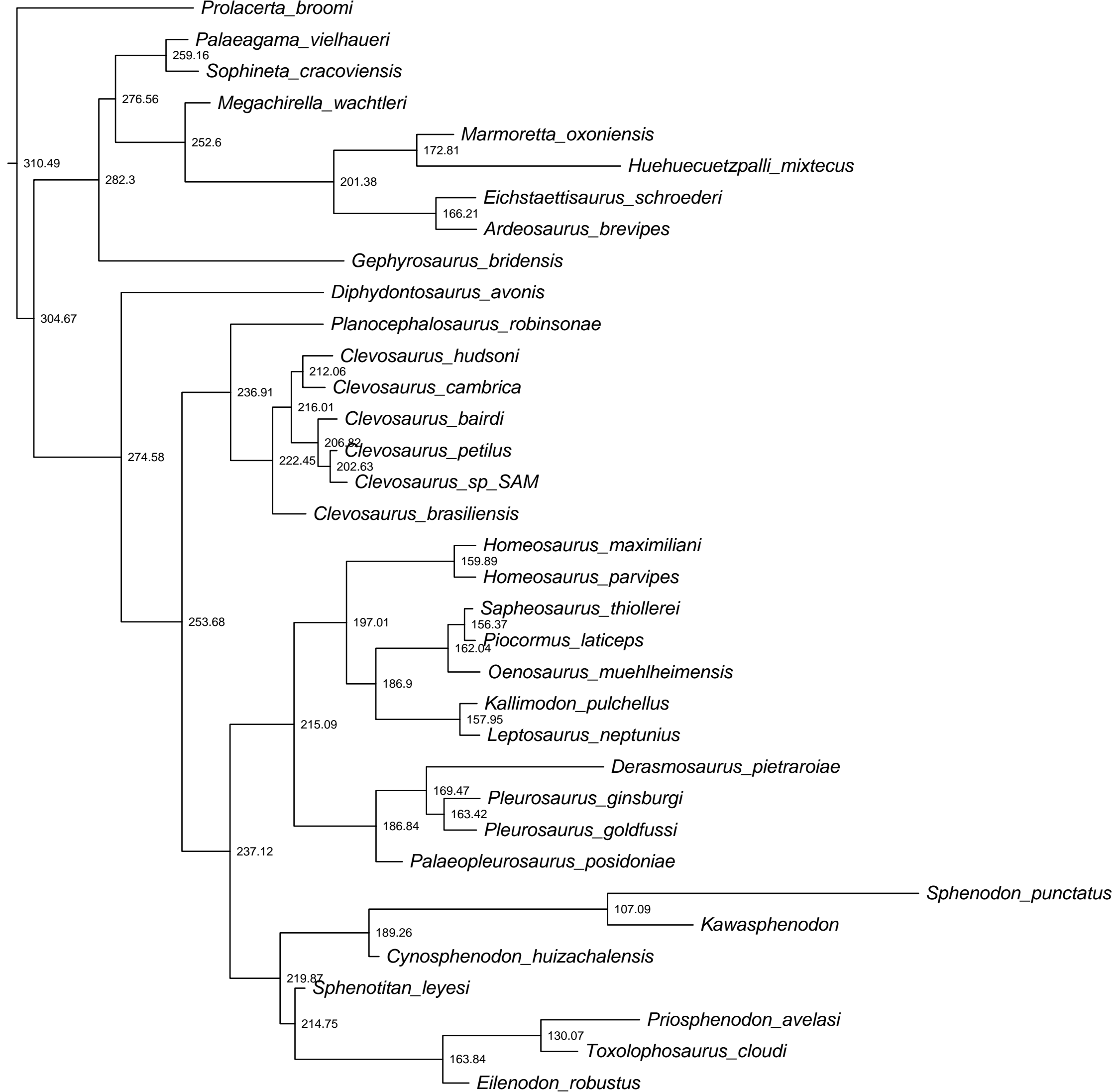

Supplement: Supplementary file 6 — Additional file 6. Input files including the dataset and all necessary coding (see Mr. Bayes blocks) to reproduce the analyses. [file 12915_2020_901_MOESM6_ESM.zip › InputFiles&OutputTrees/BayesCalibrated/Diversity(NoSA)/BayesCal_TK02_ln_p3_StartTr_3per_60G_DvNoSA_SFBD(s)2_2l/BayesCal_TK02_ln_p3_StartTr_3per_DvNoSA_SFBD2_AllCom.t.con.tre_Age.pdf]

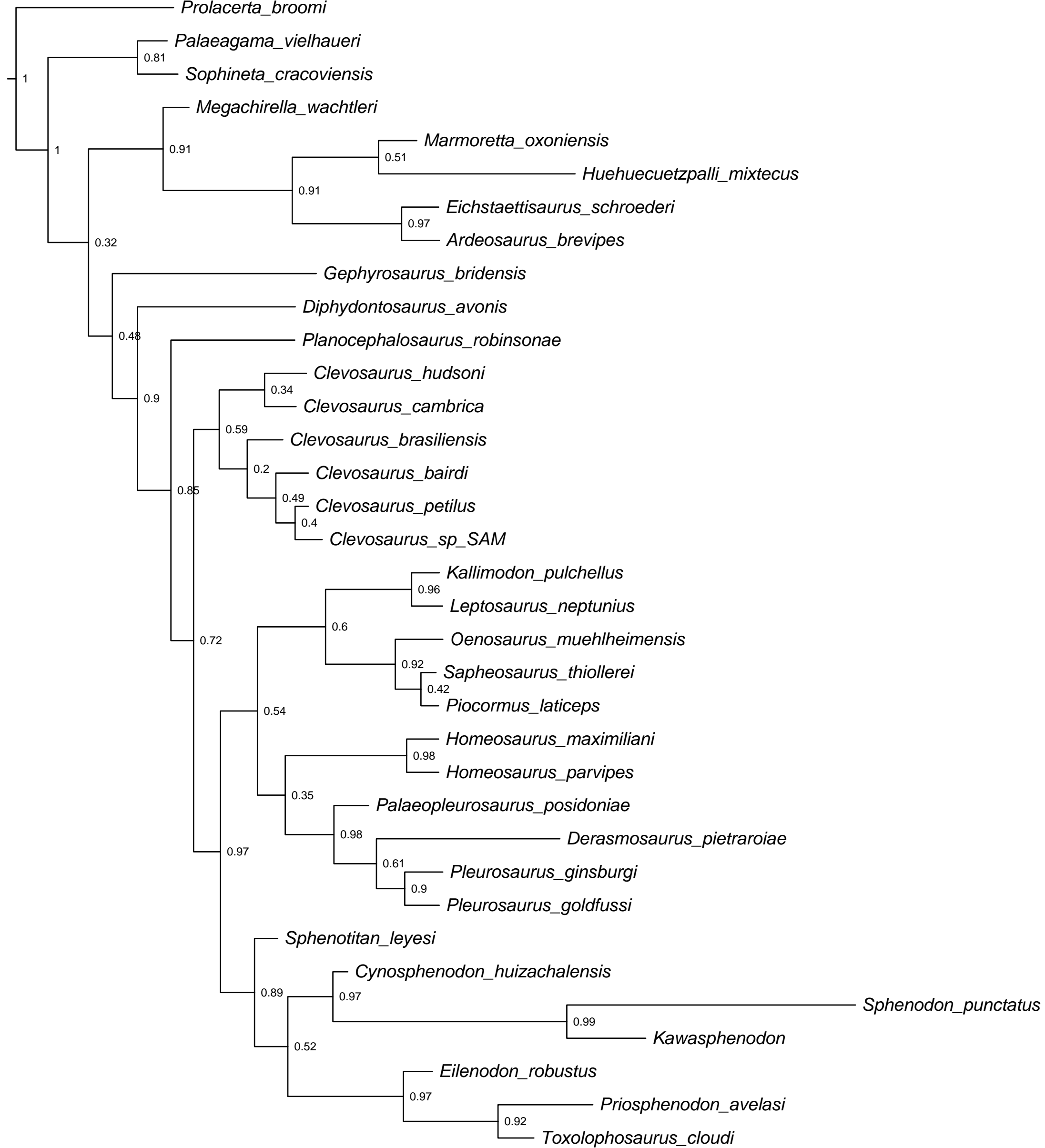

40.0

Supplement: Supplementary file 6 — Additional file 6. Input files including the dataset and all necessary coding (see Mr. Bayes blocks) to reproduce the analyses. [file 12915_2020_901_MOESM6_ESM.zip › InputFiles&OutputTrees/BayesCalibrated/FossilTips/BayesCal_IGR_ln_p1_60G_FT/BayesCal_IGR_ln_p1_FT_AllCom.t.con.tre.pdf]

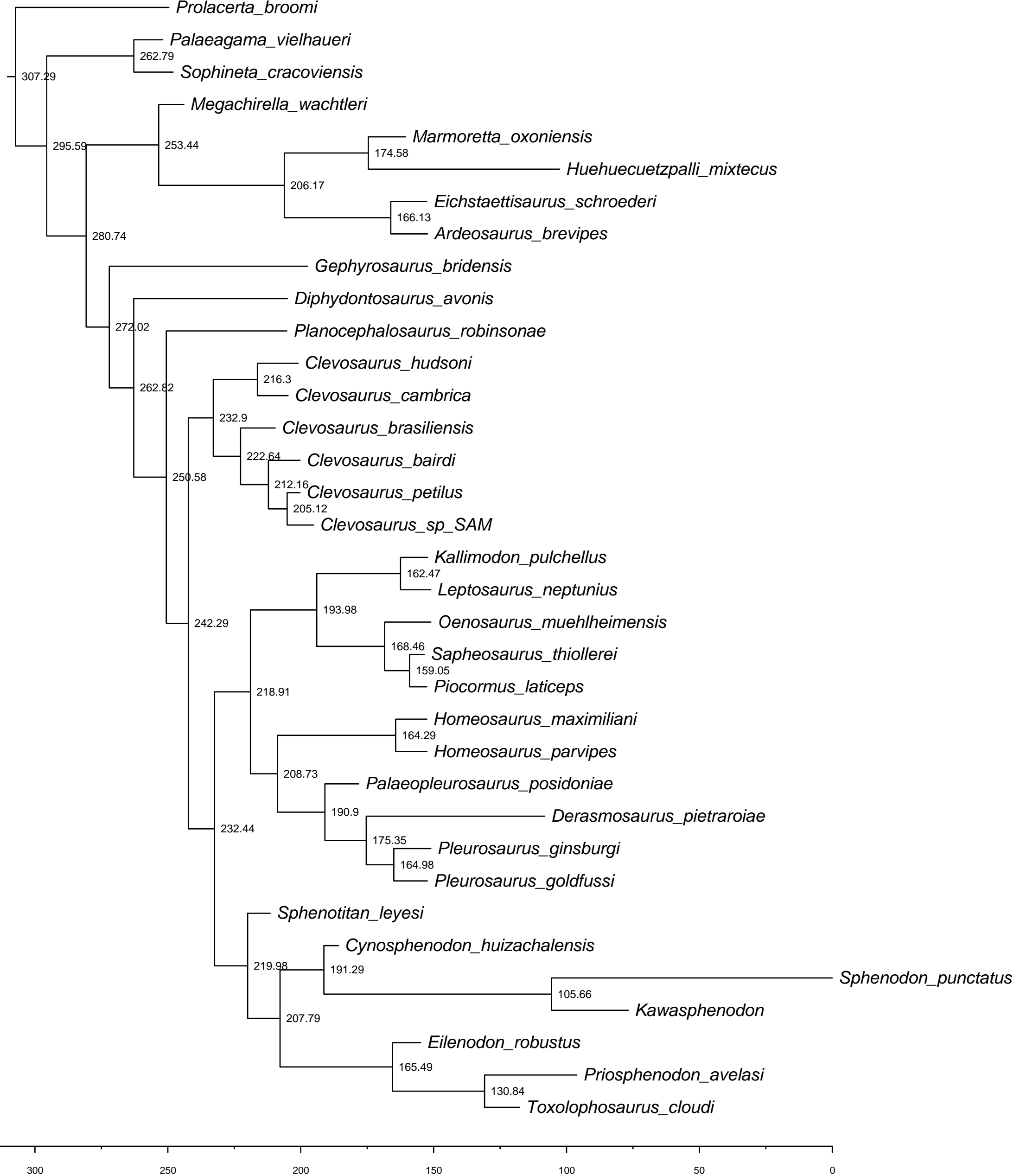

Supplement: Supplementary file 6 — Additional file 6. Input files including the dataset and all necessary coding (see Mr. Bayes blocks) to reproduce the analyses. [file 12915_2020_901_MOESM6_ESM.zip › InputFiles&OutputTrees/BayesCalibrated/FossilTips/BayesCal_IGR_ln_p1_60G_FT/BayesCal_IGR_ln_p1_FT_AllCom.t.con.tre_Age.pdf]

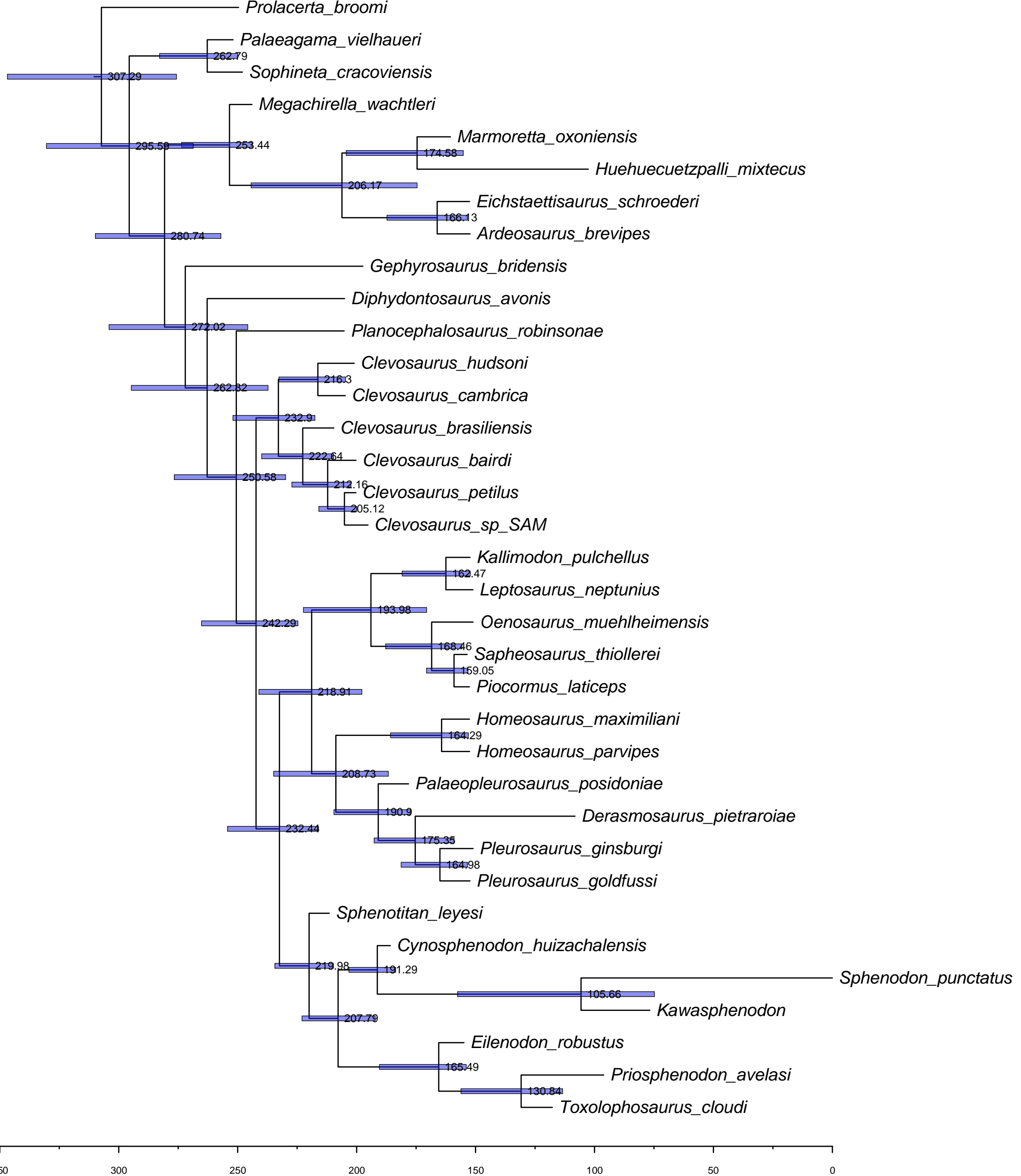

Supplement: Supplementary file 6 — Additional file 6. Input files including the dataset and all necessary coding (see Mr. Bayes blocks) to reproduce the analyses. [file 12915_2020_901_MOESM6_ESM.zip › InputFiles&OutputTrees/BayesCalibrated/FossilTips/BayesCal_IGR_ln_p1_60G_FT/BayesCal_IGR_ln_p1_FT_AllCom.t.con.tre_AgeBars.pdf]

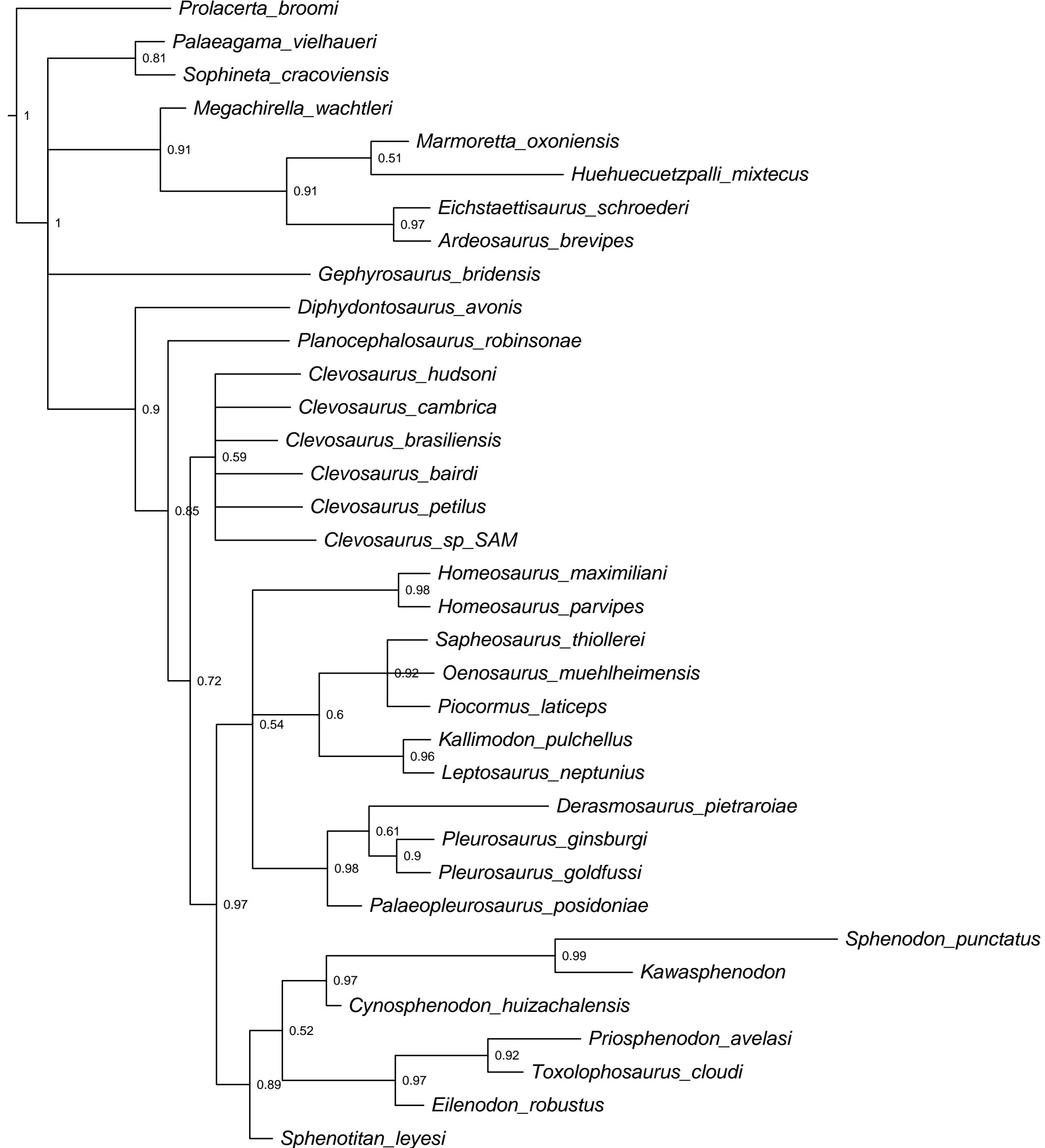

40.0

Supplement: Supplementary file 6 — Additional file 6. Input files including the dataset and all necessary coding (see Mr. Bayes blocks) to reproduce the analyses. [file 12915_2020_901_MOESM6_ESM.zip › InputFiles&OutputTrees/BayesCalibrated/FossilTips/BayesCal_IGR_ln_p1_60G_FT/BayesCal_IGR_ln_p1_FT_MRC.t.con.tre.pdf]

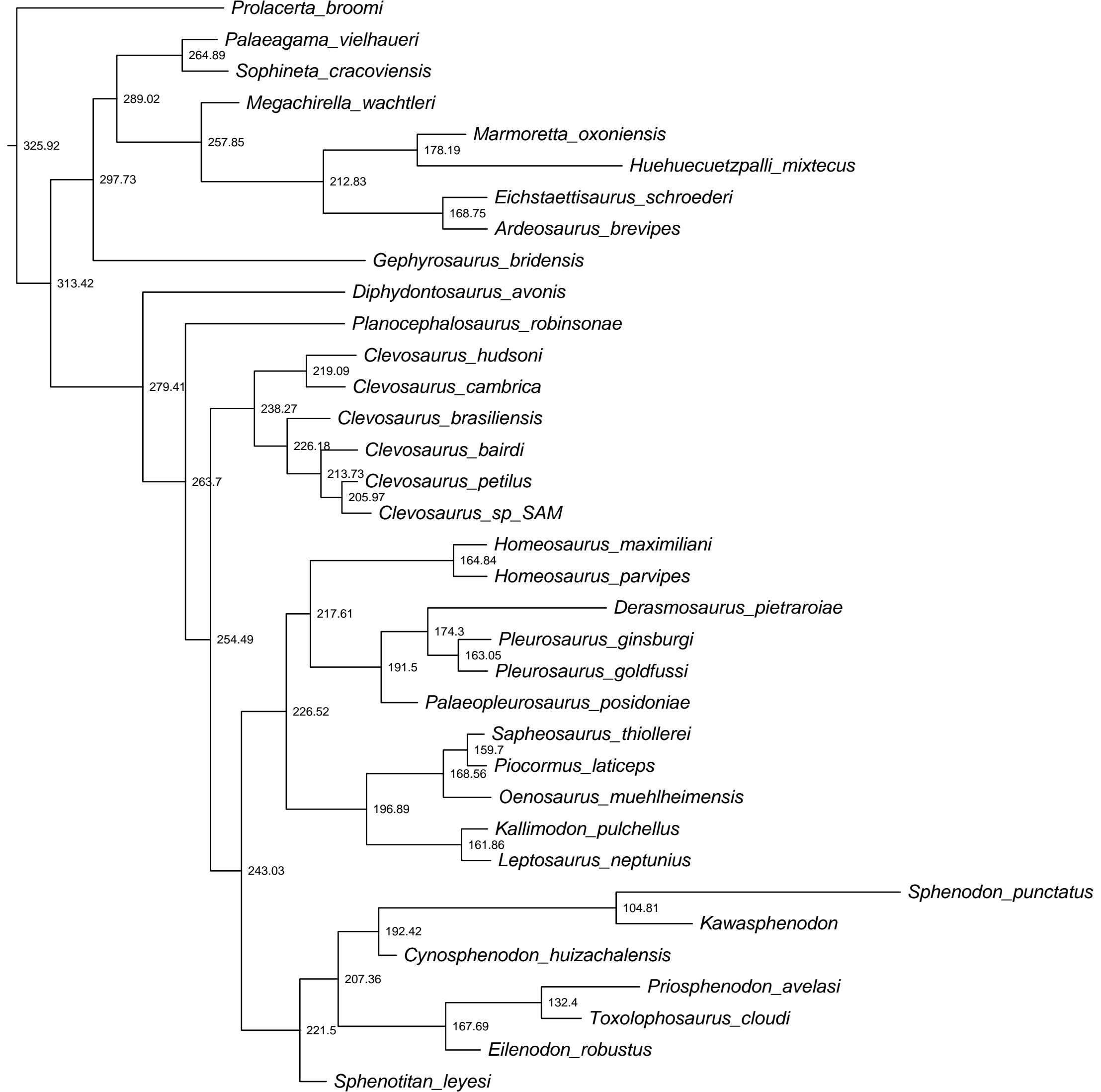

40.0

Supplement: Supplementary file 6 — Additional file 6. Input files including the dataset and all necessary coding (see Mr. Bayes blocks) to reproduce the analyses. [file 12915_2020_901_MOESM6_ESM.zip › InputFiles&OutputTrees/BayesCalibrated/FossilTips/BayesCal_IGR_ln_p3_60G_FT/BayesCal_IGR_FT_ln_p3_60g_AllCom.t.con.tre_Age.pdf]

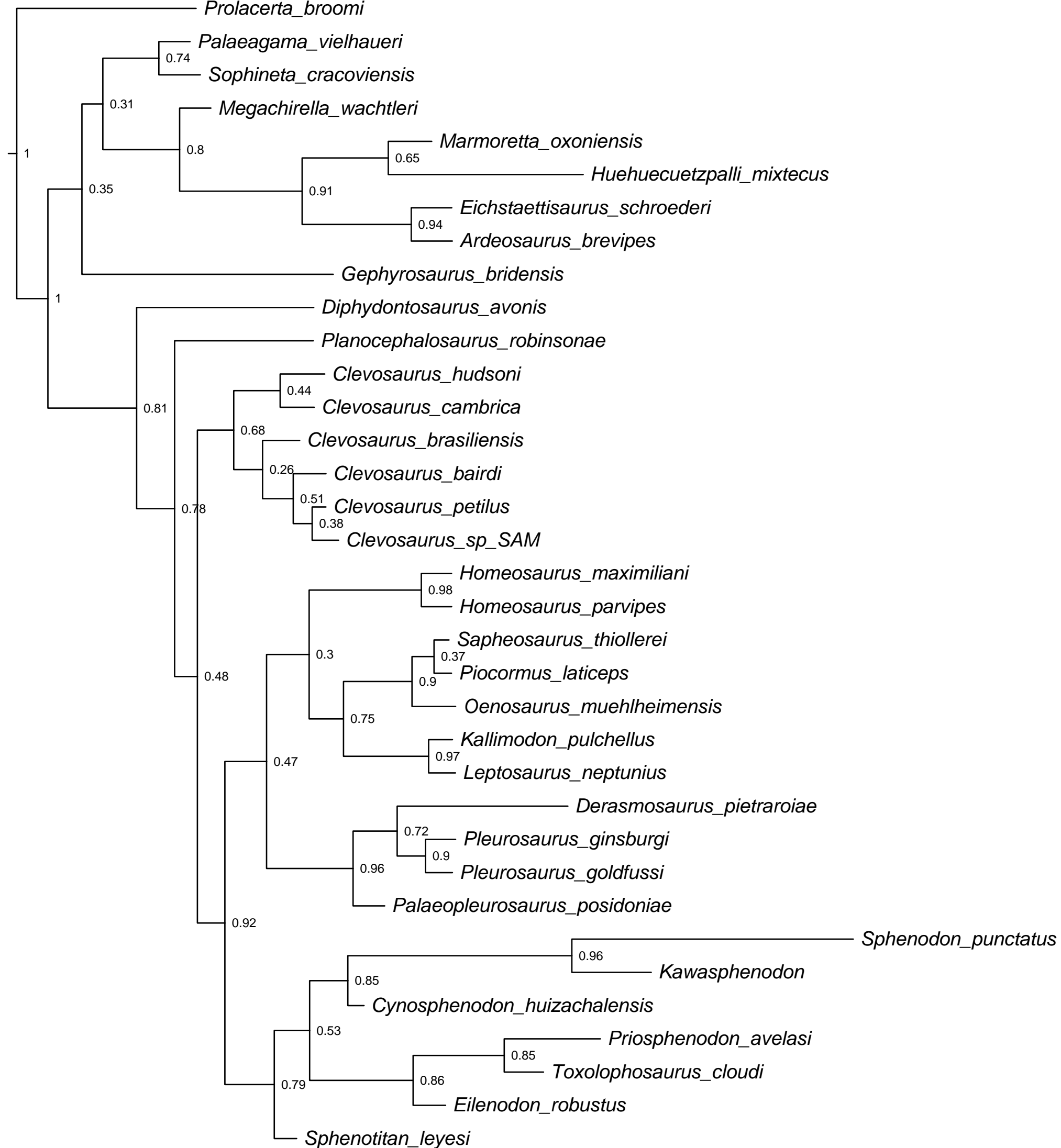

Supplement: Supplementary file 6 — Additional file 6. Input files including the dataset and all necessary coding (see Mr. Bayes blocks) to reproduce the analyses. [file 12915_2020_901_MOESM6_ESM.zip › InputFiles&OutputTrees/BayesCalibrated/FossilTips/BayesCal_IGR_ln_p3_60G_FT_SFBD(s)3l/BayesCal_IGR_ln_p3_60G_FT_SFBD2_AllCom.t.con.tre.pdf]

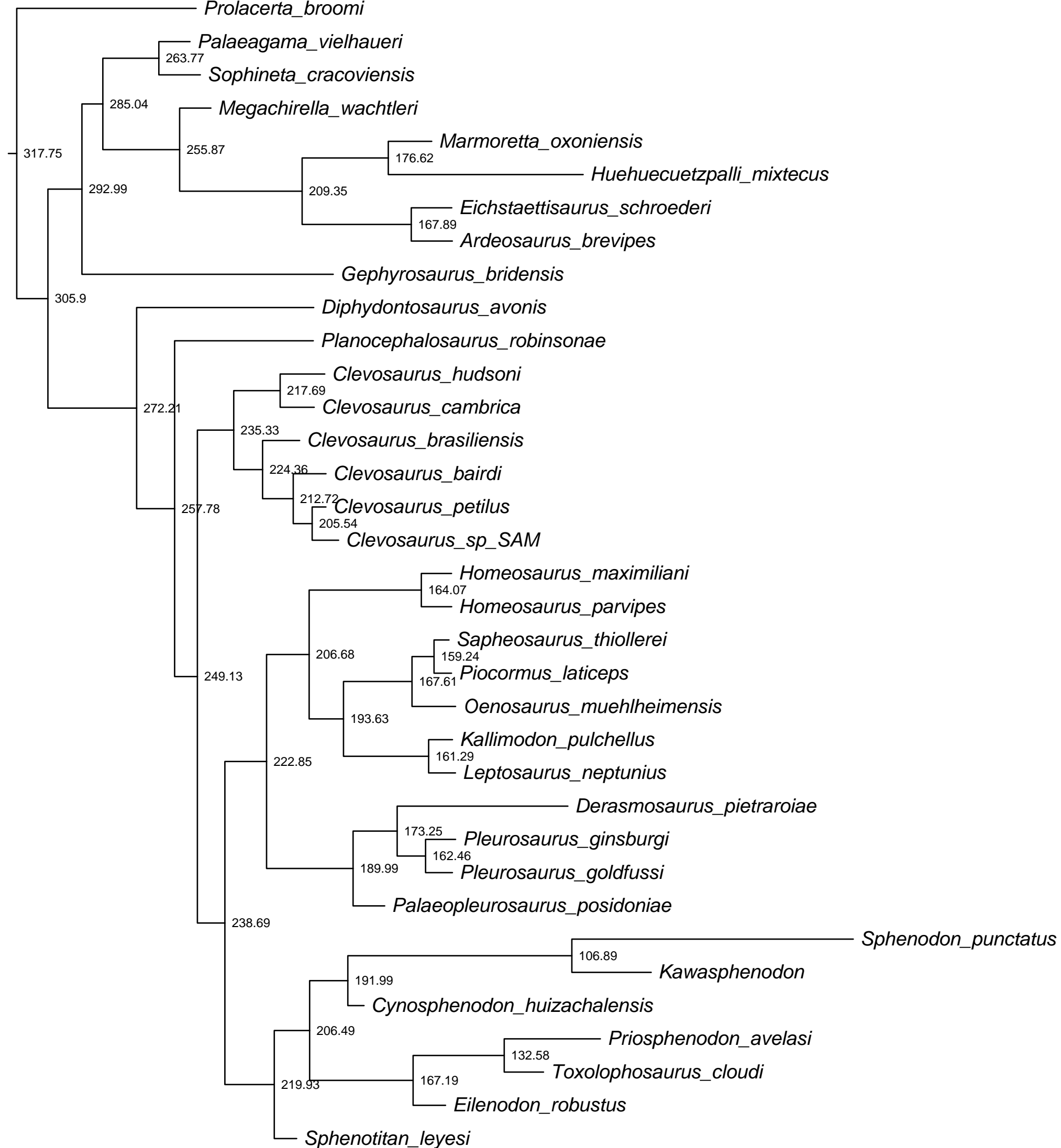

Supplement: Supplementary file 6 — Additional file 6. Input files including the dataset and all necessary coding (see Mr. Bayes blocks) to reproduce the analyses. [file 12915_2020_901_MOESM6_ESM.zip › InputFiles&OutputTrees/BayesCalibrated/FossilTips/BayesCal_IGR_ln_p3_60G_FT_SFBD(s)3l/BayesCal_IGR_ln_p3_60G_FT_SFBD2_AllCom.t.con.tre_Age.pdf]

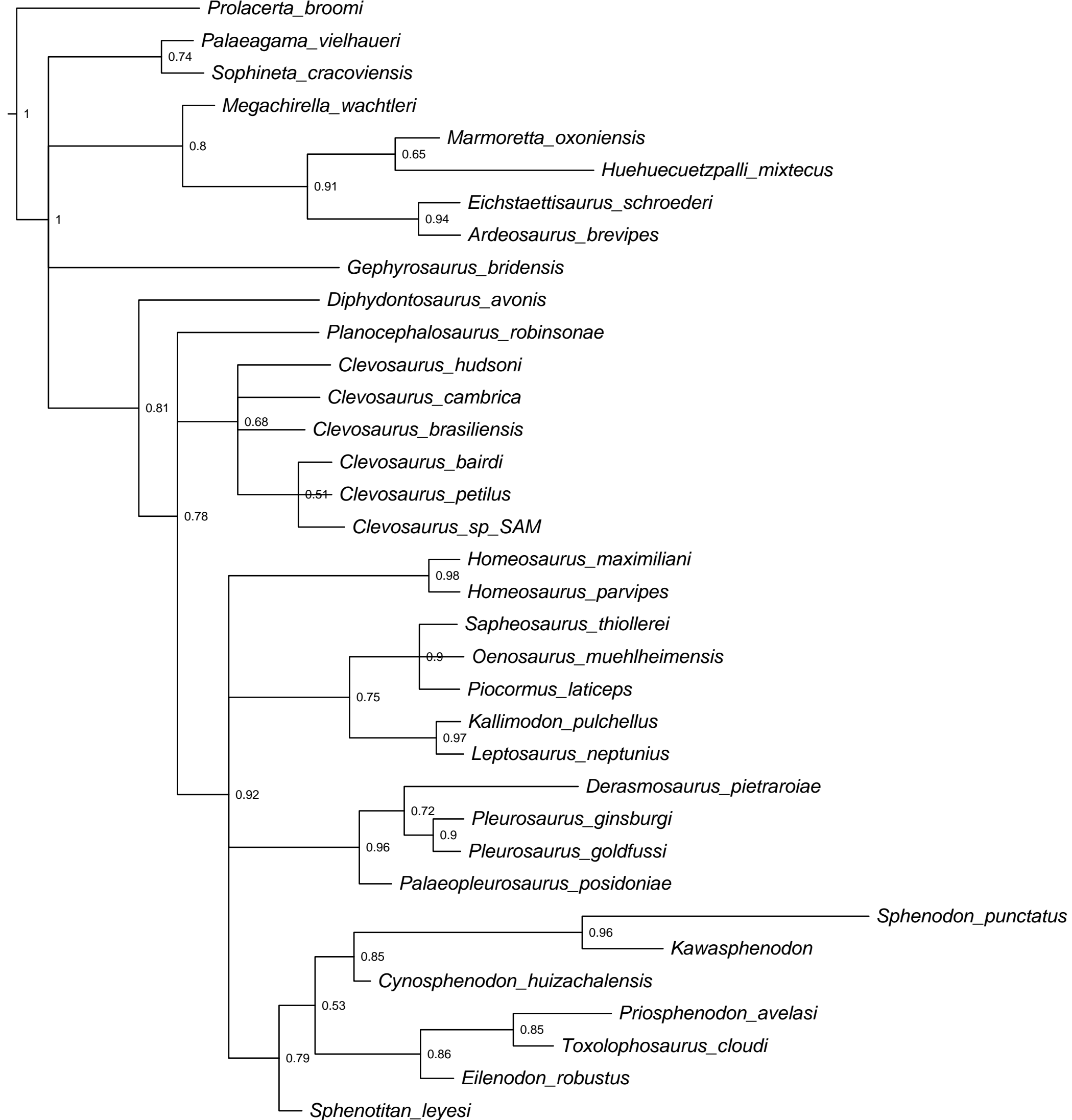

Supplement: Supplementary file 6 — Additional file 6. Input files including the dataset and all necessary coding (see Mr. Bayes blocks) to reproduce the analyses. [file 12915_2020_901_MOESM6_ESM.zip › InputFiles&OutputTrees/BayesCalibrated/FossilTips/BayesCal_IGR_ln_p3_60G_FT_SFBD(s)3l/BayesCal_IGR_ln_p3_60G_FT_SFBD2_MRC.t.con.tre.pdf]

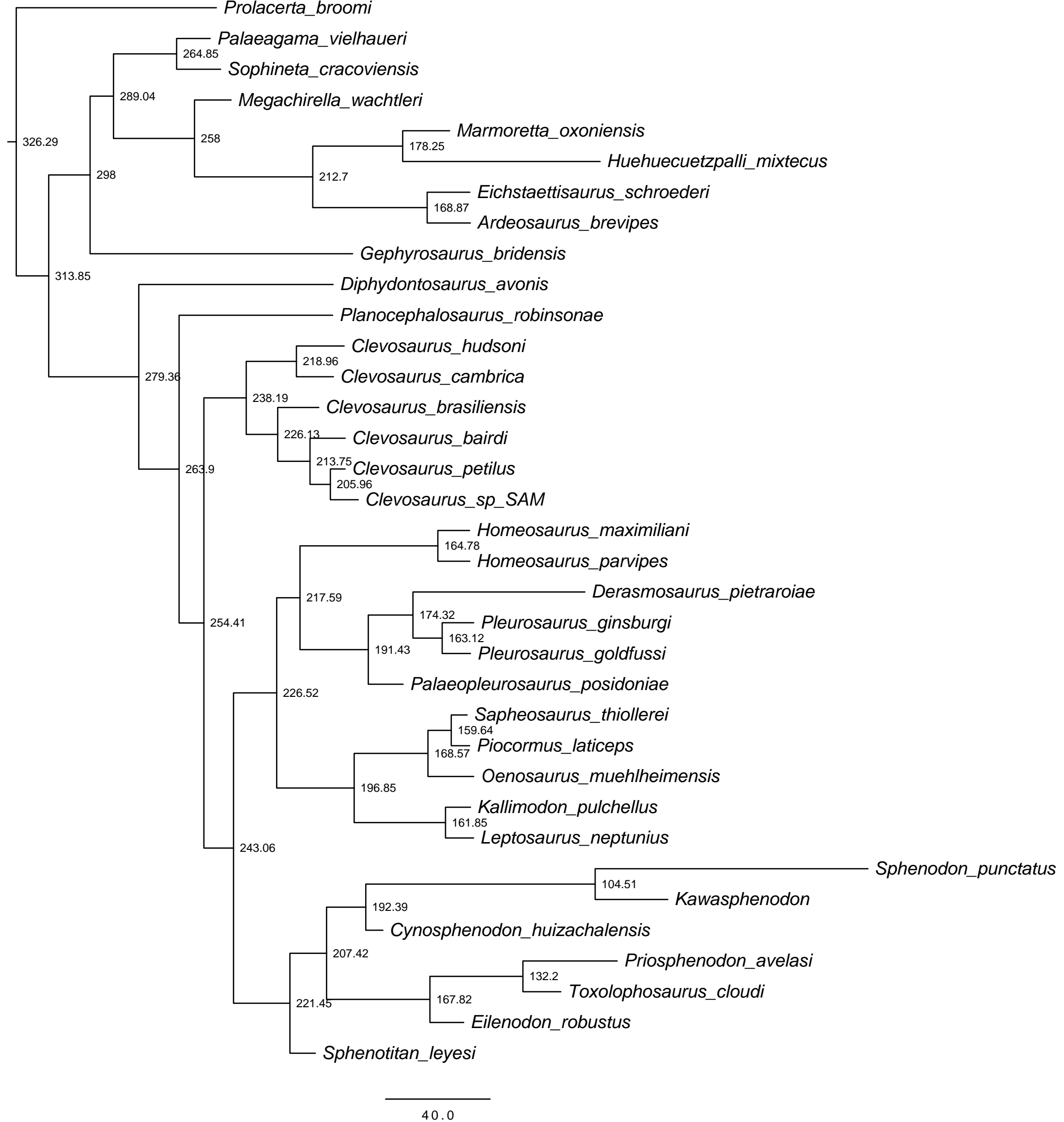

Supplement: Supplementary file 6 — Additional file 6. Input files including the dataset and all necessary coding (see Mr. Bayes blocks) to reproduce the analyses. [file 12915_2020_901_MOESM6_ESM.zip › InputFiles&OutputTrees/BayesCalibrated/FossilTips/BayesCal_IGR_ln_p3_60G_StartTr_3per_FT/BayesCal_IGR_ln_FT_Start_p3_3per_60g_AllCom.t.con.tre_Age.pdf]
