# Supplementary material for: Sphenodontian phylogeny and the impact of model choice in Bayesian morphological clock estimates of divergence times and evolutionary rates
Source: BMC Biol. 2020 Dec 7;18:191. doi: 10.1186/s12915-020-00901-5 (PMC7720557; doi:10.1186/s12915-020-00901-5)
Supplement: Supplementary file 6 — Additional file 6. Input files including the dataset and all necessary coding (see Mr. Bayes blocks) to reproduce the analyses. [file 12915_2020_901_MOESM6_ESM.zip › InputFiles&OutputTrees/BayesCalibrated/FossilTips/BayesCal_TK02_ln_p1_60G_FT/BayesCal_TK02_ln_p1_FT_AllCom.t.con.tre_Ages.pdf]

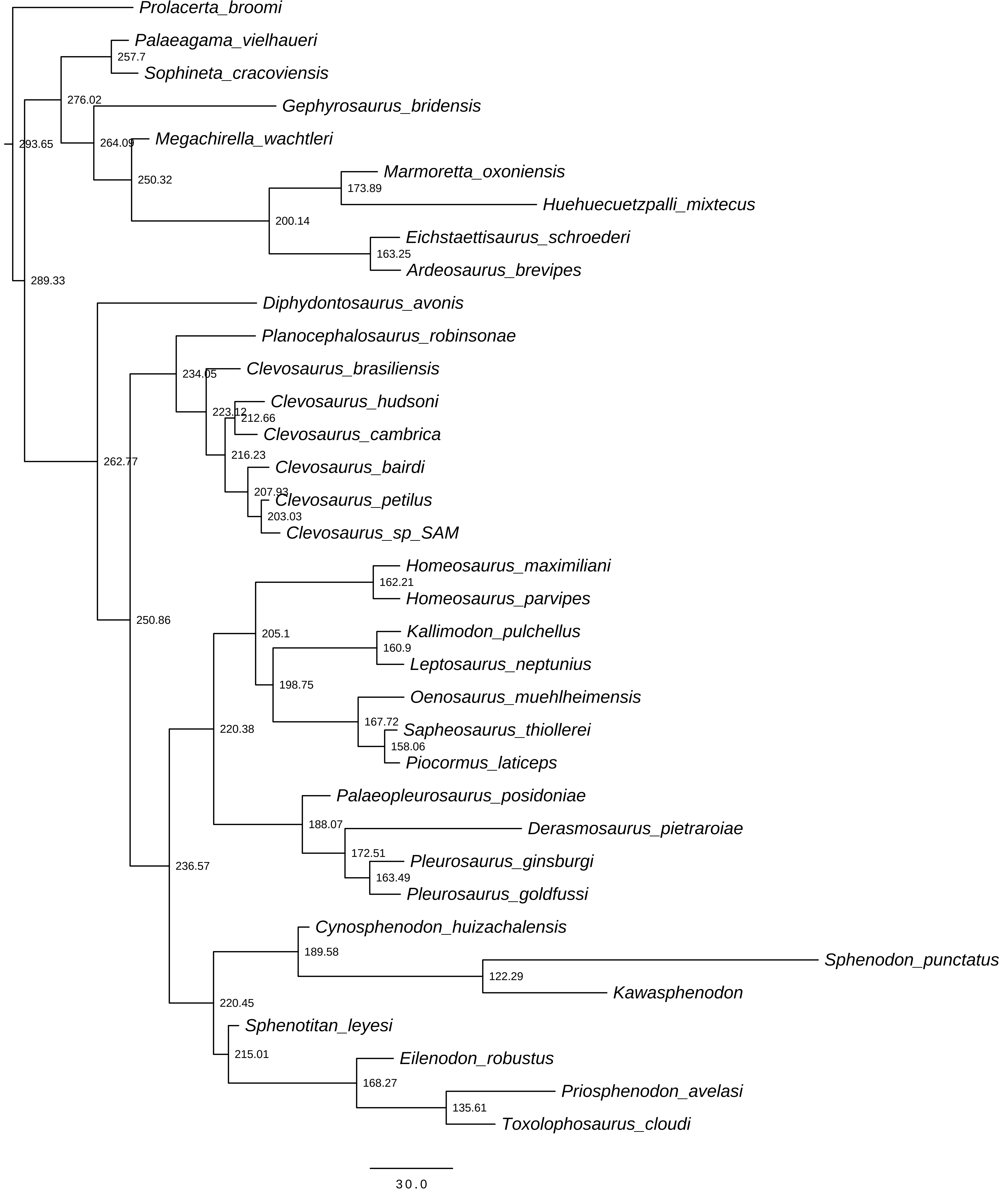
Prolacerta_broomi
Palaeagama_vielhaueri
257.7
Sophineta_cracoviensis
276.02
Gephyrosaurus_bridensis
293.65
264.09
Megachirella_wachtleri
Marmoretta_oxoniensis
250.32
173.89
Huehuecuetzpalli_mixtecus
200.14
Eichstaettisaurus_schroederi
163.25
Ardeosaurus_brevipes
289.33
Diphydontosaurus_avonis
Planocephalosaurus_robinsonae
234.05
Clevosaurus_brasiliensis
Clevosaurus_hudsoni
223.12
212.66
Clevosaurus_cambrica
262.77
216.23
Clevosaurus_bairdi
207.93
Clevosaurus_petilus
203.03
Clevosaurus_sp_SAM
Homeosaurus_maximiliani
162.21
Homeosaurus_parvipes
250.86
Kallimodon_pulchellus
205.1
160.9
Leptosaurus_neptunius
198.75
Oenosaurus_muehlheimensis
167.72
220.38
Sapheosaurus_thiollerei
158.06
Piocormus_laticeps
Palaeopleurosaurus_posidoniae
188.07
Derasmosaurus_pietraroiae
172.51
236.57
Pleurosaurus_ginsburgi
163.49
Pleurosaurus_goldfussi
Cynosphenodon_huizachalensis
189.58
Sphenodon_punctatus
122.29
Kawasphenodon
220.45
Sphenotitan_leyesi
215.01
Eilenodon_robustus
168.27
Priosphenodon_avelasi
135.61
Toxolophosaurus_cloudi
30.0
