# Supplementary material for: Sphenodontian phylogeny and the impact of model choice in Bayesian morphological clock estimates of divergence times and evolutionary rates
Source: BMC Biol. 2020 Dec 7;18:191. doi: 10.1186/s12915-020-00901-5 (PMC7720557; doi:10.1186/s12915-020-00901-5)
Supplement: Supplementary file 6 — Additional file 6. Input files including the dataset and all necessary coding (see Mr. Bayes blocks) to reproduce the analyses. [file 12915_2020_901_MOESM6_ESM.zip › InputFiles&OutputTrees/BayesCalibrated/FossilTips/BayesCal_TK02_ln_p1_60G_FT/BayesCal_TK02_ln_p1_FT_MRC.t.con.tre.pdf]

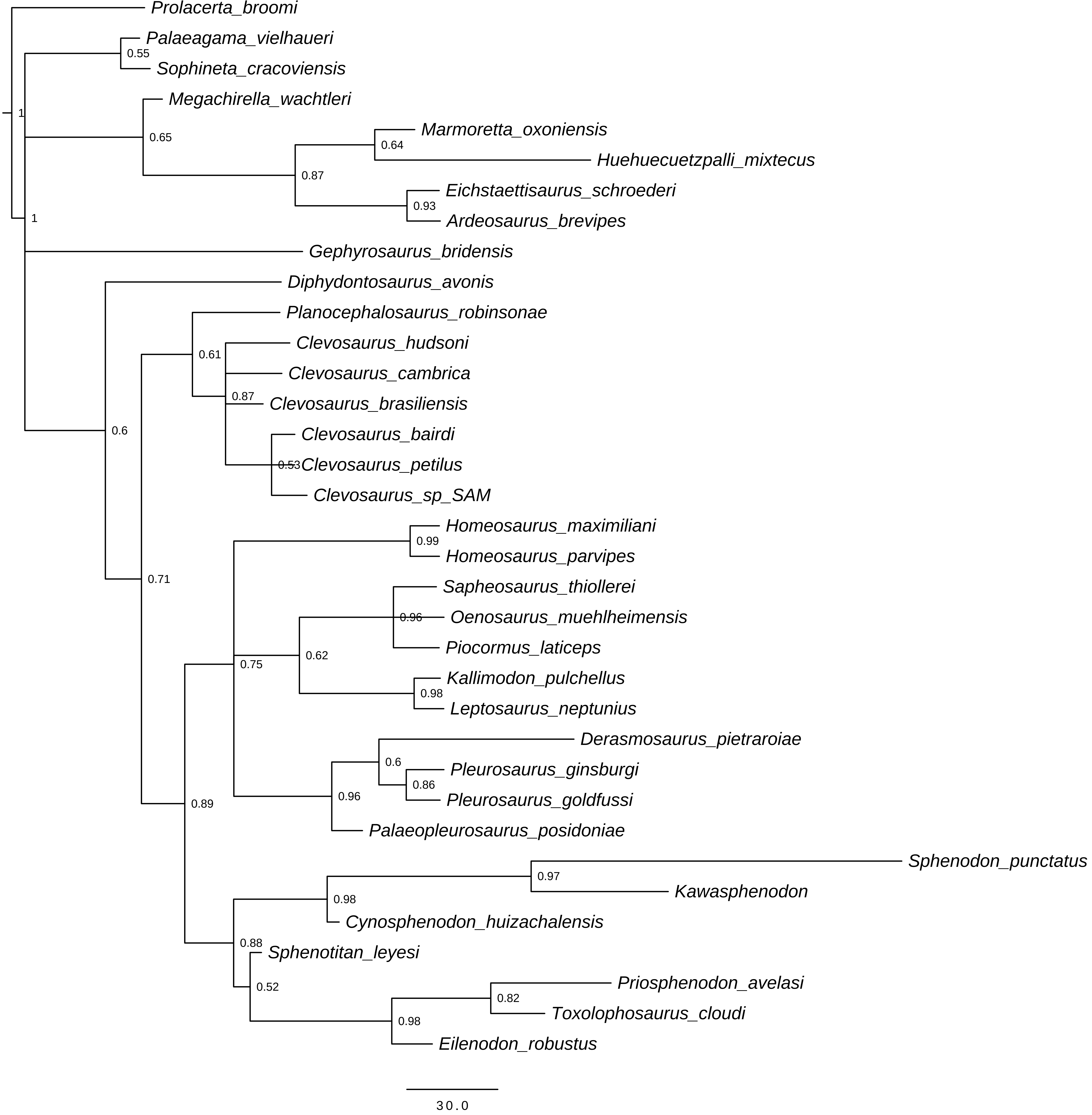
Prolacerta_broomi
Palaeagama_vielhaueri
0.55
Sophineta_cracoviensis
1
Megachirella_wachtleri
0.65
Marmoretta_oxoniensis
0.64
Huehuecuetzpalli_mixtecus
0.87
Eichstaettisaurus_schroederi
0.93
Ardeosaurus_brevipes
1
Gephyrosaurus_bridensis
Diphydontosaurus_avonis
Planocephalosaurus_robinsonae
0.61
Clevosaurus_hudsoni
Clevosaurus_cambrica
0.87
Clevosaurus_brasiliensis
0.6
Clevosaurus_bairdi
0.53
Clevosaurus_petilus
Clevosaurus_sp_SAM
Homeosaurus_maximiliani
0.99
Homeosaurus_parvipes
0.71
Sapheosaurus_thiollerei
0.96
Oenosaurus_muehlheimensis
Piocormus_laticeps
0.62
0.75
Kallimodon_pulchellus
0.98
Leptosaurus_neptunius
Derasmosaurus_pietraroiae
0.6
Pleurosaurus_ginsburgi
0.86
0.96
Pleurosaurus_goldfussi
0.89
Palaeopleurosaurus_posidoniae
Sphenodon_punctatus
0.97
Kawasphenodon
0.98
Cynosphenodon_huizachalensis
0.88
Sphenotitan_leyesi
0.52
Priosphenodon_avelasi
0.82
Toxolophosaurus_cloudi
0.98
Eilenodon_robustus
30.0
