# Supplementary material for: Sphenodontian phylogeny and the impact of model choice in Bayesian morphological clock estimates of divergence times and evolutionary rates
Source: BMC Biol. 2020 Dec 7;18:191. doi: 10.1186/s12915-020-00901-5 (PMC7720557; doi:10.1186/s12915-020-00901-5)
Supplement: Supplementary file 6 — Additional file 6. Input files including the dataset and all necessary coding (see Mr. Bayes blocks) to reproduce the analyses. [file 12915_2020_901_MOESM6_ESM.zip › InputFiles&OutputTrees/BayesCalibrated/FossilTips/BayesCal_TK02_ln_p3_60G_FT/BayesCal_TK02_FT_ln_p3_60g_AllCom.t.con.tre.pdf]

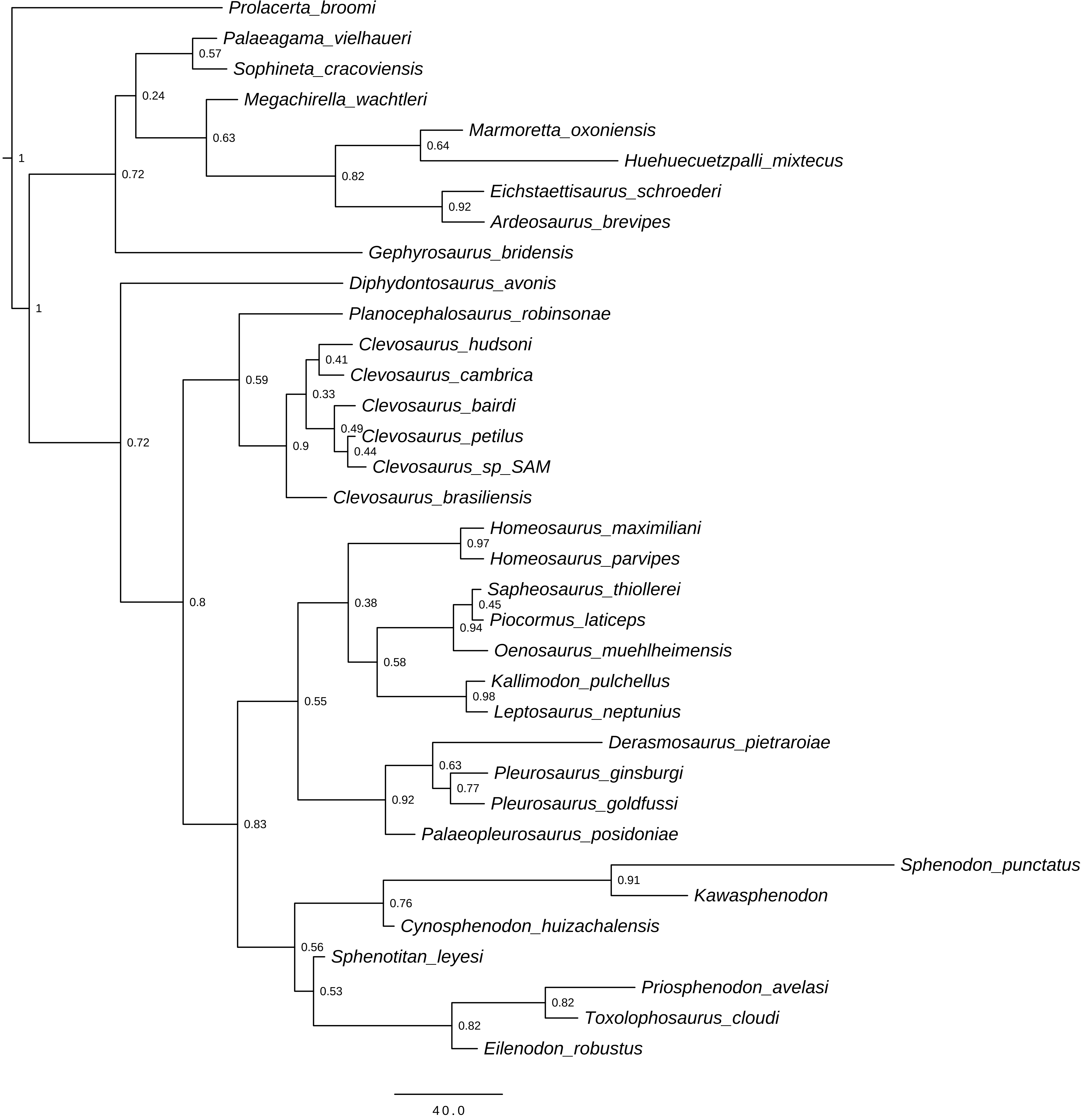

Prolacerta_broomi
Palaeagama_vielhaueri
0.57
Sophineta_cracoviensis
0.24
Megachirella_wachtleri
Marmoretta_oxoniensis
1
0.63
0.64
Huehuecuetzpalli_mixtecus
0.72
0.82
Eichstaettisaurus_schroederi
0.92
Ardeosaurus_brevipes
Gephyrosaurus_bridensis
Diphydontosaurus_avonis
1
Planocephalosaurus_robinsonae
Clevosaurus_hudsoni
0.41
Clevosaurus_cambrica
0.59
0.33
Clevosaurus_bairdi
0.49
Clevosaurus_petilus
0.72
0.44
0.9
Clevosaurus_sp_SAM
Clevosaurus_brasiliensis
Homeosaurus_maximiliani
0.97
Homeosaurus_parvipes
Sapheosaurus_thiollerei
0.38
0.45
0.8
Piocormus_laticeps
0.94
Oenosaurus_muehlheimensis
0.58
Kallimodon_pulchellus
0.55
0.98
Leptosaurus_neptunius
Derasmosaurus_pietraroiae
0.63
Pleurosaurus_ginsburgi
0.77
0.92
Pleurosaurus_goldfussi
0.83
Palaeopleurosaurus_posidoniae
Sphenodon_punctatus
0.91
Kawasphenodon
0.76
Cynosphenodon_huizachalensis
0.56
Sphenotitan_leyesi
Priosphenodon_avelasi
0.53
0.82
Toxolophosaurus_cloudi
0.82
Eilenodon_robustus
40.0
