# Supplementary material for: Sphenodontian phylogeny and the impact of model choice in Bayesian morphological clock estimates of divergence times and evolutionary rates
Source: BMC Biol. 2020 Dec 7;18:191. doi: 10.1186/s12915-020-00901-5 (PMC7720557; doi:10.1186/s12915-020-00901-5)
Supplement: Supplementary file 6 — Additional file 6. Input files including the dataset and all necessary coding (see Mr. Bayes blocks) to reproduce the analyses. [file 12915_2020_901_MOESM6_ESM.zip › InputFiles&OutputTrees/BayesCalibrated/FossilTips/BayesCal_TK02_ln_p3_60G_FT/BayesCal_TK02_FT_ln_p3_60g_AllCom.t.con.tre_Age.pdf]

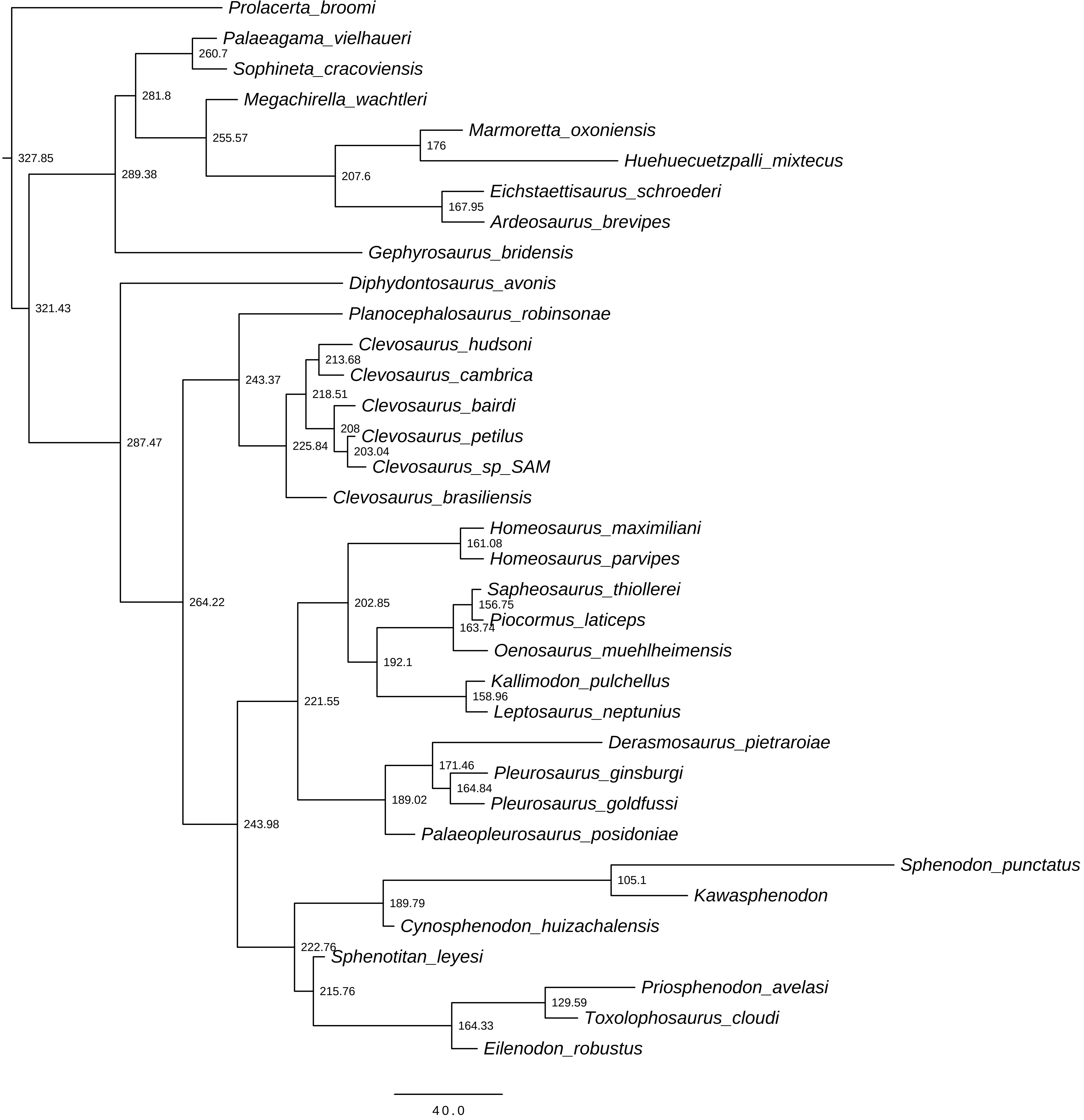
Prolacerta_broomi
Palaeagama_vielhaueri
260.7
Sophineta_cracoviensis
281.8
Megachirella_wachtleri
Marmoretta_oxoniensis
176
Huehuecuetzpalli_mixtecus
327.85
289.38
255.57
207.6
Eichstaettisaurus_schroederi
167.95
Ardeosaurus_brevipes
Gephyrosaurus_bridensis
Diphydontosaurus_avonis
321.43
Planocephalosaurus_robinsonae
Clevosaurus_hudsoni
213.68
Clevosaurus_cambrica
243.37
218.51
Clevosaurus_bairdi
208
Clevosaurus_petilus
287.47
225.84
203.04
Clevosaurus_sp_SAM
Clevosaurus_brasiliensis
Homeosaurus_maximiliani
161.08
Homeosaurus_parvipes
Sapheosaurus_thiollerei
264.22
202.85
156.75
Piocormus_laticeps
163.74
Oenosaurus_muehlheimensis
192.1
Kallimodon_pulchellus
221.55
158.96
Leptosaurus_neptunius
Derasmosaurus_pietraroiae
171.46
Pleurosaurus_ginsburgi
164.84
189.02
Pleurosaurus_goldfussi
243.98
Palaeopleurosaurus_posidoniae
Sphenodon_punctatus
105.1
Kawasphenodon
189.79
Cynosphenodon_huizachalensis
222.76
Sphenotitan_leyesi
Priosphenodon_avelasi
215.76
129.59
Toxolophosaurus_cloudi
164.33
Eilenodon_robustus
40.0
