# Supplementary material for: Sphenodontian phylogeny and the impact of model choice in Bayesian morphological clock estimates of divergence times and evolutionary rates
Source: BMC Biol. 2020 Dec 7;18:191. doi: 10.1186/s12915-020-00901-5 (PMC7720557; doi:10.1186/s12915-020-00901-5)
Supplement: Supplementary file 6 — Additional file 6. Input files including the dataset and all necessary coding (see Mr. Bayes blocks) to reproduce the analyses. [file 12915_2020_901_MOESM6_ESM.zip › InputFiles&OutputTrees/BayesCalibrated/FossilTips/BayesCal_TK02_ln_p3_60G_FT_NoR/BayesCal_TK02_FT_ln_p3_60g_NoR_AllCom.t.con.tre.pdf]

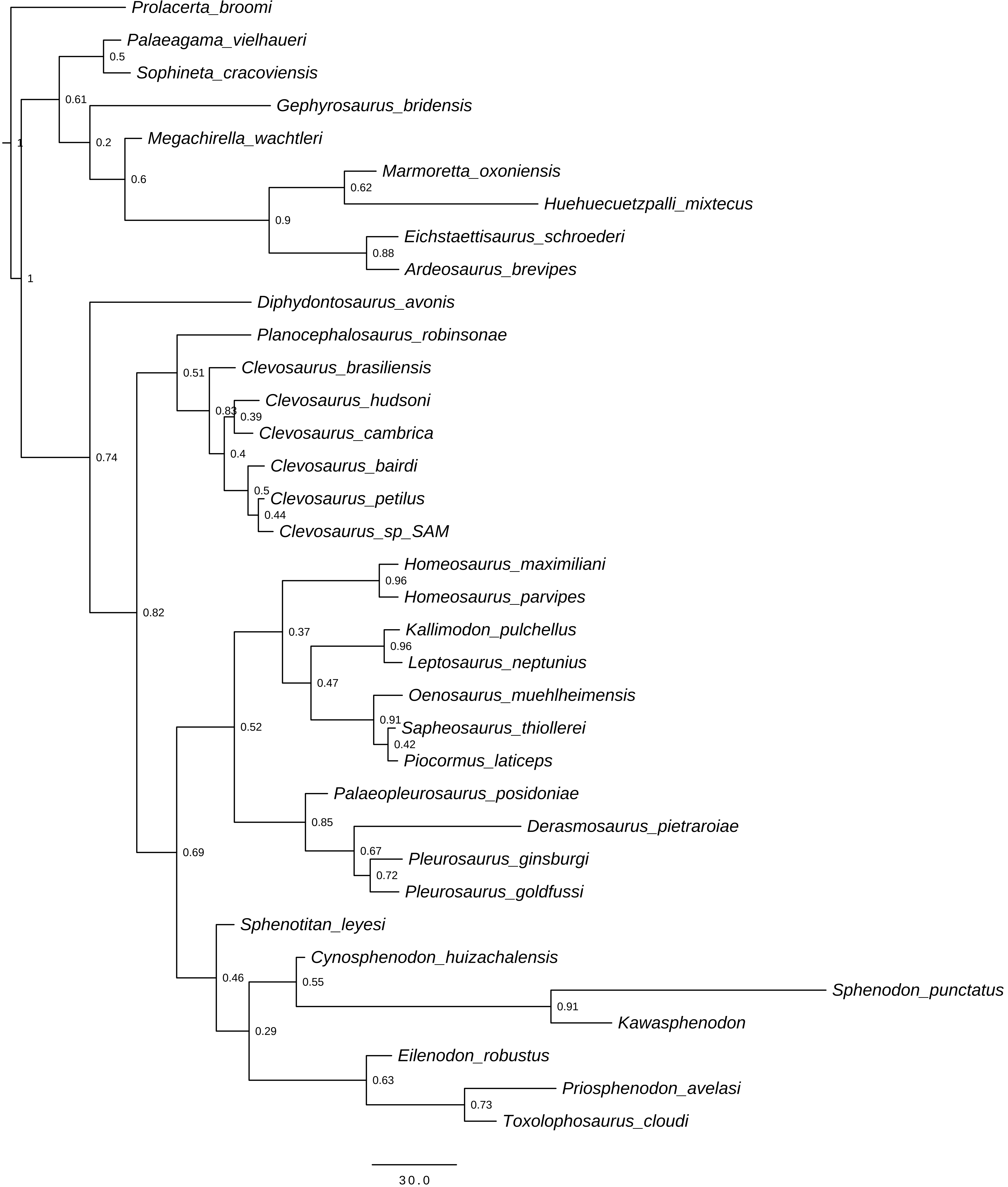
Prolacerta_broomi
Palaeagama_vielhaueri
0.5
Sophineta_cracoviensis
0.61
Gephyrosaurus_bridensis
1
0.2
Megachirella_wachtleri
0.6
Marmoretta_oxoniensis
0.62
Huehuecuetzpalli_mixtecus
0.9
Eichstaettisaurus_schroederi
0.88
Ardeosaurus_brevipes
1
Diphydontosaurus_avonis
Planocephalosaurus_robinsonae
0.51
Clevosaurus_brasiliensis
Clevosaurus_hudsoni
0.83
0.39
Clevosaurus_cambrica
0.74
0.4
Clevosaurus_bairdi
0.5
Clevosaurus_petilus
0.44
Clevosaurus_sp_SAM
Homeosaurus_maximiliani
0.96
Homeosaurus_parvipes
0.82
0.37
Kallimodon_pulchellus
0.96
Leptosaurus_neptunius
0.47
Oenosaurus_muehlheimensis
0.91
Sapheosaurus_thiollerei
0.52
0.42
Piocormus_laticeps
Palaeopleurosaurus_posidoniae
0.85
Derasmosaurus_pietraroiae
0.67
0.69
Pleurosaurus_ginsburgi
0.72
Pleurosaurus_goldfussi
Sphenotitan_leyesi
Cynosphenodon_huizachalensis
0.46
0.55
Sphenodon_punctatus
0.91
Kawasphenodon
0.29
Eilenodon_robustus
0.63
Priosphenodon_avelasi
0.73
Toxolophosaurus_cloudi
30.0
