# Supplementary material for: Sphenodontian phylogeny and the impact of model choice in Bayesian morphological clock estimates of divergence times and evolutionary rates
Source: BMC Biol. 2020 Dec 7;18:191. doi: 10.1186/s12915-020-00901-5 (PMC7720557; doi:10.1186/s12915-020-00901-5)
Supplement: Supplementary file 6 — Additional file 6. Input files including the dataset and all necessary coding (see Mr. Bayes blocks) to reproduce the analyses. [file 12915_2020_901_MOESM6_ESM.zip › InputFiles&OutputTrees/BayesCalibrated/FossilTips/BayesCal_TK02_ln_p3_60G_FT_NoR/BayesCal_TK02_FT_ln_p3_60g_NoR_AllCom.t.con.tre_Age95HPD.pdf]

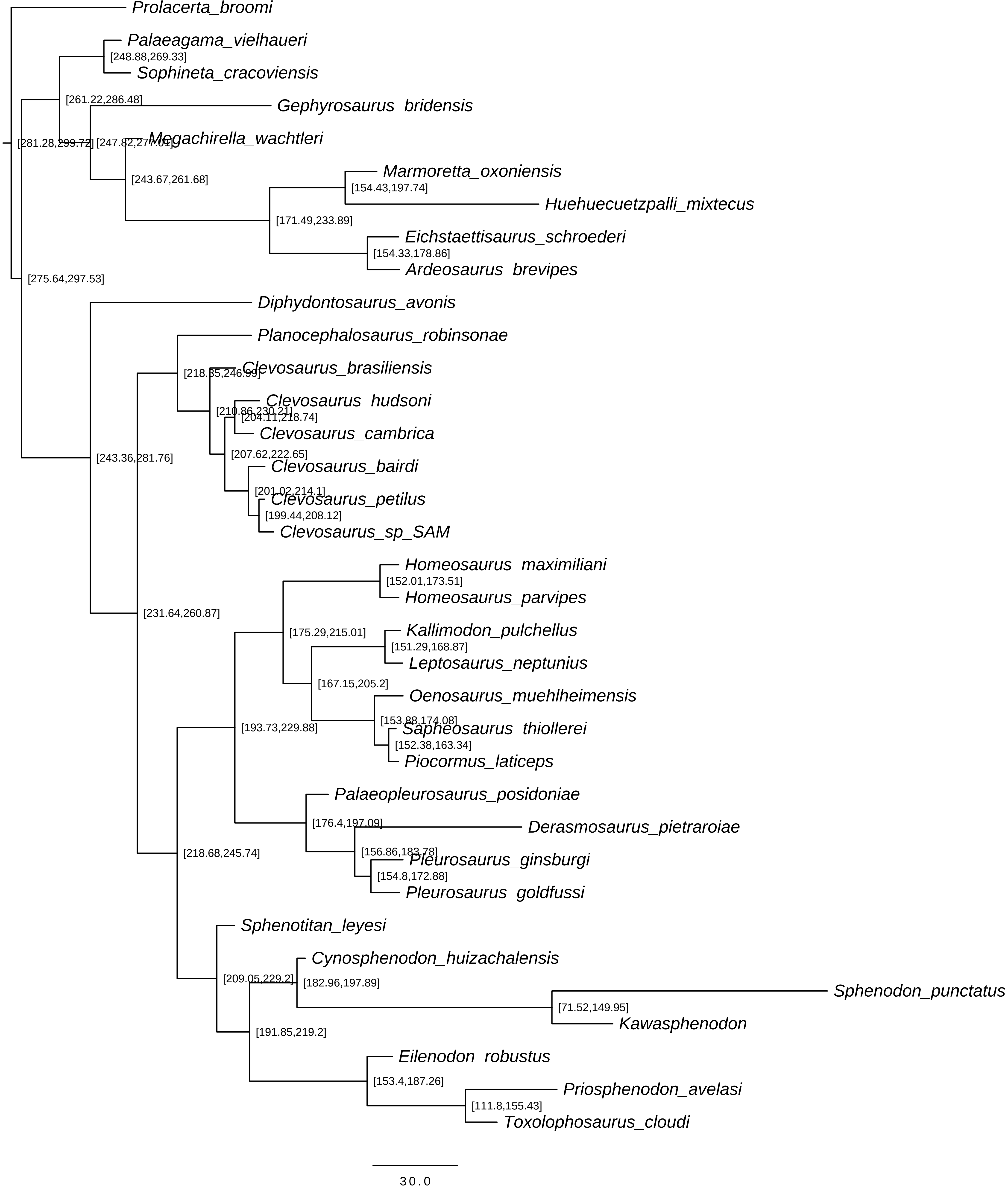
Prolacerta_broomi
Palaeagama_vielhaueri
[248.88,269.33]
Sophineta_cracoviensis
[261.22,286.48]
Gephyrosaurus_bridensis
[281.28,299.72]
[247.82,277.01]
Megachirella_wachtleri
Marmoretta_oxoniensis
[243.67,261.68]
[154.43,197.74]
Huehuecuetzpalli_mixtecus
[171.49,233.89]
Eichstaettisaurus_schroederi
[154.33,178.86]
Ardeosaurus_brevipes
[275.64,297.53]
Diphydontosaurus_avonis
Planocephalosaurus_robinsonae
[218.35,246.99]
Clevosaurus_brasiliensis
Clevosaurus_hudsoni
[210.86,230.21]
[204.11,218.74]
Clevosaurus_cambrica
[243.36,281.76]
[207.62,222.65]
Clevosaurus_bairdi
[201.02,214.1]
Clevosaurus_petilus
[199.44,208.12]
Clevosaurus_sp_SAM
Homeosaurus_maximiliani
[152.01,173.51]
Homeosaurus_parvipes
[231.64,260.87]
Kallimodon_pulchellus
[175.29,215.01]
[151.29,168.87]
Leptosaurus_neptunius
[167.15,205.2]
Oenosaurus_muehlheimensis
[153.88,174.08]
[193.73,229.88]
Sapheosaurus_thiollerei
[152.38,163.34]
Piocormus_laticeps
Palaeopleurosaurus_posidoniae
[176.4,197.09]
Derasmosaurus_pietraroiae
[218.68,245.74]
[156.86,183.78]
Pleurosaurus_ginsburgi
[154.8,172.88]
Pleurosaurus_goldfussi
Sphenotitan_leyesi
Cynosphenodon_huizachalensis
[209.05,229.2]
[182.96,197.89]
Sphenodon_punctatus
[71.52,149.95]
Kawasphenodon
[191.85,219.2]
Eilenodon_robustus
[153.4,187.26]
Priosphenodon_avelasi
[111.8,155.43]
Toxolophosaurus_cloudi
30.0
