# Supplementary material for: Sphenodontian phylogeny and the impact of model choice in Bayesian morphological clock estimates of divergence times and evolutionary rates
Source: BMC Biol. 2020 Dec 7;18:191. doi: 10.1186/s12915-020-00901-5 (PMC7720557; doi:10.1186/s12915-020-00901-5)
Supplement: Supplementary file 6 — Additional file 6. Input files including the dataset and all necessary coding (see Mr. Bayes blocks) to reproduce the analyses. [file 12915_2020_901_MOESM6_ESM.zip › InputFiles&OutputTrees/BayesCalibrated/FossilTips/BayesCal_TK02_ln_p3_60G_FT_NoR/BayesCal_TK02_FT_ln_p3_60g_NoR_AllCom.t.con.tre_AgeBars.pdf]

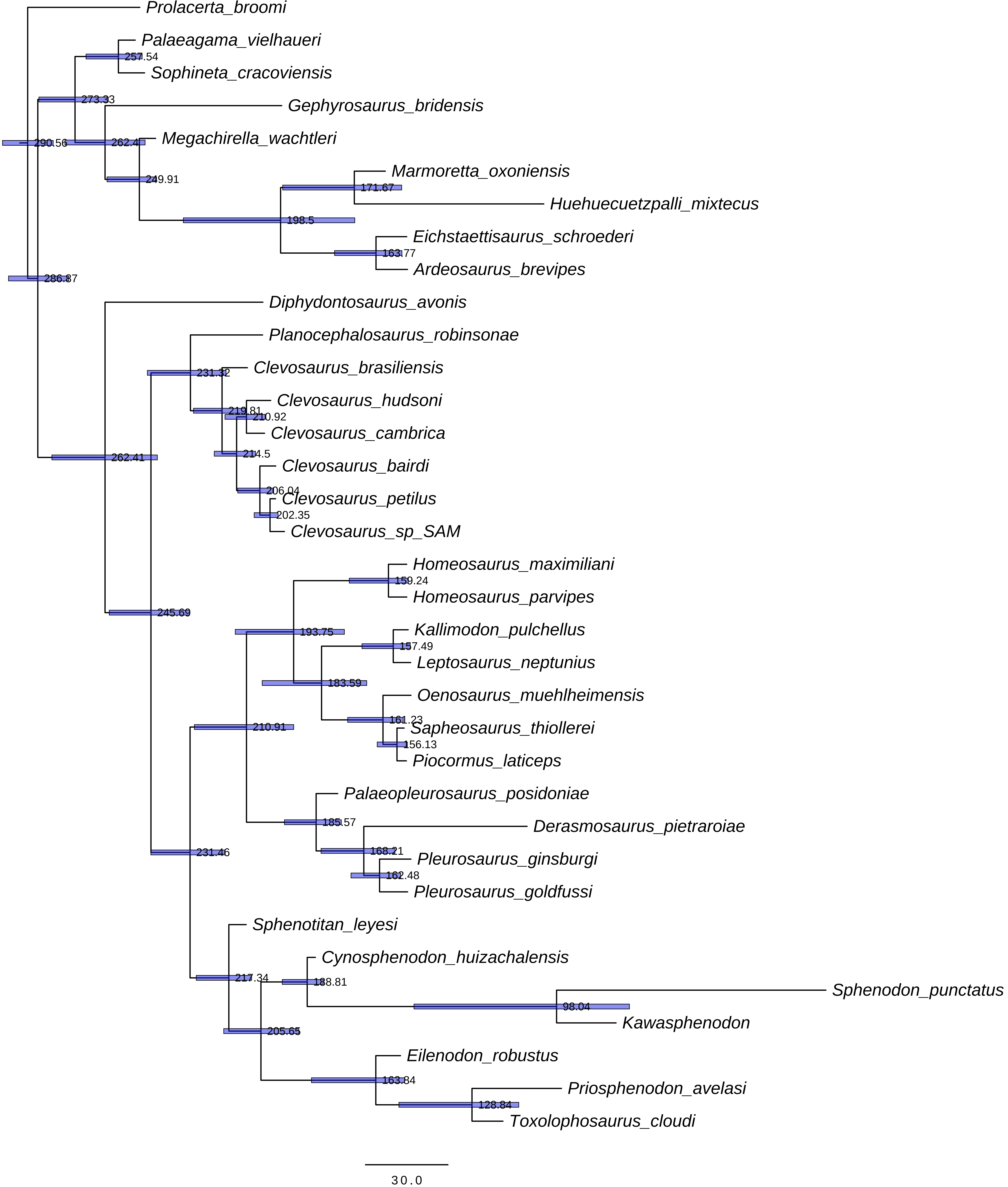

Prolacerta_broomi
Palaeagama_vielhaueri
257.54
Sophineta_cracoviensis
273.33
Gephyrosaurus_bridensis
290.56
262.4
Megachirella_wachtleri
249.91
Marmoretta_oxoniensis
171.67
Huehuecuetzpalli_mixtecus
198.5
Eichstaettisaurus_schroederi
163.77
Ardeosaurus_brevipes
286.87
Diphydontosaurus_avonis
Planocephalosaurus_robinsonae
231.32
Clevosaurus_brasiliensis
Clevosaurus_hudsoni
219.81
210.92
Clevosaurus_cambrica
262.41
214.5
Clevosaurus_bairdi
206.04
Clevosaurus_petilus
202.35
Clevosaurus_sp_SAM
Homeosaurus_maximiliani
159.24
Homeosaurus_parvipes
245.69
193.75
Kallimodon_pulchellus
157.49
Leptosaurus_neptunius
183.59
Oenosaurus_muehlheimensis
161.23
Sapheosaurus_thiollerei
156.13
Piocormus_laticeps
210.91
Palaeopleurosaurus_posidoniae
185.57
Derasmosaurus_pietraroiae
231.46
168.21
Pleurosaurus_ginsburgi
162.48
Pleurosaurus_goldfussi
Sphenotitan_leyesi
Cynosphenodon_huizachalensis
217.34
188.81
Sphenodon_punctatus
98.04
Kawasphenodon
205.65
Eilenodon_robustus
163.84
Priosphenodon_avelasi
128.84
Toxolophosaurus_cloudi
30.0
