# Supplementary material for: Sphenodontian phylogeny and the impact of model choice in Bayesian morphological clock estimates of divergence times and evolutionary rates
Source: BMC Biol. 2020 Dec 7;18:191. doi: 10.1186/s12915-020-00901-5 (PMC7720557; doi:10.1186/s12915-020-00901-5)
Supplement: Supplementary file 6 — Additional file 6. Input files including the dataset and all necessary coding (see Mr. Bayes blocks) to reproduce the analyses. [file 12915_2020_901_MOESM6_ESM.zip › InputFiles&OutputTrees/BayesCalibrated/FossilTips/BayesCal_TK02_ln_p3_60G_FT_NoR_LExct/BayesCal_TK02_FT_ln_p3_60g_NoR_LExct_AllCom.t.con.tre_Age.pdf]

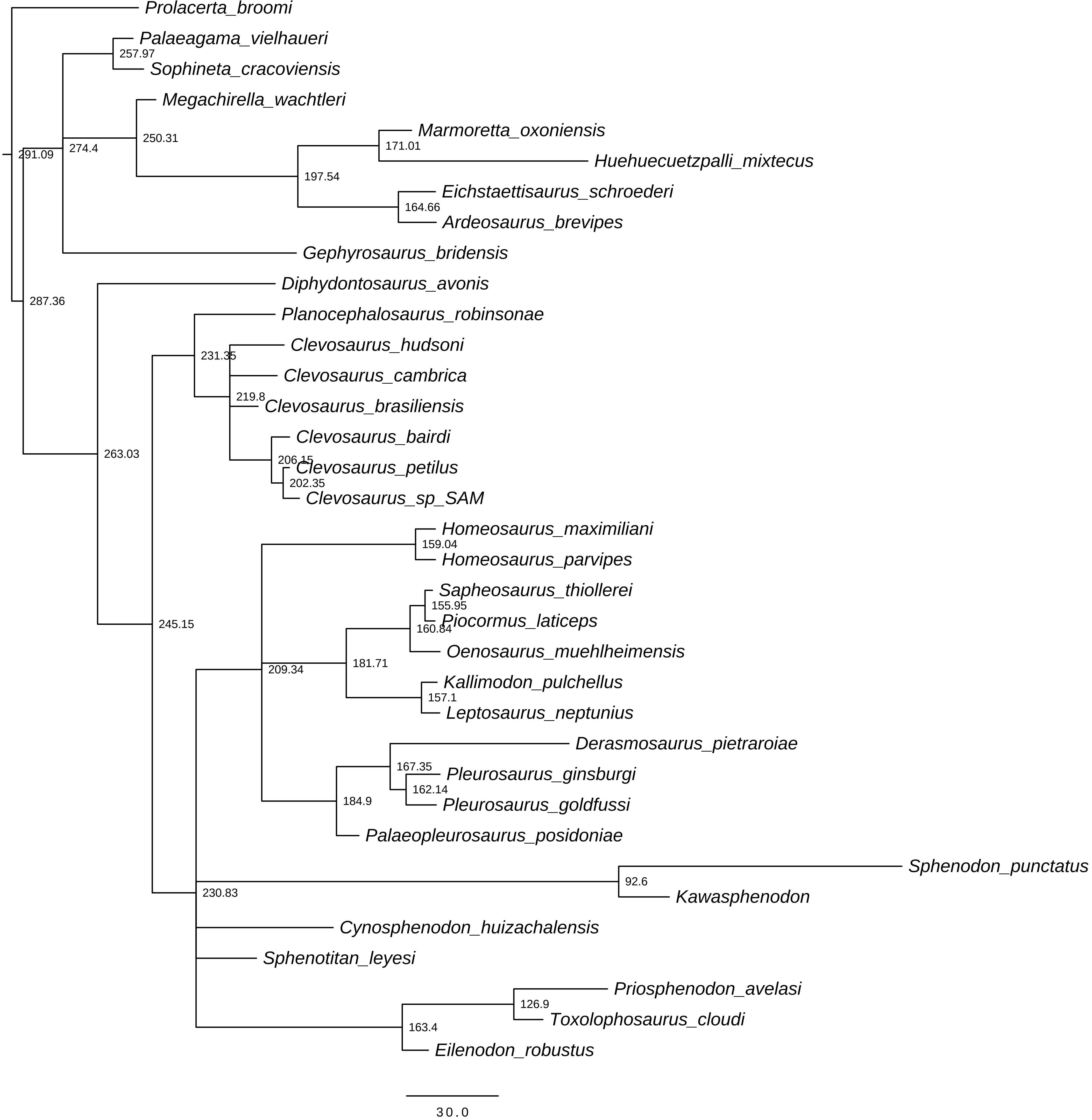

Prolacerta_broomi
Palaeagama_vielhaueri
257.97
Sophineta_cracoviensis
Megachirella_wachtleri
Marmoretta_oxoniensis
171.01
Huehuecuetzpalli_mixtecus
291.09
274.4
250.31
197.54
Eichstaettisaurus_schroederi
164.66
Ardeosaurus_brevipes
Gephyrosaurus_bridensis
Diphydontosaurus_avonis
287.36
Planocephalosaurus_robinsonae
Clevosaurus_hudsoni
231.35
Clevosaurus_cambrica
219.8
Clevosaurus_brasiliensis
Clevosaurus_bairdi
263.03
206.15
Clevosaurus_petilus
202.35
Clevosaurus_sp_SAM
Homeosaurus_maximiliani
159.04
Homeosaurus_parvipes
Sapheosaurus_thiollerei
155.95
Piocormus_laticeps
160.84
245.15
Oenosaurus_muehlheimensis
181.71
209.34
Kallimodon_pulchellus
157.1
Leptosaurus_neptunius
Derasmosaurus_pietraroiae
167.35
Pleurosaurus_ginsburgi
162.14
184.9
Pleurosaurus_goldfussi
Palaeopleurosaurus_posidoniae
Sphenodon_punctatus
92.6
230.83
Kawasphenodon
Cynosphenodon_huizachalensis
Sphenotitan_leyesi
Priosphenodon_avelasi
126.9
Toxolophosaurus_cloudi
163.4
Eilenodon_robustus
30.0
