# Supplementary material for: Sphenodontian phylogeny and the impact of model choice in Bayesian morphological clock estimates of divergence times and evolutionary rates
Source: BMC Biol. 2020 Dec 7;18:191. doi: 10.1186/s12915-020-00901-5 (PMC7720557; doi:10.1186/s12915-020-00901-5)
Supplement: Supplementary file 6 — Additional file 6. Input files including the dataset and all necessary coding (see Mr. Bayes blocks) to reproduce the analyses. [file 12915_2020_901_MOESM6_ESM.zip › InputFiles&OutputTrees/BayesCalibrated/FossilTips/BayesCal_TK02_ln_p3_60G_FT_NoR_LExct/BayesCal_TK02_FT_ln_p3_60g_NoR_LExct_MRC.t.con.tre.pdf]

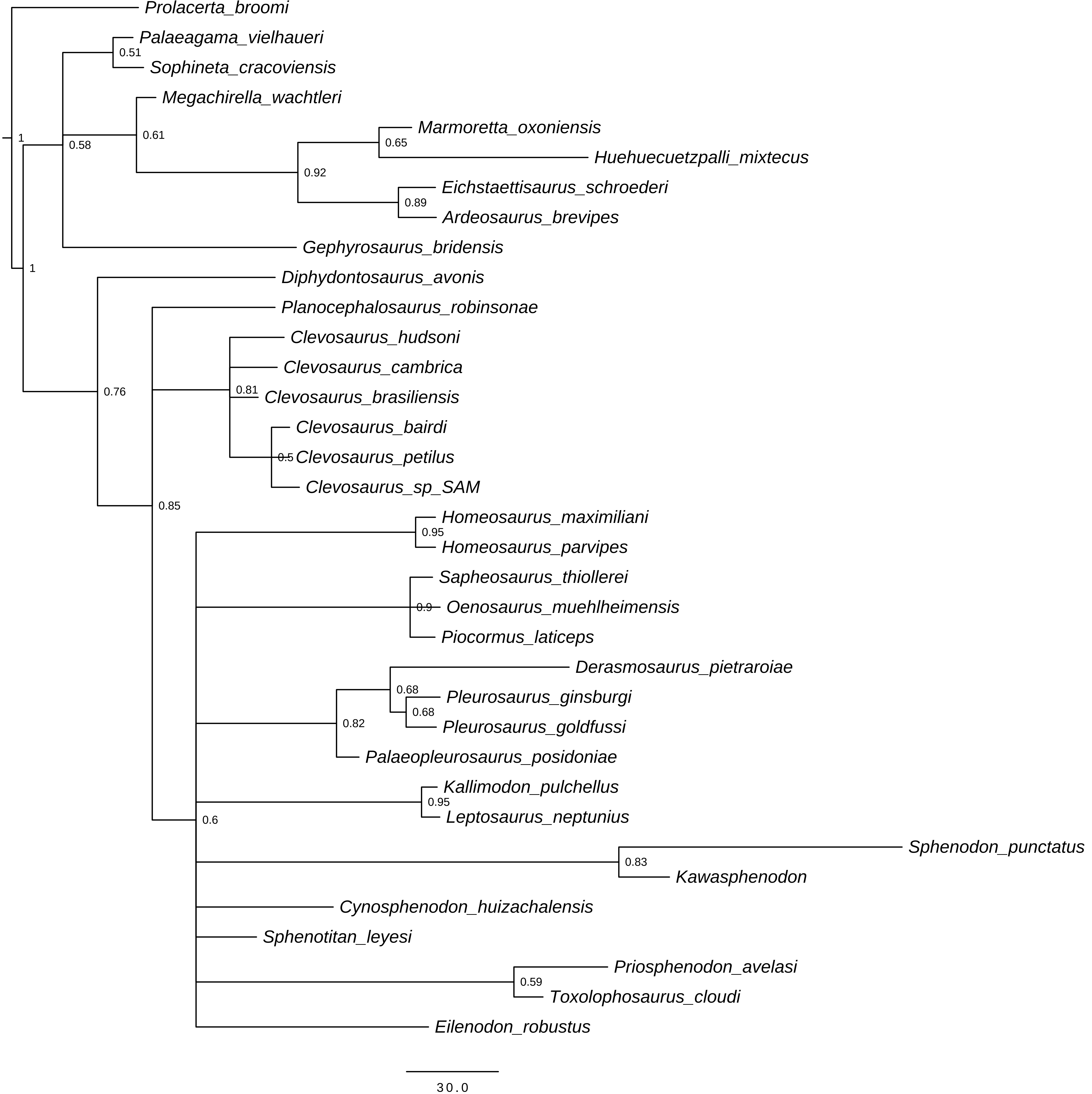

Prolacerta_broomi
Palaeagama_vielhaueri
0.51
Sophineta_cracoviensis
Megachirella_wachtleri
Marmoretta_oxoniensis
0.65
Huehuecuetzpalli_mixtecus
Eichstaettisaurus_schroederi
0.89
Ardeosaurus_brevipes
0.92
0.61
0.58
1
Gephyrosaurus_bridensis
1
Diphydontosaurus_avonis
Planocephalosaurus_robinsonae
Clevosaurus_hudsoni
Clevosaurus_cambrica
0.81
Clevosaurus_brasiliensis
Clevosaurus_bairdi
0.5
Clevosaurus_petilus
Clevosaurus_sp_SAM
0.76
0.85
Homeosaurus_maximiliani
0.95
Homeosaurus_parvipes
Sapheosaurus_thiollerei
0.9
Oenosaurus_muehlheimensis
Piocormus_laticeps
Derasmosaurus_pietraroiae
0.68
Pleurosaurus_ginsburgi
0.68
Pleurosaurus_goldfussi
0.82
Palaeopleurosaurus_posidoniae
Kallimodon_pulchellus
0.95
Leptosaurus_neptunius
0.6
Sphenodon_punctatus
0.83
Kawasphenodon
Cynosphenodon_huizachalensis
Sphenotitan_leyesi
Priosphenodon_avelasi
0.59
Toxolophosaurus_cloudi
Eilenodon_robustus
30.0
