# Supplementary material for: Sphenodontian phylogeny and the impact of model choice in Bayesian morphological clock estimates of divergence times and evolutionary rates
Source: BMC Biol. 2020 Dec 7;18:191. doi: 10.1186/s12915-020-00901-5 (PMC7720557; doi:10.1186/s12915-020-00901-5)
Supplement: Supplementary file 6 — Additional file 6. Input files including the dataset and all necessary coding (see Mr. Bayes blocks) to reproduce the analyses. [file 12915_2020_901_MOESM6_ESM.zip › InputFiles&OutputTrees/BayesCalibrated/FossilTips/BayesCal_TK02_ln_p3_60G_FT_SFBD(s)3l/BayesCal_TK02_ln_p3_60G_FT_SFBD2_AllCom.t.con.tre.pdf]

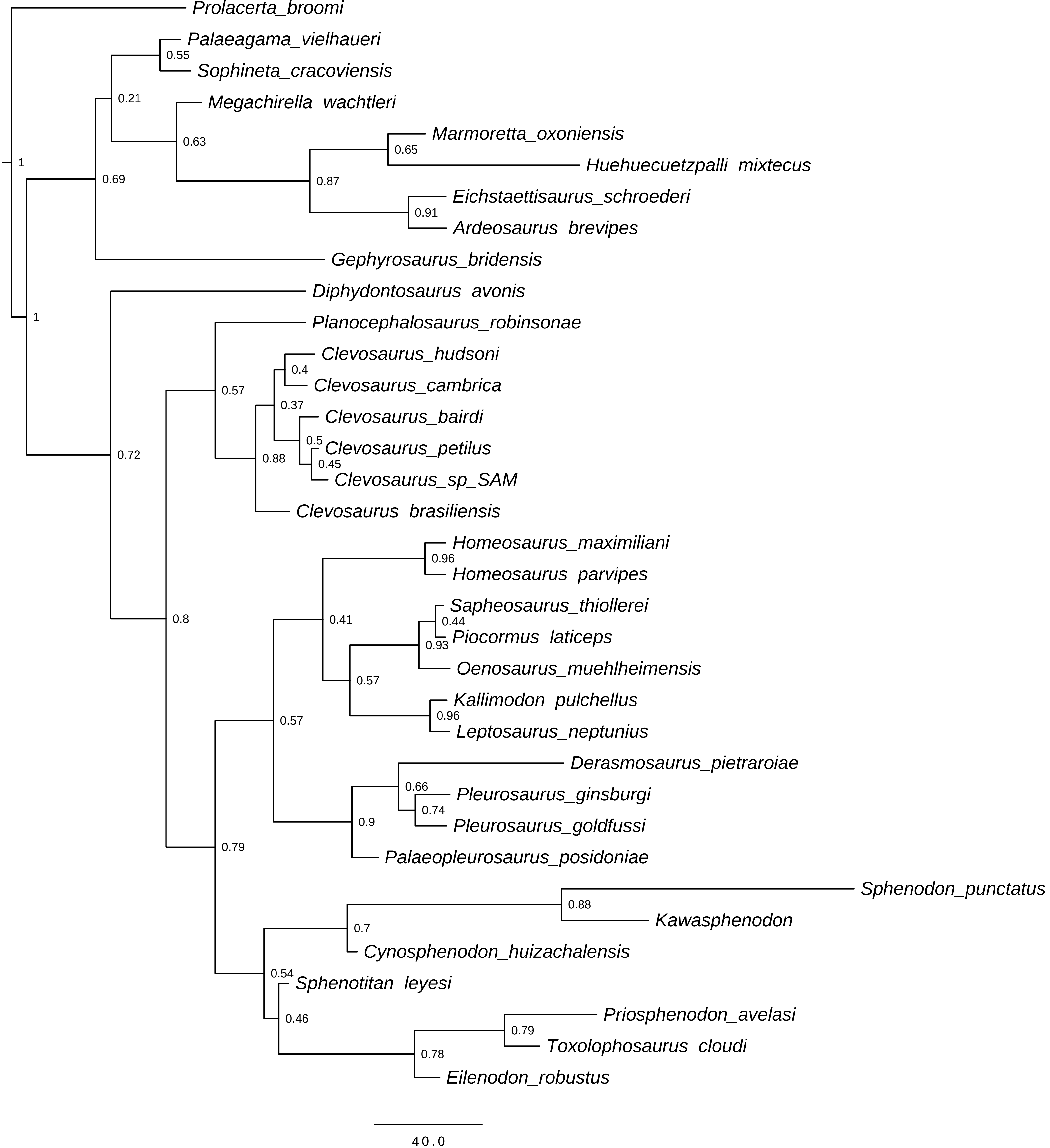
Prolacerta_broomi
Palaeagama_vielhaueri
0.55
Sophineta_cracoviensis
0.21
Megachirella_wachtleri
0.63
Marmoretta_oxoniensis
0.65
Huehuecuetzpalli_mixtecus
1
0.69
0.87
Eichstaettisaurus_schroederi
0.91
Ardeosaurus_brevipes
Gephyrosaurus_bridensis
Diphydontosaurus_avonis
1
Planocephalosaurus_robinsonae
Clevosaurus_hudsoni
0.4
Clevosaurus_cambrica
0.57
0.37
Clevosaurus_bairdi
0.5
Clevosaurus_petilus
0.72
0.88
0.45
Clevosaurus_sp_SAM
Clevosaurus_brasiliensis
Homeosaurus_maximiliani
0.96
Homeosaurus_parvipes
Sapheosaurus_thiollerei
0.41
0.44
0.8
Piocormus_laticeps
0.93
Oenosaurus_muehlheimensis
0.57
Kallimodon_pulchellus
0.96
0.57
Leptosaurus_neptunius
Derasmosaurus_pietraroiae
0.66
Pleurosaurus_ginsburgi
0.74
Pleurosaurus_goldfussi
0.9
0.79
Palaeopleurosaurus_posidoniae
Sphenodon_punctatus
0.88
Kawasphenodon
0.7
Cynosphenodon_huizachalensis
0.54
Sphenotitan_leyesi
0.46
Priosphenodon_avelasi
0.79
Toxolophosaurus_cloudi
0.78
Eilenodon_robustus
40.0
