# Supplementary material for: Sphenodontian phylogeny and the impact of model choice in Bayesian morphological clock estimates of divergence times and evolutionary rates
Source: BMC Biol. 2020 Dec 7;18:191. doi: 10.1186/s12915-020-00901-5 (PMC7720557; doi:10.1186/s12915-020-00901-5)
Supplement: Supplementary file 6 — Additional file 6. Input files including the dataset and all necessary coding (see Mr. Bayes blocks) to reproduce the analyses. [file 12915_2020_901_MOESM6_ESM.zip › InputFiles&OutputTrees/BayesCalibrated/FossilTips/BayesCal_TK02_ln_p3_60G_FT_SFBD(s)3l/BayesCal_TK02_ln_p3_60G_FT_SFBD2_AllCom.t.con.tre_Ages.pdf]

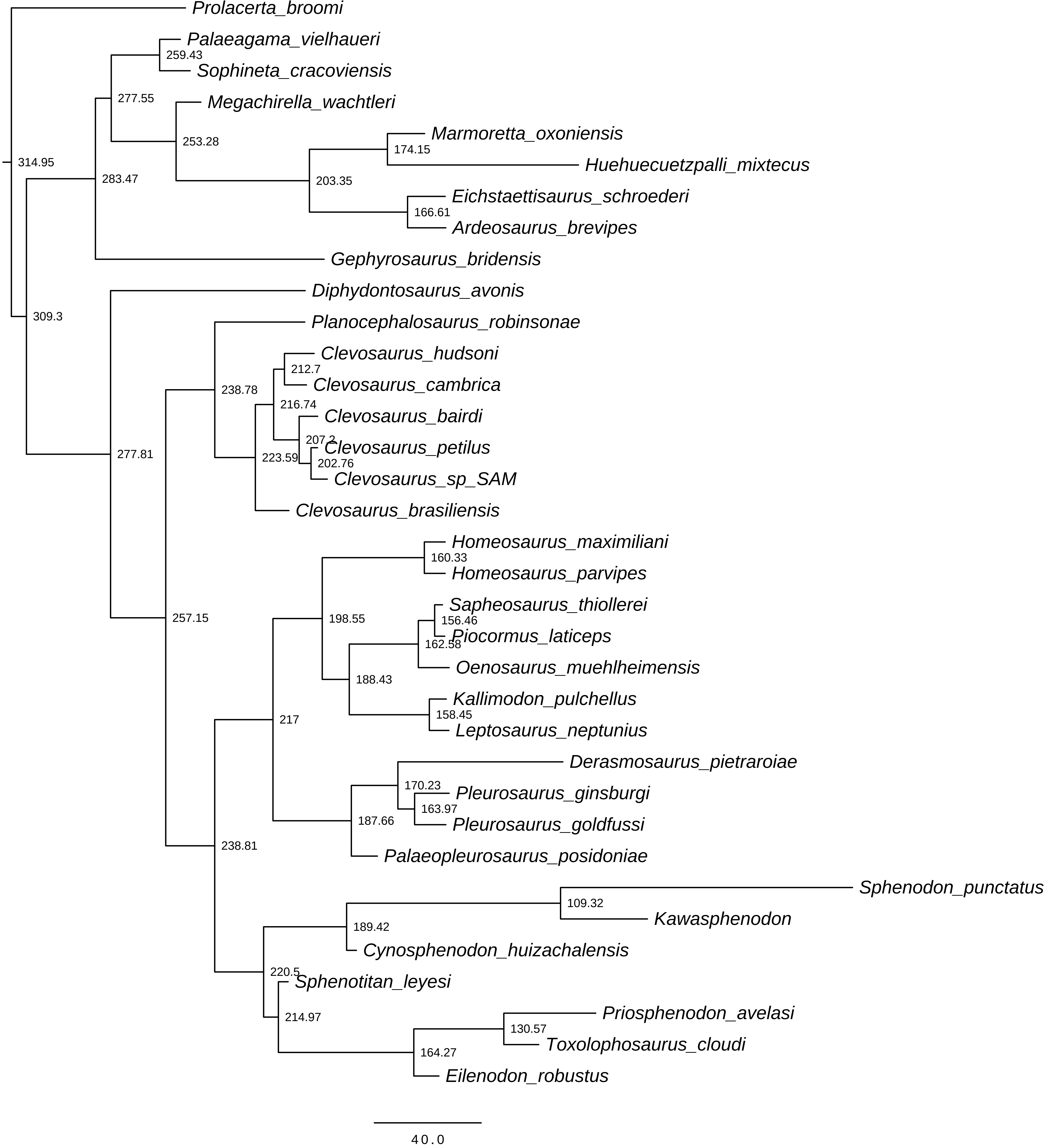

Prolacerta_broomi
Palaeagama_vielhaueri
259.43
Sophineta_cracoviensis
277.55
Megachirella_wachtleri
Marmoretta_oxoniensis
174.15
Huehuecuetzpalli_mixtecus
253.28
203.35
Eichstaettisaurus_schroederi
166.61
Ardeosaurus_brevipes
314.95
283.47
Gephyrosaurus_bridensis
Diphydontosaurus_avonis
309.3
Planocephalosaurus_robinsonae
Clevosaurus_hudsoni
212.7
Clevosaurus_cambrica
238.78
216.74
Clevosaurus_bairdi
207.2
Clevosaurus_petilus
223.59
202.76
Clevosaurus_sp_SAM
277.81
Clevosaurus_brasiliensis
Homeosaurus_maximiliani
160.33
Homeosaurus_parvipes
Sapheosaurus_thiollerei
156.46
198.55
Piocormus_laticeps
162.58
Oenosaurus_muehlheimensis
257.15
188.43
Kallimodon_pulchellus
158.45
Leptosaurus_neptunius
217
Derasmosaurus_pietraroiae
170.23
Pleurosaurus_ginsburgi
163.97
Pleurosaurus_goldfussi
187.66
238.81
Palaeopleurosaurus_posidoniae
Sphenodon_punctatus
109.32
Kawasphenodon
189.42
Cynosphenodon_huizachalensis
220.5
Sphenotitan_leyesi
Priosphenodon_avelasi
214.97
130.57
Toxolophosaurus_cloudi
164.27
Eilenodon_robustus
40.0
