# Supplementary material for: Sphenodontian phylogeny and the impact of model choice in Bayesian morphological clock estimates of divergence times and evolutionary rates
Source: BMC Biol. 2020 Dec 7;18:191. doi: 10.1186/s12915-020-00901-5 (PMC7720557; doi:10.1186/s12915-020-00901-5)
Supplement: Supplementary file 6 — Additional file 6. Input files including the dataset and all necessary coding (see Mr. Bayes blocks) to reproduce the analyses. [file 12915_2020_901_MOESM6_ESM.zip › InputFiles&OutputTrees/BayesCalibrated/FossilTips/BayesCal_TK02_ln_p3_60G_FT_SFBD(s)3l_NoR/BayesCal_TK02_ln_p3_60G_FT_SFBD2_NoR_AllCom.t.con.tre.pdf]

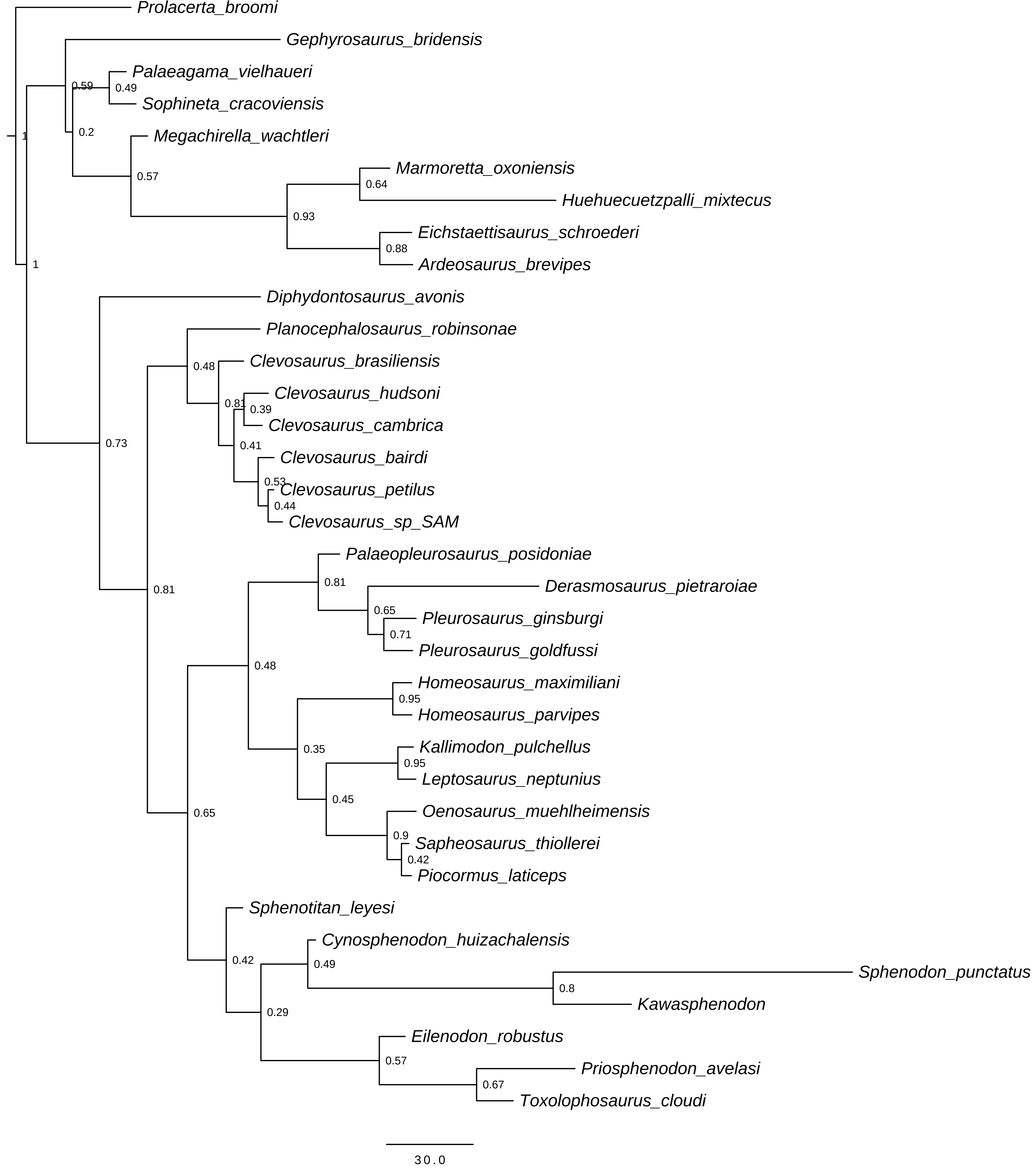
Prolacerta_broomi
Gephyrosaurus_bridensis
Palaeagama_vielhaueri
Sophineta_cracoviensis
Megachirella_wachtleri
Marmoretta_oxoniensis
Huehuecuetzpalli_mixtecus
Eichstaettisaurus_schroederi
Ardeosaurus_brevipes
Diphydontosaurus_avonis
Planocephalosaurus_robinsonae
Clevosaurus_brasiliensis
Clevosaurus_hudsoni
Clevosaurus_cambrica
Clevosaurus_bairdi
Clevosaurus_petilus
Clevosaurus_sp_SAM
Palaeopleurosaurus_posidoniae
Derasmosaurus_pietraroiae
Pleurosaurus_ginsburgi
Pleurosaurus_goldfussi
Homeosaurus_maximiliani
Homeosaurus_parvipes
Kallimodon_pulchellus
Leptosaurus_neptunius
Oenosaurus_muehlheimensis
Sapheosaurus_thiollerei
Piocormus_laticeps
Sphenotitan_leyesi
Cynosphenodon_huizachalensis
Sphenodon_punctatus
Kawasphenodon
Eilenodon_robustus
Priosphenodon_avelasi
Toxolophosaurus_cloudi
1
1
0.59
0.49
0.2
0.57
0.64
0.93
0.88
0.73
0.48
0.81
0.39
0.41
0.53
0.44
0.81
0.81
0.65
0.71
0.48
0.95
0.35
0.95
0.45
0.9
0.42
0.65
0.42
0.49
0.8
0.29
0.57
0.67
30.0
