# Supplementary material for: Sphenodontian phylogeny and the impact of model choice in Bayesian morphological clock estimates of divergence times and evolutionary rates
Source: BMC Biol. 2020 Dec 7;18:191. doi: 10.1186/s12915-020-00901-5 (PMC7720557; doi:10.1186/s12915-020-00901-5)
Supplement: Supplementary file 6 — Additional file 6. Input files including the dataset and all necessary coding (see Mr. Bayes blocks) to reproduce the analyses. [file 12915_2020_901_MOESM6_ESM.zip › InputFiles&OutputTrees/BayesCalibrated/FossilTips/BayesCal_TK02_ln_p3_60G_FT_SFBD(s)3l_NoR/BayesCal_TK02_ln_p3_60G_FT_SFBD2_NoR_AllCom.t.con.tre_Ages.pdf]

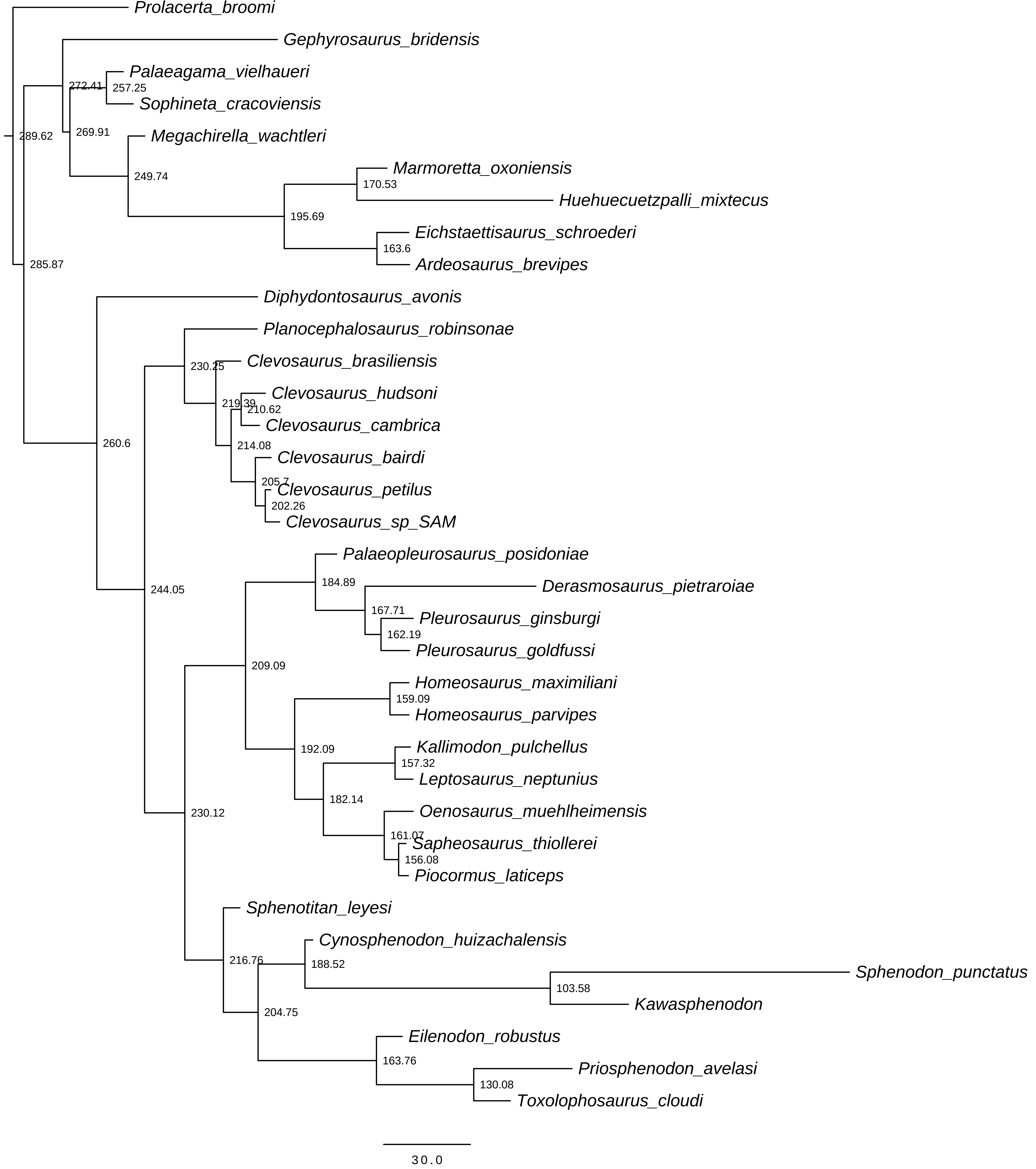

Prolacerta_broomi
Gephyrosaurus_bridensis
Palaeagama_vielhaueri
Sophineta_cracoviensis
Megachirella_wachtleri
Marmoretta_oxoniensis
Huehuecuetzpalli_mixtecus
Eichstaettisaurus_schroederi
Ardeosaurus_brevipes
Diphydontosaurus_avonis
Planocephalosaurus_robinsonae
Clevosaurus_brasiliensis
Clevosaurus_hudsoni
Clevosaurus_cambrica
Clevosaurus_bairdi
Clevosaurus_petilus
Clevosaurus_sp_SAM
Palaeopleurosaurus_posidoniae
Derasmosaurus_pietraroiae
Pleurosaurus_ginsburgi
Pleurosaurus_goldfussi
Homeosaurus_maximiliani
Homeosaurus_parvipes
Kallimodon_pulchellus
Leptosaurus_neptunius
Oenosaurus_muehlheimensis
Sapheosaurus_thiollerei
Piocormus_laticeps
Sphenotitan_leyesi
Cynosphenodon_huizachalensis
Sphenodon_punctatus
Kawasphenodon
Eilenodon_robustus
Priosphenodon_avelasi
Toxolophosaurus_cloudi
289.62
285.87
272.41
257.25
269.91
249.74
170.53
195.69
163.6
260.6
230.25
219.39
210.62
214.08
205.7
202.26
244.05
184.89
167.71
162.19
209.09
159.09
192.09
157.32
182.14
161.07
156.08
230.12
216.76
188.52
103.58
204.75
163.76
130.08
30.0
