# Supplementary material for: Sphenodontian phylogeny and the impact of model choice in Bayesian morphological clock estimates of divergence times and evolutionary rates
Source: BMC Biol. 2020 Dec 7;18:191. doi: 10.1186/s12915-020-00901-5 (PMC7720557; doi:10.1186/s12915-020-00901-5)
Supplement: Supplementary file 6 — Additional file 6. Input files including the dataset and all necessary coding (see Mr. Bayes blocks) to reproduce the analyses. [file 12915_2020_901_MOESM6_ESM.zip › InputFiles&OutputTrees/BayesCalibrated/FossilTips/BayesCal_TK02_ln_p3_60G_FT_SFBD(s)3l_NoR/BayesCal_TK02_ln_p3_60G_FT_SFBD2_NoR_MRC.t.con.tre.pdf]

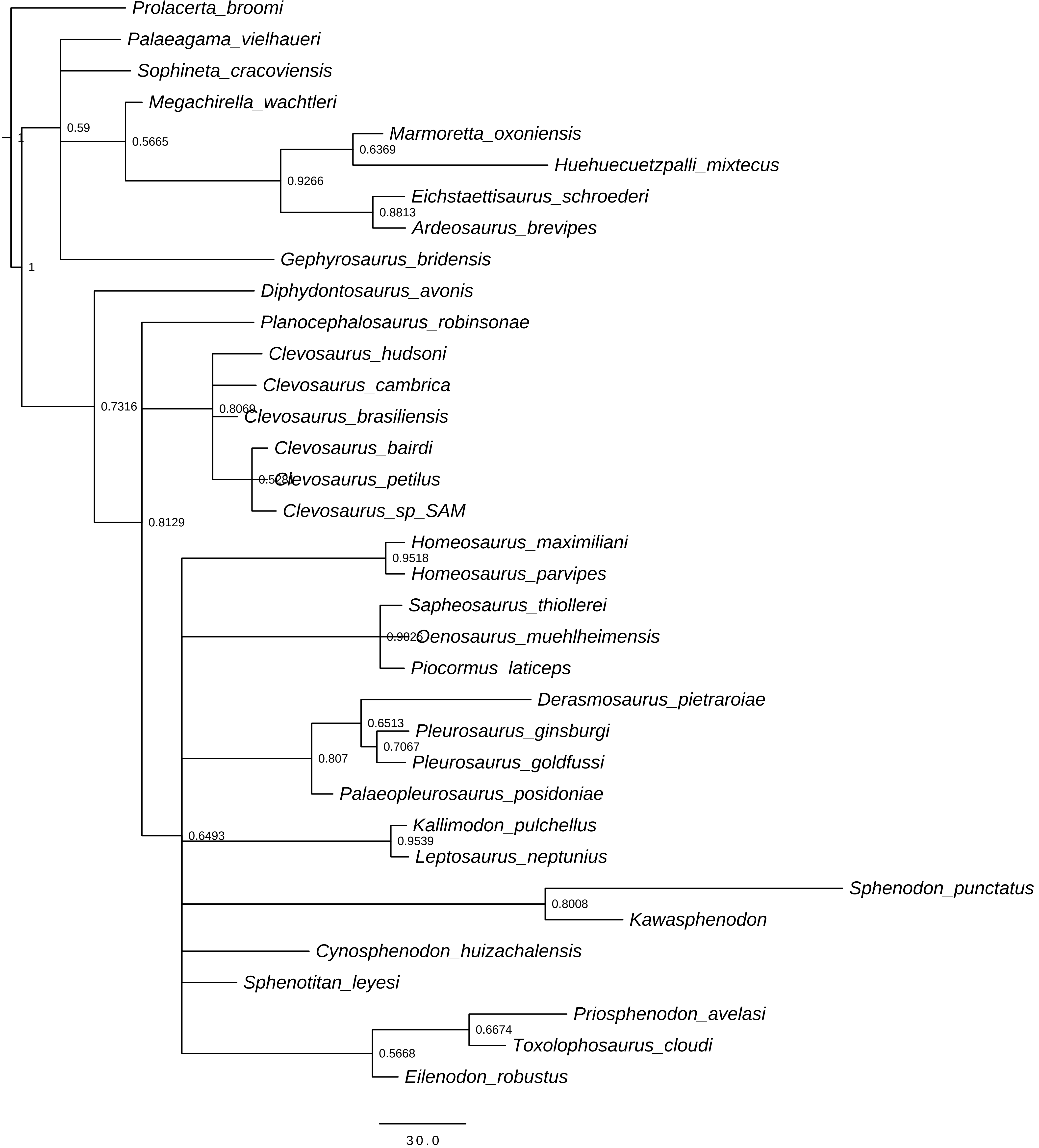
Prolacerta_broomi
Palaeagama_vielhaueri
Sophineta_cracoviensis
Megachirella_wachtleri
Marmoretta_oxoniensis
Huehuecuetzpalli_mixtecus
Eichstaettisaurus_schroederi
Ardeosaurus_brevipes
Gephyrosaurus_bridensis
Diphydontosaurus_avonis
Planocephalosaurus_robinsonae
Clevosaurus_hudsoni
Clevosaurus_cambrica
Clevosaurus_brasiliensis
Clevosaurus_bairdi
Clevosaurus_petilus
Clevosaurus_sp_SAM
Homeosaurus_maximiliani
Homeosaurus_parvipes
Sapheosaurus_thiollerei
Oenosaurus_muehlheimensis
Piocormus_laticeps
Derasmosaurus_pietraroiae
Pleurosaurus_ginsburgi
Pleurosaurus_goldfussi
Palaeopleurosaurus_posidoniae
Kallimodon_pulchellus
Leptosaurus_neptunius
Sphenodon_punctatus
Kawasphenodon
Cynosphenodon_huizachalensis
Sphenotitan_leyesi
Priosphenodon_avelasi
Toxolophosaurus_cloudi
Eilenodon_robustus
1
1
0.59
0.5665
0.6369
0.9266
0.8813
0.7316
0.8069
0.8129
0.9518
0.807
0.6513
0.7067
0.6493
0.9539
0.8008
0.6674
0.5668
30.0
