# Supplementary material for: Sphenodontian phylogeny and the impact of model choice in Bayesian morphological clock estimates of divergence times and evolutionary rates
Source: BMC Biol. 2020 Dec 7;18:191. doi: 10.1186/s12915-020-00901-5 (PMC7720557; doi:10.1186/s12915-020-00901-5)
Supplement: Supplementary file 6 — Additional file 6. Input files including the dataset and all necessary coding (see Mr. Bayes blocks) to reproduce the analyses. [file 12915_2020_901_MOESM6_ESM.zip › InputFiles&OutputTrees/BayesCalibrated/FossilTips/BayesCal_TK02_ln_p3_60G_FT_SFBD(s)3l_NoR_LExct/BayesCal_TK02_ln_p3_60G_FT_SFBD2_NoR_LExct_AllCom.t.con.tre.pdf]

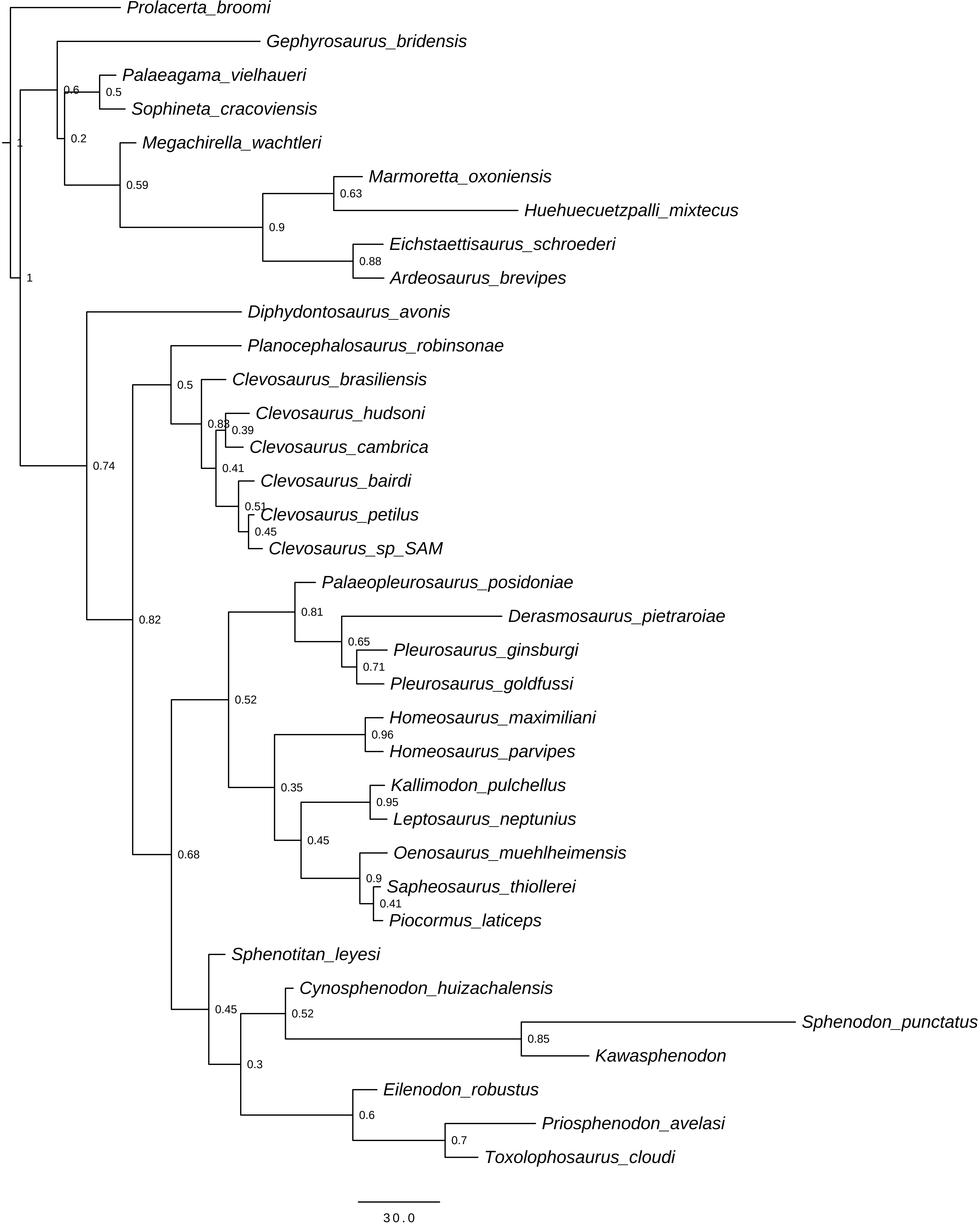
Prolacerta_broomi
Gephyrosaurus_bridensis
0.6
Palaeagama_vielhaueri
0.5
Sophineta_cracoviensis
1
0.2
Megachirella_wachtleri
0.59
Marmoretta_oxoniensis
0.63
Huehuecuetzpalli_mixtecus
0.9
Eichstaettisaurus_schroederi
0.88
Ardeosaurus_brevipes
1
Diphydontosaurus_avonis
Planocephalosaurus_robinsonae
0.5
Clevosaurus_brasiliensis
Clevosaurus_hudsoni
0.83
0.39
Clevosaurus_cambrica
0.74
0.41
Clevosaurus_bairdi
0.51
Clevosaurus_petilus
0.45
Clevosaurus_sp_SAM
Palaeopleurosaurus_posidoniae
0.81
Derasmosaurus_pietraroiae
0.82
0.65
Pleurosaurus_ginsburgi
0.71
Pleurosaurus_goldfussi
0.52
Homeosaurus_maximiliani
0.96
Homeosaurus_parvipes
Kallimodon_pulchellus
0.35
0.95
Leptosaurus_neptunius
0.45
Oenosaurus_muehlheimensis
0.68
0.9
Sapheosaurus_thiollerei
0.41
Piocormus_laticeps
Sphenotitan_leyesi
Cynosphenodon_huizachalensis
0.45
0.52
Sphenodon_punctatus
0.85
Kawasphenodon
0.3
Eilenodon_robustus
0.6
Priosphenodon_avelasi
0.7
Toxolophosaurus_cloudi
30.0
