# Supplementary material for: Sphenodontian phylogeny and the impact of model choice in Bayesian morphological clock estimates of divergence times and evolutionary rates
Source: BMC Biol. 2020 Dec 7;18:191. doi: 10.1186/s12915-020-00901-5 (PMC7720557; doi:10.1186/s12915-020-00901-5)
Supplement: Supplementary file 6 — Additional file 6. Input files including the dataset and all necessary coding (see Mr. Bayes blocks) to reproduce the analyses. [file 12915_2020_901_MOESM6_ESM.zip › InputFiles&OutputTrees/BayesCalibrated/FossilTips/BayesCal_TK02_ln_p3_60G_FT_SFBD(s)3l_NoR_LExct/BayesCal_TK02_ln_p3_60G_FT_SFBD2_NoR_LExct_MRC.t.con.tre.pdf]

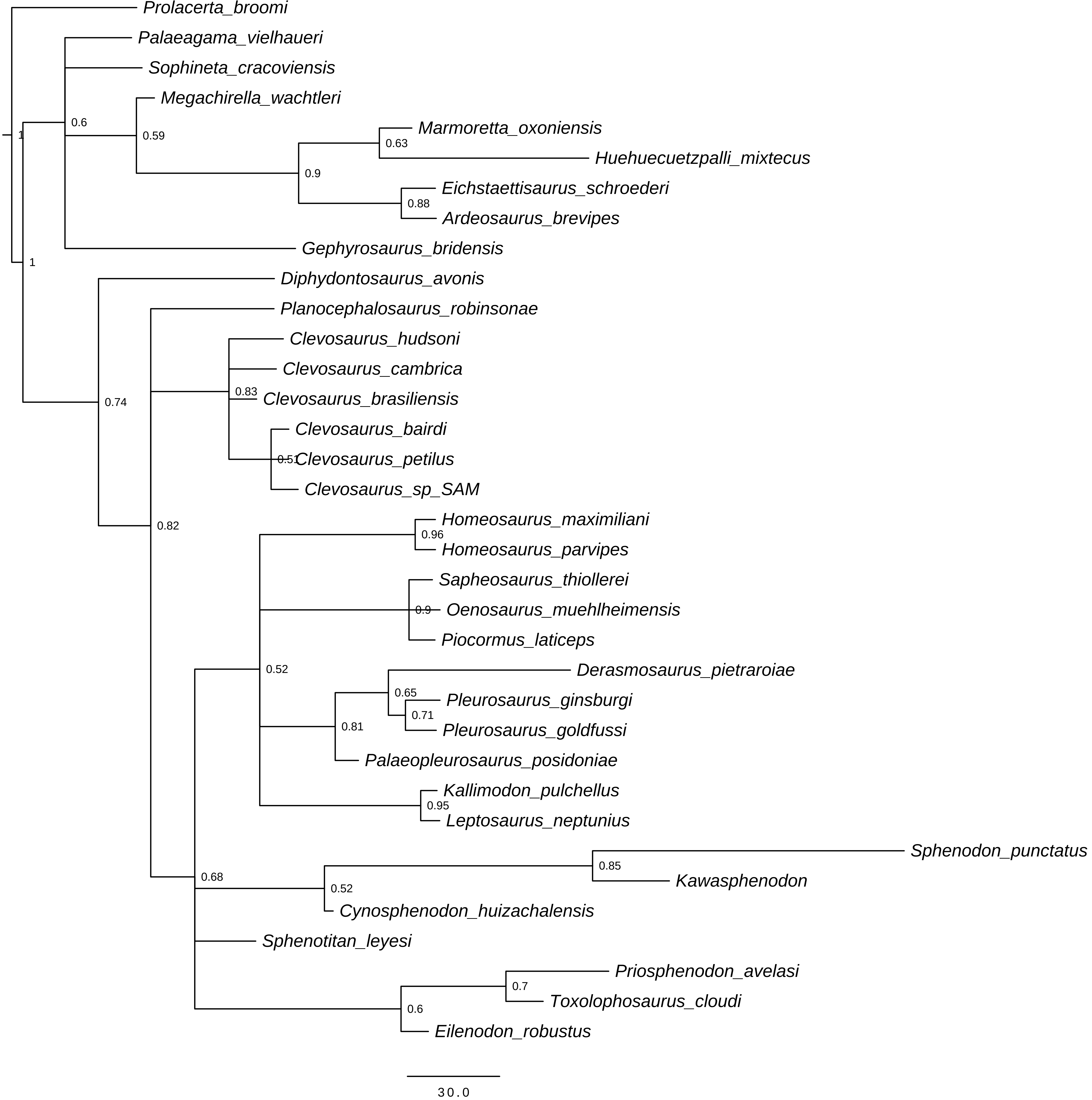

Prolacerta_broomi
Palaeagama_vielhaueri
Sophineta_cracoviensis
Megachirella_wachtleri
Marmoretta_oxoniensis
Huehuecuetzpalli_mixtecus
Eichstaettisaurus_schroederi
Ardeosaurus_brevipes
Gephyrosaurus_bridensis
Diphydontosaurus_avonis
Planocephalosaurus_robinsonae
Clevosaurus_hudsoni
Clevosaurus_cambrica
Clevosaurus_brasiliensis
Clevosaurus_bairdi
Clevosaurus_petilus
Clevosaurus_sp_SAM
Homeosaurus_maximiliani
Homeosaurus_parvipes
Sapheosaurus_thiollerei
Oenosaurus_muehlheimensis
Piocormus_laticeps
Derasmosaurus_pietraroiae
Pleurosaurus_ginsburgi
Pleurosaurus_goldfussi
Palaeopleurosaurus_posidoniae
Kallimodon_pulchellus
Leptosaurus_neptunius
Sphenodon_punctatus
Kawasphenodon
Cynosphenodon_huizachalensis
Sphenotitan_leyesi
Priosphenodon_avelasi
Toxolophosaurus_cloudi
Eilenodon_robustus
1
0.6
0.59
0.63
0.9
0.88
1
0.74
0.83
0.51
0.82
0.96
0.9
0.52
0.65
0.71
0.81
0.95
0.85
0.68
0.52
0.7
0.6
30.0
