# Supplementary material for: Sphenodontian phylogeny and the impact of model choice in Bayesian morphological clock estimates of divergence times and evolutionary rates
Source: BMC Biol. 2020 Dec 7;18:191. doi: 10.1186/s12915-020-00901-5 (PMC7720557; doi:10.1186/s12915-020-00901-5)
Supplement: Supplementary file 6 — Additional file 6. Input files including the dataset and all necessary coding (see Mr. Bayes blocks) to reproduce the analyses. [file 12915_2020_901_MOESM6_ESM.zip › InputFiles&OutputTrees/BayesCalibrated/FossilTips/BayesCal_TK02_ln_p3_StartTr_3per_60G_FT/BayesCal_TK02_ln_FT_Start_p3_3per_60g_MRC.t.con.tre.pdf]

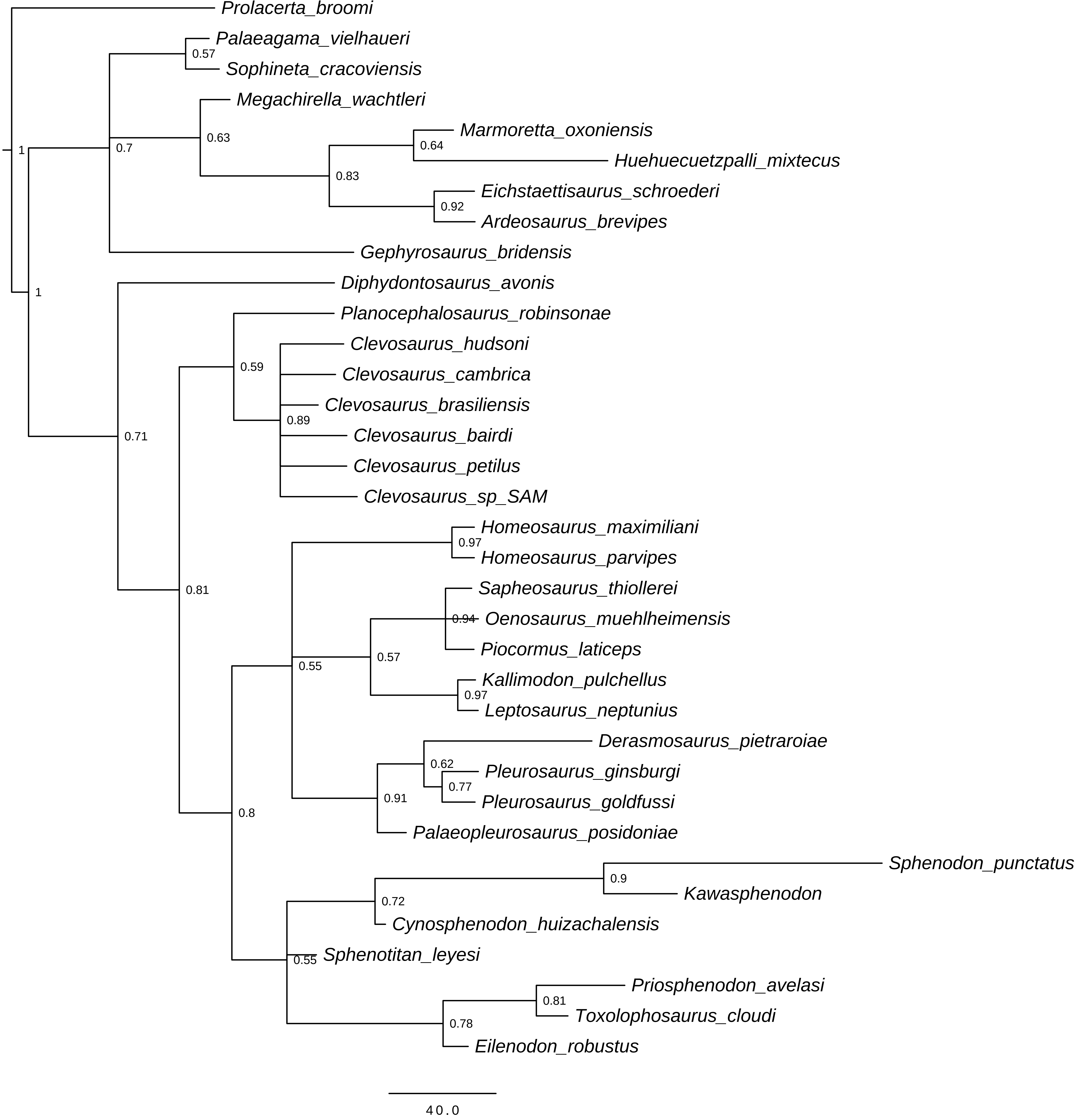

Prolacerta_broomi
Palaeagama_vielhaueri
0.57
Sophineta_cracoviensis
Megachirella_wachtleri
Marmoretta_oxoniensis
0.64
Huehuecuetzpalli_mixtecus
0.63
0.83
Eichstaettisaurus_schroederi
0.92
Ardeosaurus_brevipes
1
0.7
Gephyrosaurus_bridensis
Diphydontosaurus_avonis
1
Planocephalosaurus_robinsonae
Clevosaurus_hudsoni
0.59
Clevosaurus_cambrica
Clevosaurus_brasiliensis
0.89
0.71
Clevosaurus_bairdi
Clevosaurus_petilus
Clevosaurus_sp_SAM
Homeosaurus_maximiliani
0.97
Homeosaurus_parvipes
0.81
Sapheosaurus_thiollerei
0.94
Oenosaurus_muehlheimensis
Piocormus_laticeps
0.55
0.57
Kallimodon_pulchellus
0.97
Leptosaurus_neptunius
Derasmosaurus_pietraroiae
0.62
Pleurosaurus_ginsburgi
0.77
0.91
Pleurosaurus_goldfussi
0.8
Palaeopleurosaurus_posidoniae
Sphenodon_punctatus
0.9
Kawasphenodon
0.72
Cynosphenodon_huizachalensis
Sphenotitan_leyesi
0.55
Priosphenodon_avelasi
0.81
Toxolophosaurus_cloudi
0.78
Eilenodon_robustus
40.0
