# Supplementary material for: Sphenodontian phylogeny and the impact of model choice in Bayesian morphological clock estimates of divergence times and evolutionary rates
Source: BMC Biol. 2020 Dec 7;18:191. doi: 10.1186/s12915-020-00901-5 (PMC7720557; doi:10.1186/s12915-020-00901-5)
Supplement: Supplementary file 6 — Additional file 6. Input files including the dataset and all necessary coding (see Mr. Bayes blocks) to reproduce the analyses. [file 12915_2020_901_MOESM6_ESM.zip › InputFiles&OutputTrees/BayesCalibrated/Random/BayesCal_IGR_ln_p1_Ra/BayesCal_IGR_ln_p1_Ra_AllCom.t.con.tre.pdf]

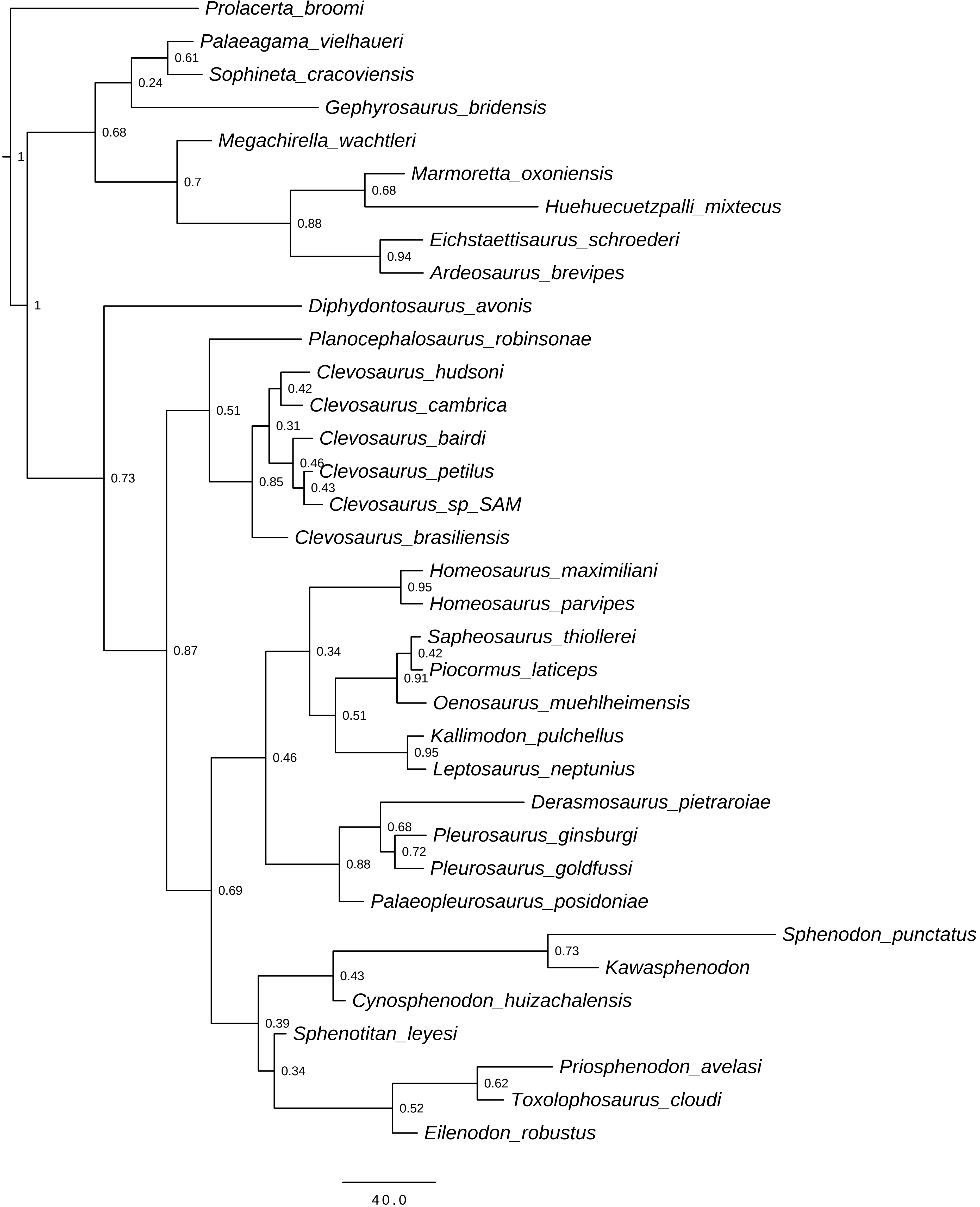

Prolacerta_broomi
Palaeagama_vielhaueri
0.61
Sophineta_cracoviensis
0.24
Gephyrosaurus_bridensis
0.68
Megachirella_wachtleri
1
Marmoretta_oxoniensis
0.7
0.68
Huehuecuetzpalli_mixtecus
0.88
Eichstaettisaurus_schroederi
0.94
Ardeosaurus_brevipes
Diphydontosaurus_avonis
1
Planocephalosaurus_robinsonae
Clevosaurus_hudsoni
0.42
Clevosaurus_cambrica
0.51
0.31
Clevosaurus_bairdi
0.46
Clevosaurus_petilus
0.73
0.85
0.43
Clevosaurus_sp_SAM
Clevosaurus_brasiliensis
Homeosaurus_maximiliani
0.95
Homeosaurus_parvipes
Sapheosaurus_thiollerei
0.42
0.87
0.34
Piocormus_laticeps
0.91
Oenosaurus_muehlheimensis
0.51
Kallimodon_pulchellus
0.95
0.46
Leptosaurus_neptunius
Derasmosaurus_pietraroiae
0.68
Pleurosaurus_ginsburgi
0.72
Pleurosaurus_goldfussi
0.88
0.69
Palaeopleurosaurus_posidoniae
Sphenodon_punctatus
0.73
Kawasphenodon
0.43
Cynosphenodon_huizachalensis
0.39
Sphenotitan_leyesi
Priosphenodon_avelasi
0.34
0.62
Toxolophosaurus_cloudi
0.52
Eilenodon_robustus
40.0
