# Supplementary material for: Sphenodontian phylogeny and the impact of model choice in Bayesian morphological clock estimates of divergence times and evolutionary rates
Source: BMC Biol. 2020 Dec 7;18:191. doi: 10.1186/s12915-020-00901-5 (PMC7720557; doi:10.1186/s12915-020-00901-5)
Supplement: Supplementary file 6 — Additional file 6. Input files including the dataset and all necessary coding (see Mr. Bayes blocks) to reproduce the analyses. [file 12915_2020_901_MOESM6_ESM.zip › InputFiles&OutputTrees/BayesCalibrated/Random/BayesCal_IGR_ln_p1_Ra/BayesCal_IGR_ln_p1_Ra_AllCom.t.con.tre_Age.pdf]

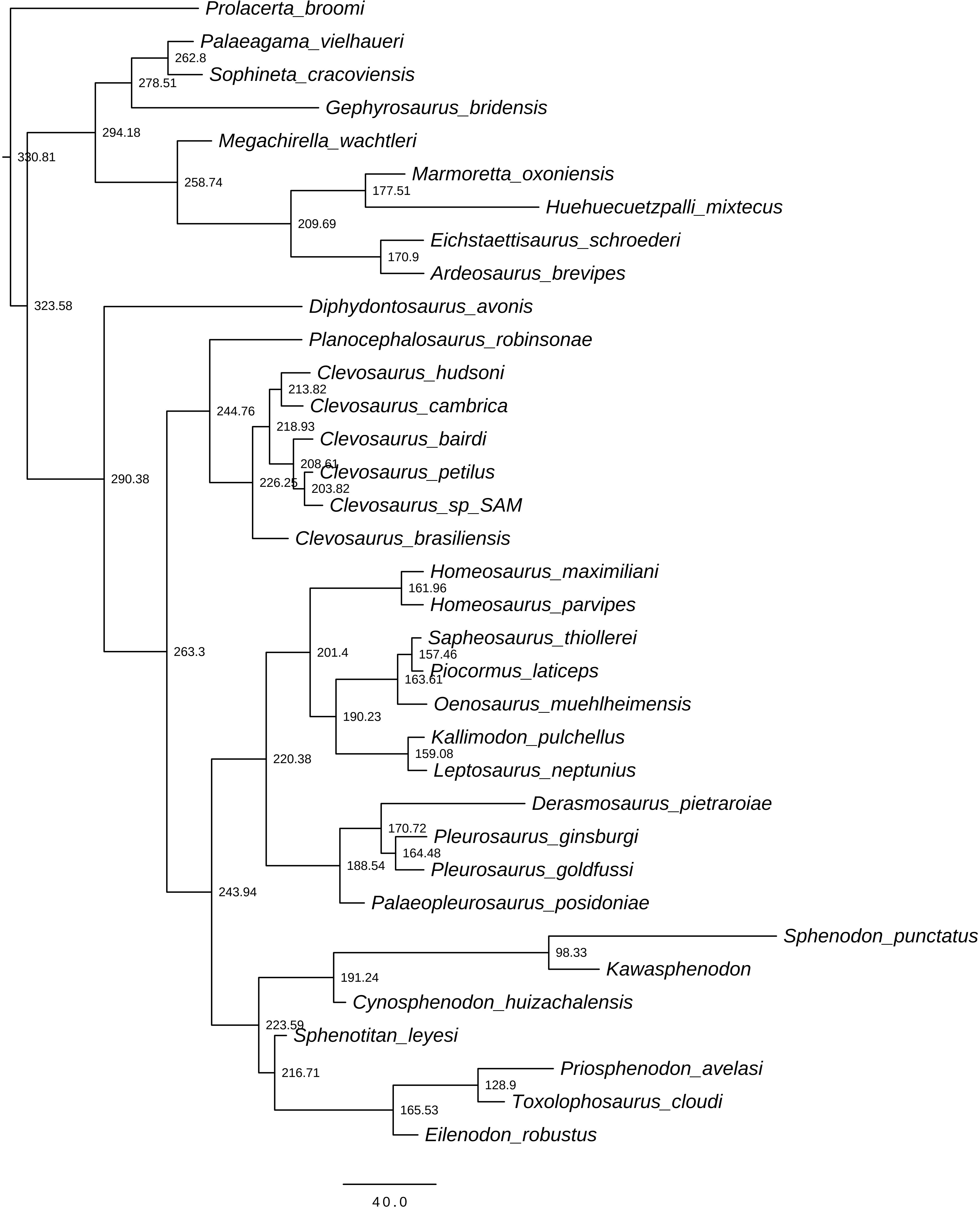
Prolacerta_broomi
Palaeagama_vielhaueri
262.8
Sophineta_cracoviensis
278.51
Gephyrosaurus_bridensis
294.18
Megachirella_wachtleri
330.81
Marmoretta_oxoniensis
258.74
177.51
Huehuecuetzpalli_mixtecus
209.69
Eichstaettisaurus_schroederi
170.9
Ardeosaurus_brevipes
323.58
Diphydontosaurus_avonis
Planocephalosaurus_robinsonae
Clevosaurus_hudsoni
213.82
Clevosaurus_cambrica
244.76
218.93
Clevosaurus_bairdi
208.61
Clevosaurus_petilus
290.38
226.25
203.82
Clevosaurus_sp_SAM
Clevosaurus_brasiliensis
Homeosaurus_maximiliani
161.96
Homeosaurus_parvipes
Sapheosaurus_thiollerei
157.46
263.3
201.4
Piocormus_laticeps
163.61
Oenosaurus_muehlheimensis
190.23
Kallimodon_pulchellus
159.08
220.38
Leptosaurus_neptunius
Derasmosaurus_pietraroiae
170.72
Pleurosaurus_ginsburgi
164.48
188.54
Pleurosaurus_goldfussi
243.94
Palaeopleurosaurus_posidoniae
Sphenodon_punctatus
98.33
Kawasphenodon
191.24
Cynosphenodon_huizachalensis
223.59
Sphenotitan_leyesi
216.71
Priosphenodon_avelasi
128.9
Toxolophosaurus_cloudi
165.53
Eilenodon_robustus
40.0
