# Supplementary material for: Sphenodontian phylogeny and the impact of model choice in Bayesian morphological clock estimates of divergence times and evolutionary rates
Source: BMC Biol. 2020 Dec 7;18:191. doi: 10.1186/s12915-020-00901-5 (PMC7720557; doi:10.1186/s12915-020-00901-5)
Supplement: Supplementary file 6 — Additional file 6. Input files including the dataset and all necessary coding (see Mr. Bayes blocks) to reproduce the analyses. [file 12915_2020_901_MOESM6_ESM.zip › InputFiles&OutputTrees/BayesCalibrated/Random/BayesCal_TK02_ln_p1_Ra/BayesCal_TK02_ln_p1_Ra_MRC.t.con.tre.pdf]

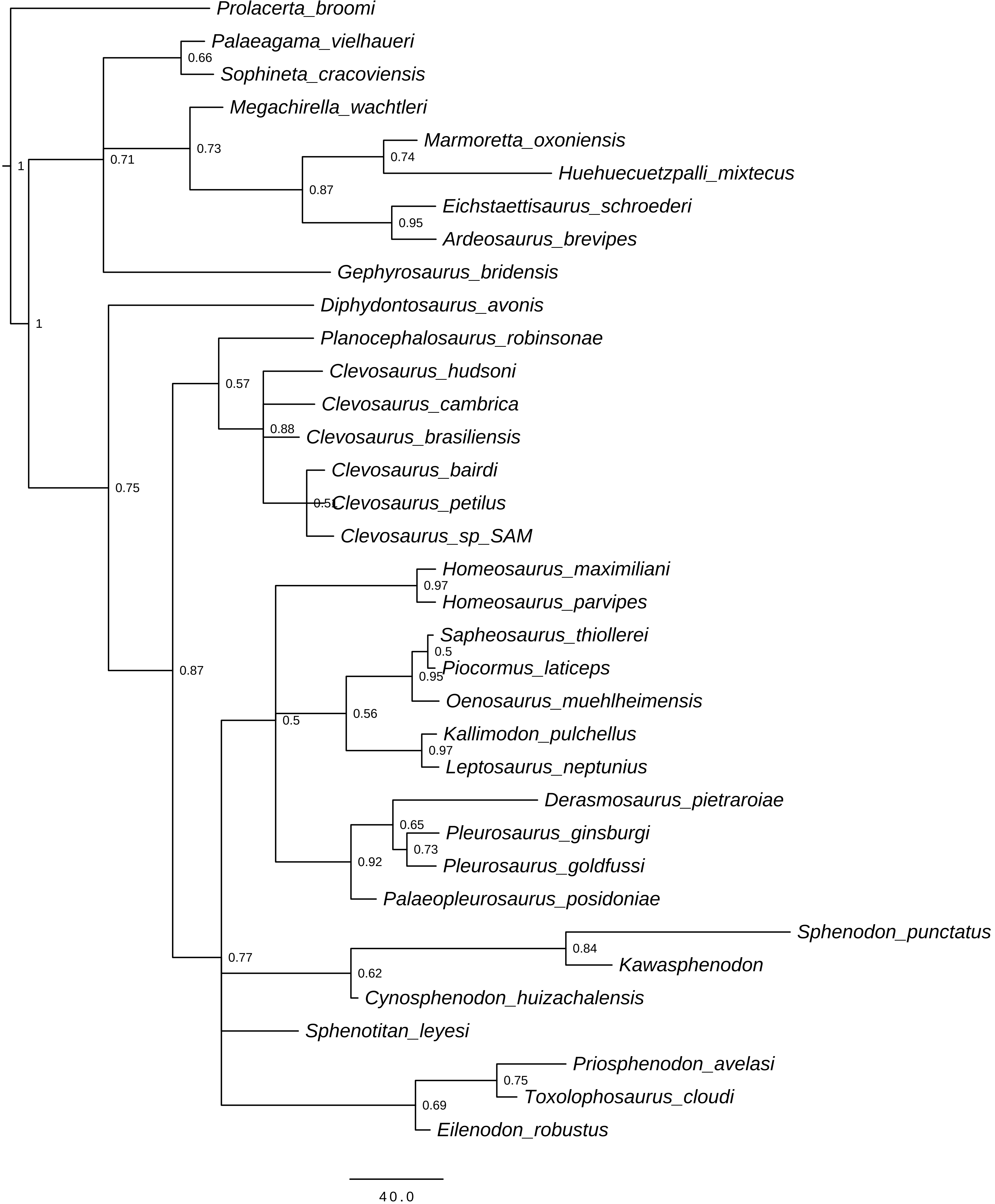
Prolacerta_broomi
Palaeagama_vielhaueri
Sophineta_cracoviensis
Megachirella_wachtleri
Marmoretta_oxoniensis
Huehuecuetzpalli_mixtecus
Eichstaettisaurus_schroederi
Ardeosaurus_brevipes
Gephyrosaurus_bridensis
Diphydontosaurus_avonis
Planocephalosaurus_robinsonae
Clevosaurus_hudsoni
Clevosaurus_cambrica
Clevosaurus_brasiliensis
Clevosaurus_bairdi
Clevosaurus_petilus
Clevosaurus_sp_SAM
Homeosaurus_maximiliani
Homeosaurus_parvipes
Sapheosaurus_thiollerei
Piocormus_laticeps
Oenosaurus_muehlheimensis
Kallimodon_pulchellus
Leptosaurus_neptunius
Derasmosaurus_pietraroiae
Pleurosaurus_ginsburgi
Pleurosaurus_goldfussi
Palaeopleurosaurus_posidoniae
Sphenodon_punctatus
Kawasphenodon
Cynosphenodon_huizachalensis
Sphenotitan_leyesi
Priosphenodon_avelasi
Toxolophosaurus_cloudi
Eilenodon_robustus
1
1
0.66
0.71
0.73
0.74
0.87
0.95
0.75
0.57
0.88
0.51
0.87
0.97
0.5
0.95
0.5
0.56
0.97
0.65
0.73
0.92
0.77
0.84
0.62
0.75
0.69
40.0
